# Genomic Insights and Plant Growth-Promoting Characterization of *Priestia megaterium* Strain 53B2, Isolated from Maize-Associated Soil in the Yaqui Valley, Mexico

**DOI:** 10.3390/plants14132081

**Published:** 2025-07-07

**Authors:** Alina Escalante-Beltrán, Pamela Helué Morales-Sandoval, Claudia Berenice González-Astorga, Amelia C. Montoya-Martínez, Edgar A. Cubedo-Ruiz, Gustavo Santoyo, Fannie Isela Parra-Cota, Sergio de los Santos-Villalobos

**Affiliations:** 1 Instituto Tecnológico de Sonora, 5 de Febrero 818 Sur, Col. Centro, Cd. Obregón C.P. 8500, Sonora, Mexico; alinaescalanteb@gmail.com (A.E.-B.); pamesandov37@gmail.com (P.H.M.-S.); claudia.gonzalez112316@potros.itson.edu.mx (C.B.G.-A.); cristina_montoya14@hotmail.com (A.C.M.-M.); 2 Campo Experimental Norman E. Borlaug, Instituto Nacional de Investigaciones Forestales, Agrícolas y Pecuarias (INIFAP), Norman E. Borlaug Km. 12, Cd. Obregón C.P. 85000, Sonora, Mexico; ecubedo@hotmail.com; 3 Instituto de Investigaciones Químico Biológicas, Universidad Michoacana de San Nicolás de Hidalgo, Morelia C.P. 58030, Michoacán, Mexico; gustavo.santoyo@umich.mx

**Keywords:** whole-genome sequencing, PGPM, taxonomic affiliation, genome annotation, genome mining

## Abstract

Strain 53B2 was isolated from a commercial maize (*Zea mays* L.) field located in the Yaqui Valley, Mexico. Its draft genome comprises 5,844,085 bp, with a G + C content of 37.5%, an N50 of 602,122 bp, an L50 of 4, and a total of 129 contigs. Genome-based taxonomic affiliation showed this strain belonged to *Priestia megaterium*. Genome annotation revealed 6394 coding DNA sequences (CDSs), organized into 332 subsystems. Among these, several CDSs were associated with traits relevant to plant growth promotion, including categories such as iron acquisition and metabolism (40 CDSs) and secondary metabolism (6 CDSs), among others. In vitro metabolic assays supported genomic predictions, confirming the strain’s ability to produce IAA, solubilize phosphate, and tolerate abiotic stress. Additionally, greenhouse trials demonstrated that inoculation with *Priestia megaterium* 53B2 significantly enhanced plant growth parameters (*p* ≤ 0.05) versus uninoculated control: stem height increased by 22.8%, root length by 35.7%, stem and root fresh weights by 39.6% and 66.1%, and stem and root dry weights by 33.7% and 44.7%, respectively. This first report on the beneficial potential of *Priestia megaterium* 53B2 highlights its potential as a sustainable bioinoculant for maize cultivation.

## 1. Introduction

Ensuring global food security is one of the greatest challenges of the 21st century. As the world population continues to grow and the effects of climate change intensify, the demand for agricultural production is expected to rise dramatically. Projections indicate that food output must increase by 70–100% by 2050 to sustain the global population [1]. In this context, cereals will remain fundamental to human nutrition, as they contribute significantly to daily caloric and protein intake [2]. Research in agriculture has changed over the decades. For example, since the 1950s, agricultural progress has been largely driven by the goal of boosting staple crop production in response to growing concerns about population growth and the capacity of food systems to meet rising demand [3]. Whole grains such as maize provide essential nutrients that contribute to addressing the global triple burden of malnutrition: undernutrition, micronutrient deficiencies, and overnutrition-related conditions, such as obesity and non-communicable diseases [4]. Maize is one of the world’s most widely cultivated crops, with global production averaging 1229.63 million tons annually [5]. Its adaptability to diverse agroecological environments (including a broad range of temperatures, altitudes, latitudes, and soil types) makes it a staple in many regions [2]. In 2023, maize production in Mexico surpassed 27.5 million metric tons, making a 3.74% increase compared to the previous year [6]. In this context, the Yaqui Valley—located in the State of Sonora, Mexico—is internationally recognized for its role in the scientific developments led by Dr. Norman E. Borlaug, which contributed to the Green Revolution between the 1960s and the 1980s [7]. Today, it remains one of the main farming regions of Mexico and one of the most intensively cultivated agricultural regions in the world, relying heavily on irrigation systems, chemical fertilizers, and improved crop varieties to produce key crops such as wheat, soybeans, maize, safflower, and sorghum [8,9]. In 2023 alone, Sonora contributed approximately 503,014 tons (1.76%) of maize to national production; however, decades of intensive cultivation have led to significant soil degradation, characterized by low organic matter content (<1.5%), high susceptibility to wind erosion due to the region’s arid climate (estimated 200 ton ha^−1^ year^−1^), and the development of saline or alkaline soil conditions. Additionally, the overuse of agrochemicals and intensive agronomic practices has reduced fertilizer efficiency, posing challenges to long-term soil health and crop productivity [10].

Maize, like many crops, is increasingly vulnerable to the combined pressures of climate change, facing both biotic stressors, such as pests and diseases, and abiotic stressors, including water scarcity, salinity, and extreme temperatures. These factors can severely impact plant physiology, leading to yield losses ranging from 50% to 82% [11]. Moreover, the adoption of high-input agricultural practices aimed at maximizing productivity has exacerbated environmental issues such as soil degradation, water shortages, eutrophication, and deforestation, while also posing potential risks to human health [12,13]. In response to the increasing challenges of sustainable global food production, especially under the pressure of climate change and population growth, there is a critical need to implement innovative and sustainable agricultural strategies. One such approach involves the use of beneficial microorganisms, particularly plant growth-promoting microorganisms (PGPMs), which play a vital role in maintaining the health and functionality of agroecosystems [14]. PGPMs support plant development by improving nutrient acquisition, mitigating environmental stress, and providing biocontrol against phytopathogens through multiple mechanisms [15]. Currently, they are widely applied as microbial inoculants, contributing directly to plant nutrition via biological nitrogen fixation, the mobilization of organic and inorganic phosphorus, and siderophore production. Additionally, they enhance growth by producing phytohormones such as indole-3-acetic acid (IAA), gibberellins, cytokinins, and the enzyme ACC deaminase, which helps modulate ethylene levels under stress conditions. Beyond these direct benefits, PGPMs are also effective in disease management; they suppress plant pathogens through competition for essential nutrients and space, induction of systemic resistance, and production of compounds like antibiotics, exopolysaccharides, hydrogen cyanide, and lytic enzymes [11,14,16]. Given their multifunctionality, the bioprospecting of new microbial strains with biotechnological applications in agriculture has become a priority, aiming to enhance productivity while minimizing agrochemical inputs and improving soil health [17,18].

In this sense, the genus *Priestia* belongs to the Bacillaceae family, characterized by Gram-positive, mostly rod-shaped bacteria. Recently reclassified, species within this genus have been previously reported as plant growth-promoting bacteria in maize, tomato, tea, wheat, rice, bean, and soybean, among others [12,19,20,21]. Thus, the present study offers a detailed genomic and functional characterization of strain 53B2, isolated from a maize (*Zea mays* L.) commercial field in the Yaqui Valley, Mexico, including genomic mining enabled the identification of genes associated with plant-growth promotion traits, which were subsequently validated through both metabolic assays and greenhouse trials.

## 2. Results

### 2.1. Morphological and Metabolic Characterization

**Figure 1:**
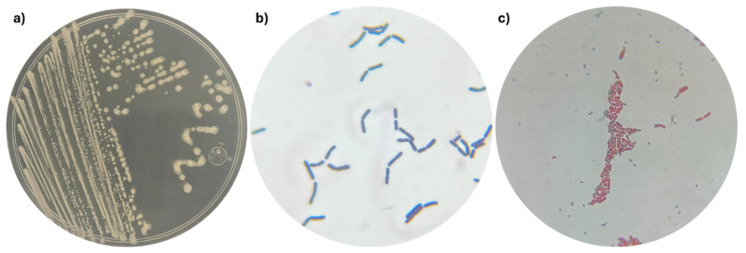
Morphology of strain 53B2: (**a**) Macroscopic morphology of the colonies; (**b**) Microscopic morphology of Gram-positive cells; (**c**) Spores, endospores, and vegetative cells of strain 53B2. Spores and endospores were stained with malachite green stain and appear green/light blue; vegetative cells are stained with safranin and appear pink/red.

**Figure 2:**
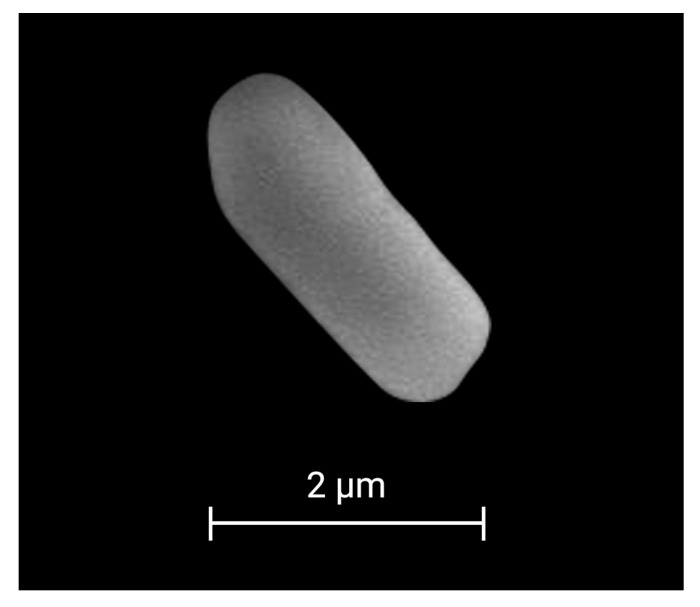
Scanning electron microscopy image of a rod-shaped cell of strain 53B2.

Strain 53B2 was isolated as one of the most abundant bacterial strains associated with maize (*Zea mays* L.) in a commercial field located in the Yaqui Valley, Mexico. Strain 53B2 showed a circular, white, flat, opaque colony, and Gram-positive, rod-shaped cells with a size of 3.97 × 1.18 µm (Figure 1 and Figure 2). Endospore staining revealed the presence of green-stained endospores within pink/red vegetative cells of strain 53B2, confirming its ability to form endospores under nutrient-limited conditions. Microscopy images showed that the endospores were predominantly located in a central position within the cells (Figure 1).

### 2.2. Genomic Analysis

**Figure 3:**
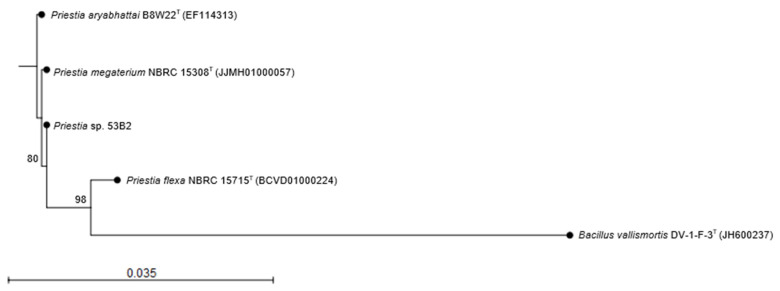
Phylogenetic relationships based on 16S rRNA gene sequences between strain 53B2 and closely related *Priestia* species: *Priestia megaterium* NBRC 15308^T^ (JJMH01000057), *P*. *aryabhattai* B8W22^T^ (EF114313), and *P*. *flexa* NBRC 15715^T^ (BCVD01000224). *Bacillus vallismortis* DV1-F-3^T^ (JH600237) was used as an outgroup. The phylogenetic tree was constructed using CLC Sequence Viewer version 8.0, employing the Jukes–Cantor distance model and the neighbor-joining method, with 1000 bootstrap replicates. The scale bar (0.035) represents the number of nucleotide substitutions per site.

After DNA extraction and whole-genome sequencing, strain 53B2’s genomic DNA resulted in a total of 776,413 paired-end reads (2 × 250 bp). The genome assembly yielded 129 contigs (≥200 bp), totaling 5,844,085 base pairs, with a G + C content of 37.5%. The assembly quality metrics included an N50 value of 602,122 bp and an L50 of 4. No plasmid sequences were identified. This Whole Genome Shotgun project has been submitted to DDBJ/ENA/GenBank and is available under the accession number JBLZXA000000000. The version reference in this study corresponds to JBLZXA010000000. Analysis of the 16S rRNA gene revealed 99.92% sequence identity with *Priestia megaterium* NBRC 15308^T^, 99.76% with *Priestia aryabhattai* B8W22^T^, and 99.04% with *Priestia flexa* NBRC 15715^T^ (Table 1). Phylogenetic reconstruction based on the 16S rRNA gene sequence, in combination with morphological features, confirmed that strain 53B2 belongs to the genus *Priestia* (Figure 3). In addition, OGRI analysis confirms the taxonomic identification of strain 53B2 as *Priestia megaterium*, due to ANI (96.23) and GGDC (76.58) values being higher than those established for species delimitation (ANI ≥ 95–96% and GGDC ≥ 70%) (Table 1).

### 2.3. Genome Annotation

**Figure 4:**
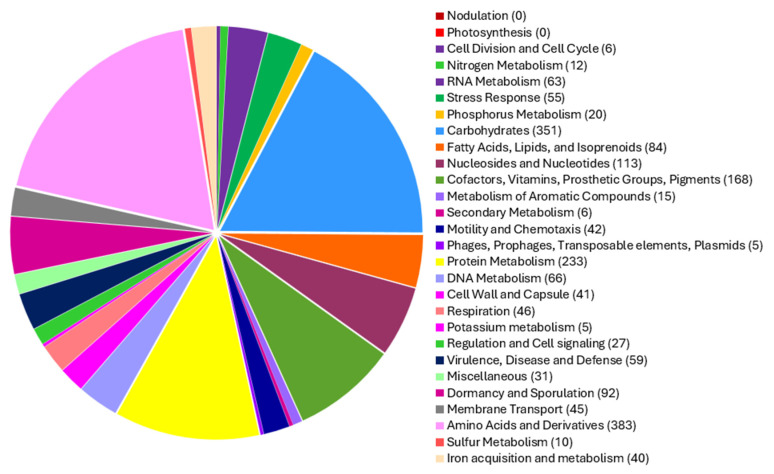
Pie chart with the distribution of subsystem categories among the CDSs of *Priestia megaterium* 53B2, generated using the RAST server (version 2.0).

**Figure 5:**
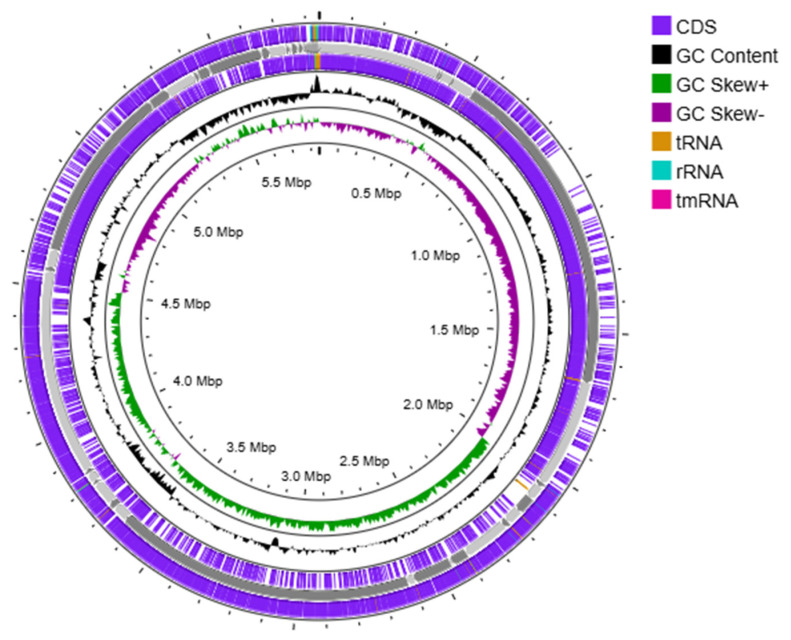
Circular chromosome map of *Priestia megaterium* 53B2, including the distribution of CDSs, tRNAs, rRNAs, and GC content skew, created through genome annotation using Prokka (version 1.2.0).

Annotation of the *Priestia megaterium* 53B2 genome using the RAST platform revealed 166 RNA genes and 6394 coding DNA sequences (CDSs), organized into 332 functional subsystems (Figure 4). Among these, the most significant subsystems were amino acids and derivatives (383 CDS), carbohydrates (351 CDS), protein metabolism (233), cofactors, vitamins, prosthetic groups and pigments (168 CDS), and nucleosides and nucleotides (113 CDS). Additionally, multiple subsystems related to plant growth promotion functions were detected. These included (i) 40 CDS involved in iron acquisition and metabolism, notably 18 CDS related to siderophore biosynthesis (molecules that sequester iron and reduce its availability to plant pathogens); (ii) 6 CDS associated with secondary metabolite production, with 4 of them involved in plant hormone synthesis; and (iii) 20 CDS involved in phosphorus metabolism. The genome also harbored genes supporting environmental stress adaptation, with 55 CDS related to stress responses, including osmotic (17 CDS) and oxidative (19 CDS) stress pathways. Complementing these results, a circular chromosome map generated using Prokka annotated 6028 CDS, along with 144 tRNA genes and 1 tmRNA (Figure 5), further confirming the genome’s coding potential and functional diversity.

### 2.4. Genome Mining

**Figure 6:**
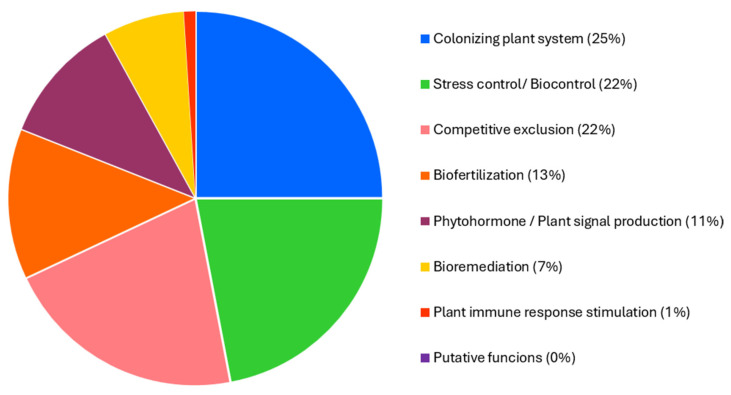
PLaBAse analysis of the genome of *Priestia megaterium* 53B2 by blastp + hmmer.

Genome analysis of *Priestia megaterium* 53B2 revealed that approximately 25% of its genetic content is related to plant colonization processes. Additionally, 22% was associated with stress management and biocontrol functions, 21% with competitive exclusion mechanisms, 13% with biofertilization capabilities, 11% with phytohormone production and plant signaling, 7% with bioremediation potential, and 1% with the stimulation of plant immune responses (Figure 6 and Table S1).

Genome mining analysis performed using the AntiSMASH 7.0 web server identified three secondary metabolites: surfactin (13% similarity), carotenoid (50%), and the siderophore schizokinen (62%). However, none of these clusters showed a similarity above the 75% threshold typically used to consider a biosynthetic gene cluster (BGC) as potentially functional or well-characterized for biocontrol activity [22]. Therefore, no putative biocontrol-related BGCs were detected in strain 53B2, suggesting a limited capacity for secondary metabolite-mediated antagonism against phytopathogens.

### 2.5. Metabolic Characterization and Biocontrol Assay

**Figure 7:**
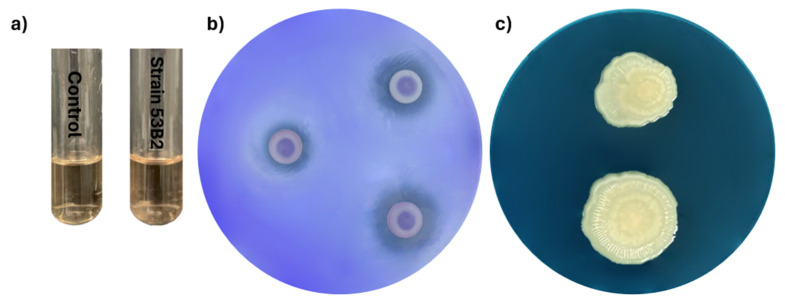
Metabolic characterization of *P*. *megaterium* 53B2: (**a**) production of IAA using Salkowski reagent; (**b**) phosphate solubilization on Pikovskaya (PVK) agar; (**c**) siderophore production on Chrome Azurol S (CAS) agar.

Metabolic assays of *Priestia megaterium* 53B2 demonstrated its potential ability to promote the growth of plants. For example, strain 53B2 was able to synthesize indole-3-acetic acid (IAA) at a concentration of 2.29 µg/mL and a phosphate solubilization efficiency of 36.83 ± 11.06%; however, the ability to produce siderophores was not observed (Figure 7). The strain also exhibited resilience at various abiotic stress conditions, showing tolerance to salinity (34.24%), water deficit (99.23%), and elevated temperatures (99.22%). Moreover, biocontrol activity against *Fusarium verticillioides*, *Curvularia* sp. H3-5 and *Fusarium* sp. H2-3 was not observed.

### 2.6. Maize Growth Promotion Assay

The inoculation of *Priestia megaterium* 53B2 in maize led to statistically significant increases (*p* ≤ 0.05) in stem height (22.84%), root length (35.72%), stem and root weight (39.59% and 66.07%, respectively), and stem and root dry weight (33.66% and 44.73%, respectively), compared to the uninoculated control (Table 2).

## 3. Discussion

Since the need for sustainable strategies in agriculture continues to grow, the search for alternative solutions to enhance crop productivity while minimizing environmental impact has become increasingly critical. In this context, bioprospecting for plant growth-promoting bacteria (PGPB) offers a promising strategy. These microorganisms employ a variety of mechanisms to enhance plant growth and protect plants from pathogens, both directly and indirectly [13].

With the advancement and widespread availability of sequencing technologies, the identification and reclassification of microbial taxa have accelerated significantly. A notable example is the recent taxonomic revision of the genus *Priestia*, a group of Gram-positive, primarily rod-shaped bacteria within the Bacillaceae family, recently reclassified by Gupta and colleagues [20]. In this sense, strain 53B2 was isolated from a commercial maize field and exhibited colony morphology typically associated with *Priestia* species: circular, white, flat, opaque colony, and Gram-positive, with rod-shaped cells (Figure 1 and Figure 2) [12,21,23,24,25,26]. For taxonomic affiliation, the 16S rRNA gene sequence was retrieved from the sequenced genome, as this gene contains both conserved and variable regions that are widely used to distinguish between bacterial genera [27,28]. Analysis of the 16S rRNA sequence (Table 1, Figure 3) provided strong evidence supporting the assignment of strain 53B2 to the genus *Priestia*. Moreover, OGRI analysis revealed that strain 53B2 belongs to the species *Priestia megaterium* (Table 1). Additionally, while plasmids are frequently observed among *Priestia* species, approximately 25% of known strains within this genus have been reported to lack plasmids [19], such as the case of *Priestia megaterium* 53B2. The ability of strain 53B2 to form endospores is consistent with the sporulation capacity commonly reported in *Priestia megaterium* strains [29,30]. Genome analysis of *Priestia megaterium* 53B2 revealed a comprehensive set of genes associated with sporulation, *spo0A*, *spo0B*, *spo0E*, *spo0F*, and *spo0M*, as well as the operons *spoIIIA*-*spoIIID*, *spoIIA*, *spoIIAB*, *spoIIB*-*spoIVFB*, along with coat protein genes, all linked to endospore development (Table S1). The presence of *spo0A*, the master regulator, and upstream phosphorelay genes (*spo0B*, *spo0F*) confirms the conserved signal transduction pathway that initiates under nutrient-limited conditions [31,32,33,34,35,36,37]. Gene *spo0E*, acting as a phosphatase, fine-tunes *spo0A* phosphorylation, integrating stress signals via σ^B^, linking environmental stress responses directly to sporulation control [33,38,39,40,41]. The identification of genes encoding coat proteins (*cotA*, *cotD*, *cotE*, *cotH*, *cotN*, *cotV*, *cotX*, *cotY*, and *cotZ*) further supports the capacity of *P*. *megaterium* 53B2 to produce structurally resilient endospores [42,43,44]. Beyond sporulation, *spo0A* also plays a pivotal role in stress resilience, controlling genes involved in survival under temperature fluctuations and oxidative pressure, particularly in soil-dwelling *Bacillus* species [32,33,36,40,43,45]. Together, the presence of a complete sporulation gene toolkit along with coat proteins suggests that *Priestia megaterium* 53B2 is well-equipped for endospore formation and stress resistance, findings corroborated by the malachite green staining results showing central endospores (Figure 1). This genomic evidence further supports strain 53B2’s classification within the spore-forming genus *Priestia* and strengthens its ecological relevance for survival under harsh environmental conditions.

The plant-associated microbiota plays a vital role in improving nutrient availability by mobilizing otherwise inaccessible resources, such as inorganic phosphate and iron, through mechanisms like solubilization, mineralization, and the secretion of iron-chelating siderophores [46]. To determine the potential of *Priestia megaterium* 53B2 in enhancing corn production, its genome was annotated and mined, and the obtained information was correlated with metabolic and functional traits. Specifically, genome analysis revealed 18 CDSs related to siderophore biosynthesis, including *Sdab*, *Sdad*, *SbsM*, *PchA*, *PchB*, *PchC*, *PchD*, *PchEPchF*, *ICM, BDH*, and *PchK*, among others [47]. Additionally, genome mining reveals that *Priestia megaterium* 53B2 possesses a sophisticated, multi-tiered iron acquisition system featuring (i) heme biosynthesis/transport (*hemABCDL*, *feuA*), (ii) ferric citrate uptake (*fecABCD*), and (iii) ferrous iron transport (*efeUOB*) (Table S1) [48,49,50,51,52]. While this genetic repertoire suggests metabolic flexibility, the absence of CAS reactivity indicates conditional silencing of siderophore production, a phenomenon observed in bacteria that prioritize alternative iron sources when available [48,49]. This finding might position *Priestia megaterium* 53B2 to study metal homeostasis, where genomic potential and phenotypic expression diverge based on environmental cues.

On the other hand, several genes related to phosphate solubilization were identified. These include *pqq* and *gdh*, which participate in the oxidation of glucose to gluconic acid (the main organic acid responsible for dissolving insoluble phosphate compounds [53,54]. Also, the presence of genes such as *phoA*, *ppx*, *phoD*, *phn*, *ppk,* and other related genes suggests the potential for organic phosphorus mineralization via the production of phosphatases; many of the carrier genes encode different enzymes, such as alkaline phosphatases, extracellular polyphosphatase, and polyphosphate kinase [55,56]. The functionality of these genes was supported by in vitro assays, where *Priestia megaterium* 53B2 demonstrated a phosphate solubilization capacity of 36.83 ± 11.06%. This trait is highly desirable in plant growth-promoting bacteria, as phosphorus is an essential macronutrient involved in numerous metabolic and physiological processes. In arid and semi-arid soils, where phosphorus availability is frequently a limiting factor for crop productivity, such microbial traits offer promising solutions for sustainable agriculture [57].

Moreover, genome annotation of *Priestia megaterium* 53B2 using the RAST server revealed five CDSs associated with auxin biosynthesis: *APRT*, *PRAI*, *IGS*, *TSa,* and *TSb*. Several genes from the tryptophan biosynthesis pathway were also identified, including *trpA*, *trpB*, *trpC*, *trpD*, *trpE*, *trpF*, *trpR*, and *trpS*, which contribute not only to the synthesis of indole-3-acetic acid (IAA) but are also involved in ethylene and ammonia enzymes in the indole-3-pyruvic acid (IPA) pathway, one of the most widespread IAA biosynthetic routes among microorganisms [47]. In this pathway, tryptophan is first converted into IPA, which is then transformed into indole-3-acetaldehyde (IAAld), and subsequently oxidized into IAA via the action of aldehyde dehydrogenases [58]. The presence and functionality of these genes were supported by metabolic assays, where *Priestia megaterium* produced 2.29 µg/mL of IAA, as determined by the Salkowski colorimetric method. This trait is particularly valuable in plant-beneficial microorganisms, as IAA plays a pivotal role in regulating key developmental processes such as cell elongation, division, root initiation, fruit development, and senescence [58,59].

Given the arid and semi-arid conditions of the Yaqui Valley, Mexico [60], the ability of *Priestia megaterium* 53B2 to tolerate abiotic stressors such as salinity, drought, and temperature fluctuations is of particular interest. The strain exhibited tolerance levels of 34.24% to salinity, 99.23% to water deficit, and 99.22% to thermal stress, respectively [61]. These phenotypic traits are likely supported by the presence of 17 CDSs related to osmotic stress identified in its genome. The genomic landscape reveals a coordinated network of stress-responsive pathways centered on redox balance, ion homeostasis, and energy metabolism. Notable genes include *betA*, *betB*, *betT*, *betC*, *betI*, *opuAA*, *opuAB*, and *opuAC*, which are involved in the synthesis and transport of osmoprotectants. Specifically, these genes facilitate the uptake and conversion of choline into glycine betaine, a well-known osmoprotectant that enables bacterial survival under high salinity and drought conditions [62,63]. The presence of the *nadABCDE* operon ensures robust NAD^+^ biosynthesis, critical for maintaining redox poise under oxidative stress [64], while its linkage to the *mnhABCDEFG* operon (encoding a multisubunit NA^+^/H^+^ antiporter) suggests coupled NADH recycling and pH homeostasis during osmotic stress [65,66]. Sulfur assimilation via *cys* genes (*cysACEGHIJKSTW*) further supports resilience, as cysteine is a precursor for glutathione (GSH) and iron–sulfur (Fe-S) clusters required for oxidative defense [67]. Notably, the *clpBCEPXR* protease system, regulated by NAD^+^-dependent sirtuins, links protein quality control to metabolic status, degrading misfolded proteins during heat shock [68]. Further work should probe the transcriptional coordination of these systems under combinational stresses (e.g., oxidative + osmotic) to elucidate regulatory checkpoints.

Genome mining of *P*. *megaterium* 53B2 on AntiSMASH revealed low similarity scores (<70%) to known biosynthetic gene clusters, including schizokinen (62%), carotenoid (50%), and surfactin (13%). These values suggest that although homologous sequences exist, the cluster may be incomplete, divergent, or non-functional, and such variation is not uncommon at the intraspecies level [69,70]. *Priestia* species are widely recognized for their biocontrol potential through a variety of mechanisms associated with plant protection, including the production of (i) organic volatile compounds (VOCs), such as polyketones [71]; (ii) lipopeptides like surfactin and iturin [25]; (iii) siderophores such as schizokinen [72]; and (iv) organic compounds like phenazine [73]. Nevertheless, the results of genomic and in vitro assays indicate that strain 53B2, despite belonging to a genus known for its plant-beneficial properties, lacks biocontrol capacity.

Thus, based on genome mining and metabolic tests, *Priestia megaterium* 53B2 demonstrated significant improvements (*p* ≤ 0.05) in stem height by 22.8%, root length by 35.7%, stem and root fresh weights by 39.6% and 66.1%, and stem and root dry weights by 33.7% and 44.7%, respectively. These effects suggest the presence of multiple plant growth-promotion traits in this strain. Genomic analysis supported this, revealing genes associated with auxin biosynthesis, phosphate solubilization, and abiotic stress tolerance. These genomic features, along with confirmed IAA production and phosphate solubilization in vitro, likely underpin the observed improvements in plant biomass and morphology.

Although functional validation at the transcriptomic or metabolomic level is still required, the simultaneous enhancement of both shoot and root biomass suggests a balanced and potentially adaptive plant growth-promotion mechanism. This differentiates *P. megaterium* 53B2 from strains that tend to favor root development over aerial growth.

To contextualize these findings, other *Priestia* strains have shown similar traits. For example, *P. megaterium* HY-01 promoted *Centella asiatica* leaf elongation via auxin production, and strain ZS-3 enhanced cotton biomass [74,75]. However, *P. megaterium* 53B2 showed dry weight increment (33.66% stem, 44.73% root), suggesting enhanced nutrient transporters, which parallels *P*. *megaterium* BP-R2’s IAA-mediated root architecture modifications but with a more balanced shoot–root enhancement, diverging from root-prioritizing strains like *P. aryabhattai* C1-9 [76,77,78]. The observed growth promotion likely stems from *Priestia megaterium* 53B2’s multifaceted PGP traits. Auxin biosynthesis, a hallmark of *P*. *megaterium*, such as HY-01’s tryptophan-dependent IAA pathway, may explain root elongation and stem cell expansion. Concurrent phosphate solubilization, documented in ZS-3 through gene expression, could drive biomass accumulation. Notably, the performance of *P*. *megaterium* 53B2 surpasses typical *P. megaterium* strains (e.g., 20–50% dry weight gains), potentially due to its origin in an intensive maize agroecosystem, which may have been selected for genomic adaptations related to nutrient acquisition and stress tolerance (for example, *pqq*-like operons).

Isolated from maize fields in the Yaqui Valley, *P*. *megaterium* 53B2 may offer valuable support in addressing two regional agricultural challenges: fertilizer dependence and climate volatility. Its performance under stress conditions, along with its genomic potential, supports its use as a versatile bacterial inoculant candidate for nutrient-poor or saline soils. Nonetheless, further studies are needed to clarify the precise mechanisms driving its plant-growth-promoting effects and to evaluate its efficacy under field conditions.

## 4. Materials and Methods

### 4.1. Bacterial Isolation and Cultivation

Strain 53B2 was isolated from the soil of a maize (*Zea mays* L.) commercial field located in the Yaqui Valley, Mexico (coordinates 27° 25′ 38.3′′ N, 110° 06′ 27.8′′ W). To isolate this strain, a composite soil sampling weighing 10 kg was collected from a one-hectare area, comprising ten subsamples. Then, 10 g were mixed with 90 mL of sterile distilled water and subjected to serial dilutions up to 10^−4^. Subsequently, 100 µL of each dilution was inoculated, in triplicate, onto Petri dishes containing nutrient agar (NA) and incubated at 28 °C for 48 h. Morphological traits, such as colony form, pigmentation, elevation, opacity, and cellular shape, were used to identify and describe one of the most abundant bacterial strains, designated as 53B2. To obtain microscopic images, strain 53B2 was cultured in NB at 30 °C for 24 h and subsequently fixed with 1% formaldehyde. The samples were then dehydrated through a graded ethanol series. Microscopic observations were performed using a JSM-6400 scanning electron microscope (JEOL, Peabody, MA, USA). The presence of endospores was assessed using the Schaeffer–Fulton staining method [79,80]. This strain was preserved in nutrient broth (NB) with 30% glycerol at −80 °C and is currently stored in the Culture Collection of Native Soil and Endophytic Microorganisms (itson.mx/colmena, accessed on 1 April 2025) [13].

### 4.2. Genomic Analysis

Examining the genomic background of strain 53B2, particularly regarding traits advantageous for agriculture, started with obtaining high-quality DNA from a freshly cultured sample grown in NB for 24 h at 30 °C with constant agitation at 120 rpm, reaching a concentration of 1 × 10^8^ CFU/mL. Then, 40 µL of the cellular suspension was treated with 120 µL of TE buffer containing lysozyme (0.1 mg/mL final concentration) and RNAse A (0.1 mg/mL, ITW Reagents, Barcelona, Spain), followed by incubation at 37 °C for 25 min. Next, proteinase K (0.1 mg/mL, VWR Chemicals, Solon, OH, USA) and 0.5% SDS (v/v, Sigma-Aldrich, St. Louis, MO, USA) were added, and the mixture was incubated at 65 °C for 5 min. DNA purification was performed using SPRI beads, and the resulting DNA was eluted in EB buffer (10 mM Tris-HCl, pH 8.0). DNA concentration (≥20 ng/µL) and yield (≥1 µg) were determined using the Quant-iT dsDNA HS kit (ThermoFisher Scientific, Waltham, MA, USA) and read on an Eppendorf AF2200 plate reader (Eppendorf UK Ltd., Stevenage, Hertfordshire, UK), followed by appropriate dilution [81].

For sequencing, libraries were prepared from the high-quality DNA using the Nextera XT Library Prep Kit (Illumina, San Diego, CA, USA) with modifications to the manufacturer’s protocol, including doubling the input DNA and extending the PCR elongation step to 45 s. Quantification and library preparation were automated using a Hamilton Microlab STAR system (Hamilton Bonaduz AG, Bonaduz, Switzerland) [82]. Sequencing was carried out using a 2 × 250 bp paired-end protocol on the Illumina NovaSeq 6000 platform. Raw reads were quality-filtered and trimmed using Trimmomatic version 0.30, applying a sliding window approach with a quality threshold of 15. A de novo assembly of the genome was generated with SPAdes version 3.15.4, using the “careful” parameter for error correction in reads [83].

The resulting contigs were organized using Mauve Contig Mover version 2.4.0 [84], referencing the *Priestia megaterium* ATCC 14581^T^ genome (GenBank: GCA_006094495.1), showing 99.92% similarity and 100% completeness based on the 16S rRNA gene sequence, which was submitted to the EzBioCloud database [85] to identify the closest phylogenetic relatives, using the established species delimitation threshold of >98.7% similarity. The 16S rRNA gene sequence of strain 53B2 was submitted to the NCBI GenBank under accession number PV037199. A phylogenetic tree based on the 16S rRNA gene was generated using the neighbor-joining method in CLC Sequence Viewer version 8.0 (Qiagen, Aarhus, Denmark), employing *Bacillus vallismortis* DV1-F-3^T^ (GenBank: JH600237) as the outgroup. To determine the species-level classification of strain 53B2, its genome was analyzed in comparison with those of the closest related strains (with >98.7% 16S rRNA gene sequence similarity), using overall genome relatedness indices (OGRI), such as average nucleotide identity (ANI), calculated using the OrthoANI algorithm, ANIb using the BLAST+ version 2.2.29 algorithm, ANIm by the MUMmer version 3.0 algorithm, and digital DNA-DNA hybridization, estimated through the Genome-to-Genome Distance Calculator (GGDC) version 3.0 via BLAST+ [86,87,88,89,90,91]. Finally, plasmid sequences were identified using PlasmidFinder 2.0 [92].

### 4.3. Genome Annotation

Genome annotation for strain 53B2 was carried out using the Rapid Annotation Using Subsystem Technology (RAST) server version 2.0 (https://rast.nmpdr.org/), employing the RASTk pipeline integrated within the PathoSystem Resources Integration Center (PATRIC) platform (accessed on 13 February 2025) [93]. In addition, the Proksee platform (https://proksee.ca/, accessed on 13 February 2025) [94], which utilizes Rapid Prokaryotic Genome Annotation (Prokka) [95], was used to construct a circular chromosome map of strain 53B2, illustrating coding sequences (CDSs), tRNAs, rRNAs, and GC skew.

### 4.4. Genome Mining

Plant growth-promoting traits were inferred using the PGPT-Pred tool available in PLaBAse (version 1.02) (https://plabase.cs.uni-tuebingen.de/pb/plabase.php, accessed on 24 March 2025) [96]. For this analysis, the protein sequence file obtained from genome annotation using RAST was uploaded to the PGPT-Pred interface to detect genetic elements in strain 53B2. Additionally, to explore biosynthetic gene clusters (BGCs) potentially involved in biocontrol, the genome of strain 53B2 was analyzed through AntiSMASH version 7.0 (https://antismash.secondarymetabolites.org, accessed on 19 February 2025), using the “relaxed” detection settings. This approach allowed the identification of diverse biosynthetic pathways related to secondary metabolite production, including those coding for non-ribosomal peptide synthetases (NRPSs), polyketide synthases (PKSs), types I and II, and ribosomally synthesized and post-translationally modified peptides (RiPP) such as lanthipeptides, lasso peptides, sactipeptides, and thiopeptides [22].

### 4.5. Metabolic Characterization

The metabolic characterization of strain 53B2 related to plant growth promotion was carried out to validate putative functions identified by genome mining. Thus, indole-3-acetic acid (IAA) production was assessed by inoculating 1 × 10^4^ CFU into 10 mL of nutrient broth (NB) enriched with 100 ppm of L-tryptophan, in triplicate [97]. Cultures were incubated at 30 ± 2 °C for five days with constant shaking at 120 rpm. After incubation, 1 mL of each culture was centrifuged at 13,000 rpm for 10 min. Then, 100 µL of the resulting supernatant was mixed with 200 µL of Salkowski reagent, incubated in the dark at room temperature for 30 min, and the absorbance was measured at 530 nm using a BioTek ELx800 Absorbance™ Microplate Reader (BioTek Instruments, Winooski, VT, USA). IAA concentration was calculated based on a standard calibration curve. Phosphate solubilization ability was evaluated using Pikovskaya (PVK) agar [98], where Petri dishes were inoculated with 1 × 10^4^ CFU of the studied strain, in triplicate, and incubated at 28 °C for seven days. The formation of a clear halo around colonies indicated positive solubilization. Phosphate solubilization efficiency (PSE) was calculated using the formula %PSE = $(\frac{HD-CD}{CD})\times 100$, where HD is the halo diameter including the bacteria and CD is the bacterial colony diameter. Siderophore production was determined using Chrome Azurol S (CAS) agar [99]. Inoculations of 1 × 10^4^ CFU in triplicate, followed by incubation at 28 °C for seven days. A yellow-to-orange halo around the colony indicated positive siderophore activity. Siderophore production efficiency (SPE) was calculated as %SPE = $(\frac{HD-CD}{CD})\times 100$, as mentioned before. Abiotic stress tolerance was also assessed by inoculating 1 × 10^4^ CFU of strain 53B2 on Falcon tubes with nutrient broth (NB) supplemented with (i) 5% sodium chloride (68 dS/m) for salt stress and (ii) 10% polyethylene glycol 6000 (PEG 6000; −0.84 MPa) to simulate water deficit, both incubated at 28 °C and 120 rpm for three days [100]; the control treatment did not include sodium chloride or polyethylene glycol. For heat stress, the studied strain was inoculated on Falcon tubes with NB and incubated at 43.5 °C and 120 rpm for three days. Control tubes were incubated at 28 °C for three days [101]. Each stress test was conducted in triplicate. Stress tolerance (ST) was expressed as a percentage using the formula %ST = $(100-\left(\frac{BGs-BG}{BG}\right))\times 100$, where BGs means the colony-forming units obtained under stress conditions and BG represents the colony-forming units obtained in the control treatment [102].

### 4.6. Biocontrol In Vitro Assay

Confrontations of *Priestia megaterium* 53B2 against *Fusarium verticillioides*, *Curvularia* sp. H3-5, and *Fusarium* sp. H2-3 were carried out. For each phytopathogen, the assay was performed using Petri dishes containing PDA. In the center, a circle with a diameter of 0.5 cm was sown from a dish with fresh mycelium; two slices of bacterial biomass were taken and placed at two equidistant points, with 1 cm of separation from the edge of the plate. Incubation was for five days at 30 °C [12,103]. The assays were conducted with three independent replicates, and control treatments were only inoculated with the phytopathogens.

### 4.7. Maize Growth Promotion Assay

To evaluate the interaction between maize plants and strain 53B2, a greenhouse experiment was conducted. Twenty-four maize seeds (var. Hipopótamo) were sown in a forest tray with cavities, 4.7 cm in diameter, filled with commercial GTX PRO-MIX substrate (PRO-MIX, Québec, QC, Canada). Strain 53B2 was grown in a 250 mL Erlenmeyer flask containing 100 mL of NB and incubated at 30 °C for 48 h with constant agitation at 120 rpm. Following incubation, the bacterial culture was centrifuged at 3600 rpm for 10 min. The pellet was washed three times with sterile water and then resuspended in 100 mL of sterile water. The optical density of the final suspension was adjusted to 0.5 at 630 nm, corresponding to 8.5 × 10^6^ CFU/mL. Each maize seed was inoculated with 3 mL of the bacterial suspension, while the control group was treated with 3 mL of sterile distilled water. After three weeks, plant growth parameters were measured, including stem height, root length, and both root and stem dry and fresh biomass [104].

### 4.8. Statistical Analysis

All statistics were performed using STATGRAPHICS Plus version 5.1. Data were analyzed through one-way analysis of variance (ANOVA), considering differences significant at *p* ≤ 0.05.

## 5. Conclusions

The bioprospection of plant growth-promoting bacteria (PGPB) through integrative genomic and metabolic approaches represents a valuable strategy for identifying effective microbial inoculants aimed at fostering sustainable agriculture. *Priestia megaterium* strain 53B2 exhibited a diverse repertoire of genes associated with plant growth promotion. These genetic features were confirmed through targeted metabolic assays and validated in planta under greenhouse trials, showing a significant positive impact on maize development. The integration of genome mining with phenotypic and plant interaction studies provides a robust framework for selecting microbial candidates with high agronomic potential. This study constitutes the first report on the plant-beneficial potential of *P. megaterium* 53B2 in maize. However, further research on modes of action under field conditions is essential to ensure the successful implementation of this strain in sustainable crop production systems.

## Figures and Tables

**Table 1:** 16S rRNA-based similarity, ANIb, ANIm, OrthoANI, and Genome-to-Genome Distance Calculator (GGDC) values of closely related species strain 53B2.

Taxon Name	Strain	GenBank Accession Number	16S Similarity (%)	Strain	ANIb	ANIm	OrthoANI	GGDC (Formula 2)
*Priestia megaterium*	NBRC 15308^T^	JJMH01000057	99.92	ATCC 14581^T^	95.49	96.7	96.51	76.58
*Priestia aryabhattai*	B8W22^T^	EF114313	99.76	B8W22^T^	94.78	95.7	95.67	76.01
*Priestia flexa*	NBRC 15715^T^	BCVD01000224	99.04	NBRC 15715^T^	73.43	84.62	74.43	0

^T^ = type strain.

**Table 2:** Maize plants’ growth promotion by the inoculation of *Priestia megaterium* 53B2.

	Uninoculated Maize (Control)	Inoculated Maize (*Priestia megaterium* 53B2)	Increment vs. Control (%)
Stem height (cm)	10.55 ± 1.1	12.96 ± 1.27 *	22.8%
Root length (cm)	9.07 ± 1.17	12.31 ± 2.25 *	35.2%
Stem weight (mg)	418.8 ± 80	584.60 ± 70 *	39.6%
Root weight (mg)	313.6 ± 140	520.80 ± 80 *	66.1%
Stem dry weight (mg)	52.91 ± 14	70.72 ± 9 *	33.7%
Root dry weight (mg)	54.52 ± 0.15	78.91 ± 0.09 *	44.7%

Asterisks (*) indicate statistically significant differences between inoculated vs. uninoculated treatments. Means (*n* = 24).

## Data Availability

The draft genome sequence has been deposited in DDBJ/ENA/GenBank under accession number JBLZXA000000000. The version described in this paper is the first version, JBLZXA010000000, under BioProject number PRJNA1080047 and BioSample number SAMN45897227.

## Supplementary Materials

The following supporting information can be downloaded at: https://www.mdpi.com/article/10.3390/plants14132081/s1, Table S1: Features of strain 53B2 genome by PLaBAse.
