# Supplementary material for: Genomic Insights and Plant Growth-Promoting Characterization of Priestia megaterium Strain 53B2, Isolated from Maize-Associated Soil in the Yaqui Valley, Mexico
Source: Plants (Basel). 2025 Jul 7;14(13):2081. doi: 10.3390/plants14132081 (PMC12251681; doi:10.3390/plants14132081)
Supplement: Supplementary file 1 [file plants-14-02081-s001.zip › plants-3705302-supplementary.pdf]

Genomic Insights and Plant Growth-Promoting Characterization of *Priestia megaterium* Strain 53B2, Isolated from Maize-Associated Soil in the Yaqui Valley, Mexico

Alina Escalante-Beltrán [1], Pamela Helué Morales-Sandoval [1], Claudia Berenice González-Astorga [1], Amelia C. Montoya-Martínez [1], Edgar A. Cubedo-Ruiz [2], Gustavo Santoyo [3], Fannie Isela Parra-Cota [2,*] and Sergio de los Santos-Villalobos [1,*]

[1] Instituto Tecnológico de Sonora, 5 de Febrero 818 Sur, Col. Centro, Cd. Obregón C.P. 8500, Sonora, Mexico; alinaescalanteb@gmail.com (A.E.-B.); pamesandov37@gmail.com (P.H.M.-S.); claudia.gonzalez112316@potros.itson.edu.mx (C.B.G.-A.); cristina_montoya14@hotmail.com (A.C.M.-M.)

[2] Campo Experimental Norman E. Borlaug, Instituto Nacional de Investigaciones Forestales, Agrícolas y Pecuarias (INIFAP), Norman E. Borlaug Km. 12, Cd. Obregón C.P. 85000, Sonora, Mexico; ecubedo@hotmail.com

[3] Instituto de Investigaciones Químico Biológicas, Universidad Michoacana de San Nicolás de Hidalgo, Morelia C.P. 58030, Michoacán, Mexico; gustavo.santoyo@umich.mx

* Correspondence: parra.fannie@inifap.gob.mx (F.I.P.-C.); sergiodelossantos@itson.edu.mx (S.d.l.S.-V.)

Table S1. Features of strain *Priestia megaterium* 53B2 genome by PLaBAse.

Level 1	Level 2	Level 3	Level 4	Level 5	Level 6	Freq
Direct Effects	Biofertilization	Carbon Dioxid Fixation	CO2 Fixation - Alternative Pathways	CO2 Fixation - Phosphoenolpyruvate Carboxylase Biosynthesis	ppc->PGPT0001165	1
Direct Effects	Biofertilization	Carbon Dioxid Fixation	CO2 Fixation - Alternative Pathways	CO2 Fixation - Reductive Citric Acid Cycle	icd->PGPT0001170	1
Direct Effects	Biofertilization	Carbon Dioxid Fixation	CO2 Fixation - Alternative Pathways	CO2 Fixation - Reductive Citric Acid Cycle	korA|oorA|OforA->PGPT0001175	1
Direct	Biofertiliz	Carbon Dioxid	CO2 Fixation -	CO2 Fixation -	korB|oorB|OforB-	1

Effects	ation	Fixation	Alternative Pathways	Reductive Citric Acid Cycle	>PGPT0001180	
Direct Effects	Biofertilization	Carbon Dioxid Fixation	CO2 Fixation - Alternative Pathways	Photosynthetic CO2 Fixation	cynT|can->PGPT0002415	2
Direct Effects	Biofertilization	Carbon Dioxid Fixation	CO2 Fixation - RuBisCo	CO2 Fixation - RuBisCo Biosynthesis	rbcL|cbbL->PGPT0001150	1
Direct Effects	Biofertilization	Carbon Dioxid Fixation	CO2 Fixation - RuBisCo	CO2 Fixation - RuBisCo Biosynthesis Regulation	cbbR|cmpR|ndhR->PGPT0001160	12
Direct Effects	Biofertilization	Iron Acquisition	Iron Acquisition-Hemophores	Hemophores-Heme|Hemin Utilization	hmoA->PGPT0003630	1
Direct Effects	Biofertilization	Iron Acquisition	Iron Acquisition-Hemophores	Hemophores-SiroHeme	cysG->PGPT0003690	1
Direct Effects	Biofertilization	Iron Acquisition	Iron Acquisition-Hemophores	Hemophores-SiroHeme	hemA->PGPT0003650	1
Direct Effects	Biofertilization	Iron Acquisition	Iron Acquisition-Hemophores	Hemophores-SiroHeme	hemB->PGPT0003655	1
Direct Effects	Biofertilization	Iron Acquisition	Iron Acquisition-Hemophores	Hemophores-SiroHeme	hemC->PGPT0003660	1
Direct Effects	Biofertilization	Iron Acquisition	Iron Acquisition-Hemophores	Hemophores-SiroHeme	hemD->PGPT0003665	2
Direct Effects	Biofertilization	Iron Acquisition	Iron Acquisition-Hemophores	Hemophores-SiroHeme	hemL->PGPT0003680	2
Direct Effects	Biofertilization	Iron Acquisition	Iron Acquisition-Hemophores	Hemophores-SiroHeme	sirA|ylnD|cysG|cobA->PGPT0003685	3
Direct Effects	Biofertilization	Iron Acquisition	Iron Acquisition-Iron Homeostasis	Iron Homeostasis-Fmn|Dmk|Ppl|Ndh|Eet System	fmnA|ecfT->PGPT0004015	1
Direct Effects	Biofertilization	Iron Acquisition	Iron Acquisition-Iron Homeostasis	Iron Homeostasis-Fmn|Dmk|Ppl|Ndh|Eet System	ndh->PGPT0004020	3

Direct Effects	Biofertilization	Iron Acquisition	Iron Acquisition-Iron Homeostasis	Iron Homeostasis-Fmn|Dmk|Ppl|Ndh|Eet System	nosX|apbE|yojL|fmnB->PGPT0000430	2
Direct Effects	Biofertilization	Iron Acquisition	Iron Acquisition-Iron Homeostasis	Iron Homeostasis-FoxABCD	coxC|ctaE->PGPT0004035	3
Direct Effects	Biofertilization	Iron Acquisition	Iron Acquisition-Iron Homeostasis	Iron Homeostasis-FoxABCD	foxA|coxA|ctaD->PGPT0004025	3
Direct Effects	Biofertilization	Iron Acquisition	Iron Acquisition-Iron Homeostasis	Iron Homeostasis-FoxABCD	foxB|coxB|ctaC->PGPT0004030	3
Direct Effects	Biofertilization	Iron Acquisition	Iron Acquisition-Iron Homeostasis	Iron Homeostasis-Related Proteins	dps|dpsA->PGPT0004055	2
Direct Effects	Biofertilization	Iron Acquisition	Iron Acquisition-Other transport Systems	Iron Acquisition-BacteriOferritin-Associated Ferredoxin	bfd|yheA->PGPT0003975	1
Direct Effects	Biofertilization	Iron Acquisition	Iron Acquisition-Other transport Systems	Iron Acquisition-Iron Uptake Regulation	fur|furB|zur->PGPT0003880	1
Direct Effects	Biofertilization	Iron Acquisition	Iron Acquisition-Other transport Systems	Iron Acquisition-Iron Uptake Regulation	pmrA->PGPT0003915	3
Direct Effects	Biofertilization	Iron Acquisition	Iron Acquisition-Other transport Systems	Iron Acquisition-Iron Uptake Regulation	troR->PGPT0003890	1
Direct Effects	Biofertilization	Iron Acquisition	Iron Acquisition-Other transport Systems	Iron Acquisition-Lipoic Acid Chelators	lipA->PGPT0003935	1
Direct Effects	Biofertilization	Iron Acquisition	Iron Acquisition-Other transport Systems	Iron Acquisition-Lipoic Acid Chelators	lipL->PGPT0003940	1
Direct Effects	Biofertilization	Iron Acquisition	Iron Acquisition-Other transport Systems	Iron Acquisition-Related Proteins-BaeSR System	baeS->PGPT0003985	1
Direct Effects	Biofertilization	Iron Acquisition	Iron Acquisition-Other transport Systems	Iron Acquisition-Related Proteins Ferritin	bfr->PGPT0003965	1
Direct Effects	Biofertilization	Iron Acquisition	Iron Acquisition-Other transport Systems	Iron Acquisition-Related Proteins	ftnA|ftn->PGPT0003970	1

				Ferritin		
Direct Effects	Biofertilization	Iron Acquisition	Iron Acquisition-Other transport Systems	Iron transport-Catechol Related Ferric Citrate transport	fecB|htsA->PGPT0003800	1
Direct Effects	Biofertilization	Iron Acquisition	Iron Acquisition-Other transport Systems	Iron transport-Catechol Related Ferric Citrate transport	fecC|htsB->PGPT0003805	2
Direct Effects	Biofertilization	Iron Acquisition	Iron Acquisition-Other transport Systems	Iron transport-Catechol Related Ferric Citrate transport	fecD|htsC->PGPT0003810	2
Direct Effects	Biofertilization	Iron Acquisition	Iron Acquisition-Other transport Systems	Iron transport-Catechol Related Ferric Citrate transport	fecE->PGPT0003815	1
Direct Effects	Biofertilization	Iron Acquisition	Iron Acquisition-Other transport Systems	Iron transport-FeuABC|YusV-transport Complex	feuA|yvrC|ABC FEVS|fatB->PGPT0003765	7
Direct Effects	Biofertilization	Iron Acquisition	Iron Acquisition-Other transport Systems	Iron transport-FeuABC|YusV-transport Complex	feuB|feuC|chuU|yfhA|hmuU|ABC FEVP|fatC|fatD->PGPT0003770	7
Direct Effects	Biofertilization	Iron Acquisition	Iron Acquisition-Other transport Systems	Iron transport-FeuABC|YusV-transport Complex	yusV|ABC FEVA|feuD->PGPT0003760	4
Direct Effects	Biofertilization	Iron Acquisition	Iron Acquisition-Other transport Systems	Iron transport-Iron II transport System	efeB->PGPT0003720	1
Direct Effects	Biofertilization	Iron Acquisition	Iron Acquisition-Other transport Systems	Iron transport-Iron II transport System	efeO->PGPT0003715	1
Direct Effects	Biofertilization	Iron Acquisition	Iron Acquisition-Other transport Systems	Iron transport-Iron II transport System	efeU|FTR|FTH1->PGPT0003710	1
Direct Effects	Biofertilization	Iron Acquisition	Iron Acquisition-Other transport Systems	Iron transport-Iron II transport System	feoA->PGPT0003695	2
Direct	Biofertiliz	Iron Acquisition	Iron Acquisition-Other	Iron transport-Iron II	feoB->PGPT0003700	5

Effects	ation		transport Systems	transport System		
Direct Effects	Biofertiliz ation	Iron Acquisition	Iron Acquisition-siderophores	siderophores-Acinetoferrin Biosynthesis	acbB->PGPT0003152	1
Direct Effects	Biofertiliz ation	Iron Acquisition	Iron Acquisition-siderophores	siderophores-Bacillibactin Metabolism	mta|ywnD->PGPT0003175	1
Direct Effects	Biofertiliz ation	Iron Acquisition	Iron Acquisition-siderophores	siderophores-Bacillibactin Metabolism	ymfI|fabG|efpI->PGPT0003180	14
Direct Effects	Biofertiliz ation	Iron Acquisition	Iron Acquisition-siderophores	siderophores-Bacillibactin transport	blt->PGPT0003185	5
Direct Effects	Biofertiliz ation	Iron Acquisition	Iron Acquisition-siderophores	siderophores-Bacillibactin transport	ydfK|yqgA->PGPT0003195	1
Direct Effects	Biofertiliz ation	Iron Acquisition	Iron Acquisition-siderophores	siderophores-Bisucaberin Biosynthesis	bsbB->PGPT0031170	1
Direct Effects	Biofertiliz ation	Iron Acquisition	Iron Acquisition-siderophores	siderophores-Coprogen Biosynthesis	hemN|hemZ->PGPT0003200	2
Direct Effects	Biofertiliz ation	Iron Acquisition	Iron Acquisition-siderophores	siderophores-Cyclooctatin Metabolism	cotB3->PGPT0003075	1
Direct Effects	Biofertiliz ation	Iron Acquisition	Iron Acquisition-siderophores	siderophores-DesFerrioxAmine-Biosynthesis	ddc|dfoJ|desA->PGPT0013725	1
Direct Effects	Biofertiliz ation	Iron Acquisition	Iron Acquisition-siderophores	siderophores-Enterobactin|Enteroch elin Metabolism	acrR|smeT->PGPT0003255	2
Direct Effects	Biofertiliz ation	Iron Acquisition	Iron Acquisition-siderophores	siderophores-Enterobactin|Enteroch elin Metabolism	fes|cbsF|cbsH->PGPT0003235	1
Direct	Biofertiliz	Iron Acquisition	Iron Acquisition-	siderophores-	fhuF->PGPT0003315	1

Effects	ation		siderophores	Ferrichrome|FerrioxAmine Metabolism		
Direct Effects	Biofertilization	Iron Acquisition	Iron Acquisition-siderophores	siderophores-Petrobactin Biosynthesis	asbA->PGPT0003545	1
Direct Effects	Biofertilization	Iron Acquisition	Iron Acquisition-siderophores	siderophores-Rhizobactin Metabolism	rhbD->PGPT0003404	1
Direct Effects	Biofertilization	Iron Acquisition	Iron Acquisition-siderophores	siderophores-Rhizobactin Metabolism	rhbF->PGPT0003407	1
Direct Effects	Biofertilization	Iron Acquisition	Iron Acquisition-siderophores	siderophore Export Systems	ymfE->PGPT0003580	3
Direct Effects	Biofertilization	Nitrogen Acquisition	N-Acquisition-Allantoin Usage	N-Acquisition-Allantoin Metabolism	allB->PGPT0000870	1
Direct Effects	Biofertilization	Nitrogen Acquisition	N-Acquisition-Allantoin Usage	N-Acquisition-Allantoin Metabolism	allC->PGPT0000875	1
Direct Effects	Biofertilization	Nitrogen Acquisition	N-Acquisition-Ammonium Assimilation Usage	N-Acquisition-Ammonium transport	amtB|ybaG|amt->PGPT0000840	3
Direct Effects	Biofertilization	Nitrogen Acquisition	N-Acquisition-Ammonium Assimilation Usage	N-Acquisition-Glutamate transport	TC AAT|yifK->PGPT0000815	9
Direct Effects	Biofertilization	Nitrogen Acquisition	N-Acquisition-Ammonium Assimilation Usage	N-Acquisition-Glutamate transport	gltP|gltT->PGPT0000805	5
Direct Effects	Biofertilization	Nitrogen Acquisition	N-Acquisition-Ammonium Assimilation Usage	N-Acquisition-Glutamate transport	gltS->PGPT0000810	1
Direct Effects	Biofertilization	Nitrogen Acquisition	N-Acquisition-Ammonium Assimilation Usage	N-Acquisition-Glutamate transport	ntrA|rpoN->PGPT0000795	2

Direct Effects	Biofertilization	Nitrogen Acquisition	N-Acquisition-Ammonium Assimilation Usage	N-Acquisition-Glutamate transport	peb1A|glnH->PGPT0000670	1
Direct Effects	Biofertilization	Nitrogen Acquisition	N-Acquisition-Ammonium Assimilation Usage	N-Acquisition-Glutamate transport	peb1B|glnP|glnM->PGPT0000705	2
Direct Effects	Biofertilization	Nitrogen Acquisition	N-Acquisition-Ammonium Assimilation Usage	N-Acquisition-Glutamate transport	peb1C|glnQ->PGPT0000710	1
Direct Effects	Biofertilization	Nitrogen Acquisition	N-Acquisition-Ammonium Assimilation Usage	N-Acquisition-Glutamate|Glutamine Metabolism	glnA->PGPT0000645	1
Direct Effects	Biofertilization	Nitrogen Acquisition	N-Acquisition-Ammonium Assimilation Usage	N-Acquisition-Glutamate|Glutamine Metabolism	glnB|glnY->PGPT0000650	2
Direct Effects	Biofertilization	Nitrogen Acquisition	N-Acquisition-Ammonium Assimilation Usage	N-Acquisition-Glutamate|Glutamine Metabolism	gltB->PGPT0000635	1
Direct Effects	Biofertilization	Nitrogen Acquisition	N-Acquisition-Ammonium Assimilation Usage	N-Acquisition-Glutamate|Glutamine Metabolism	gltD->PGPT0000640	1
Direct Effects	Biofertilization	Nitrogen Acquisition	N-Acquisition-Ammonium Assimilation Usage	N-Acquisition-Glutamine transport	glnT->PGPT0000745	2
Direct Effects	Biofertilization	Nitrogen Acquisition	N-Acquisition-Ammonium Assimilation Usage	N-Acquisition-Glutamine transport	peb1A|glnH->PGPT0000670	1
Direct Effects	Biofertilization	Nitrogen Acquisition	N-Acquisition-Ammonium Assimilation Usage	N-Acquisition-Glutamine transport	peb1B|glnP|glnM->PGPT0000705	2
Direct Effects	Biofertilization	Nitrogen Acquisition	N-Acquisition-Ammonium	N-Acquisition-Glutamine transport	peb1C|glnQ->PGPT0000710	1

			Assimilation Usage			
Direct Effects	Biofertilization	Nitrogen Acquisition	N-Acquisition-Ammonium Assimilation Usage	N-Acquisition-Glutamine transport	ycbA|glnK->PGPT0000735	2
Direct Effects	Biofertilization	Nitrogen Acquisition	N-Acquisition-Ammonium Assimilation Usage	N-Acquisition-Glutamine transport	ycbB|glnL->PGPT0000740	2
Direct Effects	Biofertilization	Nitrogen Acquisition	N-Acquisition-Atmospheric Nitrogen Fixation	N-Fix-Nitrogenase Biosynthesis	nifM|ppiC->PGPT0000050	2
Direct Effects	Biofertilization	Nitrogen Acquisition	N-Acquisition-Atmospheric Nitrogen Fixation	N-Fix-Nitrogenase Biosynthesis	nifS|iscS->PGPT0000065	4
Direct Effects	Biofertilization	Nitrogen Acquisition	N-Acquisition-Atmospheric Nitrogen Fixation	N-Fix-Nitrogenase Biosynthesis	nifU|iscU->PGPT0000075	2
Direct Effects	Biofertilization	Nitrogen Acquisition	N-Acquisition-Denitrification|Nitrate Usage	Denitrification-Nitrate Reduction	narK|nrtP|nrt|narU->PGPT0000300	1
Direct Effects	Biofertilization	Nitrogen Acquisition	N-Acquisition-Denitrification|Nitrate Usage	Denitrification-Nitrate Reduction	nasA|nasC|narB->PGPT0000390	1
Direct Effects	Biofertilization	Nitrogen Acquisition	N-Acquisition-Denitrification|Nitrate Usage	Denitrification-Nitrate Reduction	nfrA1|ywcG->PGPT0000290	1
Direct Effects	Biofertilization	Nitrogen Acquisition	N-Acquisition-Denitrification|Nitrate Usage	Denitrification-Nitrate Reduction	nfrA2|ycnD->PGPT0000295	1
Direct Effects	Biofertilization	Nitrogen Acquisition	N-Acquisition-Denitrification|Nitrate Usage	Denitrification-Nitrate Reduction	nirB->PGPT0000450	2
Direct	Biofertiliz	Nitrogen Acquisition	N-Acquisition-	Denitrification-Nitrate	nirD->PGPT0000455	1

Effects	ation		Denitrification|Nitrate Usage	Reduction		
Direct Effects	Biofertilization	Nitrogen Acquisition	N-Acquisition-Denitrification|Nitrate Usage	Denitrification-Nitrate Reduction	norQ->PGPT0000370	2
Direct Effects	Biofertilization	Nitrogen Acquisition	N-Acquisition-Denitrification|Nitrate Usage	Denitrification-Nitrate Reduction	nosR->PGPT0000425	1
Direct Effects	Biofertilization	Nitrogen Acquisition	N-Acquisition-Denitrification|Nitrate Usage	Denitrification-Nitrate Reduction	nosX|apbE|yojL|fmnB->PGPT0000430	2
Direct Effects	Biofertilization	Nitrogen Acquisition	N-Acquisition-Denitrification|Nitrate Usage	Denitrification-Nitrate|Nitrite Sensing	narL->PGPT0000550	1
Direct Effects	Biofertilization	Nitrogen Acquisition	N-Acquisition-Denitrification|Nitrate Usage	Denitrification-Nitrate|Nitrite Sensing	narQ->PGPT0000560	1
Direct Effects	Biofertilization	Nitrogen Acquisition	N-Acquisition-Denitrification|Nitrate Usage	Denitrification-Nitrate|Nitrite transport	nirC->PGPT0000565	1
Direct Effects	Biofertilization	Nitrogen Acquisition	N-Acquisition-EthanolAmine Usage	N-Acquisition-EthanolAmine Degradation	eutA->PGPT0000600	1
Direct Effects	Biofertilization	Nitrogen Acquisition	N-Acquisition-EthanolAmine Usage	N-Acquisition-EthanolAmine Degradation	eutB->PGPT0000605	1
Direct Effects	Biofertilization	Nitrogen Acquisition	N-Acquisition-EthanolAmine Usage	N-Acquisition-EthanolAmine Degradation	eutC->PGPT0000610	1
Direct Effects	Biofertilization	Nitrogen Acquisition	N-Acquisition-Regulation	Fumarate Based Nitrogenase Regulation	frdA->PGPT0001115	3

Direct Effects	Biofertilization	Nitrogen Acquisition	N-Acquisition-Regulation	GLN-Nitrogen Regulatory System	glnB|glnY->PGPT0000650	2
Direct Effects	Biofertilization	Nitrogen Acquisition	N-Acquisition-Regulation	GLN-Nitrogen Regulatory System	glnR->PGPT0001015	1
Direct Effects	Biofertilization	Nitrogen Acquisition	N-Acquisition-Regulation	N-Acquisition-Nitrate|Nitrite Sensing	narL->PGPT0000550	1
Direct Effects	Biofertilization	Nitrogen Acquisition	N-Acquisition-Regulation	N-Acquisition-Nitrate|Nitrite Sensing	narQ->PGPT0000560	1
Direct Effects	Biofertilization	Nitrogen Acquisition	N-Acquisition-Regulation	Nitrogen Regulating Functions	acoR->PGPT0001030	6
Direct Effects	Biofertilization	Nitrogen Acquisition	N-Acquisition-Regulation	Nitrogen Regulating Functions	draG->PGPT0001065	1
Direct Effects	Biofertilization	Nitrogen Acquisition	N-Acquisition-Regulation	Nitrogen Regulating Functions	FixA|etfB->PGPT0001035	1
Direct Effects	Biofertilization	Nitrogen Acquisition	N-Acquisition-Regulation	Nitrogen Regulating Functions	FixB|etfA->PGPT0001040	1
Direct Effects	Biofertilization	Nitrogen Acquisition	N-Acquisition-Regulation	Nitrogen Regulating Functions	tnrA->PGPT0001085	2
Direct Effects	Biofertilization	Nitrogen Acquisition	N-Acquisition-Regulation	NTR-Nitrogen Regulatory System	ntrA|rpoN->PGPT0000795	2
Direct Effects	Biofertilization	Nitrogen Acquisition	N-Acquisition-Related Functions	N-Acquisition-NitT|TauT Family transporter	ABC SN A|ytlC->PGPT0001140	3
Direct Effects	Biofertilization	Nitrogen Acquisition	N-Acquisition-Related Functions	N-Acquisition-NitT|TauT Family transporter	ABC SN P->PGPT0001145	3
Direct Effects	Biofertilization	Nitrogen Acquisition	N-Acquisition-trigonelline Usage	N-Acquisition-trigonelline Metabolism	tgnB->PGPT0000910	1
Direct	Biofertiliz	Nitrogen Acquisition	N-Acquisition-	N-Acquisition-	tgnR->PGPT0000900	3

Effects	ation		trigonelline Usage	trigonelline Metabolism		
Direct Effects	Biofertilization	Nitrogen Acquisition	N-Acquisition-Urea Usage	N-Acquisition-Urea Metabolism	atzF->PGPT0001010	1
Direct Effects	Biofertilization	Nitrogen Acquisition	N-Acquisition-Urea Usage	N-Acquisition-Urea Metabolism	uca|dur|urd->PGPT0001005	1
Direct Effects	Biofertilization	Nitrogen Acquisition	N-Acquisition-Urea Usage	N-Acquisition-Urea Metabolism	Urea->PGPT0000955	1
Direct Effects	Biofertilization	Nitrogen Acquisition	N-Acquisition-Urea Usage	N-Acquisition-Urea Metabolism	ureB->PGPT0000960	1
Direct Effects	Biofertilization	Nitrogen Acquisition	N-Acquisition-Urea Usage	N-Acquisition-Urea Metabolism	ureC->PGPT0000965	1
Direct Effects	Biofertilization	Nitrogen Acquisition	N-Acquisition-Urea Usage	N-Acquisition-Urea Metabolism	ureD->PGPT0000970	1
Direct Effects	Biofertilization	Nitrogen Acquisition	N-Acquisition-Urea Usage	N-Acquisition-Urea Metabolism	ureF->PGPT0000980	1
Direct Effects	Biofertilization	Nitrogen Acquisition	N-Acquisition-Urea Usage	N-Acquisition-Urea Metabolism	ureG->PGPT0000985	1
Direct Effects	Biofertilization	Nitrogen Acquisition	N-Acquisition-Urea Usage	N-Acquisition-Urea Metabolism	ureI->PGPT0000990	1
Direct Effects	Biofertilization	Phosphate Solubilization	P-Solubilisation-Organic Acid Metabolism	P-Solubilisation-Acetic Acid Biosynthesis	aceF|pdhC->PGPT0001390	2
Direct Effects	Biofertilization	Phosphate Solubilization	P-Solubilisation-Organic Acid Metabolism	P-Solubilisation-Acetic Acid Biosynthesis	ackA->PGPT0001360	1
Direct Effects	Biofertilization	Phosphate Solubilization	P-Solubilisation-Organic Acid Metabolism	P-Solubilisation-Acetic Acid Biosynthesis	acyP|yccX->PGPT0001372	1
Direct Effects	Biofertilization	Phosphate Solubilization	P-Solubilisation-Organic Acid Metabolism	P-Solubilisation-Acetic Acid Biosynthesis	korA|oorA|OforA->PGPT0001175	1

Direct Effects	Biofertilization	Phosphate Solubilization	P-Solubilisation-Organic Acid Metabolism	P-Solubilisation-Acetic Acid Biosynthesis	korB|oorB|OforB->PGPT0001180	1
Direct Effects	Biofertilization	Phosphate Solubilization	P-Solubilisation-Organic Acid Metabolism	P-Solubilisation-Acetic Acid Biosynthesis	lpd|pdhD->PGPT0001380	3
Direct Effects	Biofertilization	Phosphate Solubilization	P-Solubilisation-Organic Acid Metabolism	P-Solubilisation-Acetic Acid Biosynthesis	maeA|sfcA|ywkA->PGPT0001350	7
Direct Effects	Biofertilization	Phosphate Solubilization	P-Solubilisation-Organic Acid Metabolism	P-Solubilisation-Acetic Acid Biosynthesis	poxL->PGPT0007160	2
Direct Effects	Biofertilization	Phosphate Solubilization	P-Solubilisation-Organic Acid Metabolism	P-Solubilisation-Acetic Acid Biosynthesis	pta->PGPT0001365	1
Direct Effects	Biofertilization	Phosphate Solubilization	P-Solubilisation-Organic Acid Metabolism	P-Solubilisation-Acetic Acid transport	actP->PGPT0001400	1
Direct Effects	Biofertilization	Phosphate Solubilization	P-Solubilisation-Organic Acid Metabolism	P-Solubilisation-Aconitic Acid Biosynthesis	acnA->PGPT0001465	2
Direct Effects	Biofertilization	Phosphate Solubilization	P-Solubilisation-Organic Acid Metabolism	P-Solubilisation-Aconitic Acid Biosynthesis	prpF->PGPT0001520	1
Direct Effects	Biofertilization	Phosphate Solubilization	P-Solubilisation-Organic Acid Metabolism	P-Solubilisation-Butyric Acid Biosynthesis	buk->PGPT0001815	1
Direct Effects	Biofertilization	Phosphate Solubilization	P-Solubilisation-Organic Acid Metabolism	P-Solubilisation-Butyric Acid Biosynthesis	menI|ydiI|ydiL|ydiI|ybdB->PGPT0001845	1
Direct Effects	Biofertilization	Phosphate Solubilization	P-Solubilisation-Organic Acid	P-Solubilisation-Butyric Acid	ptb->PGPT0001810	1

			Metabolism	Biosynthesis		
Direct Effects	Biofertilization	Phosphate Solubilization	P-Solubilisation-Organic Acid Metabolism	P-Solubilisation-Butyric Acid Biosynthesis	ybgC->PGPT0001850	3
Direct Effects	Biofertilization	Phosphate Solubilization	P-Solubilisation-Organic Acid Metabolism	P-Solubilisation-Citric Acid Biosynthesis	CS|gltA->PGPT0001455	2
Direct Effects	Biofertilization	Phosphate Solubilization	P-Solubilisation-Organic Acid Metabolism	P-Solubilisation-Citric Acid Biosynthesis	acnA->PGPT0001465	2
Direct Effects	Biofertilization	Phosphate Solubilization	P-Solubilisation-Organic Acid Metabolism	P-Solubilisation-Citric Acid transport	TC CITMHS|CitMHS Family|citN->PGPT0001500	6
Direct Effects	Biofertilization	Phosphate Solubilization	P-Solubilisation-Organic Acid Metabolism	P-Solubilisation-Citric Acid transport	citS->PGPT0001490	2
Direct Effects	Biofertilization	Phosphate Solubilization	P-Solubilisation-Organic Acid Metabolism	P-Solubilisation-Formic Acid Biosynthesis	frc|yfdW->PGPT0002130	2
Direct Effects	Biofertilization	Phosphate Solubilization	P-Solubilisation-Organic Acid Metabolism	P-Solubilisation-Fumaric Acid Biosynthesis	nagK->PGPT0001620	1
Direct Effects	Biofertilization	Phosphate Solubilization	P-Solubilisation-Organic Acid Metabolism	P-Solubilisation-Fumaric Acid Biosynthesis	sdhA|frdA->PGPT0001605	1
Direct Effects	Biofertilization	Phosphate Solubilization	P-Solubilisation-Organic Acid Metabolism	P-Solubilisation-Fumaric Acid Biosynthesis	sdhB|frdB->PGPT0001600	1
Direct Effects	Biofertilization	Phosphate Solubilization	P-Solubilisation-Organic Acid Metabolism	P-Solubilisation-Fumaric Acid Biosynthesis	sdhC|frdC->PGPT0001595	1
Direct	Biofertiliz	Phosphate	P-Solubilisation-	P-Solubilisation-	dctA->PGPT0001450	2

Effects	ation	Solubilization	Organic Acid Metabolism	Fumaric Acid transport		
Direct Effects	Biofertilization	Phosphate Solubilization	P-Solubilisation-Organic Acid Metabolism	P-Solubilisation-Galactonic Acid transport	dgoT->PGPT0002190	3
Direct Effects	Biofertilization	Phosphate Solubilization	P-Solubilisation-Organic Acid Metabolism	P-Solubilisation-Gluconic Acid-PQQ Pathway	argD|pqqI->PGPT0014253	3
Direct Effects	Biofertilization	Phosphate Solubilization	P-Solubilisation-Organic Acid Metabolism	P-Solubilisation-Gluconic Acid-PQQ Pathway	pqqL|yddC->PGPT0001280	1
Direct Effects	Biofertilization	Phosphate Solubilization	P-Solubilisation-Organic Acid Metabolism	P-Solubilisation-Gluconic Acid transport	TC GNTP->PGPT0001340	4
Direct Effects	Biofertilization	Phosphate Solubilization	P-Solubilisation-Organic Acid Metabolism	P-Solubilisation-Glycolic Acid Biosynthesis	gph->PGPT0001730	3
Direct Effects	Biofertilization	Phosphate Solubilization	P-Solubilisation-Organic Acid Metabolism	P-Solubilisation-Glycolic Acid Biosynthesis	rbcL|cbbL->PGPT0001150	1
Direct Effects	Biofertilization	Phosphate Solubilization	P-Solubilisation-Organic Acid Metabolism	P-Solubilisation-Glyoxylic Acid Biosynthesis	aceA->PGPT0001550	1
Direct Effects	Biofertilization	Phosphate Solubilization	P-Solubilisation-Organic Acid Metabolism	P-Solubilisation-Keto-Gluconate Biosynthesis	dkgA->PGPT0001320	3
Direct Effects	Biofertilization	Phosphate Solubilization	P-Solubilisation-Organic Acid Metabolism	P-Solubilisation-Keto-|OxoGlutarate transport	TC DASS|yflS->PGPT0013960	1
Direct Effects	Biofertilization	Phosphate Solubilization	P-Solubilisation-Organic Acid Metabolism	P-Solubilisation-Keto-|OxoGlutarate transport	kgtP->PGPT0001525	1

Direct Effects	Biofertiliz ation	Phosphate Solubilization	P-Solubilisation-Organic Acid Metabolism	P-Solubilisation-Ketoglutaric Acid Biosynthesis	icd->PGPT0001170	1
Direct Effects	Biofertiliz ation	Phosphate Solubilization	P-Solubilisation-Organic Acid Metabolism	P-Solubilisation-Lactic Acid Biosynthesis	gloB|gloC->PGPT0001770	10
Direct Effects	Biofertiliz ation	Phosphate Solubilization	P-Solubilisation-Organic Acid Metabolism	P-Solubilisation-Lactic Acid Biosynthesis	ldh->PGPT0001760	1
Direct Effects	Biofertiliz ation	Phosphate Solubilization	P-Solubilisation-Organic Acid Metabolism	P-Solubilisation-Lactic Acid Biosynthesis	lra6->PGPT0001745	2
Direct Effects	Biofertiliz ation	Phosphate Solubilization	P-Solubilisation-Organic Acid Metabolism	P-Solubilisation-Lactic Acid Biosynthesis	mgsA->PGPT0001780	1
Direct Effects	Biofertiliz ation	Phosphate Solubilization	P-Solubilisation-Organic Acid Metabolism	P-Solubilisation-Lactic Acid transport	lctP->PGPT0001800	2
Direct Effects	Biofertiliz ation	Phosphate Solubilization	P-Solubilisation-Organic Acid Metabolism	P-Solubilisation-Lactic Acid transport	lldP|lctP->PGPT0001805	1
Direct Effects	Biofertiliz ation	Phosphate Solubilization	P-Solubilisation-Organic Acid Metabolism	P-Solubilisation-Malic Acid Biosynthesis	fumA|fumB->PGPT0001635	1
Direct Effects	Biofertiliz ation	Phosphate Solubilization	P-Solubilisation-Organic Acid Metabolism	P-Solubilisation-Malic Acid Biosynthesis	fumC->PGPT0001650	1
Direct Effects	Biofertiliz ation	Phosphate Solubilization	P-Solubilisation-Organic Acid Metabolism	P-Solubilisation-Malic Acid Biosynthesis	maeA|sfcA|ywkA->PGPT0001350	7
Direct Effects	Biofertiliz ation	Phosphate Solubilization	P-Solubilisation-Organic Acid	P-Solubilisation-Malic Acid Biosynthesis	mdh->PGPT0001435	1

			Metabolism			
Direct Effects	Biofertilization	Phosphate Solubilization	P-Solubilisation-Organic Acid Metabolism	P-Solubilisation-Malic Acid Biosynthesis	mqo->PGPT0001440	2
Direct Effects	Biofertilization	Phosphate Solubilization	P-Solubilisation-Organic Acid Metabolism	P-Solubilisation-Malic Acid transport	dctA->PGPT0001450	2
Direct Effects	Biofertilization	Phosphate Solubilization	P-Solubilisation-Organic Acid Metabolism	P-Solubilisation-Malic Acid transport	TC DASS|yflS->PGPT0013960	1
Direct Effects	Biofertilization	Phosphate Solubilization	P-Solubilisation-Organic Acid Metabolism	P-Solubilisation-Malic Acid transport	maeN->PGPT0001690	2
Direct Effects	Biofertilization	Phosphate Solubilization	P-Solubilisation-Organic Acid Metabolism	P-Solubilisation-Malonic Acid Biosynthesis	accA->PGPT0001695	1
Direct Effects	Biofertilization	Phosphate Solubilization	P-Solubilisation-Organic Acid Metabolism	P-Solubilisation-Malonic Acid Biosynthesis	accB|bccP->PGPT0001700	3
Direct Effects	Biofertilization	Phosphate Solubilization	P-Solubilisation-Organic Acid Metabolism	P-Solubilisation-Malonic Acid Biosynthesis	accC->PGPT0001705	3
Direct Effects	Biofertilization	Phosphate Solubilization	P-Solubilisation-Organic Acid Metabolism	P-Solubilisation-Malonic Acid Biosynthesis	accD->PGPT0001710	1
Direct Effects	Biofertilization	Phosphate Solubilization	P-Solubilisation-Organic Acid Metabolism	P-Solubilisation-Malonic Acid Biosynthesis	pccB->PGPT0001712	1
Direct Effects	Biofertilization	Phosphate Solubilization	P-Solubilisation-Organic Acid Metabolism	P-Solubilisation-Malonic Acid transport	mdcF->PGPT0001720	2
Direct	Biofertiliz	Phosphate	P-Solubilisation-	P-Solubilisation-	aceB|glcB-	1

Effects	ation	Solubilization	Organic Acid Metabolism	OxalAcetic Acid Biosynthesis	>PGPT0001445	
Direct Effects	Biofertiliz ation	Phosphate Solubilization	P-Solubilisation-Organic Acid Metabolism	P-Solubilisation-OxalAcetic Acid Biosynthesis	mdh->PGPT0001435	1
Direct Effects	Biofertiliz ation	Phosphate Solubilization	P-Solubilisation-Organic Acid Metabolism	P-Solubilisation-OxalAcetic Acid Biosynthesis	mqo->PGPT0001440	2
Direct Effects	Biofertiliz ation	Phosphate Solubilization	P-Solubilisation-Organic Acid Metabolism	P-Solubilisation-OxalAcetic Acid Biosynthesis	pckA->PGPT0001405	1
Direct Effects	Biofertiliz ation	Phosphate Solubilization	P-Solubilisation-Organic Acid Metabolism	P-Solubilisation-OxalAcetic Acid Biosynthesis	pyc->PGPT0001420	1
Direct Effects	Biofertiliz ation	Phosphate Solubilization	P-Solubilisation-Organic Acid Metabolism	P-Solubilisation-OxalAcetic Acid transport	dctA->PGPT0001450	2
Direct Effects	Biofertiliz ation	Phosphate Solubilization	P-Solubilisation-Organic Acid Metabolism	P-Solubilisation-Propionic Acid Biosynthesis	ackA->PGPT0001360	1
Direct Effects	Biofertiliz ation	Phosphate Solubilization	P-Solubilisation-Organic Acid Metabolism	P-Solubilisation-Propionic Acid Biosynthesis	acs->PGPT0002265	2
Direct Effects	Biofertiliz ation	Phosphate Solubilization	P-Solubilisation-Organic Acid Metabolism	P-Solubilisation-Propionic Acid Biosynthesis	acuI|yhdH->PGPT0002275	1
Direct Effects	Biofertiliz ation	Phosphate Solubilization	P-Solubilisation-Organic Acid Metabolism	P-Solubilisation-Propionic Acid Biosynthesis	bcd->PGPT0002285	8
Direct Effects	Biofertiliz ation	Phosphate Solubilization	P-Solubilisation-Organic Acid Metabolism	P-Solubilisation-Propionic Acid Biosynthesis	mmdA->PGPT0002300	1

Direct Effects	Biofertilization	Phosphate Solubilization	P-Solubilisation-Organic Acid Metabolism	P-Solubilisation-Propionic Acid Biosynthesis	mmsA|iolA->PGPT0002295	4
Direct Effects	Biofertilization	Phosphate Solubilization	P-Solubilisation-Organic Acid Metabolism	P-Solubilisation-Propionic Acid Biosynthesis	pta->PGPT0001365	1
Direct Effects	Biofertilization	Phosphate Solubilization	P-Solubilisation-Organic Acid Metabolism	P-Solubilisation-Pyruvic Acid Biosynthesis	cuyA->PGPT0002000	1
Direct Effects	Biofertilization	Phosphate Solubilization	P-Solubilisation-Organic Acid Metabolism	P-Solubilisation-Pyruvic Acid Biosynthesis	dapA|mosA->PGPT0002080	3
Direct Effects	Biofertilization	Phosphate Solubilization	P-Solubilisation-Organic Acid Metabolism	P-Solubilisation-Pyruvic Acid Biosynthesis	dat->PGPT0001915	1
Direct Effects	Biofertilization	Phosphate Solubilization	P-Solubilisation-Organic Acid Metabolism	P-Solubilisation-Pyruvic Acid Biosynthesis	dld->PGPT0001890	2
Direct Effects	Biofertilization	Phosphate Solubilization	P-Solubilisation-Organic Acid Metabolism	P-Solubilisation-Pyruvic Acid Biosynthesis	dsdA->PGPT0001970	1
Direct Effects	Biofertilization	Phosphate Solubilization	P-Solubilisation-Organic Acid Metabolism	P-Solubilisation-Pyruvic Acid Biosynthesis	eda->PGPT0002060	1
Direct Effects	Biofertilization	Phosphate Solubilization	P-Solubilisation-Organic Acid Metabolism	P-Solubilisation-Pyruvic Acid Biosynthesis	ilvA|tdcB->PGPT0001960	1
Direct Effects	Biofertilization	Phosphate Solubilization	P-Solubilisation-Organic Acid Metabolism	P-Solubilisation-Pyruvic Acid Biosynthesis	ldh->PGPT0001760	1
Direct Effects	Biofertilization	Phosphate Solubilization	P-Solubilisation-Organic Acid	P-Solubilisation-Pyruvic Acid	ligK|galC->PGPT0002085	5

			Metabolism	Biosynthesis		
Direct Effects	Biofertilization	Phosphate Solubilization	P-Solubilisation-Organic Acid Metabolism	P-Solubilisation-Pyruvic Acid Biosynthesis	maeA|sfcA|ywkA->PGPT0001350	7
Direct Effects	Biofertilization	Phosphate Solubilization	P-Solubilisation-Organic Acid Metabolism	P-Solubilisation-Pyruvic Acid Biosynthesis	mccB->PGPT0001985	2
Direct Effects	Biofertilization	Phosphate Solubilization	P-Solubilisation-Organic Acid Metabolism	P-Solubilisation-Pyruvic Acid Biosynthesis	metC->PGPT0001995	2
Direct Effects	Biofertilization	Phosphate Solubilization	P-Solubilisation-Organic Acid Metabolism	P-Solubilisation-Pyruvic Acid Biosynthesis	mhpE->PGPT0002050	1
Direct Effects	Biofertilization	Phosphate Solubilization	P-Solubilisation-Organic Acid Metabolism	P-Solubilisation-Pyruvic Acid Biosynthesis	nagK->PGPT0001620	1
Direct Effects	Biofertilization	Phosphate Solubilization	P-Solubilisation-Organic Acid Metabolism	P-Solubilisation-Pyruvic Acid Biosynthesis	patB|malY->PGPT0001990	1
Direct Effects	Biofertilization	Phosphate Solubilization	P-Solubilisation-Organic Acid Metabolism	P-Solubilisation-Pyruvic Acid Biosynthesis	ppdK->PGPT0002040	1
Direct Effects	Biofertilization	Phosphate Solubilization	P-Solubilisation-Organic Acid Metabolism	P-Solubilisation-Pyruvic Acid Biosynthesis	pps|ppsA->PGPT0002035	2
Direct Effects	Biofertilization	Phosphate Solubilization	P-Solubilisation-Organic Acid Metabolism	P-Solubilisation-Pyruvic Acid Biosynthesis	ptsI->PGPT0002025	1
Direct Effects	Biofertilization	Phosphate Solubilization	P-Solubilisation-Organic Acid Metabolism	P-Solubilisation-Pyruvic Acid Biosynthesis	pyk->PGPT0002020	2
Direct	Biofertiliz	Phosphate	P-Solubilisation-	P-Solubilisation-	sdaA|sdaB|tdcG-	2

Effects	ation	Solubilization	Organic Acid Metabolism	Pyruvic Acid Biosynthesis	>PGPT0001975	
Direct Effects	Biofertiliz ation	Phosphate Solubilization	P-Solubilisation-Organic Acid Metabolism	P-Solubilisation-Pyruvic Acid Biosynthesis	sseA->PGPT0002045	2
Direct Effects	Biofertiliz ation	Phosphate Solubilization	P-Solubilisation-Organic Acid Metabolism	P-Solubilisation-Pyruvic Acid Biosynthesis	tpav|bioA|yhxA->PGPT0001925	2
Direct Effects	Biofertiliz ation	Phosphate Solubilization	P-Solubilisation-Organic Acid Metabolism	P-Solubilisation-Pyruvic Acid Biosynthesis	ttuC|dmlA->PGPT0001905	1
Direct Effects	Biofertiliz ation	Phosphate Solubilization	P-Solubilisation-Organic Acid Metabolism	P-Solubilisation-Succinic Acid Acid Biosynthesis	aceA->PGPT0001550	1
Direct Effects	Biofertiliz ation	Phosphate Solubilization	P-Solubilisation-Organic Acid Metabolism	P-Solubilisation-Succinic Acid Acid Biosynthesis	frdA->PGPT0001115	3
Direct Effects	Biofertiliz ation	Phosphate Solubilization	P-Solubilisation-Organic Acid Metabolism	P-Solubilisation-Succinic Acid Acid Biosynthesis	gabD->PGPT0001580	5
Direct Effects	Biofertiliz ation	Phosphate Solubilization	P-Solubilisation-Organic Acid Metabolism	P-Solubilisation-Succinic Acid Acid Biosynthesis	icd->PGPT0001170	1
Direct Effects	Biofertiliz ation	Phosphate Solubilization	P-Solubilisation-Organic Acid Metabolism	P-Solubilisation-Succinic Acid Acid Biosynthesis	korA|oorA|OforA->PGPT0001175	1
Direct Effects	Biofertiliz ation	Phosphate Solubilization	P-Solubilisation-Organic Acid Metabolism	P-Solubilisation-Succinic Acid Acid Biosynthesis	korB|oorB|OforB->PGPT0001180	1
Direct Effects	Biofertiliz ation	Phosphate Solubilization	P-Solubilisation-Organic Acid Metabolism	P-Solubilisation-Succinic Acid Acid Biosynthesis	prpB->PGPT0001555	1

Direct Effects	Biofertilization	Phosphate Solubilization	P-Solubilisation-Organic Acid Metabolism	P-Solubilisation-Succinic Acid Acid Biosynthesis	sucA->PGPT0001530	1
Direct Effects	Biofertilization	Phosphate Solubilization	P-Solubilisation-Organic Acid Metabolism	P-Solubilisation-Succinic Acid Acid Biosynthesis	sucB->PGPT0001535	2
Direct Effects	Biofertilization	Phosphate Solubilization	P-Solubilisation-Organic Acid Metabolism	P-Solubilisation-Succinic Acid Acid Biosynthesis	sucD->PGPT0001540	1
Direct Effects	Biofertilization	Phosphate Solubilization	P-Solubilisation-Organic Acid Metabolism	P-Solubilisation-Succinic Acid Acid transport	dctA->PGPT0001450	2
Direct Effects	Biofertilization	Phosphate Solubilization	P-Solubilisation-Organic Acid Metabolism	P-Solubilisation-Tartaric Acid Biosynthesis	ttuC|dmlA->PGPT0001905	1
Direct Effects	Biofertilization	Phosphate Solubilization	P-Solubilisation-Organic Acid Metabolism	P-Solubilisation-Tartaric Acid transport	dctA->PGPT0001450	2
Direct Effects	Biofertilization	Phosphate Solubilization	P-Solubilisation-Organic Acid Metabolism	P-Solubilisation-Tartaric Acid transport	ttuB->PGPT0002325	3
Direct Effects	Biofertilization	Phosphate Solubilization	P-Solubilisation-Organic Acid Metabolism	P-Solubilisation-Valeric Acid Biosynthesis	actA->PGPT0001885	1
Direct Effects	Biofertilization	Phosphate Solubilization	P-Solubilisation-Organic Acid Metabolism	P-Solubilisation-Valeric Acid Biosynthesis	ilvD->PGPT0001875	1
Direct Effects	Biofertilization	Phosphate Solubilization	P-Solubilisation-Organic Acid Metabolism	P-Solubilisation-Valeric Acid Biosynthesis	paaF|echA->PGPT0001860	1
Direct Effects	Biofertilization	Phosphate Solubilization	P-Solubilisation-Organic Acid	P-Solubilisation D Gluconate	gnl->PGPT0001285	1

			Metabolism	Biosynthesis		
Direct Effects	Biofertilization	Phosphate Solubilization	P-Solubilisation-Other Acid Metabolism	P-Solubilisation-Carbonic Acid Biosynthesis	GammaCA Like->PGPT0002425	1
Direct Effects	Biofertilization	Phosphate Solubilization	P-Solubilisation-Other Acid Metabolism	P-Solubilisation-Carbonic Acid Biosynthesis	cynT|can->PGPT0002415	2
Direct Effects	Biofertilization	Phosphate Solubilization	P-Solubilisation-Other Acid Metabolism	P-Solubilisation-Nitric Acid Biosynthesis	nasA|nasC|narB->PGPT0000390	1
Direct Effects	Biofertilization	Phosphate Solubilization	P-Solubilisation-Other Acid Metabolism	P-Solubilisation-Phosphonate Degradation	phnF->PGPT0002515	1
Direct Effects	Biofertilization	Phosphate Solubilization	P-Solubilisation-Other Acid Metabolism	P-Solubilisation-Phosphonate Degradation	phnO->PGPT0002475	1
Direct Effects	Biofertilization	Phosphate Solubilization	P-Solubilisation-Other Acid Metabolism	P-Solubilisation-Phosphonate Degradation	phnP->PGPT0002480	2
Direct Effects	Biofertilization	Phosphate Solubilization	P-Solubilisation-Other Acid Metabolism	P-Solubilisation-Phosphonate Degradation	phnW->PGPT0002485	1
Direct Effects	Biofertilization	Phosphate Solubilization	P-Solubilisation-Other Acid Metabolism	P-Solubilisation-Sulfuric Acid Biosynthesis	SUOX Like->PGPT0002375	1
Direct Effects	Biofertilization	Phosphate Solubilization	P-Solubilisation-Other Acid Metabolism	P-Solubilisation-Sulfuric Acid Biosynthesis	sat|met3->PGPT0002410	1
Direct Effects	Biofertilization	Phosphate Solubilization	P-Solubilisation-Phosphatase Activity	P-Solubilisation-Alkaline Phosphatase	phoA->PGPT0002570	2
Direct Effects	Biofertilization	Phosphate Solubilization	P-Solubilisation-Phosphatase Activity	P-Solubilisation-Alkaline Phosphatase	phoD->PGPT0002575	1
Direct	Biofertiliz	Phosphate	P-Solubilisation-	P-Solubilisation-	ppx|ppx gppA-	1

Effects	ation	Solubilization	Phosphatase Activity	Exoployphosphatase	>PGPT0002595	
Direct Effects	Biofertilization	Phosphate Solubilization	P-Solubilisation-Phosphatase Activity	P-Solubilisation-Phytase Production	phy|phyA|phyB|phyC->PGPT0002580	1
Direct Effects	Biofertilization	Phosphate Solubilization	P-Solubilisation-Phosphatase Activity	P-Solubilisation-Uncharacterized Phosphatase	phoE->PGPT0002590	1
Direct Effects	Biofertilization	Phosphate Solubilization	P-Solubilisation-Phosphate Metabolism	P-Solubilisation-Phosphate Homeostasis|Regulation	phoB->PGPT0002660	1
Direct Effects	Biofertilization	Phosphate Solubilization	P-Solubilisation-Phosphate Metabolism	P-Solubilisation-Phosphate Homeostasis|Regulation	phoH->PGPT0002700	1
Direct Effects	Biofertilization	Phosphate Solubilization	P-Solubilisation-Phosphate Metabolism	P-Solubilisation-Phosphate Homeostasis|Regulation	phoP|phoB1->PGPT0002665	4
Direct Effects	Biofertilization	Phosphate Solubilization	P-Solubilisation-Phosphate Metabolism	P-Solubilisation-Phosphate Homeostasis|Regulation	phoR->PGPT0002705	3
Direct Effects	Biofertilization	Phosphate Solubilization	P-Solubilisation-Phosphate Metabolism	P-Solubilisation-Phosphate Homeostasis|Regulation	ppk->PGPT0002685	1
Direct Effects	Biofertilization	Phosphate Solubilization	P-Solubilisation-Phosphate Metabolism	P-Solubilisation-Phosphate Homeostasis|Regulation	regX3->PGPT0002675	2
Direct Effects	Biofertilization	Phosphate Solubilization	P-Solubilisation-Phosphate Metabolism	P-Solubilisation-Phosphate	yciG|ymdF|gsiB->PGPT0002680	3

				Homeostasis|Regulation		
Direct Effects	Biofertilization	Phosphate Solubilization	P-Solubilisation-Phosphate Metabolism	P-Solubilisation-Phosphate Homeostasis|Regulation	yxiE->PGPT0002681	4
Direct Effects	Biofertilization	Phosphate Solubilization	P-Solubilisation-Phosphate Metabolism	P-Solubilisation-Phosphate transport	TC PIT->PGPT0002655	3
Direct Effects	Biofertilization	Phosphate Solubilization	P-Solubilisation-Phosphate Metabolism	P-Solubilisation-Phosphate transport	phoU|phoY->PGPT0002630	2
Direct Effects	Biofertilization	Phosphate Solubilization	P-Solubilisation-Phosphate Metabolism	P-Solubilisation-Phosphate transport	pstA->PGPT0002610	1
Direct Effects	Biofertilization	Phosphate Solubilization	P-Solubilisation-Phosphate Metabolism	P-Solubilisation-Phosphate transport	pstB|phoT->PGPT0002615	1
Direct Effects	Biofertilization	Phosphate Solubilization	P-Solubilisation-Phosphate Metabolism	P-Solubilisation-Phosphate transport	pstC|phoW->PGPT0002620	1
Direct Effects	Biofertilization	Phosphate Solubilization	P-Solubilisation-Phosphate Metabolism	P-Solubilisation-Phosphate transport	pstS|phoS->PGPT0002625	1
Direct Effects	Biofertilization	Potassium Solubilization	K-Solubilisation-Organic Acid Metabolism	K-Solubilisation-Malic Acid transport	TC DASS|yflS->PGPT0013960	1
Direct Effects	Biofertilization	Potassium Solubilization	K-Solubilisation-Other Acid MetabolismS	K-Solubilisation-Carbonic Acid Biosynthesis	GammaCA Like->PGPT0002425	1
Direct Effects	Biofertilization	Potassium Solubilization	K-Solubilisation-Other Acid MetabolismS	K-Solubilisation-Carbonic Acid Biosynthesis	cynT|can->PGPT0002415	2
Direct Effects	Biofertilization	Potassium Solubilization	K-Solubilisation-Other Acid MetabolismS	K-Solubilisation-Nitric Acid Biosynthesis	nasA|nasC|narB->PGPT0000390	1
Direct Effects	Biofertilization	Potassium Solubilization	K-Solubilisation-Other Acid MetabolismS	K-Solubilisation-Sulfuric Acid Biosynthesis	SUOX Like->PGPT0002375	1

Direct Effects	Biofertilization	Potassium Solubilization	K-Solubilisation-Other Acid MetabolismS	K-Solubilisation-Sulfuric Acid Biosynthesis	sat|met3->PGPT0002410	1
Direct Effects	Biofertilization	Potassium Solubilization	K-Solubilization-Organic Acid Metabolism	K-Solubilisation-Acetic Acid Biosynthesis	aceF|pdhC->PGPT0001390	2
Direct Effects	Biofertilization	Potassium Solubilization	K-Solubilization-Organic Acid Metabolism	K-Solubilisation-Acetic Acid Biosynthesis	ackA->PGPT0001360	1
Direct Effects	Biofertilization	Potassium Solubilization	K-Solubilization-Organic Acid Metabolism	K-Solubilisation-Acetic Acid Biosynthesis	acyP|yccX->PGPT0001372	1
Direct Effects	Biofertilization	Potassium Solubilization	K-Solubilization-Organic Acid Metabolism	K-Solubilisation-Acetic Acid Biosynthesis	korA|oorA|OforA->PGPT0001175	1
Direct Effects	Biofertilization	Potassium Solubilization	K-Solubilization-Organic Acid Metabolism	K-Solubilisation-Acetic Acid Biosynthesis	korB|oorB|OforB->PGPT0001180	1
Direct Effects	Biofertilization	Potassium Solubilization	K-Solubilization-Organic Acid Metabolism	K-Solubilisation-Acetic Acid Biosynthesis	lpd|pdhD->PGPT0001380	3
Direct Effects	Biofertilization	Potassium Solubilization	K-Solubilization-Organic Acid Metabolism	K-Solubilisation-Acetic Acid Biosynthesis	maeA|sfcA|ywkA->PGPT0001350	7
Direct Effects	Biofertilization	Potassium Solubilization	K-Solubilization-Organic Acid Metabolism	K-Solubilisation-Acetic Acid Biosynthesis	poxL->PGPT0007160	2
Direct Effects	Biofertilization	Potassium Solubilization	K-Solubilization-Organic Acid Metabolism	K-Solubilisation-Acetic Acid Biosynthesis	pta->PGPT0001365	1
Direct Effects	Biofertilization	Potassium Solubilization	K-Solubilization-Organic Acid	K-Solubilisation-Acetic Acid transport	actP->PGPT0001400	1

			Metabolism			
Direct Effects	Biofertiliz ation	Potassium Solubilization	K-Solubilization-Organic Acid Metabolism	K-Solubilisation-Aconitic Acid Biosynthesis	acnA->PGPT0001465	2
Direct Effects	Biofertiliz ation	Potassium Solubilization	K-Solubilization-Organic Acid Metabolism	K-Solubilisation-Aconitic Acid Biosynthesis	prpF->PGPT0001520	1
Direct Effects	Biofertiliz ation	Potassium Solubilization	K-Solubilization-Organic Acid Metabolism	K-Solubilisation-Butyric Acid Biosynthesis	buk->PGPT0001815	1
Direct Effects	Biofertiliz ation	Potassium Solubilization	K-Solubilization-Organic Acid Metabolism	K-Solubilisation-Butyric Acid Biosynthesis	menI|ydiI|ydiL|ydiI| ybdB->PGPT0001845	1
Direct Effects	Biofertiliz ation	Potassium Solubilization	K-Solubilization-Organic Acid Metabolism	K-Solubilisation-Butyric Acid Biosynthesis	ptb->PGPT0001810	1
Direct Effects	Biofertiliz ation	Potassium Solubilization	K-Solubilization-Organic Acid Metabolism	K-Solubilisation-Butyric Acid Biosynthesis	ybgC->PGPT0001850	3
Direct Effects	Biofertiliz ation	Potassium Solubilization	K-Solubilization-Organic Acid Metabolism	K-Solubilisation-Citric Acid Biosynthesis	CS|gltA->PGPT0001455	2
Direct Effects	Biofertiliz ation	Potassium Solubilization	K-Solubilization-Organic Acid Metabolism	K-Solubilisation-Citric Acid Biosynthesis	acnA->PGPT0001465	2
Direct Effects	Biofertiliz ation	Potassium Solubilization	K-Solubilization-Organic Acid Metabolism	K-Solubilisation-Citric Acid transport	TC CITMHS|CitMHS Family|citN->PGPT0001500	6
Direct Effects	Biofertiliz ation	Potassium Solubilization	K-Solubilization-Organic Acid Metabolism	K-Solubilisation-Citric Acid transport	citS->PGPT0001490	2
Direct	Biofertiliz	Potassium	K-Solubilization-	K-Solubilisation-	nagK->PGPT0001620	1

Effects	ation	Solubilization	Organic Acid Metabolism	Fumaric Acid Biosynthesis		
Direct Effects	Biofertilization	Potassium Solubilization	K-Solubilization-Organic Acid Metabolism	K-Solubilisation-Fumaric Acid Biosynthesis	sdhA|frdA->PGPT0001605	1
Direct Effects	Biofertilization	Potassium Solubilization	K-Solubilization-Organic Acid Metabolism	K-Solubilisation-Fumaric Acid Biosynthesis	sdhB|frdB->PGPT0001600	1
Direct Effects	Biofertilization	Potassium Solubilization	K-Solubilization-Organic Acid Metabolism	K-Solubilisation-Fumaric Acid Biosynthesis	sdhC|frdC->PGPT0001595	1
Direct Effects	Biofertilization	Potassium Solubilization	K-Solubilization-Organic Acid Metabolism	K-Solubilisation-Fumaric Acid transport	dctA->PGPT0001450	2
Direct Effects	Biofertilization	Potassium Solubilization	K-Solubilization-Organic Acid Metabolism	K-Solubilisation-Galactonic Acid transport	dgoT->PGPT0002190	3
Direct Effects	Biofertilization	Potassium Solubilization	K-Solubilization-Organic Acid Metabolism	K-Solubilisation-Gluconic Acid-PQQ Pathway	argD|pqqI->PGPT0014253	3
Direct Effects	Biofertilization	Potassium Solubilization	K-Solubilization-Organic Acid Metabolism	K-Solubilisation-Gluconic Acid-PQQ Pathway	pqqL|yddC->PGPT0001280	1
Direct Effects	Biofertilization	Potassium Solubilization	K-Solubilization-Organic Acid Metabolism	K-Solubilisation-Gluconic Acid transport	TC GNTP->PGPT0001340	4
Direct Effects	Biofertilization	Potassium Solubilization	K-Solubilization-Organic Acid Metabolism	K-Solubilisation-Glyoxylic Acid Biosynthesis	aceA->PGPT0001550	1
Direct Effects	Biofertilization	Potassium Solubilization	K-Solubilization-Organic Acid Metabolism	K-Solubilisation-Keto-Gluconate Biosynthesis	dkgA->PGPT0001320	3

Direct Effects	Biofertilization	Potassium Solubilization	K-Solubilization-Organic Acid Metabolism	K-Solubilisation-Keto-|OxoGlutarate transport	TC DASS|yflS->PGPT0013960	1
Direct Effects	Biofertilization	Potassium Solubilization	K-Solubilization-Organic Acid Metabolism	K-Solubilisation-Keto-|OxoGlutarate transport	kgtP->PGPT0001525	1
Direct Effects	Biofertilization	Potassium Solubilization	K-Solubilization-Organic Acid Metabolism	K-Solubilisation-Ketoglutaric Acid Biosynthesis	icd->PGPT0001170	1
Direct Effects	Biofertilization	Potassium Solubilization	K-Solubilization-Organic Acid Metabolism	K-Solubilisation-Lactic Acid Biosynthesis	gloB|gloC->PGPT0001770	10
Direct Effects	Biofertilization	Potassium Solubilization	K-Solubilization-Organic Acid Metabolism	K-Solubilisation-Lactic Acid Biosynthesis	ldh->PGPT0001760	1
Direct Effects	Biofertilization	Potassium Solubilization	K-Solubilization-Organic Acid Metabolism	K-Solubilisation-Lactic Acid Biosynthesis	lra6->PGPT0001745	2
Direct Effects	Biofertilization	Potassium Solubilization	K-Solubilization-Organic Acid Metabolism	K-Solubilisation-Lactic Acid Biosynthesis	mgsA->PGPT0001780	1
Direct Effects	Biofertilization	Potassium Solubilization	K-Solubilization-Organic Acid Metabolism	K-Solubilisation-Lactic Acid transport	lctP->PGPT0001800	2
Direct Effects	Biofertilization	Potassium Solubilization	K-Solubilization-Organic Acid Metabolism	K-Solubilisation-Lactic Acid transport	lldP|lctP->PGPT0001805	1
Direct Effects	Biofertilization	Potassium Solubilization	K-Solubilization-Organic Acid Metabolism	K-Solubilisation-Malic Acid Biosynthesis	fumA|fumB->PGPT0001635	1
Direct Effects	Biofertilization	Potassium Solubilization	K-Solubilization-Organic Acid	K-Solubilisation-Malic Acid Biosynthesis	fumC->PGPT0001650	1

			Metabolism			
Direct Effects	Biofertilization	Potassium Solubilization	K-Solubilization-Organic Acid Metabolism	K-Solubilisation-Malic Acid Biosynthesis	maeA|sfcA|ywkA->PGPT0001350	7
Direct Effects	Biofertilization	Potassium Solubilization	K-Solubilization-Organic Acid Metabolism	K-Solubilisation-Malic Acid Biosynthesis	mdh->PGPT0001435	1
Direct Effects	Biofertilization	Potassium Solubilization	K-Solubilization-Organic Acid Metabolism	K-Solubilisation-Malic Acid Biosynthesis	mqo->PGPT0001440	2
Direct Effects	Biofertilization	Potassium Solubilization	K-Solubilization-Organic Acid Metabolism	K-Solubilisation-Malic Acid transport	dctA->PGPT0001450	2
Direct Effects	Biofertilization	Potassium Solubilization	K-Solubilization-Organic Acid Metabolism	K-Solubilisation-Malic Acid transport	maeN->PGPT0001690	2
Direct Effects	Biofertilization	Potassium Solubilization	K-Solubilization-Organic Acid Metabolism	K-Solubilisation-Malonic Acid Biosynthesis	accA->PGPT0001695	1
Direct Effects	Biofertilization	Potassium Solubilization	K-Solubilization-Organic Acid Metabolism	K-Solubilisation-Malonic Acid Biosynthesis	accB|bccP->PGPT0001700	3
Direct Effects	Biofertilization	Potassium Solubilization	K-Solubilization-Organic Acid Metabolism	K-Solubilisation-Malonic Acid Biosynthesis	accC->PGPT0001705	3
Direct Effects	Biofertilization	Potassium Solubilization	K-Solubilization-Organic Acid Metabolism	K-Solubilisation-Malonic Acid Biosynthesis	accD->PGPT0001710	1
Direct Effects	Biofertilization	Potassium Solubilization	K-Solubilization-Organic Acid Metabolism	K-Solubilisation-Malonic Acid Biosynthesis	pccB->PGPT0001712	1
Direct	Biofertiliz	Potassium	K-Solubilization-	K-Solubilisation-	mdcF->PGPT0001720	2

Effects	ation	Solubilization	Organic Acid Metabolism	Malonic Acid transport		
Direct Effects	Biofertiliz ation	Potassium Solubilization	K-Solubilization-Organic Acid Metabolism	K-Solubilisation-OxalAcetic Acid Biosynthesis	aceB|glcB->PGPT0001445	1
Direct Effects	Biofertiliz ation	Potassium Solubilization	K-Solubilization-Organic Acid Metabolism	K-Solubilisation-OxalAcetic Acid Biosynthesis	mdh->PGPT0001435	1
Direct Effects	Biofertiliz ation	Potassium Solubilization	K-Solubilization-Organic Acid Metabolism	K-Solubilisation-OxalAcetic Acid Biosynthesis	mqo->PGPT0001440	2
Direct Effects	Biofertiliz ation	Potassium Solubilization	K-Solubilization-Organic Acid Metabolism	K-Solubilisation-OxalAcetic Acid Biosynthesis	pckA->PGPT0001405	1
Direct Effects	Biofertiliz ation	Potassium Solubilization	K-Solubilization-Organic Acid Metabolism	K-Solubilisation-OxalAcetic Acid Biosynthesis	pyc->PGPT0001420	1
Direct Effects	Biofertiliz ation	Potassium Solubilization	K-Solubilization-Organic Acid Metabolism	K-Solubilisation-OxalAcetic Acid transport	dctA->PGPT0001450	2
Direct Effects	Biofertiliz ation	Potassium Solubilization	K-Solubilization-Organic Acid Metabolism	K-Solubilisation-Propionic Acid Biosynthesis	ackA->PGPT0001360	1
Direct Effects	Biofertiliz ation	Potassium Solubilization	K-Solubilization-Organic Acid Metabolism	K-Solubilisation-Propionic Acid Biosynthesis	acs->PGPT0002265	2
Direct Effects	Biofertiliz ation	Potassium Solubilization	K-Solubilization-Organic Acid Metabolism	K-Solubilisation-Propionic Acid Biosynthesis	acuI|yhdH->PGPT0002275	1
Direct Effects	Biofertiliz ation	Potassium Solubilization	K-Solubilization-Organic Acid Metabolism	K-Solubilisation-Propionic Acid Biosynthesis	bcd->PGPT0002285	8

Direct Effects	Biofertiliz ation	Potassium Solubilization	K-Solubilization-Organic Acid Metabolism	K-Solubilisation-Propionic Acid Biosynthesis	mmdA->PGPT0002300	1
Direct Effects	Biofertiliz ation	Potassium Solubilization	K-Solubilization-Organic Acid Metabolism	K-Solubilisation-Propionic Acid Biosynthesis	mmsA|iolA->PGPT0002295	4
Direct Effects	Biofertiliz ation	Potassium Solubilization	K-Solubilization-Organic Acid Metabolism	K-Solubilisation-Propionic Acid Biosynthesis	pta->PGPT0001365	1
Direct Effects	Biofertiliz ation	Potassium Solubilization	K-Solubilization-Organic Acid Metabolism	K-Solubilisation-Pyruvic Acid Biosynthesis	cuyA->PGPT0002000	1
Direct Effects	Biofertiliz ation	Potassium Solubilization	K-Solubilization-Organic Acid Metabolism	K-Solubilisation-Pyruvic Acid Biosynthesis	dapA|mosA->PGPT0002080	3
Direct Effects	Biofertiliz ation	Potassium Solubilization	K-Solubilization-Organic Acid Metabolism	K-Solubilisation-Pyruvic Acid Biosynthesis	dat->PGPT0001915	1
Direct Effects	Biofertiliz ation	Potassium Solubilization	K-Solubilization-Organic Acid Metabolism	K-Solubilisation-Pyruvic Acid Biosynthesis	dld->PGPT0001890	2
Direct Effects	Biofertiliz ation	Potassium Solubilization	K-Solubilization-Organic Acid Metabolism	K-Solubilisation-Pyruvic Acid Biosynthesis	dsdA->PGPT0001970	1
Direct Effects	Biofertiliz ation	Potassium Solubilization	K-Solubilization-Organic Acid Metabolism	K-Solubilisation-Pyruvic Acid Biosynthesis	eda->PGPT0002060	1
Direct Effects	Biofertiliz ation	Potassium Solubilization	K-Solubilization-Organic Acid Metabolism	K-Solubilisation-Pyruvic Acid Biosynthesis	ilvA|tdcB->PGPT0001960	1
Direct Effects	Biofertiliz ation	Potassium Solubilization	K-Solubilization-Organic Acid	K-Solubilisation-Pyruvic Acid	ldh->PGPT0001760	1

			Metabolism	Biosynthesis		
Direct Effects	Biofertilization	Potassium Solubilization	K-Solubilization-Organic Acid Metabolism	K-Solubilisation-Pyruvic Acid Biosynthesis	ligK|galC->PGPT0002085	5
Direct Effects	Biofertilization	Potassium Solubilization	K-Solubilization-Organic Acid Metabolism	K-Solubilisation-Pyruvic Acid Biosynthesis	maeA|sfcA|ywkA->PGPT0001350	7
Direct Effects	Biofertilization	Potassium Solubilization	K-Solubilization-Organic Acid Metabolism	K-Solubilisation-Pyruvic Acid Biosynthesis	mccB->PGPT0001985	2
Direct Effects	Biofertilization	Potassium Solubilization	K-Solubilization-Organic Acid Metabolism	K-Solubilisation-Pyruvic Acid Biosynthesis	metC->PGPT0001995	2
Direct Effects	Biofertilization	Potassium Solubilization	K-Solubilization-Organic Acid Metabolism	K-Solubilisation-Pyruvic Acid Biosynthesis	mhpE->PGPT0002050	1
Direct Effects	Biofertilization	Potassium Solubilization	K-Solubilization-Organic Acid Metabolism	K-Solubilisation-Pyruvic Acid Biosynthesis	nagK->PGPT0001620	1
Direct Effects	Biofertilization	Potassium Solubilization	K-Solubilization-Organic Acid Metabolism	K-Solubilisation-Pyruvic Acid Biosynthesis	patB|malY->PGPT0001990	1
Direct Effects	Biofertilization	Potassium Solubilization	K-Solubilization-Organic Acid Metabolism	K-Solubilisation-Pyruvic Acid Biosynthesis	ppdK->PGPT0002040	1
Direct Effects	Biofertilization	Potassium Solubilization	K-Solubilization-Organic Acid Metabolism	K-Solubilisation-Pyruvic Acid Biosynthesis	pps|ppsA->PGPT0002035	2
Direct Effects	Biofertilization	Potassium Solubilization	K-Solubilization-Organic Acid Metabolism	K-Solubilisation-Pyruvic Acid Biosynthesis	ptsI->PGPT0002025	1
Direct	Biofertiliz	Potassium	K-Solubilization-	K-Solubilisation-	pyk->PGPT0002020	2

Effects	ation	Solubilization	Organic Acid Metabolism	Pyruvic Acid Biosynthesis		
Direct Effects	Biofertiliz ation	Potassium Solubilization	K-Solubilization-Organic Acid Metabolism	K-Solubilisation-Pyruvic Acid Biosynthesis	sdaA|sdaB|tdcG->PGPT0001975	2
Direct Effects	Biofertiliz ation	Potassium Solubilization	K-Solubilization-Organic Acid Metabolism	K-Solubilisation-Pyruvic Acid Biosynthesis	sseA->PGPT0002045	2
Direct Effects	Biofertiliz ation	Potassium Solubilization	K-Solubilization-Organic Acid Metabolism	K-Solubilisation-Pyruvic Acid Biosynthesis	tpav|bioA|yhxA->PGPT0001925	2
Direct Effects	Biofertiliz ation	Potassium Solubilization	K-Solubilization-Organic Acid Metabolism	K-Solubilisation-Pyruvic Acid Biosynthesis	ttuC|dmlA->PGPT0001905	1
Direct Effects	Biofertiliz ation	Potassium Solubilization	K-Solubilization-Organic Acid Metabolism	K-Solubilisation-Succinic Acid Acid Biosynthesis	aceA->PGPT0001550	1
Direct Effects	Biofertiliz ation	Potassium Solubilization	K-Solubilization-Organic Acid Metabolism	K-Solubilisation-Succinic Acid Acid Biosynthesis	frdA->PGPT0001115	3
Direct Effects	Biofertiliz ation	Potassium Solubilization	K-Solubilization-Organic Acid Metabolism	K-Solubilisation-Succinic Acid Acid Biosynthesis	gabD->PGPT0001580	5
Direct Effects	Biofertiliz ation	Potassium Solubilization	K-Solubilization-Organic Acid Metabolism	K-Solubilisation-Succinic Acid Acid Biosynthesis	icd->PGPT0001170	1
Direct Effects	Biofertiliz ation	Potassium Solubilization	K-Solubilization-Organic Acid Metabolism	K-Solubilisation-Succinic Acid Acid Biosynthesis	korA|oorA|OforA->PGPT0001175	1
Direct Effects	Biofertiliz ation	Potassium Solubilization	K-Solubilization-Organic Acid Metabolism	K-Solubilisation-Succinic Acid Acid Biosynthesis	korB|oorB|OforB->PGPT0001180	1

Direct Effects	Biofertilization	Potassium Solubilization	K-Solubilization-Organic Acid Metabolism	K-Solubilisation-Succinic Acid Acid Biosynthesis	prpB->PGPT0001555	1
Direct Effects	Biofertilization	Potassium Solubilization	K-Solubilization-Organic Acid Metabolism	K-Solubilisation-Succinic Acid Acid Biosynthesis	sucA->PGPT0001530	1
Direct Effects	Biofertilization	Potassium Solubilization	K-Solubilization-Organic Acid Metabolism	K-Solubilisation-Succinic Acid Acid Biosynthesis	sucB->PGPT0001535	2
Direct Effects	Biofertilization	Potassium Solubilization	K-Solubilization-Organic Acid Metabolism	K-Solubilisation-Succinic Acid Acid Biosynthesis	sucD->PGPT0001540	1
Direct Effects	Biofertilization	Potassium Solubilization	K-Solubilization-Organic Acid Metabolism	K-Solubilisation-Succinic Acid Acid transport	dctA->PGPT0001450	2
Direct Effects	Biofertilization	Potassium Solubilization	K-Solubilization-Organic Acid Metabolism	K-Solubilisation-Tartaric Acid Biosynthesis	ttuC|dmlA->PGPT0001905	1
Direct Effects	Biofertilization	Potassium Solubilization	K-Solubilization-Organic Acid Metabolism	K-Solubilisation-Tartaric Acid transport	dctA->PGPT0001450	2
Direct Effects	Biofertilization	Potassium Solubilization	K-Solubilization-Organic Acid Metabolism	K-Solubilisation-Valeric Acid Biosynthesis	actA->PGPT0001885	1
Direct Effects	Biofertilization	Potassium Solubilization	K-Solubilization-Organic Acid Metabolism	K-Solubilisation-Valeric Acid Biosynthesis	ilvD->PGPT0001875	1
Direct Effects	Biofertilization	Potassium Solubilization	K-Solubilization-Organic Acid Metabolism	K-Solubilisation-Valeric Acid Biosynthesis	paaF|echA->PGPT0001860	1
Direct Effects	Biofertilization	Potassium Solubilization	K-Solubilization-Organic Acid	K-Solubilisation D Gluconate	gnl->PGPT0001285	1

			Metabolism	Biosynthesis		
Direct Effects	Biofertilization	Potassium Solubilization	K-Solubilization-Potassium transport	K-Solubilization-TRK-Uptake System	kch|trkA|mthK|pch->PGPT0002715	1
Direct Effects	Biofertilization	Potassium Solubilization	K-Solubilization-Potassium transport	K-Solubilization-TRK-Uptake System	trkA|ktrA->PGPT0002710	2
Direct Effects	Biofertilization	Potassium Solubilization	K-Solubilization-Potassium transport	K-Solubilization-TRK-Uptake System	trkG|trkH|ktrB->PGPT0002735	3
Direct Effects	Biofertilization	Sulfur Assimilation|Mineralization	S-Assimilation-Sulfur Metabolism	S-Metabolism-AlkaneSulfonate Degradation	ssuD->PGPT0003040	7
Direct Effects	Biofertilization	Sulfur Assimilation|Mineralization	S-Assimilation-Sulfur Metabolism	S-Metabolism-AlkaneSulfonate Degradation	ssuE->PGPT0003045	1
Direct Effects	Biofertilization	Sulfur Assimilation|Mineralization	S-Assimilation-Sulfur Metabolism	S-Metabolism-AlkaneSulfonate transport	ssuA->PGPT0003025	1
Direct Effects	Biofertilization	Sulfur Assimilation|Mineralization	S-Assimilation-Sulfur Metabolism	S-Metabolism-AlkaneSulfonate transport	ssuB->PGPT0003030	1
Direct Effects	Biofertilization	Sulfur Assimilation|Mineralization	S-Assimilation-Sulfur Metabolism	S-Metabolism-AlkaneSulfonate transport	ssuC->PGPT0003035	1
Direct Effects	Biofertilization	Sulfur Assimilation|Mineralization	S-Assimilation-Sulfur Metabolism	S-Metabolism-DMS Degradation	dmdB->PGPT0002895	1
Direct Effects	Biofertilization	Sulfur Assimilation|Mineralization	S-Assimilation-Sulfur Metabolism	S-Metabolism-Sulfate Reduction	cysC->PGPT0002780	2
Direct Effects	Biofertilization	Sulfur Assimilation|Mineralization	S-Assimilation-Sulfur Metabolism	S-Metabolism-Sulfate Reduction	cysH->PGPT0002785	1
Direct	Biofertiliz	Sulfur	S-Assimilation-Sulfur	S-Metabolism-Sulfate	cysI->PGPT0002790	1

Effects	ation	Assimilation|Mineralization	Metabolism	Reduction		
Direct Effects	Biofertilization	Sulfur Assimilation|Mineralization	S-Assimilation-Sulfur Metabolism	S-Metabolism-Sulfate Reduction	cysJ->PGPT0002795	1
Direct Effects	Biofertilization	Sulfur Assimilation|Mineralization	S-Assimilation-Sulfur Metabolism	S-Metabolism-Sulfate Reduction	cysK->PGPT0002810	3
Direct Effects	Biofertilization	Sulfur Assimilation|Mineralization	S-Assimilation-Sulfur Metabolism	S-Metabolism-Sulfate Reduction	cysK2->PGPT0002815	1
Direct Effects	Biofertilization	Sulfur Assimilation|Mineralization	S-Assimilation-Sulfur Metabolism	S-Metabolism-Sulfate Reduction	sat|met3->PGPT0002410	1
Direct Effects	Biofertilization	Sulfur Assimilation|Mineralization	S-Assimilation-Sulfur Metabolism	S-Metabolism-Sulfate transport	TC SULP->PGPT0003020	4
Direct Effects	Biofertilization	Sulfur Assimilation|Mineralization	S-Assimilation-Sulfur Metabolism	S-Metabolism-Sulfate transport	cysA->PGPT0002990	1
Direct Effects	Biofertilization	Sulfur Assimilation|Mineralization	S-Assimilation-Sulfur Metabolism	S-Metabolism-Sulfate transport	cysT|cysU->PGPT0003000	1
Direct Effects	Biofertilization	Sulfur Assimilation|Mineralization	S-Assimilation-Sulfur Metabolism	S-Metabolism-Sulfate transport	cysW->PGPT0003005	1
Direct Effects	Biofertilization	Sulfur Assimilation|Mineralization	S-Assimilation-Sulfur Metabolism	S-Metabolism-Sulfate transport	sbp->PGPT0003015	1
Direct Effects	Biofertilization	Sulfur Assimilation|Mineralization	S-Assimilation-Sulfur Metabolism	S-Metabolism-Sulfone Degradation	sfnG->PGPT0002830	1

Direct Effects	Biofertilization	Sulfur Assimilation|Mineralization	S-Assimilation-Sulfur Metabolism	S-Metabolism-Sulfur Homeostasis	nifS|iscS->PGPT0000065	4
Direct Effects	Biofertilization	Sulfur Assimilation|Mineralization	S-Assimilation-Sulfur Metabolism	S-Metabolism-Sulfur Homeostasis	sseA->PGPT0002045	2
Direct Effects	Biofertilization	Sulfur Assimilation|Mineralization	S-Assimilation-Sulfur Metabolism	S-Metabolism-Sulfur Regulation	cysE->PGPT0002970	1
Direct Effects	Biofertilization	Sulfur Assimilation|Mineralization	S-Assimilation-Sulfur Metabolism	S-Metabolism-Sulfur Regulation	cysS->PGPT0002985	1
Direct Effects	Biofertilization	Sulfur Assimilation|Mineralization	S-Assimilation-Sulfur Metabolism	S-Metabolism-Taurine Degradation	ggt->PGPT0002935	6
Direct Effects	#¡VALOR!	Fluoride Detoxification	Fluoride Resistance	Fluoride Resistance-Fluoride transport	TC CIC|eriC->PGPT0004990	1
Direct Effects	#¡VALOR!	Fluoride Detoxification	Fluoride Resistance	Fluoride Resistance-Fluoride transport	crcB->PGPT0004985	2
Direct Effects	#¡VALOR!	Heavy Metal Detoxification	Heavy Metal Antimony Resistance	Antimony Resistance-Antimony Homeostasis	iscR->PGPT0004960	3
Direct Effects	#¡VALOR!	Heavy Metal Detoxification	Heavy Metal Antimony Resistance	Antimony Resistance-Antimony transport	glpF|pduF->PGPT0004760	1
Direct Effects	#¡VALOR!	Heavy Metal Detoxification	Heavy Metal Antimony Resistance	Antimony Resistance-Putative PST-PHO-PIT transport System	pstA->PGPT0002610	1
Direct Effects	#¡VALOR!	Heavy Metal Detoxification	Heavy Metal Antimony Resistance	Antimony Resistance-Putative PST-PHO-PIT transport System	pstB|phoT->PGPT0002615	1
Direct Effects	#¡VALOR!	Heavy Metal Detoxification	Heavy Metal Antimony Resistance	Antimony Resistance-Putative PST-PHO-PIT	pstC|phoW->PGPT0002620	1

				transport System		
Direct Effects	#¡VALOR!	Heavy Metal Detoxification	Heavy Metal Antimony Resistance	Antimony Resistance-Putative PST-PHO-PIT transport System	pstS|phoS->PGPT0002625	1
Direct Effects	#¡VALOR!	Heavy Metal Detoxification	Heavy Metal Antimony Resistance	Antimony Resistance System	arsA->PGPT0004700	1
Direct Effects	#¡VALOR!	Heavy Metal Detoxification	Heavy Metal Antimony Resistance	Antimony Resistance System	arsB|Arsenical Pump Membrane Protein->PGPT0004710	5
Direct Effects	#¡VALOR!	Heavy Metal Detoxification	Heavy Metal Antimony Resistance	Antimony Resistance System	arsC->PGPT0004715	2
Direct Effects	#¡VALOR!	Heavy Metal Detoxification	Heavy Metal Antimony Resistance	Antimony Resistance System	arsC1->PGPT0004720	1
Direct Effects	#¡VALOR!	Heavy Metal Detoxification	Heavy Metal Antimony Resistance	Antimony Resistance System	arsR->PGPT0004735	12
Direct Effects	#¡VALOR!	Heavy Metal Detoxification	Heavy Metal Arsenic Resistance	Arsenic Resistance-Arsenic transport	aqpZ->PGPT0004755	1
Direct Effects	#¡VALOR!	Heavy Metal Detoxification	Heavy Metal Arsenic Resistance	Arsenic Resistance-Arsenic transport	glpF|pduF->PGPT0004760	1
Direct Effects	#¡VALOR!	Heavy Metal Detoxification	Heavy Metal Arsenic Resistance	Arsenic Resistance-PST-PHO-PIT transport System	phoU|phoY->PGPT0002630	2
Direct Effects	#¡VALOR!	Heavy Metal Detoxification	Heavy Metal Arsenic Resistance	Arsenic Resistance-PST-PHO-PIT transport System	pstA->PGPT0002610	1
Direct Effects	#¡VALOR!	Heavy Metal Detoxification	Heavy Metal Arsenic Resistance	Arsenic Resistance-PST-PHO-PIT transport System	pstB|phoT->PGPT0002615	1
Direct Effects	#¡VALOR!	Heavy Metal Detoxification	Heavy Metal Arsenic Resistance	Arsenic Resistance-PST-PHO-PIT transport System	pstC|phoW->PGPT0002620	1
Direct	#¡VALOR!	Heavy Metal	Heavy Metal Arsenic	Arsenic Resistance-	pstS|phoS-	1

Effects		Detoxification	Resistance	PST-PHO-PIT transport System	>PGPT0002625	
Direct Effects	#¡VALOR!	Heavy Metal Detoxification	Heavy Metal Arsenic Resistance	Arsenic Resistance System	arsA->PGPT0004700	1
Direct Effects	#¡VALOR!	Heavy Metal Detoxification	Heavy Metal Arsenic Resistance	Arsenic Resistance System	arsB|Arsenical Pump Membrane Protein->PGPT0004710	5
Direct Effects	#¡VALOR!	Heavy Metal Detoxification	Heavy Metal Arsenic Resistance	Arsenic Resistance System	arsC->PGPT0004715	2
Direct Effects	#¡VALOR!	Heavy Metal Detoxification	Heavy Metal Arsenic Resistance	Arsenic Resistance System	arsC1->PGPT0004720	1
Direct Effects	#¡VALOR!	Heavy Metal Detoxification	Heavy Metal Arsenic Resistance	Arsenic Resistance System	arsR->PGPT0004735	12
Direct Effects	#¡VALOR!	Heavy Metal Detoxification	Heavy Metal Bismuth Resistance	Bismuth Resistance-Bismuth Homeostasis	arsR->PGPT0004735	12
Direct Effects	#¡VALOR!	Heavy Metal Detoxification	Heavy Metal Bismuth Resistance	Bismuth Resistance-Bismuth Homeostasis	cadC|smtB->PGPT0004290	2
Direct Effects	#¡VALOR!	Heavy Metal Detoxification	Heavy Metal Cadmium Resistance	Cadmium Resistance-Cadmium Homeostasis	cadC|smtB->PGPT0004290	2
Direct Effects	#¡VALOR!	Heavy Metal Detoxification	Heavy Metal Cadmium Resistance	Cadmium Resistance-Cadmium transport	czcD|zitB|yrdO->PGPT0004255	3
Direct Effects	#¡VALOR!	Heavy Metal Detoxification	Heavy Metal Cadmium Resistance	Cadmium Resistance-Cadmium transport	zntA|cadA->PGPT0004200	3
Direct Effects	#¡VALOR!	Heavy Metal Detoxification	Heavy Metal Chromate Resistance	Chromate Resistance-CHR transport System	chrC|sodB|sodA->PGPT0004190	2
Direct Effects	#¡VALOR!	Heavy Metal Detoxification	Heavy Metal Chromate Resistance	Chromate Resistance-CHR transport System	chrR->PGPT0004195	1
Direct Effects	#¡VALOR!	Heavy Metal Detoxification	Heavy Metal Chromate Resistance	Chromate Resistance-CHR transport System	chrS->PGPT0004196	1
Direct Effects	#¡VALOR!	Heavy Metal Detoxification	Heavy Metal Cobalt Resistance	Cobalt Resistance-CBI transport System	cbiL->PGPT0004690	1

Direct Effects	#¡VALOR!	Heavy Metal Detoxification	Heavy Metal Cobalt Resistance	Cobalt Resistance-CBI transport System	cbiO->PGPT0004455	2
Direct Effects	#¡VALOR!	Heavy Metal Detoxification	Heavy Metal Cobalt Resistance	Cobalt Resistance-Cobalt Homeostasis	cbiA->PGPT0004605	1
Direct Effects	#¡VALOR!	Heavy Metal Detoxification	Heavy Metal Cobalt Resistance	Cobalt Resistance-Cobalt Homeostasis	cbiB|cobD->PGPT0004660	1
Direct Effects	#¡VALOR!	Heavy Metal Detoxification	Heavy Metal Cobalt Resistance	Cobalt Resistance-Cobalt Homeostasis	cbiC->PGPT0004610	1
Direct Effects	#¡VALOR!	Heavy Metal Detoxification	Heavy Metal Cobalt Resistance	Cobalt Resistance-Cobalt Homeostasis	cbiD->PGPT0004615	1
Direct Effects	#¡VALOR!	Heavy Metal Detoxification	Heavy Metal Cobalt Resistance	Cobalt Resistance-Cobalt Homeostasis	cbiF->PGPT0004625	1
Direct Effects	#¡VALOR!	Heavy Metal Detoxification	Heavy Metal Cobalt Resistance	Cobalt Resistance-Cobalt Homeostasis	cbiG->PGPT0004630	1
Direct Effects	#¡VALOR!	Heavy Metal Detoxification	Heavy Metal Cobalt Resistance	Cobalt Resistance-Cobalt Homeostasis	cbiJ->PGPT0004640	1
Direct Effects	#¡VALOR!	Heavy Metal Detoxification	Heavy Metal Cobalt Resistance	Cobalt Resistance-Cobalt Homeostasis	cobA|btuR->PGPT0004645	1
Direct Effects	#¡VALOR!	Heavy Metal Detoxification	Heavy Metal Cobalt Resistance	Cobalt Resistance-Cobalt Homeostasis	cobC|phpB->PGPT0004650	1
Direct Effects	#¡VALOR!	Heavy Metal Detoxification	Heavy Metal Cobalt Resistance	Cobalt Resistance-Cobalt Homeostasis	cobP|cobU->PGPT0004585	1
Direct Effects	#¡VALOR!	Heavy Metal Detoxification	Heavy Metal Cobalt Resistance	Cobalt Resistance-Cobalt Homeostasis	cobQ|cbiP->PGPT0004580	1
Direct Effects	#¡VALOR!	Heavy Metal Detoxification	Heavy Metal Cobalt Resistance	Cobalt Resistance-Cobalt Homeostasis	cobS|cobV->PGPT0004590	1
Direct Effects	#¡VALOR!	Heavy Metal Detoxification	Heavy Metal Cobalt Resistance	Cobalt Resistance-Cobalt Homeostasis	cobU|cobT->PGPT0004595	3
Direct Effects	#¡VALOR!	Heavy Metal Detoxification	Heavy Metal Cobalt Resistance	Cobalt Resistance-Cobalt Homeostasis	rhnA cobC->PGPT0004575	1
Direct Effects	#¡VALOR!	Heavy Metal Detoxification	Heavy Metal Cobalt Resistance	Cobalt Resistance-Cobalt transport	corC->PGPT0004695	1

Direct Effects	#¡VALOR!	Heavy Metal Detoxification	Heavy Metal Cobalt Resistance	Cobalt Resistance-Cobalt transport	czcD|zitB|yrdO->PGPT0004255	3
Direct Effects	#¡VALOR!	Heavy Metal Detoxification	Heavy Metal Copper Resistance	Copper Resistance-Copper transport	copA|ctpA->PGPT0004090	2
Direct Effects	#¡VALOR!	Heavy Metal Detoxification	Heavy Metal Copper Resistance	Copper Resistance-Copper transport	cutC->PGPT0004085	2
Direct Effects	#¡VALOR!	Heavy Metal Detoxification	Heavy Metal Copper Resistance	Copper Resistance-Related Functions	csoR|ricR->PGPT0004175	3
Direct Effects	#¡VALOR!	Heavy Metal Detoxification	Heavy Metal Copper Resistance	Copper Resistance-Related Functions	cueR->PGPT0004165	1
Direct Effects	#¡VALOR!	Heavy Metal Detoxification	Heavy Metal Copper Resistance	Copper Resistance-Related Functions	mhqA|mhqE|mhqO|yaiA->PGPT0028510	3
Direct Effects	#¡VALOR!	Heavy Metal Detoxification	Heavy Metal Copper Resistance	Copper Resistance Mediation	AToX1|ATX1|copZ|golB->PGPT0004125	1
Direct Effects	#¡VALOR!	Heavy Metal Detoxification	Heavy Metal Copper Resistance	Copper Resistance Mediation	comR|ycfQ->PGPT0004136	2
Direct Effects	#¡VALOR!	Heavy Metal Detoxification	Heavy Metal Copper Resistance	Copper Resistance Mediation	cueR->PGPT0004135	9
Direct Effects	#¡VALOR!	Heavy Metal Detoxification	Heavy Metal Copper Resistance	Copper Resistance Mediation	cusR|copR|silR->PGPT0004105	1
Direct Effects	#¡VALOR!	Heavy Metal Detoxification	Heavy Metal Copper Resistance	Copper Resistance Mediation	cusS|copS|silS->PGPT0004100	5
Direct Effects	#¡VALOR!	Heavy Metal Detoxification	Heavy Metal Copper Resistance	Copper Resistance Mediation	dps|dpsA->PGPT0004055	2
Direct Effects	#¡VALOR!	Heavy Metal Detoxification	Heavy Metal Copper Resistance	Copper Resistance Mediation	pcoC|copC->PGPT0004115	1
Direct Effects	#¡VALOR!	Heavy Metal Detoxification	Heavy Metal Copper Resistance	Copper Resistance Mediation	pcoD|copD->PGPT0004120	2
Direct Effects	#¡VALOR!	Heavy Metal Detoxification	Heavy Metal Gold Resistance	Gold Resistance-Dleftibactin Metabolism	Dleftibactin Hydrolase Domain Protein->PGPT0004151	1
Direct	#¡VALOR!	Heavy Metal	Heavy Metal Iron	Iron Resistance-	acbB->PGPT0003152	1

<table>
<tr><td>Effects</td><td></td><td>Detoxification</td><td>Resistance</td><td>AcineToferrin Biosynthesis</td><td></td><td></td></tr>
<tr><td>Direct Effects</td><td>#¡VALOR!</td><td>Heavy Metal Detoxification</td><td>Heavy Metal Iron Resistance</td><td>Iron Resistance-Bacillibactin Metabolism</td><td>mta|ywnD->PGPT0003175</td><td>1</td></tr>
<tr><td>Direct Effects</td><td>#¡VALOR!</td><td>Heavy Metal Detoxification</td><td>Heavy Metal Iron Resistance</td><td>Iron Resistance-Bacillibactin Metabolism</td><td>ymfI|fabG|efpI->PGPT0003180</td><td>14</td></tr>
<tr><td>Direct Effects</td><td>#¡VALOR!</td><td>Heavy Metal Detoxification</td><td>Heavy Metal Iron Resistance</td><td>Iron Resistance-Bacillibactin transport</td><td>blt->PGPT0003185</td><td>5</td></tr>
<tr><td>Direct Effects</td><td>#¡VALOR!</td><td>Heavy Metal Detoxification</td><td>Heavy Metal Iron Resistance</td><td>Iron Resistance-Bacillibactin transport</td><td>ydfK|yqgA->PGPT0003195</td><td>1</td></tr>
<tr><td>Direct Effects</td><td>#¡VALOR!</td><td>Heavy Metal Detoxification</td><td>Heavy Metal Iron Resistance</td><td>Iron Resistance-BacteriOferritin-Associated Ferredoxin</td><td>bfd|yheA->PGPT0003975</td><td>1</td></tr>
<tr><td>Direct Effects</td><td>#¡VALOR!</td><td>Heavy Metal Detoxification</td><td>Heavy Metal Iron Resistance</td><td>Iron Resistance-Bisucaberin Biosynthesis</td><td>bsbB->PGPT0031170</td><td>1</td></tr>
<tr><td>Direct Effects</td><td>#¡VALOR!</td><td>Heavy Metal Detoxification</td><td>Heavy Metal Iron Resistance</td><td>Iron Resistance-Catechol Related Ferric Citrate transport</td><td>fecB|htsA->PGPT0003800</td><td>1</td></tr>
<tr><td>Direct Effects</td><td>#¡VALOR!</td><td>Heavy Metal Detoxification</td><td>Heavy Metal Iron Resistance</td><td>Iron Resistance-Catechol Related Ferric Citrate transport</td><td>fecC|htsB->PGPT0003805</td><td>2</td></tr>
<tr><td>Direct Effects</td><td>#¡VALOR!</td><td>Heavy Metal Detoxification</td><td>Heavy Metal Iron Resistance</td><td>Iron Resistance-Catechol Related Ferric Citrate transport</td><td>fecD|htsC->PGPT0003810</td><td>2</td></tr>
<tr><td>Direct Effects</td><td>#¡VALOR!</td><td>Heavy Metal Detoxification</td><td>Heavy Metal Iron Resistance</td><td>Iron Resistance-Catechol Related Ferric Citrate transport</td><td>fecE->PGPT0003815</td><td>1</td></tr>
<tr><td>Direct Effects</td><td>#¡VALOR!</td><td>Heavy Metal Detoxification</td><td>Heavy Metal Iron Resistance</td><td>Iron Resistance-Coprogen Biosynthesis</td><td>hemN|hemZ->PGPT0003200</td><td>2</td></tr>
</table>

Direct Effects	#¡VALOR!	Heavy Metal Detoxification	Heavy Metal Iron Resistance	Iron Resistance-Cyclooctatin Metabolism	cotB3->PGPT0003075	1
Direct Effects	#¡VALOR!	Heavy Metal Detoxification	Heavy Metal Iron Resistance	Iron Resistance-DesFerrioxAmine-Biosynthesis	ddc|dfoJ|desA->PGPT0013725	1
Direct Effects	#¡VALOR!	Heavy Metal Detoxification	Heavy Metal Iron Resistance	Iron Resistance-Enterobactin|Enterochelin Metabolism	acrR|smeT->PGPT0003255	2
Direct Effects	#¡VALOR!	Heavy Metal Detoxification	Heavy Metal Iron Resistance	Iron Resistance-Enterobactin|Enterochelin Metabolism	fes|cbsF|cbsH->PGPT0003235	1
Direct Effects	#¡VALOR!	Heavy Metal Detoxification	Heavy Metal Iron Resistance	Iron Resistance-Export Systems	ymfE->PGPT0003580	3
Direct Effects	#¡VALOR!	Heavy Metal Detoxification	Heavy Metal Iron Resistance	Iron Resistance-Ferrichrome|FerrioxAmine Metabolism	fhuF->PGPT0003315	1
Direct Effects	#¡VALOR!	Heavy Metal Detoxification	Heavy Metal Iron Resistance	Iron Resistance-FeuABC|YusV-transport Complex	feuA|yvrC|ABC FEVS|fatB->PGPT0003765	7
Direct Effects	#¡VALOR!	Heavy Metal Detoxification	Heavy Metal Iron Resistance	Iron Resistance-FeuABC|YusV-transport Complex	feuB|feuC|chuU|yfhA|hmuU|ABC FEVP|fatC|fatD->PGPT0003770	7
Direct Effects	#¡VALOR!	Heavy Metal Detoxification	Heavy Metal Iron Resistance	Iron Resistance-FeuABC|YusV-transport Complex	yusV|ABC FEVA|feuD->PGPT0003760	4
Direct Effects	#¡VALOR!	Heavy Metal Detoxification	Heavy Metal Iron Resistance	Iron Resistance-Fmn|Dmk|Ppl|Ndh|Eet System	fmnA|ecfT->PGPT0004015	1
Direct Effects	#¡VALOR!	Heavy Metal Detoxification	Heavy Metal Iron Resistance	Iron Resistance-Fmn|Dmk|Ppl|Ndh|	ndh->PGPT0004020	3

				Eet System		
Direct Effects	#¡VALOR!	Heavy Metal Detoxification	Heavy Metal Iron Resistance	Iron Resistance-Fmn|Dmk|Ppl|Ndh|Eet System	nosX|apbE|yojL|fmnB->PGPT0000430	2
Direct Effects	#¡VALOR!	Heavy Metal Detoxification	Heavy Metal Iron Resistance	Iron Resistance-FoxABCD	coxC|ctaE->PGPT0004035	3
Direct Effects	#¡VALOR!	Heavy Metal Detoxification	Heavy Metal Iron Resistance	Iron Resistance-FoxABCD	foxA|coxA|ctaD->PGPT0004025	3
Direct Effects	#¡VALOR!	Heavy Metal Detoxification	Heavy Metal Iron Resistance	Iron Resistance-FoxABCD	foxB|coxB|ctaC->PGPT0004030	3
Direct Effects	#¡VALOR!	Heavy Metal Detoxification	Heavy Metal Iron Resistance	Iron Resistance-Hemophores-Heme|Hemin Utilization	hmoA->PGPT0003630	1
Direct Effects	#¡VALOR!	Heavy Metal Detoxification	Heavy Metal Iron Resistance	Iron Resistance-Hemophores-SiroHeme	cysG->PGPT0003690	1
Direct Effects	#¡VALOR!	Heavy Metal Detoxification	Heavy Metal Iron Resistance	Iron Resistance-Hemophores-SiroHeme	hemA->PGPT0003650	1
Direct Effects	#¡VALOR!	Heavy Metal Detoxification	Heavy Metal Iron Resistance	Iron Resistance-Hemophores-SiroHeme	hemB->PGPT0003655	1
Direct Effects	#¡VALOR!	Heavy Metal Detoxification	Heavy Metal Iron Resistance	Iron Resistance-Hemophores-SiroHeme	hemC->PGPT0003660	1
Direct Effects	#¡VALOR!	Heavy Metal Detoxification	Heavy Metal Iron Resistance	Iron Resistance-Hemophores-SiroHeme	hemD->PGPT0003665	2
Direct Effects	#¡VALOR!	Heavy Metal Detoxification	Heavy Metal Iron Resistance	Iron Resistance-Hemophores-SiroHeme	hemL->PGPT0003680	2

Direct Effects	#¡VALOR!	Heavy Metal Detoxification	Heavy Metal Iron Resistance	Iron Resistance-Hemophores-SiroHeme	sirA|ylnD|cysG|cobA->PGPT0003685	3
Direct Effects	#¡VALOR!	Heavy Metal Detoxification	Heavy Metal Iron Resistance	Iron Resistance-Iron Uptake Regulation	fur|furB|zur->PGPT0003880	1
Direct Effects	#¡VALOR!	Heavy Metal Detoxification	Heavy Metal Iron Resistance	Iron Resistance-Iron Uptake Regulation	pmrA->PGPT0003915	3
Direct Effects	#¡VALOR!	Heavy Metal Detoxification	Heavy Metal Iron Resistance	Iron Resistance-Iron Uptake Regulation	troR->PGPT0003890	1
Direct Effects	#¡VALOR!	Heavy Metal Detoxification	Heavy Metal Iron Resistance	Iron Resistance-Lipoic Acid ChelaTors	lipA->PGPT0003935	1
Direct Effects	#¡VALOR!	Heavy Metal Detoxification	Heavy Metal Iron Resistance	Iron Resistance-Lipoic Acid ChelaTors	lipL->PGPT0003940	1
Direct Effects	#¡VALOR!	Heavy Metal Detoxification	Heavy Metal Iron Resistance	Iron Resistance-Petrobactin Biosynthesis	asbA->PGPT0003545	1
Direct Effects	#¡VALOR!	Heavy Metal Detoxification	Heavy Metal Iron Resistance	Iron Resistance-Related Proteins	dps|dpsA->PGPT0004055	2
Direct Effects	#¡VALOR!	Heavy Metal Detoxification	Heavy Metal Iron Resistance	Iron Resistance-Related Proteins-BaeSR System	baeS->PGPT0003985	1
Direct Effects	#¡VALOR!	Heavy Metal Detoxification	Heavy Metal Iron Resistance	Iron Resistance-Related Proteins Ferritin	bfr->PGPT0003965	1
Direct Effects	#¡VALOR!	Heavy Metal Detoxification	Heavy Metal Iron Resistance	Iron Resistance-Related Proteins Ferritin	ftnA|ftn->PGPT0003970	1
Direct Effects	#¡VALOR!	Heavy Metal Detoxification	Heavy Metal Iron Resistance	Iron Resistance-Rhizobactin Metabolism	rhbD->PGPT0003404	1
Direct Effects	#¡VALOR!	Heavy Metal Detoxification	Heavy Metal Iron Resistance	Iron Resistance-Rhizobactin	rhbF->PGPT0003407	1

				Metabolism		
Direct Effects	#¡VALOR!	Heavy Metal Detoxification	Heavy Metal Iron Resistance	Iron transport-Iron II transport System	efeB->PGPT0003720	1
Direct Effects	#¡VALOR!	Heavy Metal Detoxification	Heavy Metal Iron Resistance	Iron transport-Iron II transport System	efeO->PGPT0003715	1
Direct Effects	#¡VALOR!	Heavy Metal Detoxification	Heavy Metal Iron Resistance	Iron transport-Iron II transport System	efeU|FTR|FTH1->PGPT0003710	1
Direct Effects	#¡VALOR!	Heavy Metal Detoxification	Heavy Metal Iron Resistance	Iron transport-Iron II transport System	feoA->PGPT0003695	2
Direct Effects	#¡VALOR!	Heavy Metal Detoxification	Heavy Metal Iron Resistance	Iron transport-Iron II transport System	feoB->PGPT0003700	5
Direct Effects	#¡VALOR!	Heavy Metal Detoxification	Heavy Metal Lead Resistance	Lead Resistance-Lead Homeostasis	cadC|smtB->PGPT0004290	2
Direct Effects	#¡VALOR!	Heavy Metal Detoxification	Heavy Metal Lead Resistance	Lead Resistance-PBR transport System	pbrB|pbrC->PGPT0004770	1
Direct Effects	#¡VALOR!	Heavy Metal Detoxification	Heavy Metal Lead Resistance	Lead Resistance-PBR transport System	zntA|cadA->PGPT0004200	3
Direct Effects	#¡VALOR!	Heavy Metal Detoxification	Heavy Metal Manganese Resistance	Manganese Resistance-Homeostasis	troR->PGPT0003890	1
Direct Effects	#¡VALOR!	Heavy Metal Detoxification	Heavy Metal Manganese Resistance	Manganese Resistance-MNT transport System	mntH->PGPT0004370	1
Direct Effects	#¡VALOR!	Heavy Metal Detoxification	Heavy Metal Manganese Resistance	Manganese Resistance-MNT transport System	mntP->PGPT0004375	1
Direct Effects	#¡VALOR!	Heavy Metal Detoxification	Heavy Metal Manganese Resistance	Manganese Resistance-Related Protein	yceF->PGPT0003866	1
Direct Effects	#¡VALOR!	Heavy Metal Detoxification	Heavy Metal Manganese Resistance	Manganese Resistance-Related Protein	ykoY->PGPT0003867	4

Direct Effects	#¡VALOR!	Heavy Metal Detoxification	Heavy Metal Nickel Resistance	Nickel Resistance-CBI transport System	cbiO->PGPT0004455	2
Direct Effects	#¡VALOR!	Heavy Metal Detoxification	Heavy Metal Nickel Resistance	Nickel Resistance-DDP transport System	ddpA|ABC PE S->PGPT0004430	6
Direct Effects	#¡VALOR!	Heavy Metal Detoxification	Heavy Metal Nickel Resistance	Nickel Resistance-DDP transport System	ddpB|appB->PGPT0004445	4
Direct Effects	#¡VALOR!	Heavy Metal Detoxification	Heavy Metal Nickel Resistance	Nickel Resistance-DDP transport System	ddpC|appC->PGPT0004450	4
Direct Effects	#¡VALOR!	Heavy Metal Detoxification	Heavy Metal Nickel Resistance	Nickel Resistance-DDP transport System	ddpD->PGPT0004435	4
Direct Effects	#¡VALOR!	Heavy Metal Detoxification	Heavy Metal Nickel Resistance	Nickel Resistance-DDP transport System	ddpF->PGPT0004440	4
Direct Effects	#¡VALOR!	Heavy Metal Detoxification	Heavy Metal Nickel Resistance	Nickel Resistance-Nickel transport	dppB1->PGPT0004530	1
Direct Effects	#¡VALOR!	Heavy Metal Detoxification	Heavy Metal Nickel Resistance	Nickel Resistance-Nickel transport	dppC1->PGPT0004535	1
Direct Effects	#¡VALOR!	Heavy Metal Detoxification	Heavy Metal Nickel Resistance	Nickel Resistance-Nickel transport	dppD1->PGPT0004540	1
Direct Effects	#¡VALOR!	Heavy Metal Detoxification	Heavy Metal Nickel Resistance	Nickel Resistance-Nickel transport	dppE->PGPT0004525	1
Direct Effects	#¡VALOR!	Heavy Metal Detoxification	Heavy Metal Nickel Resistance	Nickel Resistance-Nickel transport	hoxN|nixA->PGPT0004545	1
Direct Effects	#¡VALOR!	Heavy Metal Detoxification	Heavy Metal Nickel Resistance	Nickel Resistance-NIK-CNT transport System	nikA|cntA->PGPT0004405	1
Direct Effects	#¡VALOR!	Heavy Metal Detoxification	Heavy Metal Nickel Resistance	Nickel Resistance-NIK-CNT transport System	nikB|cntB->PGPT0004410	1
Direct Effects	#¡VALOR!	Heavy Metal Detoxification	Heavy Metal Nickel Resistance	Nickel Resistance-NIK-CNT transport System	nikC|cntC->PGPT0004415	1
Direct	#¡VALOR!	Heavy Metal	Heavy Metal Nickel	Nickel Resistance-	nikD|cntD-	1

Effects		Detoxification	Resistance	NIK-CNT transport System	>PGPT0004420	
Direct Effects	#¡VALOR!	Heavy Metal Detoxification	Heavy Metal Nickel Resistance	Nickel Resistance-NIK-CNT transport System	nikE|cntF->PGPT0004425	1
Direct Effects	#¡VALOR!	Heavy Metal Detoxification	Heavy Metal Selenium Resistance	Selenium Resistance-Selenium transport	cysA->PGPT0002990	1
Direct Effects	#¡VALOR!	Heavy Metal Detoxification	Heavy Metal Selenium Resistance	Selenium Resistance-Selenium transport	dedA->PGPT0004890	4
Direct Effects	#¡VALOR!	Heavy Metal Detoxification	Heavy Metal Tellurium Resistance	Heavy Metal Tellurium Resistance	Tellurium Resistance-Tellurium transporter->PGPT0001400	1
Direct Effects	#¡VALOR!	Heavy Metal Detoxification	Heavy Metal Tellurium Resistance	Tellurium Resistance-TER-System	terD|terE|terF->PGPT0004910	3
Direct Effects	#¡VALOR!	Heavy Metal Detoxification	Heavy Metal Tungstate Resistance	Tungstate Resistance-YCE System	yceG->PGPT0004946	1
Direct Effects	#¡VALOR!	Heavy Metal Detoxification	Heavy Metal Tungstate Resistance	Tungstate Resistance-YCE System	yceH->PGPT0004947	2
Direct Effects	#¡VALOR!	Heavy Metal Detoxification	Heavy Metal Zinc Resistance	Zinc Resistance-Zinc-Nickel transport	TC ZIP|zupT|ZRT3|ZIP2->PGPT0004250	5
Direct Effects	#¡VALOR!	Heavy Metal Detoxification	Heavy Metal Zinc Resistance	Zinc Resistance-Zinc-Nickel transport	czcD|zitB|yrdO->PGPT0004255	3
Direct Effects	#¡VALOR!	Heavy Metal Detoxification	Heavy Metal Zinc Resistance	Zinc Resistance-Zinc Homeostasis	cadC|smtB->PGPT0004290	2
Direct Effects	#¡VALOR!	Heavy Metal Detoxification	Heavy Metal Zinc Resistance	Zinc Resistance-Zinc Homeostasis	czrA|yozA->PGPT0004305	1
Direct Effects	#¡VALOR!	Heavy Metal Detoxification	Heavy Metal Zinc Resistance	Zinc Resistance-Zinc Homeostasis	zurR|zur->PGPT0004295	2
Direct Effects	#¡VALOR!	Heavy Metal Detoxification	Heavy Metal Zinc Resistance	Zinc Resistance-Zinc|Iron-transport	fieF->PGPT0003831	2
Direct	#¡VALOR!	Heavy Metal	Heavy Metal Zinc	Zinc Resistance-ZNT	zntA|cadA-	3

<table>
<tr><td>Effects</td><td></td><td>Detoxification</td><td>Resistance</td><td>transport System</td><td>>PGPT0004200</td><td></td></tr>
<tr><td>Direct Effects</td><td>#¡VALOR!</td><td>Heavy Metal Detoxification</td><td>Heavy Metal Zinc Resistance</td><td>Zinc Resistance-ZNU transport System</td><td>znuA->PGPT0004220</td><td>1</td></tr>
<tr><td>Direct Effects</td><td>#¡VALOR!</td><td>Heavy Metal Detoxification</td><td>Heavy Metal Zinc Resistance</td><td>Zinc Resistance-ZNU transport System</td><td>znuB->PGPT0004225</td><td>1</td></tr>
<tr><td>Direct Effects</td><td>#¡VALOR!</td><td>Heavy Metal Detoxification</td><td>Heavy Metal Zinc Resistance</td><td>Zinc Resistance-ZNU transport System</td><td>znuC->PGPT0004230</td><td>1</td></tr>
<tr><td>Direct Effects</td><td>#¡VALOR!</td><td>Heavy Metal Detoxification</td><td>Other Heavy Metal Resistance Systems</td><td>Heavy Metal Resistance-CUS transport System</td><td>cusR|copR|silR->PGPT0004105</td><td>1</td></tr>
<tr><td>Direct Effects</td><td>#¡VALOR!</td><td>Heavy Metal Detoxification</td><td>Other Heavy Metal Resistance Systems</td><td>Heavy Metal Resistance-CUS transport System</td><td>cusS|copS|silS->PGPT0004100</td><td>5</td></tr>
<tr><td>Direct Effects</td><td>#¡VALOR!</td><td>Heavy Metal Detoxification</td><td>Other Heavy Metal Resistance Systems</td><td>Heavy Metal Resistance-CUS transport System</td><td>cusS|copS|silS->PGPT0004980</td><td>1</td></tr>
<tr><td>Direct Effects</td><td>#¡VALOR!</td><td>Xenobiotics Biodegradation</td><td>Xenobiotic Atrazine|Derivate Degradation</td><td>Xenobiotic Cyanuric Acid Degradation</td><td>atzF->PGPT0001010</td><td>1</td></tr>
<tr><td>Direct Effects</td><td>#¡VALOR!</td><td>Xenobiotics Biodegradation</td><td>Xenobiotic Benzoate|Derivate Degradation</td><td>Xenobiotic Benzoate Degradation VIA Catechol Degradation</td><td>catE->PGPT0005050</td><td>1</td></tr>
<tr><td>Direct Effects</td><td>#¡VALOR!</td><td>Xenobiotics Biodegradation</td><td>Xenobiotic Benzoate|Derivate Degradation</td><td>Xenobiotic Benzoate Degradation VIA b-Ketoadipate Pathway</td><td>pcaC->PGPT0005005</td><td>6</td></tr>
<tr><td>Direct Effects</td><td>#¡VALOR!</td><td>Xenobiotics Biodegradation</td><td>Xenobiotic Benzoate|Derivate Degradation</td><td>Xenobiotic Benzoate Degradation VIA b-Ketoadipate Pathway</td><td>pcaD|catD->PGPT0004995</td><td>3</td></tr>
<tr><td>Direct Effects</td><td>#¡VALOR!</td><td>Xenobiotics Biodegradation</td><td>Xenobiotic Benzoate|Derivate Degradation</td><td>Xenobiotic Nitro-|Aminobenzoate Degradation</td><td>namA->PGPT0005575</td><td>1</td></tr>
<tr><td>Direct</td><td>#¡VALOR!</td><td>Xenobiotics</td><td>Xenobiotic</td><td>Xenobiotic Nitro-</td><td>nbaA->PGPT0005560</td><td>1</td></tr>
</table>

Effects		Biodegradation	Benzoate|Derivate Degradation	|Aminobenzoate Degradation		
Direct Effects	#¡VALOR!	Xenobiotics Biodegradation	Xenobiotic Benzoate|Derivate Degradation	Xenobiotic Phenol Degradation	bsdC->PGPT0005435	1
Direct Effects	#¡VALOR!	Xenobiotics Biodegradation	Xenobiotic CaproLactam|Derivate Degradation	Xenobiotic adipate Degradation	paaF|echA->PGPT0001860	1
Direct Effects	#¡VALOR!	Xenobiotics Biodegradation	Xenobiotic Cyanate Detoxification	Xenobiotic Cyanate Uptake	cynX|yeaN->PGPT0005211	1
Direct Effects	#¡VALOR!	Xenobiotics Biodegradation	Xenobiotic Degradation Of Organo-Phosphorus Insecticides	Xenobiotic OrganoPhosphate Degradation	glpA|glpD->PGPT0006775	1
Direct Effects	#¡VALOR!	Xenobiotics Biodegradation	Xenobiotic Degradation Of Organo-Phosphorus Insecticides	Xenobiotic OrganoPhosphate Degradation	mpd->PGPT0006762	1
Direct Effects	#¡VALOR!	Xenobiotics Biodegradation	Xenobiotic Degradation Of Organo-Phosphorus Insecticides	Xenobiotic OrganoPhosphate Degradation	opaA|pepQ->PGPT0006760	1
Direct Effects	#¡VALOR!	Xenobiotics Biodegradation	Xenobiotic Degradation Of Organo-Phosphorus Insecticides	Xenobiotic OrganoPhosphate Degradation	pepP->PGPT0006770	1
Direct Effects	#¡VALOR!	Xenobiotics Biodegradation	Xenobiotic Degradation Of Organo-Phosphorus Insecticides	Xenobiotic OrganoPhosphate Degradation	php|opd|adpB->PGPT0006755	1
Direct Effects	#¡VALOR!	Xenobiotics Biodegradation	Xenobiotic Degradation Of Other	Xenobiotic Azo Dye Degradation	acpD|azoR->PGPT0006790	5

			Nitro-Compounds			
Direct Effects	#¡VALOR!	Xenobiotics Biodegradation	Xenobiotic Degradation Of Other Nitro-Compounds	Xenobiotic Azo Dye Degradation	azr|azoB->PGPT0006791	1
Direct Effects	#¡VALOR!	Xenobiotics Biodegradation	Xenobiotic Degradation Of Other Nitro-Compounds	Xenobiotic Nitro-Aromate Degradation	bluB|drgA->PGPT0006810	1
Direct Effects	#¡VALOR!	Xenobiotics Biodegradation	Xenobiotic Degradation Of Other Nitro-Compounds	Xenobiotic Nitro-Aromate Degradation	nfnB|nfsB->PGPT0005925	2
Direct Effects	#¡VALOR!	Xenobiotics Biodegradation	Xenobiotic Degradation Of Other Nitro-Compounds	Xenobiotic Nitro-Aromate Degradation	nfrA1|ywcG->PGPT0000290	1
Direct Effects	#¡VALOR!	Xenobiotics Biodegradation	Xenobiotic Degradation Of Other Nitro-Compounds	Xenobiotic Nitro-Aromate Degradation	nfrA2|ycnD->PGPT0000295	1
Direct Effects	#¡VALOR!	Xenobiotics Biodegradation	Xenobiotic Degradation Of Other Nitro-Compounds	Xenobiotic Nitro-Aromate Degradation	ssuE->PGPT0003045	1
Direct Effects	#¡VALOR!	Xenobiotics Biodegradation	Xenobiotic Degradation Of Other Nitro-Compounds	Xenobiotic Nitroalkane|Nitronate Degradation	ncd2|npd|pnoA->PGPT0006800	4
Direct Effects	#¡VALOR!	Xenobiotics Biodegradation	Xenobiotic Detoxification By transport	Xenobiotics transport	ybhF|yadG|ecsA->PGPT0006725	14
Direct Effects	#¡VALOR!	Xenobiotics Biodegradation	Xenobiotic Detoxification By transport	Xenobiotics transport	ybhS|ecsB->PGPT0006730	6
Direct Effects	#¡VALOR!	Xenobiotics Biodegradation	Xenobiotic DIoxin|Derivate Degradation	Xenobiotic OxoPent-eonate|PCDD|PCDF|PCB Degradation	mhpE->PGPT0002050	1
Direct	#¡VALOR!	Xenobiotics	Xenobiotic Fatty	Xenobiotic Fatty	cpxB->PGPT0006740	1

Effects		Biodegradation	Acid|Vitamin|Terpene Degradation	Acid|Vitamin|Terpene Degradation-CyTochrome P450		
Direct Effects	#¡VALOR!	Xenobiotics Biodegradation	Xenobiotic Fatty Acid|Vitamin|Terpene Degradation	Xenobiotic Fatty Acid|Vitamin|Terpene Degradation-CyTochrome P450	cpx|cyp109E1->PGPT0006735	2
Direct Effects	#¡VALOR!	Xenobiotics Biodegradation	Xenobiotic Fatty Acid|Vitamin|Terpene Degradation	Xenobiotic Fatty Acid|Vitamin|Terpene Degradation-CyTochrome P450	cyp106A2|yjiB->PGPT0006750	1
Direct Effects	#¡VALOR!	Xenobiotics Biodegradation	Xenobiotic Hydrocarbons|Oil Degradation	Xenobiotic DiChloroethane Degradation	aldH|dhaS->PGPT0006875	5
Direct Effects	#¡VALOR!	Xenobiotics Biodegradation	Xenobiotic Hydrocarbons|Oil Degradation	Xenobiotic DiChloroethane Degradation	dehI->PGPT0006885	2
Direct Effects	#¡VALOR!	Xenobiotics Biodegradation	Xenobiotic Hydrocarbons|Oil Degradation	Xenobiotic DiChloroethane Degradation	dhaA->PGPT0006855	1
Direct Effects	#¡VALOR!	Xenobiotics Biodegradation	Xenobiotic Hydrocarbons|Oil Degradation	Xenobiotic DiChloropropene Degradation	EC 1 1 1 1|adh->PGPT0006375	1
Direct Effects	#¡VALOR!	Xenobiotics Biodegradation	Xenobiotic Hydrocarbons|Oil Degradation	Xenobiotic DiChloropropene Degradation	aldH|dhaS->PGPT0006875	5
Direct Effects	#¡VALOR!	Xenobiotics Biodegradation	Xenobiotic Hydrocarbons|Oil Degradation	Xenobiotic DiChloropropene Degradation	dhaA->PGPT0006855	1
Direct Effects	#¡VALOR!	Xenobiotics Biodegradation	Xenobiotic Hydrocarbons|Oil Degradation	Xenobiotic DiChloropropene Degradation	frmA|ADH5|adhC->PGPT0006355	2

Direct Effects	#¡VALOR!	Xenobiotics Biodegradation	Xenobiotic Hydrocarbons|Oil Degradation	Xenobiotic DiChloropropene Degradation	yiaY->PGPT0006370	1
Direct Effects	#¡VALOR!	Xenobiotics Biodegradation	Xenobiotic Hydrocarbons|Oil Degradation	Xenobiotic Hexa-|Penta-Chlorocyclohexane Degradation	dhaA->PGPT0006855	1
Direct Effects	#¡VALOR!	Xenobiotics Biodegradation	Xenobiotic Hydrocarbons|Oil Degradation	Xenobiotic Oil|RahmnoLipid Degradation	rhlB->PGPT0006835	2
Direct Effects	#¡VALOR!	Xenobiotics Biodegradation	Xenobiotic Hydrocarbons|Oil Degradation	Xenobiotic TriChlorohexadiene Degradation	dhaA->PGPT0006855	1
Direct Effects	#¡VALOR!	Xenobiotics Biodegradation	Xenobiotic Naphtalene|Derivate Degradation	Xenobiotic Methylnaphtalene Degradation	EC 1 1 1 1|adh->PGPT0006375	1
Direct Effects	#¡VALOR!	Xenobiotics Biodegradation	Xenobiotic Naphtalene|Derivate Degradation	Xenobiotic Methylnaphtalene Degradation	frmA|ADH5|adhC->PGPT0006355	2
Direct Effects	#¡VALOR!	Xenobiotics Biodegradation	Xenobiotic Naphtalene|Derivate Degradation	Xenobiotic Methylnaphtalene Degradation	nmoAB->PGPT0006385	1
Direct Effects	#¡VALOR!	Xenobiotics Biodegradation	Xenobiotic Naphtalene|Derivate Degradation	Xenobiotic Methylnaphtalene Degradation	yiaY->PGPT0006370	1
Direct Effects	#¡VALOR!	Xenobiotics Biodegradation	Xenobiotic Naphtalene|Derivate Degradation	Xenobiotic Naphtalene Degradation	nahD->PGPT0006330	1
Direct Effects	#¡VALOR!	Xenobiotics Biodegradation	Xenobiotic Styrene|Derivate Degradation	Xenobiotic Acrylonitrite|Acrylamide Degradation	EC 3 5 1 4|amiE->PGPT0006115	1
Direct	#¡VALOR!	Xenobiotics	Xenobiotic	Xenobiotic	EC 3 5 1 4|amiE-	1

Effects		Biodegradation	Styrene|Derivate Degradation	PhenylAcetaldoxime Degradation	>PGPT0006115	
Direct Effects	#¡VALOR!	Xenobiotics Biodegradation	Xenobiotic Styrene|Derivate Degradation	Xenobiotic PhenylAcetate Degradation	paaF|echA->PGPT0001860	1
Direct Effects	#¡VALOR!	Xenobiotics Biodegradation	Xenobiotic Styrene|Derivate Degradation	Xenobiotic PhenylAcetate Degradation	paaG->PGPT0006070	3
Direct Effects	#¡VALOR!	Xenobiotics Biodegradation	Xenobiotic Styrene|Derivate Degradation	Xenobiotic PhenylAcetate Degradation	paaH|hbd|fadB|mmgB->PGPT0006075	2
Direct Effects	#¡VALOR!	Xenobiotics Biodegradation	Xenobiotic Styrene|Derivate Degradation	Xenobiotic PhenylAcetate Degradation	paaI->PGPT0006080	1
Direct Effects	#¡VALOR!	Xenobiotics Biodegradation	Xenobiotic Styrene|Derivate Degradation	Xenobiotic Styrene Degradation	catE->PGPT0005050	1
Direct Effects	#¡VALOR!	Xenobiotics Biodegradation	Xenobiotic Toluene|Derivate Degradation	Xenobiotic NitroToluene Degradation	nemA->PGPT0005930	1
Direct Effects	#¡VALOR!	Xenobiotics Biodegradation	Xenobiotic Toluene|Derivate Degradation	Xenobiotic NitroToluene Degradation	nfnB|nfsB->PGPT0005925	2
Direct Effects	#¡VALOR!	Xenobiotics Biodegradation	Xenobiotic Xylene|Derivate Degradation	Xenobiotic Methylcatechol Degradation	catE->PGPT0005050	1
Direct Effects	#¡VALOR!	Xenobiotics Biodegradation	Xenobiotic Xylene|Derivate Degradation	Xenobiotic Methylcatechol Degradation	mhpE->PGPT0002050	1
Direct Effects	PhyTohormone|PlantSignal	PhyTohormone-Abscisic Acid Degradation	PhyTohormone-Auxin|Indole‚Äê3‚Äê Acetic Acid|IAA	PhyTohormone-IAA-Auxin transport Plant Like	Auxin Symporter->PGPT0007208	1

	Production		Metabolism			
Direct Effects	PhyTohormone|PlantSignal Production	PhyTohormone-Abscisic Acid Degradation	PhyTohormone-Auxin|Indole,Äê3,Äê Acetic Acid|IAA Metabolism	PhyTohormone-IAA Pathway	EC 3 5 1 4|amiE->PGPT0006115	1
Direct Effects	PhyTohormone|PlantSignal Production	PhyTohormone-Abscisic Acid Degradation	PhyTohormone-Auxin|Indole,Äê3,Äê Acetic Acid|IAA Metabolism	PhyTohormone-IAA Pathway	iaaT|yedL|ysnE->PGPT0007180	1
Direct Effects	PhyTohormone|PlantSignal Production	PhyTohormone-Abscisic Acid Degradation	PhyTohormone-Auxin|Indole,Äê3,Äê Acetic Acid|IAA Metabolism	PhyTohormone-IAA Pathway	yhcX->PGPT0007175	2
Direct Effects	PhyTohormone|PlantSignal Production	PhyTohormone-Abscisic Acid Degradation	PhyTohormone-Auxin|Indole,Äê3,Äê Acetic Acid|IAA Metabolism	PhyTohormone-IAA Related Indole-3-Acetaldehyde Pathway	aldH|dhaS->PGPT0006875	5
Direct Effects	PhyTohormone|PlantSignal Production	PhyTohormone-Abscisic Acid Degradation	PhyTohormone-Auxin|Indole,Äê3,Äê Acetic Acid|IAA Metabolism	PhyTohormone-IAA Related Indole-3-Acetaldehyde Pathway	betB Homologous->PGPT0007165	4
Direct Effects	PhyTohormone|PlantSignal Production	PhyTohormone-Abscisic Acid Degradation	PhyTohormone-Auxin|Indole,Äê3,Äê Acetic Acid|IAA Metabolism	PhyTohormone-IAA Related Indole-3-Acetaldehyde Pathway	bsdC->PGPT0005435	1

Direct Effects	PhyTohormone|PlantSignal Production	PhyTohormone-Abscisic Acid Degradation	PhyTohormone-Auxin|Indole,Äê3,Äê Acetic Acid|IAA Metabolism	PhyTohormone-IAA Related Indole-3-Acetaldehyde Pathway	poxL->PGPT0007160	2
Direct Effects	PhyTohormone|PlantSignal Production	PhyTohormone-Abscisic Acid Degradation	PhyTohormone-Auxin|Indole,Äê3,Äê Acetic Acid|IAA Metabolism	PhyTohormone-IAA Related Indole-3-Acetaldehyde Pathway	pyc->PGPT0001420	1
Direct Effects	PhyTohormone|PlantSignal Production	PhyTohormone-Abscisic Acid Degradation	PhyTohormone-Auxin|Indole,Äê3,Äê Acetic Acid|IAA Metabolism	PhyTohormone-IAA Related TRP-Dependent Pathway 2	EC 3 5 1 4|amiE->PGPT0006115	1
Direct Effects	PhyTohormone|PlantSignal Production	PhyTohormone-Abscisic Acid Degradation	PhyTohormone-Auxin|Indole,Äê3,Äê Acetic Acid|IAA Metabolism	PhyTohormone-IAA Related TrypTophan Pathway	lysN->PGPT0007135	3
Direct Effects	PhyTohormone|PlantSignal Production	PhyTohormone-Abscisic Acid Degradation	PhyTohormone-Auxin|Indole,Äê3,Äê Acetic Acid|IAA Metabolism	PhyTohormone-IAA Related TrypTophan Pathway	patA->PGPT0007150	1
Direct Effects	PhyTohormone|PlantSignal Production	PhyTohormone-Abscisic Acid Degradation	PhyTohormone-Auxin|Indole,Äê3,Äê Acetic Acid|IAA Metabolism	PhyTohormone-IAA Related TrypTophan Pathway	patA1->PGPT0007155	1
Direct Effects	PhyTohormone|Pla	PhyTohormone-Abscisic Acid	PhyTohormone-Auxin|Indole,Äê3,Äê	PhyTohormone-IAA Related TrypTophan	puuE->PGPT0007140	3

	ntSignal Productio n	Degradation	Acetic Acid|IAA Metabolism	Pathway		
Direct Effects	PhyTohor mone|Pla ntSignal Productio n	PhyTohormone-Abscisic Acid Degradation	PhyTohormone-Auxin|Indole,Äê3,Äê Acetic Acid|IAA Metabolism	PhyTohormone-IAA Related TrypTophan Pathway	tpav|bioA|yhxA->PGPT0001925	2
Direct Effects	PhyTohor mone|Pla ntSignal Productio n	PhyTohormone-Abscisic Acid Degradation	PhyTohormone-Auxin|Indole,Äê3,Äê Acetic Acid|IAA Metabolism	PhyTohormone-IAA Related TrypTophan Pathway	trpA->PGPT0007070	1
Direct Effects	PhyTohor mone|Pla ntSignal Productio n	PhyTohormone-Abscisic Acid Degradation	PhyTohormone-Auxin|Indole,Äê3,Äê Acetic Acid|IAA Metabolism	PhyTohormone-IAA Related TrypTophan Pathway	trpB->PGPT0007075	1
Direct Effects	PhyTohor mone|Pla ntSignal Productio n	PhyTohormone-Abscisic Acid Degradation	PhyTohormone-Auxin|Indole,Äê3,Äê Acetic Acid|IAA Metabolism	PhyTohormone-IAA Related TrypTophan Pathway	trpC->PGPT0007080	1
Direct Effects	PhyTohor mone|Pla ntSignal Productio n	PhyTohormone-Abscisic Acid Degradation	PhyTohormone-Auxin|Indole,Äê3,Äê Acetic Acid|IAA Metabolism	PhyTohormone-IAA Related TrypTophan Pathway	trpD->PGPT0007090	2
Direct Effects	PhyTohor mone|Pla ntSignal Productio	PhyTohormone-Abscisic Acid Degradation	PhyTohormone-Auxin|Indole,Äê3,Äê Acetic Acid|IAA Metabolism	PhyTohormone-IAA Related TrypTophan Pathway	trpE|phnA->PGPT0007095	3

	n					
Direct Effects	PhyTohormone|PlantSignal Production	PhyTohormone-Abscisic Acid Degradation	PhyTohormone-Auxin|Indole,Äê3,Äê Acetic Acid|IAA Metabolism	PhyTohormone-IAA Related TrypTophan Pathway	trpF->PGPT0007115	1
Direct Effects	PhyTohormone|PlantSignal Production	PhyTohormone-Abscisic Acid Degradation	PhyTohormone-Auxin|Indole,Äê3,Äê Acetic Acid|IAA Metabolism	PhyTohormone-IAA Related TrypTophan Pathway	trpR->PGPT0007130	1
Direct Effects	PhyTohormone|PlantSignal Production	PhyTohormone-Abscisic Acid Degradation	PhyTohormone-Auxin|Indole,Äê3,Äê Acetic Acid|IAA Metabolism	PhyTohormone-IAA Related TrypTophan Pathway	trpS->PGPT0007120	1
Direct Effects	PhyTohormone|PlantSignal Production	PhyTohormone-Abscisic Acid Degradation	PhyTohormone-Auxin|Indole,Äê3,Äê Acetic Acid|IAA Metabolism	PhyTohormone-IAA Related TrypTophan Pathway	yodT->PGPT0007136	2
Direct Effects	PhyTohormone|PlantSignal Production	PhyTohormone-Abscisic Acid Degradation	PhyTohormone-Auxin|Indole,Äê3,Äê Acetic Acid|IAA Metabolism	PhyTohormone-IAA Related TrypTophan Pathway	yugH->PGPT0007145	1
Direct Effects	PhyTohormone|PlantSignal Production	PhyTohormone-CyTokininS|Derivate Production	PhyTohormone-CyTokinin Metabolism	PhyTohormone-CyTokinin Biosynthesis	dapF->PGPT0007230	1
Direct	PhyTohor	PhyTohormone-	PhyTohormone-	PhyTohormone-	hflX->PGPT0007245	1

Effects	mone|PlantSignal Production	CyTokininS|Derivate Production	CyTokinin Metabolism	CyTokinin Biosynthesis		
Direct Effects	PhyTohormone|PlantSignal Production	PhyTohormone-CyTokininS|Derivate Production	PhyTohormone-CyTokinin Metabolism	PhyTohormone-CyTokinin Biosynthesis	log|yvdD->PGPT0007225	2
Direct Effects	PhyTohormone|PlantSignal Production	PhyTohormone-CyTokininS|Derivate Production	PhyTohormone-CyTokinin Metabolism	PhyTohormone-CyTokinin Biosynthesis	miaA|ipt->PGPT0007210	1
Direct Effects	PhyTohormone|PlantSignal Production	PhyTohormone-CyTokininS|Derivate Production	PhyTohormone-CyTokinin Metabolism	PhyTohormone-CyTokinin Biosynthesis	miaB->PGPT0007215	2
Direct Effects	PhyTohormone|PlantSignal Production	PhyTohormone-CyTokininS|Derivate Production	PhyTohormone-CyTokinin Metabolism	PhyTohormone-CyTokinin Biosynthesis	recA->PGPT0007235	3
Direct Effects	PhyTohormone|PlantSignal Production	PhyTohormone-CyTokininS|Derivate Production	PhyTohormone-CyTokinin Metabolism	PhyTohormone-CyTokinin Biosynthesis	recX->PGPT0007240	1
Direct Effects	PhyTohormone|PlantSignal	PhyTohormone-CyTokininS|Derivate Production	PhyTohormone-Xanthine Metabolism	PhyTohormone-Xanthine Biosynthesis	hprT|hpt->PGPT0007295	1

	Production					
Direct Effects	PhyTohormone|PlantSignal Production	PhyTohormone-CyTokininS|Derivate Production	PhyTohormone-Xanthine Metabolism	PhyTohormone-Xanthine Biosynthesis	ndmA->PGPT0007310	2
Direct Effects	PhyTohormone|PlantSignal Production	PhyTohormone-CyTokininS|Derivate Production	PhyTohormone-Xanthine Metabolism	PhyTohormone-Xanthine Biosynthesis	xdhB|pucD->PGPT0007265	1
Direct Effects	PhyTohormone|PlantSignal Production	PhyTohormone-CyTokininS|Derivate Production	PhyTohormone-Xanthine Metabolism	PhyTohormone-Xanthine Biosynthesis	xdhC|paod|ygeB|pucA->PGPT0007280	2
Direct Effects	PhyTohormone|PlantSignal Production	PhyTohormone-CyTokininS|Derivate Production	PhyTohormone-Xanthine Metabolism	PhyTohormone-Xanthine Biosynthesis	xpt->PGPT0007305	1
Direct Effects	PhyTohormone|PlantSignal Production	PhyTohormone-CyTokininS|Derivate Production	PhyTohormone-Xanthine Metabolism	PhyTohormone-Xanthine Biosynthesis	yagT->PGPT0007290	1
Direct Effects	PhyTohormone|PlantSignal Production	PhyTohormone-CyTokininS|Derivate Production	PhyTohormone-Xanthine Metabolism	PhyTohormone-Xanthine Biosynthesis	ygeT|xdhB->PGPT0007270	1

Direct Effects	PhyTohormone|PlantSignal Production	PhyTohormone-CyTokininS|Derivate Production	PhyTohormone-Xanthine Metabolism	PhyTohormone-Xanthine transport	pbuG|azgA|ghxP|ghxQ|adeQ->PGPT0007325	5
Direct Effects	PhyTohormone|PlantSignal Production	PhyTohormone-CyTokininS|Derivate Production	PhyTohormone-Xanthine Metabolism	PhyTohormone-Xanthine transport	pubX->PGPT0007330	4
Direct Effects	PhyTohormone|PlantSignal Production	PhyTohormone-Gamma-Aminobutyric Acid|GABA Production	PhyTohormone-GABA Metabolism	PhyTohormone-GABA Biosynthesis	aldH|dhaS->PGPT0006875	5
Direct Effects	PhyTohormone|PlantSignal Production	PhyTohormone-Gamma-Aminobutyric Acid|GABA Production	PhyTohormone-GABA Metabolism	PhyTohormone-GABA Biosynthesis	aOfH->PGPT0007641	1
Direct Effects	PhyTohormone|PlantSignal Production	PhyTohormone-Gamma-Aminobutyric Acid|GABA Production	PhyTohormone-GABA Metabolism	PhyTohormone-GABA Biosynthesis	gadA|gadB->PGPT0007630	1
Direct Effects	PhyTohormone|PlantSignal Production	PhyTohormone-Gamma-Aminobutyric Acid|GABA Production	PhyTohormone-GABA Metabolism	PhyTohormone-GABA Biosynthesis	patA1->PGPT0007155	1
Direct Effects	PhyTohormone|Pla	PhyTohormone-Gamma-Aminobutyric	PhyTohormone-GABA Metabolism	PhyTohormone-GABA Biosynthesis	puuB|ordL->PGPT0007637	1

	ntSignal Production	Acid|GABA Production				
Direct Effects	PhyTohormone|PlantSignal Production	PhyTohormone-Gamma-Aminobutyric Acid|GABA Production	PhyTohormone-GABA Metabolism	PhyTohormone-GABA Conversion	gabT->PGPT0007660	1
Direct Effects	PhyTohormone|PlantSignal Production	PhyTohormone-Gamma-Aminobutyric Acid|GABA Production	PhyTohormone-GABA Metabolism	PhyTohormone-GABA Conversion	puuE->PGPT0007140	3
Direct Effects	PhyTohormone|PlantSignal Production	PhyTohormone-Gamma-Aminobutyric Acid|GABA Production	PhyTohormone-GABA Metabolism	PhyTohormone-GABA Degradation	gabD->PGPT0001580	5
Direct Effects	PhyTohormone|PlantSignal Production	PhyTohormone-Gamma-Aminobutyric Acid|GABA Production	PhyTohormone-GABA Metabolism	PhyTohormone-GABA transport	gabP->PGPT0007665	4
Direct Effects	PhyTohormone|PlantSignal Production	PhyTohormone-Gibberellins Production	PhyTohormone-Gibberellins Metabolism	PhyTohormone-Gibberellins Biosynthesis	Uncharacterized P450 System 3Fe 4S Ferredoxin->PGPT0007360	4
Direct Effects	PhyTohormone|PlantSignal Productio	Plant Signal-Branching Inhibition	Plant Debranching-Spermidine|Putrescine Metabolism	Plant Debranching-N1-|N8-AcetylSpermidine Formation	paiA->PGPT0007785	10

	n					
Direct Effects	PhyTohormone|PlantSignal Production	Plant Signal-Branching Inhibition	Plant Debranching-Spermidine|Putrescine Metabolism	Plant Debranching-N1-|N8-AcetylSpermidine Formation	speG->PGPT0007790	6
Direct Effects	PhyTohormone|PlantSignal Production	Plant Signal-Branching Inhibition	Plant Debranching-Spermidine|Putrescine Metabolism	Plant Debranching-Putrescine Biosynthesis	speB->PGPT0007775	3
Direct Effects	PhyTohormone|PlantSignal Production	Plant Signal-Branching Inhibition	Plant Debranching-Spermidine|Putrescine Metabolism	Plant Debranching-Putrescine Degradation	aldH|dhaS->PGPT0006875	5
Direct Effects	PhyTohormone|PlantSignal Production	Plant Signal-Branching Inhibition	Plant Debranching-Spermidine|Putrescine Metabolism	Plant Debranching-Putrescine Degradation	aOfH->PGPT0007641	1
Direct Effects	PhyTohormone|PlantSignal Production	Plant Signal-Branching Inhibition	Plant Debranching-Spermidine|Putrescine Metabolism	Plant Debranching-Putrescine Degradation	patA1->PGPT0007155	1
Direct Effects	PhyTohormone|PlantSignal Production	Plant Signal-Branching Inhibition	Plant Debranching-Spermidine|Putrescine Metabolism	Plant Debranching-Putrescine Degradation	puuB|ordL->PGPT0007637	1
Direct	PhyTohor	Plant Signal-Branching	Plant Debranching-	Plant Debranching-	puuR->PGPT0019781	3

Effects	mone|PlantSignal Production	Inhibition	Spermidine|Putrescine Metabolism	Putrescine Degradation		
Direct Effects	PhyTohormone|PlantSignal Production	Plant Signal-Branching Inhibition	Plant Debranching-Spermidine|Putrescine Metabolism	Plant Debranching-Putrescine transport	puuP->PGPT0007795	4
Direct Effects	PhyTohormone|PlantSignal Production	Plant Signal-Branching Inhibition	Plant Debranching-Spermidine|Putrescine Metabolism	Plant Debranching-Spermidine Biosynthesis	gsp->PGPT0007755	1
Direct Effects	PhyTohormone|PlantSignal Production	Plant Signal-Branching Inhibition	Plant Debranching-Spermidine|Putrescine Metabolism	Plant Debranching-Spermidine Biosynthesis	speE|SRM|SPEC3|SPSD->PGPT0007750	3
Direct Effects	PhyTohormone|PlantSignal Production	Plant Signal-Branching Inhibition	Plant Debranching-Spermidine|Putrescine Metabolism	Plant Debranching-Spermidine|Putrescine transport	potA->PGPT0007840	1
Direct Effects	PhyTohormone|PlantSignal Production	Plant Signal-Branching Inhibition	Plant Debranching-Spermidine|Putrescine Metabolism	Plant Debranching-Spermidine|Putrescine transport	potB->PGPT0007835	1
Direct Effects	PhyTohormone|PlantSignal	Plant Signal-Branching Inhibition	Plant Debranching-Spermidine|Putrescine Metabolism	Plant Debranching-Spermidine|Putrescine transport	potC->PGPT0007830	1

	Productio n					
Direct Effects	PhyTohor mone|Pla ntSignal Productio n	Plant Signal-Branching Inhibition	Plant Debranching-Spermidine|Putrescin e Metabolism	Plant Debranching-Spermidine|Putrescin e transport	potD->PGPT0007825	1
Direct Effects	PhyTohor mone|Pla ntSignal Productio n	Plant Signal-Branching Stimulation	Plant Branching-Auxin|IAA Metabolism	Plant Branching-IAA Metabolism	EC 3 5 1 4|amiE->PGPT0006115	1
Direct Effects	PhyTohor mone|Pla ntSignal Productio n	Plant Signal-Branching Stimulation	Plant Branching-Auxin|IAA Metabolism	Plant Branching-IAA Metabolism	iaaT|yedL|ysnE->PGPT0007180	1
Direct Effects	PhyTohor mone|Pla ntSignal Productio n	Plant Signal-Branching Stimulation	Plant Branching-Auxin|IAA Metabolism	Plant Branching-IAA Metabolism	yhcX->PGPT0007175	2
Direct Effects	PhyTohor mone|Pla ntSignal Productio n	Plant Signal-Branching Stimulation	Plant Branching-Auxin|IAA Metabolism	Plant Branching-IAA Related Indole-3-Acetaldehyde Metabolism	aldH|dhaS->PGPT0006875	5
Direct Effects	PhyTohor mone|Pla ntSignal Productio n	Plant Signal-Branching Stimulation	Plant Branching-Auxin|IAA Metabolism	Plant Branching-IAA Related Indole-3-Acetaldehyde Metabolism	betB Homologous->PGPT0007165	4

Direct Effects	PhyTohormone|PlantSignal Production	Plant Signal-Branching Stimulation	Plant Branching-Auxin|IAA Metabolism	Plant Branching-IAA Related Indole-3-Acetaldehyde Metabolism	bsdC->PGPT0005435	1
Direct Effects	PhyTohormone|PlantSignal Production	Plant Signal-Branching Stimulation	Plant Branching-Auxin|IAA Metabolism	Plant Branching-IAA Related Indole-3-Acetaldehyde Metabolism	poxL->PGPT0007160	2
Direct Effects	PhyTohormone|PlantSignal Production	Plant Signal-Branching Stimulation	Plant Branching-Auxin|IAA Metabolism	Plant Branching-IAA Related Indole-3-Acetaldehyde Metabolism	pyc->PGPT0001420	1
Direct Effects	PhyTohormone|PlantSignal Production	Plant Signal-Branching Stimulation	Plant Branching-Auxin|IAA Metabolism	Plant Branching-IAA Related TRP-Dependent Pathway 2	EC 3 5 1 4|amiE->PGPT0006115	1
Direct Effects	PhyTohormone|PlantSignal Production	Plant Signal-Branching Stimulation	Plant Branching-Auxin|IAA Metabolism	Plant Branching-IAA Related TrypTophan Metabolism	lysN->PGPT0007135	3
Direct Effects	PhyTohormone|PlantSignal Production	Plant Signal-Branching Stimulation	Plant Branching-Auxin|IAA Metabolism	Plant Branching-IAA Related TrypTophan Metabolism	patA->PGPT0007150	1
Direct Effects	PhyTohormone|Pla	Plant Signal-Branching Stimulation	Plant Branching-Auxin|IAA	Plant Branching-IAA Related TrypTophan	patA1->PGPT0007155	1

	ntSignal Production		Metabolism	Metabolism		
Direct Effects	PhyTohormone|PlantSignal Production	Plant Signal-Branching Stimulation	Plant Branching-Auxin|IAA Metabolism	Plant Branching-IAA Related TrypTophan Metabolism	puuE->PGPT0007140	3
Direct Effects	PhyTohormone|PlantSignal Production	Plant Signal-Branching Stimulation	Plant Branching-Auxin|IAA Metabolism	Plant Branching-IAA Related TrypTophan Metabolism	tpav|bioA|yhxA->PGPT0001925	2
Direct Effects	PhyTohormone|PlantSignal Production	Plant Signal-Branching Stimulation	Plant Branching-Auxin|IAA Metabolism	Plant Branching-IAA Related TrypTophan Metabolism	trpA->PGPT0007070	1
Direct Effects	PhyTohormone|PlantSignal Production	Plant Signal-Branching Stimulation	Plant Branching-Auxin|IAA Metabolism	Plant Branching-IAA Related TrypTophan Metabolism	trpB->PGPT0007075	1
Direct Effects	PhyTohormone|PlantSignal Production	Plant Signal-Branching Stimulation	Plant Branching-Auxin|IAA Metabolism	Plant Branching-IAA Related TrypTophan Metabolism	trpC->PGPT0007080	1
Direct Effects	PhyTohormone|PlantSignal Productio	Plant Signal-Branching Stimulation	Plant Branching-Auxin|IAA Metabolism	Plant Branching-IAA Related TrypTophan Metabolism	trpD->PGPT0007090	2

	n					
Direct Effects	PhyTohormone|PlantSignal Production	Plant Signal-Branching Stimulation	Plant Branching-Auxin|IAA Metabolism	Plant Branching-IAA Related TrypTophan Metabolism	trpE|phnA->PGPT0007095	3
Direct Effects	PhyTohormone|PlantSignal Production	Plant Signal-Branching Stimulation	Plant Branching-Auxin|IAA Metabolism	Plant Branching-IAA Related TrypTophan Metabolism	trpF->PGPT0007115	1
Direct Effects	PhyTohormone|PlantSignal Production	Plant Signal-Branching Stimulation	Plant Branching-Auxin|IAA Metabolism	Plant Branching-IAA Related TrypTophan Metabolism	trpR->PGPT0007130	1
Direct Effects	PhyTohormone|PlantSignal Production	Plant Signal-Branching Stimulation	Plant Branching-Auxin|IAA Metabolism	Plant Branching-IAA Related TrypTophan Metabolism	trpS->PGPT0007120	1
Direct Effects	PhyTohormone|PlantSignal Production	Plant Signal-Branching Stimulation	Plant Branching-Auxin|IAA Metabolism	Plant Branching-IAA Related TrypTophan Metabolism	yugH->PGPT0007145	1
Direct Effects	PhyTohormone|PlantSignal Production	Plant Signal-EmbryoGenesis	Plant EmbryoGenesis-Spermidine|Putrescine Metabolism	Plant EmbryoGenesis-N1-|N8-AcetylSpermidine Biosynthesis	paiA->PGPT0007785	10
Direct	PhyTohor	Plant Signal-	Plant EmbryoGenesis-	Plant EmbryoGenesis-	speG->PGPT0007790	6

Effects	mone|PlantSignal Production	EmbryoGenesis	Spermidine|Putrescine Metabolism	N1-|N8-AcetylSpermidine Biosynthesis		
Direct Effects	PhyTohormone|PlantSignal Production	Plant Signal-EmbryoGenesis	Plant EmbryoGenesis-Spermidine|Putrescine Metabolism	Plant EmbryoGenesis-Putrescine Biosynthesis	speB->PGPT0007775	3
Direct Effects	PhyTohormone|PlantSignal Production	Plant Signal-EmbryoGenesis	Plant EmbryoGenesis-Spermidine|Putrescine Metabolism	Plant EmbryoGenesis-Putrescine Degradation	aldH|dhaS->PGPT0006875	5
Direct Effects	PhyTohormone|PlantSignal Production	Plant Signal-EmbryoGenesis	Plant EmbryoGenesis-Spermidine|Putrescine Metabolism	Plant EmbryoGenesis-Putrescine Degradation	aOfH->PGPT0007641	1
Direct Effects	PhyTohormone|PlantSignal Production	Plant Signal-EmbryoGenesis	Plant EmbryoGenesis-Spermidine|Putrescine Metabolism	Plant EmbryoGenesis-Putrescine Degradation	patA1->PGPT0007155	1
Direct Effects	PhyTohormone|PlantSignal Production	Plant Signal-EmbryoGenesis	Plant EmbryoGenesis-Spermidine|Putrescine Metabolism	Plant EmbryoGenesis-Putrescine Degradation	puuB|ordL->PGPT0007637	1
Direct Effects	PhyTohormone|PlantSignal	Plant Signal-EmbryoGenesis	Plant EmbryoGenesis-Spermidine|Putrescine Metabolism	Plant EmbryoGenesis-Putrescine Degradation	puuR->PGPT0019781	3

	Production					
Direct Effects	PhyTohormone|PlantSignal Production	Plant Signal-EmbryoGenesis	Plant EmbryoGenesis-Spermidine|Putrescine Metabolism	Plant EmbryoGenesis-Putrescine transport	puuP->PGPT0007795	4
Direct Effects	PhyTohormone|PlantSignal Production	Plant Signal-EmbryoGenesis	Plant EmbryoGenesis-Spermidine|Putrescine Metabolism	Plant EmbryoGenesis-Spermidine Biosynthesis	gsp->PGPT0007755	1
Direct Effects	PhyTohormone|PlantSignal Production	Plant Signal-EmbryoGenesis	Plant EmbryoGenesis-Spermidine|Putrescine Metabolism	Plant EmbryoGenesis-Spermidine Biosynthesis	speE|SRM|SPEC3|SPSD->PGPT0007750	3
Direct Effects	PhyTohormone|PlantSignal Production	Plant Signal-EmbryoGenesis	Plant EmbryoGenesis-Spermidine|Putrescine Metabolism	Plant EmbryoGenesis-Spermidine|Putrescine transport	potA->PGPT0007840	1
Direct Effects	PhyTohormone|PlantSignal Production	Plant Signal-EmbryoGenesis	Plant EmbryoGenesis-Spermidine|Putrescine Metabolism	Plant EmbryoGenesis-Spermidine|Putrescine transport	potB->PGPT0007835	1
Direct Effects	PhyTohormone|PlantSignal Production	Plant Signal-EmbryoGenesis	Plant EmbryoGenesis-Spermidine|Putrescine Metabolism	Plant EmbryoGenesis-Spermidine|Putrescine transport	potC->PGPT0007830	1

Direct Effects	PhyTohormone|PlantSignal Production	Plant Signal-EmbryoGenesis	Plant EmbryoGenesis-Spermidine|Putrescine Metabolism	Plant EmbryoGenesis-Spermidine|Putrescine transport	potD->PGPT0007825	1
Direct Effects	PhyTohormone|PlantSignal Production	Plant Signal-Germination Stimulation	Plant Germination-H2S Production	Plant Germination-H2S-Sulfur Pathway	cysA->PGPT0002990	1
Direct Effects	PhyTohormone|PlantSignal Production	Plant Signal-Germination Stimulation	Plant Germination-H2S Production	Plant Germination-H2S-Sulfur Pathway	cysC->PGPT0002780	2
Direct Effects	PhyTohormone|PlantSignal Production	Plant Signal-Germination Stimulation	Plant Germination-H2S Production	Plant Germination-H2S-Sulfur Pathway	cysE->PGPT0002970	1
Direct Effects	PhyTohormone|PlantSignal Production	Plant Signal-Germination Stimulation	Plant Germination-H2S Production	Plant Germination-H2S-Sulfur Pathway	cysH->PGPT0002785	1
Direct Effects	PhyTohormone|PlantSignal Production	Plant Signal-Germination Stimulation	Plant Germination-H2S Production	Plant Germination-H2S-Sulfur Pathway	cysI->PGPT0002790	1
Direct Effects	PhyTohormone|Pla	Plant Signal-Germination	Plant Germination-H2S Production	Plant Germination-H2S-Sulfur Pathway	cysJ->PGPT0002795	1

	ntSignal Productio n	Stimulation				
Direct Effects	PhyTohor mone|Pla ntSignal Productio n	Plant Signal-Germination Stimulation	Plant Germination-H2S Production	Plant Germination-H2S-Sulfur Pathway	cysK->PGPT0002810	3
Direct Effects	PhyTohor mone|Pla ntSignal Productio n	Plant Signal-Germination Stimulation	Plant Germination-H2S Production	Plant Germination-H2S-Sulfur Pathway	cysK2->PGPT0002815	1
Direct Effects	PhyTohor mone|Pla ntSignal Productio n	Plant Signal-Germination Stimulation	Plant Germination-H2S Production	Plant Germination-H2S-Sulfur Pathway	cysS->PGPT0002985	1
Direct Effects	PhyTohor mone|Pla ntSignal Productio n	Plant Signal-Germination Stimulation	Plant Germination-H2S Production	Plant Germination-H2S-Sulfur Pathway	cysT|cysU->PGPT0003000	1
Direct Effects	PhyTohor mone|Pla ntSignal Productio n	Plant Signal-Germination Stimulation	Plant Germination-H2S Production	Plant Germination-H2S-Sulfur Pathway	cysW->PGPT0003005	1
Direct Effects	PhyTohor mone|Pla ntSignal Productio	Plant Signal-Germination Stimulation	Plant Germination-H2S Production	Plant Germination-H2S-Sulfur Pathway	sat|met3->PGPT0002410	1

	n					
Direct Effects	PhyTohormone|PlantSignal Production	Plant Signal-Germination Stimulation	Plant Germination-Lipoic Acid Production	Plant Germination-Lipoic Acid Biosynthesis	lipA->PGPT0003935	1
Direct Effects	PhyTohormone|PlantSignal Production	Plant Signal-Germination Stimulation	Plant Germination-Lipoic Acid Production	Plant Germination-Lipoic Acid Biosynthesis	lipL->PGPT0003940	1
Direct Effects	PhyTohormone|PlantSignal Production	Plant Signal-Germination Stimulation	Plant Germination-Taurine Derivate Production	Plant Germination-5-Glutamyl-Taurine Biosynthesis	ggt->PGPT0002935	6
Direct Effects	PhyTohormone|PlantSignal Production	Plant Signal-Germination Stimulation	Plant Germination-Taurine Derivate Production	Plant Germination-Taurine Derivate Biosynthesis	gadA|gadB->PGPT0007630	1
Direct Effects	PhyTohormone|PlantSignal Production	Plant Signal-Germination Stimulation	Plant Germination-Taurine Derivate Production	Plant Germination-Taurine Derivate Biosynthesis	tpav|bioA|yhxA->PGPT0001925	2
Direct Effects	PhyTohormone|PlantSignal Production	Plant Signal-Germination Stimulation	Plant Germination-Vitamin B9|Folate Production	Plant Germination-Vitamin B9|Folate Biosynthesis	fhs->PGPT0008065	1
Direct	PhyTohor	Plant Signal-	Plant Germination-	Plant Germination-	fmt->PGPT0008125	1

Effects	mone|PlantSignal Production	Germination Stimulation	Vitamin B9|Folate Production	Vitamin B9|Folate Biosynthesis		
Direct Effects	PhyTohormone|PlantSignal Production	Plant Signal-Germination Stimulation	Plant Germination-Vitamin B9|Folate Production	Plant Germination-Vitamin B9|Folate Biosynthesis	folA->PGPT0007945	1
Direct Effects	PhyTohormone|PlantSignal Production	Plant Signal-Germination Stimulation	Plant Germination-Vitamin B9|Folate Production	Plant Germination-Vitamin B9|Folate Biosynthesis	folB->PGPT0007905	1
Direct Effects	PhyTohormone|PlantSignal Production	Plant Signal-Germination Stimulation	Plant Germination-Vitamin B9|Folate Production	Plant Germination-Vitamin B9|Folate Biosynthesis	folC->PGPT0007975	1
Direct Effects	PhyTohormone|PlantSignal Production	Plant Signal-Germination Stimulation	Plant Germination-Vitamin B9|Folate Production	Plant Germination-Vitamin B9|Folate Biosynthesis	folD->PGPT0008075	1
Direct Effects	PhyTohormone|PlantSignal Production	Plant Signal-Germination Stimulation	Plant Germination-Vitamin B9|Folate Production	Plant Germination-Vitamin B9|Folate Biosynthesis	folE->PGPT0007875	2
Direct Effects	PhyTohormone|PlantSignal	Plant Signal-Germination Stimulation	Plant Germination-Vitamin B9|Folate Production	Plant Germination-Vitamin B9|Folate Biosynthesis	folE2->PGPT0007880	2

	Production					
Direct Effects	PhyTohormone|PlantSignal Production	Plant Signal-Germination Stimulation	Plant Germination-Vitamin B9|Folate Production	Plant Germination-Vitamin B9|Folate Biosynthesis	folK->PGPT0007910	1
Direct Effects	PhyTohormone|PlantSignal Production	Plant Signal-Germination Stimulation	Plant Germination-Vitamin B9|Folate Production	Plant Germination-Vitamin B9|Folate Biosynthesis	folP->PGPT0007915	1
Direct Effects	PhyTohormone|PlantSignal Production	Plant Signal-Germination Stimulation	Plant Germination-Vitamin B9|Folate Production	Plant Germination-Vitamin B9|Folate Biosynthesis	gcvT->PGPT0008130	1
Direct Effects	PhyTohormone|PlantSignal Production	Plant Signal-Germination Stimulation	Plant Germination-Vitamin B9|Folate Production	Plant Germination-Vitamin B9|Folate Biosynthesis	glyA->PGPT0008090	1
Direct Effects	PhyTohormone|PlantSignal Production	Plant Signal-Germination Stimulation	Plant Germination-Vitamin B9|Folate Production	Plant Germination-Vitamin B9|Folate Biosynthesis	metH->PGPT0008135	2
Direct Effects	PhyTohormone|PlantSignal Production	Plant Signal-Germination Stimulation	Plant Germination-Vitamin B9|Folate Production	Plant Germination-Vitamin B9|Folate Biosynthesis	pabA->PGPT0008000	1

Direct Effects	PhyTohormone|PlantSignal Production	Plant Signal-Germination Stimulation	Plant Germination-Vitamin B9|Folate Production	Plant Germination-Vitamin B9|Folate Biosynthesis	pabB->PGPT0008005	1
Direct Effects	PhyTohormone|PlantSignal Production	Plant Signal-Germination Stimulation	Plant Germination-Vitamin B9|Folate Production	Plant Germination-Vitamin B9|Folate Biosynthesis	pabC->PGPT0008020	1
Direct Effects	PhyTohormone|PlantSignal Production	Plant Signal-Germination Stimulation	Plant Germination-Vitamin B9|Folate Production	Plant Germination-Vitamin B9|Folate Biosynthesis	phoA->PGPT0002570	2
Direct Effects	PhyTohormone|PlantSignal Production	Plant Signal-Germination Stimulation	Plant Germination-Vitamin B9|Folate Production	Plant Germination-Vitamin B9|Folate Biosynthesis	phoD->PGPT0002575	1
Direct Effects	PhyTohormone|PlantSignal Production	Plant Signal-Germination Stimulation	Plant Germination-Vitamin B9|Folate Production	Plant Germination-Vitamin B9|Folate Biosynthesis	purH->PGPT0008110	2
Direct Effects	PhyTohormone|PlantSignal Production	Plant Signal-Germination Stimulation	Plant Germination-Vitamin B9|Folate Production	Plant Germination-Vitamin B9|Folate Biosynthesis	purN->PGPT0008095	1
Direct Effects	PhyTohormone|Pla	Plant Signal-Germination	Plant Germination-Vitamin B9|Folate	Plant Germination-Vitamin B9|Folate	purU->PGPT0008155	1

	ntSignal Productio n	Stimulation	Production	Biosynthesis		
Direct Effects	PhyTohor mone|Pla ntSignal Productio n	Plant Signal-Germination Stimulation	Plant Germination-Vitamin B9|Folate Production	Plant Germination-Vitamin B9|Folate Biosynthesis	ribA->PGPT0007985	1
Direct Effects	PhyTohor mone|Pla ntSignal Productio n	Plant Signal-Germination Stimulation	Plant Germination-Vitamin B9|Folate Production	Plant Germination-Vitamin B9|Folate Biosynthesis	ribBA->PGPT0007990	1
Direct Effects	PhyTohor mone|Pla ntSignal Productio n	Plant Signal-Germination Stimulation	Plant Germination-Vitamin B9|Folate Production	Plant Germination-Vitamin B9|Folate Biosynthesis	thyA->PGPT0008145	1
Direct Effects	PhyTohor mone|Pla ntSignal Productio n	Plant Signal-Germination Stimulation	Plant Germination-Vitamin B9|Folate Production	Plant Germination-Vitamin B9|Folate Biosynthesis	ygfA|fthC|yqgN|folN ->PGPT0008170	1
Direct Effects	PhyTohor mone|Pla ntSignal Productio n	Plant Signal-Germination Stimulation	Plant Germination-Vitamin B9|Folate Production	Plant Germination-Vitamin B9|Folate Biosynthesis	yitJ->PGPT0008140	1
Direct Effects	PhyTohor mone|Pla ntSignal Productio	Plant Signal-Other Terpenoid|Derivate Production	Plant Signal-Carotenoid Metabolism	Plant Signal-Carotenoid Biosynthesis	crtE|ispA->PGPT0007560	1

	n					
Direct Effects	PhyTohormone|PlantSignal Production	Plant Signal-Other Terpenoid|Derivate Production	Plant Signal-Carotenoid Metabolism	Plant Signal-Carotenoid Biosynthesis	crtH|crtISO->PGPT0007400	1
Direct Effects	PhyTohormone|PlantSignal Production	Plant Signal-Other Terpenoid|Derivate Production	Plant Signal-Carotenoid Metabolism	Plant Signal-Carotenoid Biosynthesis	crtI->PGPT0007405	1
Direct Effects	PhyTohormone|PlantSignal Production	Plant Signal-Other Terpenoid|Derivate Production	Plant Signal-Carotenoid Metabolism	Plant Signal-Carotenoid Biosynthesis	crtM->PGPT0007420	2
Direct Effects	PhyTohormone|PlantSignal Production	Plant Signal-Other Terpenoid|Derivate Production	Plant Signal-Carotenoid Metabolism	Plant Signal-Carotenoid Biosynthesis	crtN->PGPT0007425	1
Direct Effects	PhyTohormone|PlantSignal Production	Plant Signal-Other Terpenoid|Derivate Production	Plant Signal-Carotenoid Metabolism	Plant Signal-Carotenoid Biosynthesis	crTo->PGPT0007435	1
Direct Effects	PhyTohormone|PlantSignal Production	Plant Signal-Other Terpenoid|Derivate Production	Plant Signal-Carotenoid Metabolism	Plant Signal-Carotenoid Biosynthesis	crtP->PGPT0007440	2
Direct	PhyTohor	Plant Signal-Other	Plant Signal-	Plant Signal-	crtQ->PGPT0007450	1

Effects	mone|PlantSignal Production	Terpenoid|Derivate Production	Carotenoid Metabolism	Carotenoid Biosynthesis		
Direct Effects	PhyTohormone|PlantSignal Production	Plant Signal-Other Terpenoid|Derivate Production	Plant Signal-Carotenoid Metabolism	Plant Signal-Carotenoid Biosynthesis	cruC->PGPT0007495	1
Direct Effects	PhyTohormone|PlantSignal Production	Plant Signal-Other Terpenoid|Derivate Production	Plant Signal-Carotenoid Metabolism	Plant Signal-Carotenoid Biosynthesis	cruD->PGPT0007500	1
Direct Effects	PhyTohormone|PlantSignal Production	Plant Signal-Other Terpenoid|Derivate Production	Plant Signal-Terpenoid Derivate Metabolism	Plant Signal-Terpenoid-DiMethylallyl Diphosphate|DMAPP Pathway	ispH|lytB->PGPT0007615	2
Direct Effects	PhyTohormone|PlantSignal Production	Plant Signal-Other Terpenoid|Derivate Production	Plant Signal-Terpenoid Derivate Metabolism	Plant Signal-Terpenoid-IsoPentenyl Diphosphate|IPP Pathway	gcpE|ispG->PGPT0007580	1
Direct Effects	PhyTohormone|PlantSignal Production	Plant Signal-Other Terpenoid|Derivate Production	Plant Signal-Terpenoid Derivate Metabolism	Plant Signal-Terpenoid-IsoPentenyl Diphosphate|IPP Pathway	idi->PGPT0007530	2
Direct Effects	PhyTohormone|PlantSignal	Plant Signal-Other Terpenoid|Derivate Production	Plant Signal-Terpenoid Derivate Metabolism	Plant Signal-Terpenoid-IsoPentenyl Diphosphate|IPP	ispD->PGPT0007590	1

	Productio n			Pathway		
Direct Effects	PhyTohor mone|Pla ntSignal Productio n	Plant Signal-Other Terpenoid|Derivate Production	Plant Signal-Terpenoid Derivate Metabolism	Plant Signal-Terpenoid-IsoPentenyl Diphosphate|IPP Pathway	ispE->PGPT0007605	1
Direct Effects	PhyTohor mone|Pla ntSignal Productio n	Plant Signal-Other Terpenoid|Derivate Production	Plant Signal-Terpenoid Derivate Metabolism	Plant Signal-Terpenoid-IsoPentenyl Diphosphate|IPP Pathway	ispF->PGPT0007595	2
Direct Effects	PhyTohor mone|Pla ntSignal Productio n	Plant Signal-Other Terpenoid|Derivate Production	Plant Signal-Terpenoid Derivate Metabolism	Plant Signal-Terpenoid-IsoPentenyl Diphosphate|IPP Pathway	ispH|lytB->PGPT0007615	2
Direct Effects	PhyTohor mone|Pla ntSignal Productio n	Plant Signal-Other Terpenoid|Derivate Production	Plant Signal-Terpenoid Derivate Metabolism	Plant Signal-Terpenoid-Sporulenol Biosynthesis	sphC->PGPT0007620	1
Direct Effects	PhyTohor mone|Pla ntSignal Productio n	Plant Signal-Other Terpenoid|Derivate Production	Plant Signal-Terpenoid Derivate Metabolism	Plant Signal-Terpenoid Backbone Biosynthesis	crtE|ispA->PGPT0007560	1
Direct Effects	PhyTohor mone|Pla ntSignal Productio n	Plant Signal-Other Terpenoid|Derivate Production	Plant Signal-Terpenoid Derivate Metabolism	Plant Signal-Terpenoid Backbone Biosynthesis	dxs->PGPT0008960	1

Direct Effects	PhyTohormone|PlantSignal Production	Plant Signal-Other Terpenoid|Derivate Production	Plant Signal-Terpenoid Derivate Metabolism	Plant Signal-Terpenoid Backbone Biosynthesis	hepST->PGPT0007531	1
Direct Effects	PhyTohormone|PlantSignal Production	Plant Signal-Other Terpenoid|Derivate Production	Plant Signal-Terpenoid Derivate Metabolism	Plant Signal-Terpenoid Backbone Biosynthesis	idi->PGPT0007530	1
Direct Effects	PhyTohormone|PlantSignal Production	Plant Signal-PhosphoLipid Production	Plant Signal-PhosphoLipid Metabolism	Plant Signal-Cyclopropane-Fatty-acyl-PhosphoLipid Biosynthesis	cfa->PGPT0007680	1
Direct Effects	PhyTohormone|PlantSignal Production	Plant Signal-PhosphoLipid Production	Plant Signal-PhosphoLipid Metabolism	Plant Signal-Cytidine Diphosphate-Diacylglicerol-PhosphoLipid Biosynthesis	cdsA|ynbB->PGPT0007685	1
Direct Effects	PhyTohormone|PlantSignal Production	Plant Signal-PhosphoLipid Production	Plant Signal-PhosphoLipid Metabolism	Plant Signal-PhosphoLipid-Cardiolipin Biosynthesis	clsA B|ybhO|ywiE->PGPT0007725	5
Direct Effects	PhyTohormone|PlantSignal Production	Plant Signal-PhosphoLipid Production	Plant Signal-PhosphoLipid Metabolism	Plant Signal-PhosphoLipid-Cardiolipin Biosynthesis	clsC|ymdC->PGPT0007730	2
Direct Effects	PhyTohormone|Pla	Plant Signal-PhosphoLipid	Plant Signal-PhosphoLipid	Plant Signal-PhosphoLipid-	pmtA->PGPT0007740	3

	ntSignal Production	Production	Metabolism	Phosphatidylcholine Biosynthesis		
Direct Effects	PhyTohormone|PlantSignal Production	Plant Signal-PhosphoLipid Production	Plant Signal-PhosphoLipid Metabolism	Plant Signal-PhosphoLipid-PhosphatidylethanolAmine Biosynthesis	psd|PISD->PGPT0007700	1
Direct Effects	PhyTohormone|PlantSignal Production	Plant Signal-PhosphoLipid Production	Plant Signal-PhosphoLipid Metabolism	Plant Signal-PhosphoLipid-PhosphatidylGlycerol Biosynthesis	pgpA->PGPT0007710	2
Direct Effects	PhyTohormone|PlantSignal Production	Plant Signal-PhosphoLipid Production	Plant Signal-PhosphoLipid Metabolism	Plant Signal-PhosphoLipid-PhosphatidylSerine Biosynthesis	CHO1|pssA->PGPT0007695	3
Direct Effects	PhyTohormone|PlantSignal Production	Plant Signal-Ubiquinone|Coenzyme Q Production	Plant Signal-Ubiquinone|Coenzyme Q Metabolism	Plant Signal-Ubiquinone|Coenzyme Q Biosynthesis	ubiE->PGPT0009535	1
Direct Effects	PhyTohormone|PlantSignal Production	Plant Signal-Ubiquinone|Coenzyme Q Production	Plant Signal-Ubiquinone|Coenzyme Q Metabolism	Plant Signal-Ubiquinone|Coenzyme Q Biosynthesis	ubiX|bsdB->PGPT0009565	1
Direct Effects	PhyTohormone|PlantSignal Productio	Plant Signaling Volatiles	Plant Signal-Acetoin|2|3-Butanediol Volatile Metabolism	Plant Signal-Acetoin|2|3-Butanediol Volatile Biosynthesis	aceF|pdhC->PGPT0001390	2

	n					
Direct Effects	PhyTohormone|PlantSignal Production	Plant Signaling Volatiles	Plant Signal-Acetoin|2|3-Butanediol Volatile Metabolism	Plant Signal-Acetoin|2|3-Butanediol Volatile Biosynthesis	acoA->PGPT0008230	1
Direct Effects	PhyTohormone|PlantSignal Production	Plant Signaling Volatiles	Plant Signal-Acetoin|2|3-Butanediol Volatile Metabolism	Plant Signal-Acetoin|2|3-Butanediol Volatile Biosynthesis	acoB->PGPT0008235	1
Direct Effects	PhyTohormone|PlantSignal Production	Plant Signaling Volatiles	Plant Signal-Acetoin|2|3-Butanediol Volatile Metabolism	Plant Signal-Acetoin|2|3-Butanediol Volatile Biosynthesis	acoR->PGPT0001030	6
Direct Effects	PhyTohormone|PlantSignal Production	Plant Signaling Volatiles	Plant Signal-Acetoin|2|3-Butanediol Volatile Metabolism	Plant Signal-Acetoin|2|3-Butanediol Volatile Biosynthesis	acuB->PGPT0008220	4
Direct Effects	PhyTohormone|PlantSignal Production	Plant Signaling Volatiles	Plant Signal-Acetoin|2|3-Butanediol Volatile Metabolism	Plant Signal-Acetoin|2|3-Butanediol Volatile Biosynthesis	acuC->PGPT0008225	1
Direct Effects	PhyTohormone|PlantSignal Production	Plant Signaling Volatiles	Plant Signal-Acetoin|2|3-Butanediol Volatile Metabolism	Plant Signal-Acetoin|2|3-Butanediol Volatile Biosynthesis	budA|aldC|aldB|alsD->PGPT0008180	1
Direct	PhyTohor	Plant Signaling	Plant Signal-	Plant Signal-	budB|ilvK|alsS|ilvB|i	4

Effects	mone|PlantSignal Production	Volatiles	Acetoin|2|3-Butanediol Volatile Metabolism	Acetoin|2|3-Butanediol Volatile Biosynthesis	lvG|ilvI->PGPT0008185	
Direct Effects	PhyTohormone|PlantSignal Production	Plant Signaling Volatiles	Plant Signal-Acetoin|2|3-Butanediol Volatile Metabolism	Plant Signal-Acetoin|2|3-Butanediol Volatile Biosynthesis	budC->PGPT0008190	3
Direct Effects	PhyTohormone|PlantSignal Production	Plant Signaling Volatiles	Plant Signal-Acetoin|2|3-Butanediol Volatile Metabolism	Plant Signal-Acetoin|2|3-Butanediol Volatile Biosynthesis	butA|ydjL|budC->PGPT0008195	1
Direct Effects	PhyTohormone|PlantSignal Production	Plant Signaling Volatiles	Plant Signal-Acetoin|2|3-Butanediol Volatile Metabolism	Plant Signal-Acetoin|2|3-Butanediol Volatile Biosynthesis	butB->PGPT0008200	1
Direct Effects	PhyTohormone|PlantSignal Production	Plant Signaling Volatiles	Plant Signal-Acetoin|2|3-Butanediol Volatile Metabolism	Plant Signal-Acetoin|2|3-Butanediol Volatile Biosynthesis	ilvH|ilvN->PGPT0008205	1
Direct Effects	PhyTohormone|PlantSignal Production	Plant Signaling Volatiles	Plant Signal-Acetoin|2|3-Butanediol Volatile Metabolism	Plant Signal-Acetoin|2|3-Butanediol Volatile Biosynthesis	lpd|pdhD->PGPT0001380	3
Direct Effects	PhyTohormone|PlantSignal	Plant Signaling Volatiles	Plant Signal-Propanediol Volatile Metabolism	Plant Signal-1|2-Propanediol Volatile Biosynthesis	gldA|dhaD->PGPT0008270	1

	Production					
Direct Effects	PhyTohormone|PlantSignal Production	Plant Signaling Volatiles	Plant Signal-Sulfuric Volatile Metabolism	Plant Signal-H2S-Volatile Pathway	cysA->PGPT0002990	1
Direct Effects	PhyTohormone|PlantSignal Production	Plant Signaling Volatiles	Plant Signal-Sulfuric Volatile Metabolism	Plant Signal-H2S-Volatile Pathway	cysC->PGPT0002780	2
Direct Effects	PhyTohormone|PlantSignal Production	Plant Signaling Volatiles	Plant Signal-Sulfuric Volatile Metabolism	Plant Signal-H2S-Volatile Pathway	cysE->PGPT0002970	1
Direct Effects	PhyTohormone|PlantSignal Production	Plant Signaling Volatiles	Plant Signal-Sulfuric Volatile Metabolism	Plant Signal-H2S-Volatile Pathway	cysH->PGPT0002785	1
Direct Effects	PhyTohormone|PlantSignal Production	Plant Signaling Volatiles	Plant Signal-Sulfuric Volatile Metabolism	Plant Signal-H2S-Volatile Pathway	cysI->PGPT0002790	1
Direct Effects	PhyTohormone|PlantSignal Production	Plant Signaling Volatiles	Plant Signal-Sulfuric Volatile Metabolism	Plant Signal-H2S-Volatile Pathway	cysJ->PGPT0002795	1

Direct Effects	PhyTohormone|PlantSignal Production	Plant Signaling Volatiles	Plant Signal-Sulfuric Volatile Metabolism	Plant Signal-H2S-Volatile Pathway	cysK->PGPT0002810	3
Direct Effects	PhyTohormone|PlantSignal Production	Plant Signaling Volatiles	Plant Signal-Sulfuric Volatile Metabolism	Plant Signal-H2S-Volatile Pathway	cysK2->PGPT0002815	1
Direct Effects	PhyTohormone|PlantSignal Production	Plant Signaling Volatiles	Plant Signal-Sulfuric Volatile Metabolism	Plant Signal-H2S-Volatile Pathway	cysS->PGPT0002985	1
Direct Effects	PhyTohormone|PlantSignal Production	Plant Signaling Volatiles	Plant Signal-Sulfuric Volatile Metabolism	Plant Signal-H2S-Volatile Pathway	cysT|cysU->PGPT0003000	1
Direct Effects	PhyTohormone|PlantSignal Production	Plant Signaling Volatiles	Plant Signal-Sulfuric Volatile Metabolism	Plant Signal-H2S-Volatile Pathway	cysW->PGPT0003005	1
Direct Effects	PhyTohormone|PlantSignal Production	Plant Signaling Volatiles	Plant Signal-Sulfuric Volatile Metabolism	Plant Signal-H2S-Volatile Pathway	sat|met3->PGPT0002410	1
Direct Effects	PhyTohormone|Pla	Plant Signaling Volatiles	Plant Signal-Volatile Related Metabolism	Plant Signal-Volatile Related	OXCT Like->PGPT0008335	1

	ntSignal Production			Alcohol|Ketone Pathway		
Direct Effects	PhyTohormone|PlantSignal Production	Plant Signaling Volatiles	Plant Signal-Volatile Related Metabolism	Plant Signal-Volatile Related Alcohol|Ketone Pathway	adh1->PGPT0008305	2
Direct Effects	PhyTohormone|PlantSignal Production	Plant Signaling Volatiles	Plant Signal-Volatile Related Metabolism	Plant Signal-Volatile Related Alcohol|Ketone Pathway	adh2 2->PGPT0008295	3
Direct Effects	PhyTohormone|PlantSignal Production	Plant Signaling Volatiles	Plant Signal-Volatile Related Metabolism	Plant Signal-Volatile Related Alcohol|Ketone Pathway	bdh->PGPT0008310	2
Direct Effects	PhyTohormone|PlantSignal Production	Plant Signaling Volatiles	Plant Signal-Volatile Related Metabolism	Plant Signal-Volatile Related Alcohol|Ketone Pathway	gbsB->PGPT0008300	1
Direct Effects	PhyTohormone|PlantSignal Production	Plant Signaling Volatiles	Plant Signal-Volatile Related Metabolism	Plant Signal-Volatile Related Alcohol|Ketone Pathway	hmgL->PGPT0008315	2
Direct Effects	PhyTohormone|PlantSignal Productio	Plant Signaling Volatiles	Plant Signal-Volatile Related Metabolism	Plant Signal-Volatile Related Alcohol|Ketone Pathway	scoA->PGPT0008325	2

	n					
Direct Effects	PhyTohormone|PlantSignal Production	Plant Signaling Volatiles	Plant Signal-Volatile Related Metabolism	Plant Signal-Volatile Related Alcohol|Ketone Pathway	scoB->PGPT0008330	1
Direct Effects	PhyTohormone|PlantSignal Production	Plant Signaling Volatiles	Plant Signal-Volatile Related Metabolism	Plant Signal-Volatile Related Fatty Acid Pathway	accA->PGPT0001695	1
Direct Effects	PhyTohormone|PlantSignal Production	Plant Signaling Volatiles	Plant Signal-Volatile Related Metabolism	Plant Signal-Volatile Related Fatty Acid Pathway	accB|bccP->PGPT0001700	3
Direct Effects	PhyTohormone|PlantSignal Production	Plant Signaling Volatiles	Plant Signal-Volatile Related Metabolism	Plant Signal-Volatile Related Fatty Acid Pathway	accC->PGPT0001705	3
Direct Effects	PhyTohormone|PlantSignal Production	Plant Signaling Volatiles	Plant Signal-Volatile Related Metabolism	Plant Signal-Volatile Related Fatty Acid Pathway	accD->PGPT0001710	1
Direct Effects	PhyTohormone|PlantSignal Production	Plant Signaling Volatiles	Plant Signal-Volatile Related Metabolism	Plant Signal-Volatile Related Fatty Acid Pathway	acd->PGPT0008385	2
Direct	PhyTohor	Plant Signaling	Plant Signal-Volatile	Plant Signal-Volatile	desA->PGPT0008400	2

Effects	mone|PlantSignal Production	Volatiles	Related Metabolism	Related Fatty Acid Pathway		
Direct Effects	PhyTohormone|PlantSignal Production	Plant Signaling Volatiles	Plant Signal-Volatile Related Metabolism	Plant Signal-Volatile Related Fatty Acid Pathway	fabD|bmyD->PGPT0008350	1
Direct Effects	PhyTohormone|PlantSignal Production	Plant Signaling Volatiles	Plant Signal-Volatile Related Metabolism	Plant Signal-Volatile Related Fatty Acid Pathway	fabF->PGPT0008360	1
Direct Effects	PhyTohormone|PlantSignal Production	Plant Signaling Volatiles	Plant Signal-Volatile Related Metabolism	Plant Signal-Volatile Related Fatty Acid Pathway	fabH->PGPT0008355	4
Direct Effects	PhyTohormone|PlantSignal Production	Plant Signaling Volatiles	Plant Signal-Volatile Related Metabolism	Plant Signal-Volatile Related Fatty Acid Pathway	fabI->PGPT0008370	1
Direct Effects	PhyTohormone|PlantSignal Production	Plant Signaling Volatiles	Plant Signal-Volatile Related Metabolism	Plant Signal-Volatile Related Fatty Acid Pathway	fabL->PGPT0008375	1
Direct Effects	PhyTohormone|PlantSignal	Plant Signaling Volatiles	Plant Signal-Volatile Related Metabolism	Plant Signal-Volatile Related Fatty Acid Pathway	fabZ->PGPT0008365	3

	Production					
Direct Effects	PhyTohormone|PlantSignal Production	Plant Signaling Volatiles	Plant Signal-Volatile Related Metabolism	Plant Signal-Volatile Related Fatty Acid Pathway	fadB->PGPT0008390	2
Direct Effects	PhyTohormone|PlantSignal Production	Plant Signaling Volatiles	Plant Signal-Volatile Related Metabolism	Plant Signal-Volatile Related Fatty Acid Pathway	fadD->PGPT0008380	6
Direct Effects	PhyTohormone|PlantSignal Production	Plant Signaling Volatiles	Plant Signal-Volatile Related Metabolism	Plant Signal-Volatile Related Fatty Acid Pathway	fadN->PGPT0008395	1
Direct Effects	PhyTohormone|PlantSignal Production	Plant Signaling Volatiles	Plant Signal-Volatile Related Metabolism	Plant Signal-Volatile Related Fatty Acid Pathway	ymfI|fabG|efpI->PGPT0003180	14
Direct Effects	PhyTohormone|PlantSignal Production	Plant Vitamin Production	Plant Vitamin-Molybdenum CoFactor Metabolism	Plant Vitamin-Molybdenum CoFactor Biosynthesis	moaA->PGPT0008410	1
Direct Effects	PhyTohormone|PlantSignal Production	Plant Vitamin Production	Plant Vitamin-Molybdenum CoFactor Metabolism	Plant Vitamin-Molybdenum CoFactor Biosynthesis	moaB->PGPT0008420	1

Direct Effects	PhyTohormone|PlantSignal Production	Plant Vitamin Production	Plant Vitamin-Molybdenum CoFactor Metabolism	Plant Vitamin-Molybdenum CoFactor Biosynthesis	moaC ->PGPT0008405	1
Direct Effects	PhyTohormone|PlantSignal Production	Plant Vitamin Production	Plant Vitamin-Molybdenum CoFactor Metabolism	Plant Vitamin-Molybdenum CoFactor Biosynthesis	moaX->PGPT0008415	1
Direct Effects	PhyTohormone|PlantSignal Production	Plant Vitamin Production	Plant Vitamin-Molybdenum CoFactor Metabolism	Plant Vitamin-Molybdenum CoFactor Biosynthesis	moeA->PGPT0008430	3
Direct Effects	PhyTohormone|PlantSignal Production	Plant Vitamin Production	Plant Vitamin-Molybdenum CoFactor Metabolism	Plant Vitamin-Molybdenum CoFactor transport	modA->PGPT0008435	1
Direct Effects	PhyTohormone|PlantSignal Production	Plant Vitamin Production	Plant Vitamin-Molybdenum CoFactor Metabolism	Plant Vitamin-Molybdenum CoFactor transport	modB->PGPT0008440	1
Direct Effects	PhyTohormone|PlantSignal Production	Plant Vitamin Production	Plant Vitamin B12|Cobalamin Metabolism	Plant Vitamin B12-Related Proteins	bluB|drgA->PGPT0006810	1
Direct Effects	PhyTohormone|Pla	Plant Vitamin Production	Plant Vitamin B12|Cobalamin	Plant Vitamin B12|Cobalamin	cbiA->PGPT0004605	1

	ntSignal Production		Metabolism	Biosynthesis		
Direct Effects	PhyTohormone|PlantSignal Production	Plant Vitamin Production	Plant Vitamin B12|Cobalamin Metabolism	Plant Vitamin B12|Cobalamin Biosynthesis	cbiB|cobD->PGPT0004660	1
Direct Effects	PhyTohormone|PlantSignal Production	Plant Vitamin Production	Plant Vitamin B12|Cobalamin Metabolism	Plant Vitamin B12|Cobalamin Biosynthesis	cbiC->PGPT0004610	1
Direct Effects	PhyTohormone|PlantSignal Production	Plant Vitamin Production	Plant Vitamin B12|Cobalamin Metabolism	Plant Vitamin B12|Cobalamin Biosynthesis	cbiD->PGPT0004615	1
Direct Effects	PhyTohormone|PlantSignal Production	Plant Vitamin Production	Plant Vitamin B12|Cobalamin Metabolism	Plant Vitamin B12|Cobalamin Biosynthesis	cbiF->PGPT0004625	1
Direct Effects	PhyTohormone|PlantSignal Production	Plant Vitamin Production	Plant Vitamin B12|Cobalamin Metabolism	Plant Vitamin B12|Cobalamin Biosynthesis	cbiG->PGPT0004630	1
Direct Effects	PhyTohormone|PlantSignal Productio	Plant Vitamin Production	Plant Vitamin B12|Cobalamin Metabolism	Plant Vitamin B12|Cobalamin Biosynthesis	cbiH60->PGPT0009260	1

	n					
Direct Effects	PhyTohormone|PlantSignal Production	Plant Vitamin Production	Plant Vitamin B12|Cobalamin Metabolism	Plant Vitamin B12|Cobalamin Biosynthesis	cbiJ->PGPT0004640	1
Direct Effects	PhyTohormone|PlantSignal Production	Plant Vitamin Production	Plant Vitamin B12|Cobalamin Metabolism	Plant Vitamin B12|Cobalamin Biosynthesis	cbiL->PGPT0004690	1
Direct Effects	PhyTohormone|PlantSignal Production	Plant Vitamin Production	Plant Vitamin B12|Cobalamin Metabolism	Plant Vitamin B12|Cobalamin Biosynthesis	cbiX->PGPT0009235	1
Direct Effects	PhyTohormone|PlantSignal Production	Plant Vitamin Production	Plant Vitamin B12|Cobalamin Metabolism	Plant Vitamin B12|Cobalamin Biosynthesis	cbiZ->PGPT0009300	1
Direct Effects	PhyTohormone|PlantSignal Production	Plant Vitamin Production	Plant Vitamin B12|Cobalamin Metabolism	Plant Vitamin B12|Cobalamin Biosynthesis	cobA|btuR->PGPT0004645	1
Direct Effects	PhyTohormone|PlantSignal Production	Plant Vitamin Production	Plant Vitamin B12|Cobalamin Metabolism	Plant Vitamin B12|Cobalamin Biosynthesis	cobC|phpB->PGPT0004650	1
Direct	PhyTohor	Plant Vitamin	Plant Vitamin	Plant Vitamin	cobD->PGPT0009315	1

Effects	mone|PlantSignal Production	Production	B12|Cobalamin Metabolism	B12|Cobalamin Biosynthesis		
Direct Effects	PhyTohormone|PlantSignal Production	Plant Vitamin Production	Plant Vitamin B12|Cobalamin Metabolism	Plant Vitamin B12|Cobalamin Biosynthesis	cobL->PGPT0009270	1
Direct Effects	PhyTohormone|PlantSignal Production	Plant Vitamin Production	Plant Vitamin B12|Cobalamin Metabolism	Plant Vitamin B12|Cobalamin Biosynthesis	cobP|cobU->PGPT0004585	1
Direct Effects	PhyTohormone|PlantSignal Production	Plant Vitamin Production	Plant Vitamin B12|Cobalamin Metabolism	Plant Vitamin B12|Cobalamin Biosynthesis	cobQ|cbiP->PGPT0004580	1
Direct Effects	PhyTohormone|PlantSignal Production	Plant Vitamin Production	Plant Vitamin B12|Cobalamin Metabolism	Plant Vitamin B12|Cobalamin Biosynthesis	cobS|cobV->PGPT0004590	1
Direct Effects	PhyTohormone|PlantSignal Production	Plant Vitamin Production	Plant Vitamin B12|Cobalamin Metabolism	Plant Vitamin B12|Cobalamin Biosynthesis	cobU|cobT->PGPT0004595	3
Direct Effects	PhyTohormone|PlantSignal	Plant Vitamin Production	Plant Vitamin B12|Cobalamin Metabolism	Plant Vitamin B12|Cobalamin Biosynthesis	rhnA cobC->PGPT0004575	1

	Production					
Direct Effects	PhyTohormone|PlantSignal Production	Plant Vitamin Production	Plant Vitamin B12|Cobalamin Metabolism	Plant Vitamin B12|Cobalamin Biosynthesis	sirA|ylnD|cysG|cobA->PGPT0003685	3
Direct Effects	PhyTohormone|PlantSignal Production	Plant Vitamin Production	Plant Vitamin B12|Cobalamin Metabolism	Plant Vitamin B12|Cobalamin transport	feuB|feuC|chuU|yfhA|hmuU|ABC FEVP|fatC|fatD->PGPT0003770	7
Direct Effects	PhyTohormone|PlantSignal Production	Plant Vitamin Production	Plant Vitamin B1|Thiamine Metabolism	Plant Vitamin B1|Thiamine Biosynthesis	adk|AK->PGPT0009040	1
Direct Effects	PhyTohormone|PlantSignal Production	Plant Vitamin Production	Plant Vitamin B1|Thiamine Metabolism	Plant Vitamin B1|Thiamine Biosynthesis	dxs->PGPT0008960	1
Direct Effects	PhyTohormone|PlantSignal Production	Plant Vitamin Production	Plant Vitamin B1|Thiamine Metabolism	Plant Vitamin B1|Thiamine Biosynthesis	nifS|iscS->PGPT0000065	4
Direct Effects	PhyTohormone|PlantSignal Production	Plant Vitamin Production	Plant Vitamin B1|Thiamine Metabolism	Plant Vitamin B1|Thiamine Biosynthesis	phoA->PGPT0002570	2

Direct Effects	PhyTohormone|PlantSignal Production	Plant Vitamin Production	Plant Vitamin B1|Thiamine Metabolism	Plant Vitamin B1|Thiamine Biosynthesis	rsgA|engC->PGPT0009020	1
Direct Effects	PhyTohormone|PlantSignal Production	Plant Vitamin Production	Plant Vitamin B1|Thiamine Metabolism	Plant Vitamin B1|Thiamine Biosynthesis	tenA->PGPT0009055	2
Direct Effects	PhyTohormone|PlantSignal Production	Plant Vitamin Production	Plant Vitamin B1|Thiamine Metabolism	Plant Vitamin B1|Thiamine Biosynthesis	tenI->PGPT0008975	1
Direct Effects	PhyTohormone|PlantSignal Production	Plant Vitamin Production	Plant Vitamin B1|Thiamine Metabolism	Plant Vitamin B1|Thiamine Biosynthesis	thiC->PGPT0008905	1
Direct Effects	PhyTohormone|PlantSignal Production	Plant Vitamin Production	Plant Vitamin B1|Thiamine Metabolism	Plant Vitamin B1|Thiamine Biosynthesis	thiD->PGPT0008915	1
Direct Effects	PhyTohormone|PlantSignal Production	Plant Vitamin Production	Plant Vitamin B1|Thiamine Metabolism	Plant Vitamin B1|Thiamine Biosynthesis	thiE->PGPT0008995	1
Direct Effects	PhyTohormone|Pla	Plant Vitamin Production	Plant Vitamin B1|Thiamine	Plant Vitamin B1|Thiamine	thiF->PGPT0008935	2

	ntSignal Production		Metabolism	Biosynthesis		
Direct Effects	PhyTohormone|PlantSignal Production	Plant Vitamin Production	Plant Vitamin B1|Thiamine Metabolism	Plant Vitamin B1|Thiamine Biosynthesis	thiG->PGPT0008965	1
Direct Effects	PhyTohormone|PlantSignal Production	Plant Vitamin Production	Plant Vitamin B1|Thiamine Metabolism	Plant Vitamin B1|Thiamine Biosynthesis	thiI->PGPT0008940	1
Direct Effects	PhyTohormone|PlantSignal Production	Plant Vitamin Production	Plant Vitamin B1|Thiamine Metabolism	Plant Vitamin B1|Thiamine Biosynthesis	thiJ->PGPT0008945	4
Direct Effects	PhyTohormone|PlantSignal Production	Plant Vitamin Production	Plant Vitamin B1|Thiamine Metabolism	Plant Vitamin B1|Thiamine Biosynthesis	thiM->PGPT0008990	1
Direct Effects	PhyTohormone|PlantSignal Production	Plant Vitamin Production	Plant Vitamin B1|Thiamine Metabolism	Plant Vitamin B1|Thiamine Biosynthesis	thiN|TPK1|THI80->PGPT0009035	1
Direct Effects	PhyTohormone|PlantSignal Productio	Plant Vitamin Production	Plant Vitamin B1|Thiamine Metabolism	Plant Vitamin B1|Thiamine Biosynthesis	thiO->PGPT0008955	2

	n					
Direct Effects	PhyTohormone|PlantSignal Production	Plant Vitamin Production	Plant Vitamin B1|Thiamine Metabolism	Plant Vitamin B1|Thiamine Biosynthesis	ylmB->PGPT0009060	1
Direct Effects	PhyTohormone|PlantSignal Production	Plant Vitamin Production	Plant Vitamin B1|Thiamine Metabolism	Plant Vitamin B1|Thiamine transport	cytX->PGPT0009075	3
Direct Effects	PhyTohormone|PlantSignal Production	Plant Vitamin Production	Plant Vitamin B1|Thiamine Metabolism	Plant Vitamin B1|Thiamine transport	thiT->PGPT0009100	1
Direct Effects	PhyTohormone|PlantSignal Production	Plant Vitamin Production	Plant Vitamin B2|Riboflavin Metabolism	Plant Vitamin B2|Riboflavin Biosynthesis	RFK|FMN1->PGPT0008615	1
Direct Effects	PhyTohormone|PlantSignal Production	Plant Vitamin Production	Plant Vitamin B2|Riboflavin Metabolism	Plant Vitamin B2|Riboflavin Biosynthesis	bluB|drgA->PGPT0006810	1
Direct Effects	PhyTohormone|PlantSignal Production	Plant Vitamin Production	Plant Vitamin B2|Riboflavin Metabolism	Plant Vitamin B2|Riboflavin Biosynthesis	nfrA1|ywcG->PGPT0000290	1
Direct	PhyTohor	Plant Vitamin	Plant Vitamin	Plant Vitamin	nfrA2|ycnD-	1

Effects	mone|PlantSignal Production	Production	B2|Riboflavin Metabolism	B2|Riboflavin Biosynthesis	>PGPT0000295	
Direct Effects	PhyTohormone|PlantSignal Production	Plant Vitamin Production	Plant Vitamin B2|Riboflavin Metabolism	Plant Vitamin B2|Riboflavin Biosynthesis	ribA->PGPT0007985	1
Direct Effects	PhyTohormone|PlantSignal Production	Plant Vitamin Production	Plant Vitamin B2|Riboflavin Metabolism	Plant Vitamin B2|Riboflavin Biosynthesis	ribBA->PGPT0007990	1
Direct Effects	PhyTohormone|PlantSignal Production	Plant Vitamin Production	Plant Vitamin B2|Riboflavin Metabolism	Plant Vitamin B2|Riboflavin Biosynthesis	ribD->PGPT0008555	2
Direct Effects	PhyTohormone|PlantSignal Production	Plant Vitamin Production	Plant Vitamin B2|Riboflavin Metabolism	Plant Vitamin B2|Riboflavin Biosynthesis	ribE|RIB5|ribC->PGPT0008610	1
Direct Effects	PhyTohormone|PlantSignal Production	Plant Vitamin Production	Plant Vitamin B2|Riboflavin Metabolism	Plant Vitamin B2|Riboflavin Biosynthesis	ribF->PGPT0008625	1
Direct Effects	PhyTohormone|PlantSignal	Plant Vitamin Production	Plant Vitamin B2|Riboflavin Metabolism	Plant Vitamin B2|Riboflavin Biosynthesis	ribH|RIB4->PGPT0008605	1

	Production					
Direct Effects	PhyTohormone|PlantSignal Production	Plant Vitamin Production	Plant Vitamin B2|Riboflavin Metabolism	Plant Vitamin B2|Riboflavin Biosynthesis	ssuE->PGPT0003045	1
Direct Effects	PhyTohormone|PlantSignal Production	Plant Vitamin Production	Plant Vitamin B2|Riboflavin Metabolism	Plant Vitamin B2|Riboflavin Biosynthesis	ycsE|yitU|ywtE->PGPT0008595	5
Direct Effects	PhyTohormone|PlantSignal Production	Plant Vitamin Production	Plant Vitamin B2|Riboflavin Metabolism	Plant Vitamin B2|Riboflavin Biosynthesis	yigB->PGPT0008585	1
Direct Effects	PhyTohormone|PlantSignal Production	Plant Vitamin Production	Plant Vitamin B5|PanTothenic Acid|Co Factor Metabolism	Plant Vitamin B5|PanTothenic Acid|Co Factor Biosynthesis	UPB1 Like|pydC->PGPT0008885	5
Direct Effects	PhyTohormone|PlantSignal Production	Plant Vitamin Production	Plant Vitamin B5|PanTothenic Acid|Co Factor Metabolism	Plant Vitamin B5|PanTothenic Acid|Co Factor Biosynthesis	acpS->PGPT0008840	1
Direct Effects	PhyTohormone|PlantSignal Production	Plant Vitamin Production	Plant Vitamin B5|PanTothenic Acid|Co Factor Metabolism	Plant Vitamin B5|PanTothenic Acid|Co Factor Biosynthesis	budB|ilvK|alsS|ilvB|ilvG|ilvI->PGPT0008185	4

Direct Effects	PhyTohormone|PlantSignal Production	Plant Vitamin Production	Plant Vitamin B5|PanTothenic Acid|Co Factor Metabolism	Plant Vitamin B5|PanTothenic Acid|Co Factor Biosynthesis	coaB->PGPT0008805	2
Direct Effects	PhyTohormone|PlantSignal Production	Plant Vitamin Production	Plant Vitamin B5|PanTothenic Acid|Co Factor Metabolism	Plant Vitamin B5|PanTothenic Acid|Co Factor Biosynthesis	coaBC|dfp->PGPT0008815	1
Direct Effects	PhyTohormone|PlantSignal Production	Plant Vitamin Production	Plant Vitamin B5|PanTothenic Acid|Co Factor Metabolism	Plant Vitamin B5|PanTothenic Acid|Co Factor Biosynthesis	coaD|kdtB->PGPT0008825	1
Direct Effects	PhyTohormone|PlantSignal Production	Plant Vitamin Production	Plant Vitamin B5|PanTothenic Acid|Co Factor Metabolism	Plant Vitamin B5|PanTothenic Acid|Co Factor Biosynthesis	coaE->PGPT0008835	1
Direct Effects	PhyTohormone|PlantSignal Production	Plant Vitamin Production	Plant Vitamin B5|PanTothenic Acid|Co Factor Metabolism	Plant Vitamin B5|PanTothenic Acid|Co Factor Biosynthesis	dht|hydA->PGPT0008880	1
Direct Effects	PhyTohormone|PlantSignal Production	Plant Vitamin Production	Plant Vitamin B5|PanTothenic Acid|Co Factor Metabolism	Plant Vitamin B5|PanTothenic Acid|Co Factor Biosynthesis	ilvC->PGPT0008735	1
Direct Effects	PhyTohormone|Pla	Plant Vitamin Production	Plant Vitamin B5|PanTothenic	Plant Vitamin B5|PanTothenic	ilvD->PGPT0001875	1

	ntSignal Productio n		Acid|Co Factor Metabolism	Acid|Co Factor Biosynthesis		
Direct Effects	PhyTohor mone|Pla ntSignal Productio n	Plant Vitamin Production	Plant Vitamin B5|PanTothenic Acid|Co Factor Metabolism	Plant Vitamin B5|PanTothenic Acid|Co Factor Biosynthesis	ilvE->PGPT0008860	1
Direct Effects	PhyTohor mone|Pla ntSignal Productio n	Plant Vitamin Production	Plant Vitamin B5|PanTothenic Acid|Co Factor Metabolism	Plant Vitamin B5|PanTothenic Acid|Co Factor Biosynthesis	ilvH|ilvN- >PGPT0008205	1
Direct Effects	PhyTohor mone|Pla ntSignal Productio n	Plant Vitamin Production	Plant Vitamin B5|PanTothenic Acid|Co Factor Metabolism	Plant Vitamin B5|PanTothenic Acid|Co Factor Biosynthesis	panB->PGPT0008740	1
Direct Effects	PhyTohor mone|Pla ntSignal Productio n	Plant Vitamin Production	Plant Vitamin B5|PanTothenic Acid|Co Factor Metabolism	Plant Vitamin B5|PanTothenic Acid|Co Factor Biosynthesis	panC->PGPT0008750	1
Direct Effects	PhyTohor mone|Pla ntSignal Productio n	Plant Vitamin Production	Plant Vitamin B5|PanTothenic Acid|Co Factor Metabolism	Plant Vitamin B5|PanTothenic Acid|Co Factor Biosynthesis	panD->PGPT0008890	1
Direct Effects	PhyTohor mone|Pla ntSignal Productio	Plant Vitamin Production	Plant Vitamin B5|PanTothenic Acid|Co Factor Metabolism	Plant Vitamin B5|PanTothenic Acid|Co Factor Biosynthesis	panE|apbA- >PGPT0008745	2

	n					
Direct Effects	PhyTohormone|PlantSignal Production	Plant Vitamin Production	Plant Vitamin B5|PanTothenic Acid|Co Factor Metabolism	Plant Vitamin B5|PanTothenic Acid|Co Factor Biosynthesis	preA->PGPT0008875	1
Direct Effects	PhyTohormone|PlantSignal Production	Plant Vitamin Production	Plant Vitamin B5|PanTothenic Acid|Co Factor Metabolism	Plant Vitamin B5|PanTothenic Acid|Co Factor Biosynthesis	preT->PGPT0008870	1
Direct Effects	PhyTohormone|PlantSignal Production	Plant Vitamin Production	Plant Vitamin B6|Pyridoxine|Pyridoxal|PyridoxAmine Metabolism	Plant Vitamin B6|Pyridoxine|Pyridoxal|PyridoxAmine Biosynthesis	pdxA->PGPT0009165	1
Direct Effects	PhyTohormone|PlantSignal Production	Plant Vitamin Production	Plant Vitamin B6|Pyridoxine|Pyridoxal|PyridoxAmine Metabolism	Plant Vitamin B6|Pyridoxine|Pyridoxal|PyridoxAmine Biosynthesis	pdxK|pdxY->PGPT0009125	1
Direct Effects	PhyTohormone|PlantSignal Production	Plant Vitamin Production	Plant Vitamin B6|Pyridoxine|Pyridoxal|PyridoxAmine Metabolism	Plant Vitamin B6|Pyridoxine|Pyridoxal|PyridoxAmine Biosynthesis	pdxS|pdx1|yaaD->PGPT0009180	2
Direct Effects	PhyTohormone|PlantSignal Production	Plant Vitamin Production	Plant Vitamin B6|Pyridoxine|Pyridoxal|PyridoxAmine Metabolism	Plant Vitamin B6|Pyridoxine|Pyridoxal|PyridoxAmine Biosynthesis	pdxT|pdx2|yaaE->PGPT0009185	2
Direct	PhyTohor	Plant Vitamin	Plant Vitamin	Plant Vitamin	serA->PGPT0009155	2

Effects	mone|PlantSignal Production	Production	B6|Pyridoxine|Pyridoxal|PyridoxAmine Metabolism	B6|Pyridoxine|Pyridoxal|PyridoxAmine Biosynthesis		
Direct Effects	PhyTohormone|PlantSignal Production	Plant Vitamin Production	Plant Vitamin B6|Pyridoxine|Pyridoxal|PyridoxAmine Metabolism	Plant Vitamin B6|Pyridoxine|Pyridoxal|PyridoxAmine Biosynthesis	serC|pdxF->PGPT0009160	1
Direct Effects	PhyTohormone|PlantSignal Production	Plant Vitamin Production	Plant Vitamin B6|Pyridoxine|Pyridoxal|PyridoxAmine Metabolism	Plant Vitamin B6|Pyridoxine|Pyridoxal|PyridoxAmine Biosynthesis	thrC->PGPT0009175	5
Direct Effects	PhyTohormone|PlantSignal Production	Plant Vitamin Production	Plant Vitamin B9|Folate Metabolism	Plant Vitamin B9|Folate Biosynthesis	fhs->PGPT0008065	1
Direct Effects	PhyTohormone|PlantSignal Production	Plant Vitamin Production	Plant Vitamin B9|Folate Metabolism	Plant Vitamin B9|Folate Biosynthesis	fmt->PGPT0008125	1
Direct Effects	PhyTohormone|PlantSignal Production	Plant Vitamin Production	Plant Vitamin B9|Folate Metabolism	Plant Vitamin B9|Folate Biosynthesis	folA->PGPT0007945	1
Direct Effects	PhyTohormone|PlantSignal	Plant Vitamin Production	Plant Vitamin B9|Folate Metabolism	Plant Vitamin B9|Folate Biosynthesis	folB->PGPT0007905	1

	Production					
Direct Effects	PhyTohormone|PlantSignal Production	Plant Vitamin Production	Plant Vitamin B9|Folate Metabolism	Plant Vitamin B9|Folate Biosynthesis	folC->PGPT0007975	1
Direct Effects	PhyTohormone|PlantSignal Production	Plant Vitamin Production	Plant Vitamin B9|Folate Metabolism	Plant Vitamin B9|Folate Biosynthesis	folD->PGPT0008075	1
Direct Effects	PhyTohormone|PlantSignal Production	Plant Vitamin Production	Plant Vitamin B9|Folate Metabolism	Plant Vitamin B9|Folate Biosynthesis	folE->PGPT0007875	2
Direct Effects	PhyTohormone|PlantSignal Production	Plant Vitamin Production	Plant Vitamin B9|Folate Metabolism	Plant Vitamin B9|Folate Biosynthesis	folE2->PGPT0007880	2
Direct Effects	PhyTohormone|PlantSignal Production	Plant Vitamin Production	Plant Vitamin B9|Folate Metabolism	Plant Vitamin B9|Folate Biosynthesis	folK->PGPT0007910	1
Direct Effects	PhyTohormone|PlantSignal Production	Plant Vitamin Production	Plant Vitamin B9|Folate Metabolism	Plant Vitamin B9|Folate Biosynthesis	folP->PGPT0007915	1

Direct Effects	PhyTohormone|PlantSignal Production	Plant Vitamin Production	Plant Vitamin B9|Folate Metabolism	Plant Vitamin B9|Folate Biosynthesis	gcvT->PGPT0008130	1
Direct Effects	PhyTohormone|PlantSignal Production	Plant Vitamin Production	Plant Vitamin B9|Folate Metabolism	Plant Vitamin B9|Folate Biosynthesis	glyA->PGPT0008090	1
Direct Effects	PhyTohormone|PlantSignal Production	Plant Vitamin Production	Plant Vitamin B9|Folate Metabolism	Plant Vitamin B9|Folate Biosynthesis	metH->PGPT0008135	2
Direct Effects	PhyTohormone|PlantSignal Production	Plant Vitamin Production	Plant Vitamin B9|Folate Metabolism	Plant Vitamin B9|Folate Biosynthesis	pabA->PGPT0008000	1
Direct Effects	PhyTohormone|PlantSignal Production	Plant Vitamin Production	Plant Vitamin B9|Folate Metabolism	Plant Vitamin B9|Folate Biosynthesis	pabB->PGPT0008005	1
Direct Effects	PhyTohormone|PlantSignal Production	Plant Vitamin Production	Plant Vitamin B9|Folate Metabolism	Plant Vitamin B9|Folate Biosynthesis	pabC->PGPT0008020	1
Direct Effects	PhyTohormone|Pla	Plant Vitamin Production	Plant Vitamin B9|Folate Metabolism	Plant Vitamin B9|Folate Biosynthesis	phoA->PGPT0002570	2

	ntSignal Production					
Direct Effects	PhyTohormone|PlantSignal Production	Plant Vitamin Production	Plant Vitamin B9|Folate Metabolism	Plant Vitamin B9|Folate Biosynthesis	phoD->PGPT0002575	1
Direct Effects	PhyTohormone|PlantSignal Production	Plant Vitamin Production	Plant Vitamin B9|Folate Metabolism	Plant Vitamin B9|Folate Biosynthesis	purH->PGPT0008110	2
Direct Effects	PhyTohormone|PlantSignal Production	Plant Vitamin Production	Plant Vitamin B9|Folate Metabolism	Plant Vitamin B9|Folate Biosynthesis	purN->PGPT0008095	1
Direct Effects	PhyTohormone|PlantSignal Production	Plant Vitamin Production	Plant Vitamin B9|Folate Metabolism	Plant Vitamin B9|Folate Biosynthesis	purU->PGPT0008155	1
Direct Effects	PhyTohormone|PlantSignal Production	Plant Vitamin Production	Plant Vitamin B9|Folate Metabolism	Plant Vitamin B9|Folate Biosynthesis	ribA->PGPT0007985	1
Direct Effects	PhyTohormone|PlantSignal Productio	Plant Vitamin Production	Plant Vitamin B9|Folate Metabolism	Plant Vitamin B9|Folate Biosynthesis	ribBA->PGPT0007990	1

	n					
Direct Effects	PhyTohormone|PlantSignal Production	Plant Vitamin Production	Plant Vitamin B9|Folate Metabolism	Plant Vitamin B9|Folate Biosynthesis	thyA->PGPT0008145	1
Direct Effects	PhyTohormone|PlantSignal Production	Plant Vitamin Production	Plant Vitamin B9|Folate Metabolism	Plant Vitamin B9|Folate Biosynthesis	ygfA|fthC|yqgN|folN ->PGPT0008170	1
Direct Effects	PhyTohormone|PlantSignal Production	Plant Vitamin Production	Plant Vitamin B9|Folate Metabolism	Plant Vitamin B9|Folate Biosynthesis	yitJ->PGPT0008140	1
Direct Effects	PhyTohormone|PlantSignal Production	Plant Vitamin Production	Plant Vitamin C|Ascorbic Acid Metabolism	Plant Vitamin C|Ascorbic Acid Biosynthesis	dkgA->PGPT0001320	3
Direct Effects	PhyTohormone|PlantSignal Production	Plant Vitamin Production	Plant Vitamin K Metabolism	Plant Vitamin K Biosynthesis	menA->PGPT0009395	1
Direct Effects	PhyTohormone|PlantSignal Production	Plant Vitamin Production	Plant Vitamin K Metabolism	Plant Vitamin K Biosynthesis	menB->PGPT0009385	1
Direct	PhyTohor	Plant Vitamin	Plant Vitamin K	Plant Vitamin K	menC->PGPT0009375	2

Effects	mone∣PlantSignal Production	Production	Metabolism	Biosynthesis		
Direct Effects	PhyTohormone∣PlantSignal Production	Plant Vitamin Production	Plant Vitamin K Metabolism	Plant Vitamin K Biosynthesis	menD->PGPT0009365	1
Direct Effects	PhyTohormone∣PlantSignal Production	Plant Vitamin Production	Plant Vitamin K Metabolism	Plant Vitamin K Biosynthesis	menE->PGPT0009380	1
Direct Effects	PhyTohormone∣PlantSignal Production	Plant Vitamin Production	Plant Vitamin K Metabolism	Plant Vitamin K Biosynthesis	menF->PGPT0009360	1
Direct Effects	PhyTohormone∣PlantSignal Production	Plant Vitamin Production	Plant Vitamin K Metabolism	Plant Vitamin K Biosynthesis	menH->PGPT0009370	1
Direct Effects	PhyTohormone∣PlantSignal Production	Plant Vitamin Production	Plant Vitamin K Metabolism	Plant Vitamin K Biosynthesis	menI∣ydiI∣ydiL∣ydiI∣ybdB->PGPT0001845	1
Direct Effects	PhyTohormone∣PlantSignal	Plant Vitamin Production	Plant Vitamin K Metabolism	Plant Vitamin K Biosynthesis	qorB->PGPT0009465	2

	Production					
Direct Effects	PhyTohormone|PlantSignal Production	Plant Vitamin Production	Plant Vitamin K Metabolism	Plant Vitamin K Biosynthesis	wrbA->PGPT0009460	1
Direct Effects	PhyTohormone|PlantSignal Production	Plant Vitamin Production	Plant Vitamin Related Heme Metabolism	Plant Vitamin-Heme|Siro-Biosynthesis	ahbC->PGPT0008510	1
Direct Effects	PhyTohormone|PlantSignal Production	Plant Vitamin Production	Plant Vitamin Related Heme Metabolism	Plant Vitamin-Heme|Siro-Biosynthesis	ctaA->PGPT0008525	2
Direct Effects	PhyTohormone|PlantSignal Production	Plant Vitamin Production	Plant Vitamin Related Heme Metabolism	Plant Vitamin-Heme|Siro-Biosynthesis	ctaB|cyoE->PGPT0008520	2
Direct Effects	PhyTohormone|PlantSignal Production	Plant Vitamin Production	Plant Vitamin Related Heme Metabolism	Plant Vitamin-Heme|Siro-Biosynthesis	cysG->PGPT0003690	1
Direct Effects	PhyTohormone|PlantSignal Production	Plant Vitamin Production	Plant Vitamin Related Heme Metabolism	Plant Vitamin-Heme|Siro-Biosynthesis	gltX->PGPT0008460	1

Direct Effects	PhyTohormone|PlantSignal Production	Plant Vitamin Production	Plant Vitamin Related Heme Metabolism	Plant Vitamin-Heme|Siro-Biosynthesis	hemA->PGPT0003650	1
Direct Effects	PhyTohormone|PlantSignal Production	Plant Vitamin Production	Plant Vitamin Related Heme Metabolism	Plant Vitamin-Heme|Siro-Biosynthesis	hemB->PGPT0003655	1
Direct Effects	PhyTohormone|PlantSignal Production	Plant Vitamin Production	Plant Vitamin Related Heme Metabolism	Plant Vitamin-Heme|Siro-Biosynthesis	hemC->PGPT0003660	1
Direct Effects	PhyTohormone|PlantSignal Production	Plant Vitamin Production	Plant Vitamin Related Heme Metabolism	Plant Vitamin-Heme|Siro-Biosynthesis	hemD->PGPT0003665	2
Direct Effects	PhyTohormone|PlantSignal Production	Plant Vitamin Production	Plant Vitamin Related Heme Metabolism	Plant Vitamin-Heme|Siro-Biosynthesis	Heme->PGPT0008465	1
Direct Effects	PhyTohormone|PlantSignal Production	Plant Vitamin Production	Plant Vitamin Related Heme Metabolism	Plant Vitamin-Heme|Siro-Biosynthesis	hemH|ywfI->PGPT0008485	1
Direct Effects	PhyTohormone|Pla	Plant Vitamin Production	Plant Vitamin Related Heme Metabolism	Plant Vitamin-Heme|Siro-	hemL->PGPT0003680	2

	ntSignal Production			Biosynthesis		
Direct Effects	PhyTohormone|PlantSignal Production	Plant Vitamin Production	Plant Vitamin Related Heme Metabolism	Plant Vitamin-Heme|Siro-Biosynthesis	hemN|hemZ->PGPT0003200	2
Direct Effects	PhyTohormone|PlantSignal Production	Plant Vitamin Production	Plant Vitamin Related Heme Metabolism	Plant Vitamin-Heme|Siro-Biosynthesis	hemQ->PGPT0008490	1
Direct Effects	PhyTohormone|PlantSignal Production	Plant Vitamin Production	Plant Vitamin Related Heme Metabolism	Plant Vitamin-Heme|Siro-Biosynthesis	hemY->PGPT0008475	2
Direct Effects	PhyTohormone|PlantSignal Production	Plant Vitamin Production	Plant Vitamin Related Heme Metabolism	Plant Vitamin-Heme|Siro-Biosynthesis	sirA|ylnD|cysG|cobA->PGPT0003685	3
Direct Effects	PhyTohormone|PlantSignal Production	Plant Vitamin Production	Plant Vitamin Related Heme Metabolism	Plant Vitamin-Heme|Siro-Biosynthesis	sirB->PGPT0008500	1
Direct Effects	PhyTohormone|PlantSignal Productio	Plant Vitamin Production	Plant Vitamin Restorage-Lipoic Acid	Plant Vitamin Restorage-Lipoic Acid Biosynthesis	lipA->PGPT0003935	1

	n					
Direct Effects	PhyTohormone|PlantSignal Production	Plant Vitamin Production	Plant Vitamin Restorage-Lipoic Acid	Plant Vitamin Restorage-Lipoic Acid Biosynthesis	lipL->PGPT0003940	1
Indirect Effects	ColonizingPlant System	Colonization-Adaptation To Plant Immune System	Adaptation To PIS-Gallate|Tannine Degradation	Adaptation To PIS-Gallate Degradation Pathway	ligK|galC->PGPT0002085	5
Indirect Effects	ColonizingPlant System	Colonization-Adaptation To Plant Immune System	Adaptation To PIS-Inhibition Of Salicylic Acid|Jasmonic Acid	Adaptation To PIS-Salicylic Acid Degradation	bsdA->PGPT0019770	4
Indirect Effects	ColonizingPlant System	Colonization-Adaptation To Plant Immune System	Adaptation To PIS-Inhibition Of Salicylic Acid|Jasmonic Acid	Adaptation To PIS-Salicylic Acid Degradation	bsdC->PGPT0005435	1
Indirect Effects	ColonizingPlant System	Colonization-Adaptation To Plant Immune System	Adaptation To PIS-Inhibition Of Salicylic Acid|Jasmonic Acid	Adaptation To PIS-Salicylic Acid Degradation	ubiX|bsdB->PGPT0009565	1
Indirect Effects	ColonizingPlant System	Colonization-Adaptation To Plant Immune System	Adaptation To PIS-Plant 2-Methylene-4-Butyrolactone Resistance	Adaptation To PIS-2-Methylene-4-Butyrolactone Degradation	mdaB->PGPT0023600	1
Indirect Effects	ColonizingPlant System	Colonization-Adaptation To Plant Immune System	Adaptation To PIS-Plant 3-Nitropropionate|3-NPA Resistance	Adaptation To PIS-3-Nitropropionate|3-NPA Detoxification	ncd2|npd|pnoA->PGPT0006800	4
Indirect Effects	ColonizingPlant System	Colonization-Adaptation To Plant Immune System	Adaptation To PIS-Plant Cinnamate Degradation	Adaptation To PIS-CA Degradation-Acetaldehyde Pathway	mhpC->PGPT0019765	2
Indirect Effects	ColonizingPlant	Colonization-Adaptation To Plant	Adaptation To PIS-Plant Cinnamate	Adaptation To PIS-CA Degradation-	mhpE->PGPT0002050	1

	System	Immune System	Degradation	Acetaldehyde Pathway		
Indirect Effects	Colonizin gPlant System	Colonization-Adaptation To Plant Immune System	Adaptation To PIS-Plant Curcumin Resistance	Adaptation To PIS-Curcumin Degradation	curA|yncB->PGPT0023605	1
Indirect Effects	Colonizin gPlant System	Colonization-Adaptation To Plant Immune System	Adaptation To PIS-Plant HydroxyCinnamic Acid Resistance	Adaptation To PIS-HCA Degradation-Vanillin Intermediate	paaF|echA->PGPT0001860	1
Indirect Effects	Colonizin gPlant System	Colonization-Adaptation To Plant Immune System	Adaptation To PIS-Plant HydroxyCinnamic Acid Resistance	Adaptation To PIS-Protocatechuic Acid Degradation	pcaC->PGPT0005005	6
Indirect Effects	Colonizin gPlant System	Colonization-Adaptation To Plant Immune System	Adaptation To PIS-Plant HydroxyCinnamic Acid Resistance	Adaptation To PIS-Protocatechuic Acid Degradation	pcaD|catD->PGPT0004995	3
Indirect Effects	Colonizin gPlant System	Colonization-Adaptation To Plant Immune System	Adaptation To PIS-Plant Lipoic Acid Interference	Plant Lipoic Acid Biosynthesis	lipA->PGPT0003935	1
Indirect Effects	Colonizin gPlant System	Colonization-Adaptation To Plant Immune System	Adaptation To PIS-Plant Lipoic Acid Interference	Plant Lipoic Acid Biosynthesis	lipL->PGPT0003940	1
Indirect Effects	Colonizin gPlant System	Colonization-Motility|Chemotaxis	Chemotaxis Proteins	Chemotaxis Related Proteins	dnaA->PGPT0014800	1
Indirect Effects	Colonizin gPlant System	Colonization-Motility|Chemotaxis	Chemotaxis Proteins	Chemotaxis Towards Sugars	rbsB->PGPT0015740	1
Indirect Effects	Colonizin gPlant System	Colonization-Motility|Chemotaxis	Chemotaxis Proteins	Chemotaxis Two Component System Proteins	cheA|wspE->PGPT0015645	2

Indirect Effects	Colonizin gPlant System	Colonization-Motility|Chemotaxis	Chemotaxis Proteins	Chemotaxis Two Component System Proteins	cheBR->PGPT0015655	1
Indirect Effects	Colonizin gPlant System	Colonization-Motility|Chemotaxis	Chemotaxis Proteins	Chemotaxis Two Component System Proteins	cheB|chpB|wspF->PGPT0015650	1
Indirect Effects	Colonizin gPlant System	Colonization-Motility|Chemotaxis	Chemotaxis Proteins	Chemotaxis Two Component System Proteins	cheD->PGPT0015665	1
Indirect Effects	Colonizin gPlant System	Colonization-Motility|Chemotaxis	Chemotaxis Proteins	Chemotaxis Two Component System Proteins	cheR|pilK->PGPT0015670	2
Indirect Effects	Colonizin gPlant System	Colonization-Motility|Chemotaxis	Chemotaxis Proteins	Chemotaxis Two Component System Proteins	cheV->PGPT0015675	1
Indirect Effects	Colonizin gPlant System	Colonization-Motility|Chemotaxis	Chemotaxis Proteins	Chemotaxis Two Component System Proteins	cheW->PGPT0015680	1
Indirect Effects	Colonizin gPlant System	Colonization-Motility|Chemotaxis	Chemotaxis Proteins	Chemotaxis Two Component System Proteins	cheY|yneI->PGPT0015690	1
Indirect Effects	Colonizin gPlant System	Colonization-Motility|Chemotaxis	Chemotaxis Proteins	Methyl-Accepting Chemotaxis Proteins	hemAT->PGPT0015705	1
Indirect Effects	Colonizin gPlant System	Colonization-Motility|Chemotaxis	Chemotaxis Proteins	Methyl-Accepting Chemotaxis Proteins	mcp|tlpC|tlpA|dcrA->PGPT0015710	2
Indirect Effects	Colonizin gPlant System	Colonization-Motility|Chemotaxis	Motility-Flagellar Assembly	Motility-Flagellum C-Ring	fliG->PGPT0015400	1
Indirect Effects	Colonizin gPlant	Colonization-Motility|Chemotaxis	Motility-Flagellar Assembly	Motility-Flagellum C-Ring	fliM->PGPT0015405	1

	System					
Indirect Effects	ColonizingPlant System	Colonization-Motility|Chemotaxis	Motility-Flagellar Assembly	Motility-Flagellum C-Ring	fliN|lfiN|fliNY|cheC|cheD->PGPT0015410	1
Indirect Effects	ColonizingPlant System	Colonization-Motility|Chemotaxis	Motility-Flagellar Assembly	Motility-Flagellum Motor|Switch	motA->PGPT0015370	2
Indirect Effects	ColonizingPlant System	Colonization-Motility|Chemotaxis	Motility-Flagellar Assembly	Motility-Flagellum Motor|Switch	motB->PGPT0015375	2
Indirect Effects	ColonizingPlant System	Colonization-Motility|Chemotaxis	Motility-Flagellar Assembly	Motility-Flagellum M|S|P|L-Rings	fliF->PGPT0015430	1
Indirect Effects	ColonizingPlant System	Colonization-Motility|Chemotaxis	Motility-Flagellar Assembly	Motility-Flagellum Rod|Hook|Filament	flbD->PGPT0015460	1
Indirect Effects	ColonizingPlant System	Colonization-Motility|Chemotaxis	Motility-Flagellar Assembly	Motility-Flagellum Rod|Hook|Filament	flgB->PGPT0015470	1
Indirect Effects	ColonizingPlant System	Colonization-Motility|Chemotaxis	Motility-Flagellar Assembly	Motility-Flagellum Rod|Hook|Filament	flgC->PGPT0015475	1
Indirect Effects	ColonizingPlant System	Colonization-Motility|Chemotaxis	Motility-Flagellar Assembly	Motility-Flagellum Rod|Hook|Filament	flgD->PGPT0015480	1
Indirect Effects	ColonizingPlant System	Colonization-Motility|Chemotaxis	Motility-Flagellar Assembly	Motility-Flagellum Rod|Hook|Filament	flgE->PGPT0015485	1
Indirect Effects	ColonizingPlant System	Colonization-Motility|Chemotaxis	Motility-Flagellar Assembly	Motility-Flagellum Rod|Hook|Filament	flgF->PGPT0015490	1
Indirect	Colonizin	Colonization-	Motility-Flagellar	Motility-Flagellum	flgK->PGPT0015195	1

Effects	gPlant<br>System	Motility|Chemotaxis	Assembly	Rod|Hook|Filament		
Indirect<br>Effects	Colonizin<br>gPlant<br>System	Colonization-<br>Motility|Chemotaxis	Motility-Flagellar<br>Assembly	Motility-Flagellum<br>Rod|Hook|Filament	flgL->PGPT0015505	1
Indirect<br>Effects	Colonizin<br>gPlant<br>System	Colonization-<br>Motility|Chemotaxis	Motility-Flagellar<br>Assembly	Motility-Flagellum<br>Rod|Hook|Filament	fliC|laf1|lafA|fla1|ha<br>g->PGPT0015190	1
Indirect<br>Effects	Colonizin<br>gPlant<br>System	Colonization-<br>Motility|Chemotaxis	Motility-Flagellar<br>Assembly	Motility-Flagellum<br>Rod|Hook|Filament	fliD|flaB-<br>>PGPT0015200	1
Indirect<br>Effects	Colonizin<br>gPlant<br>System	Colonization-<br>Motility|Chemotaxis	Motility-Flagellar<br>Assembly	Motility-Flagellum<br>Rod|Hook|Filament	fliE|lfiE-<br>>PGPT0015515	1
Indirect<br>Effects	Colonizin<br>gPlant<br>System	Colonization-<br>Motility|Chemotaxis	Motility-Flagellar<br>Assembly	Motility-Flagellum<br>Rod|Hook|Filament	fliK|motD-<br>>PGPT0015520	1
Indirect<br>Effects	Colonizin<br>gPlant<br>System	Colonization-<br>Motility|Chemotaxis	Motility-Flagellar<br>Assembly	Motility-Flagellum<br>Rod|Hook|Filament	fliL->PGPT0015525	1
Indirect<br>Effects	Colonizin<br>gPlant<br>System	Colonization-<br>Motility|Chemotaxis	Motility-Flagellar<br>Assembly	Motility-Flagellum<br>Type-III Secretion	flhA|lfhA|fhiA|rhcV-<br>>PGPT0015320	1
Indirect<br>Effects	Colonizin<br>gPlant<br>System	Colonization-<br>Motility|Chemotaxis	Motility-Flagellar<br>Assembly	Motility-Flagellum<br>Type-III Secretion	flhB->PGPT0015325	1
Indirect<br>Effects	Colonizin<br>gPlant<br>System	Colonization-<br>Motility|Chemotaxis	Motility-Flagellar<br>Assembly	Motility-Flagellum<br>Type-III Secretion	fliH->PGPT0015335	1
Indirect<br>Effects	Colonizin<br>gPlant<br>System	Colonization-<br>Motility|Chemotaxis	Motility-Flagellar<br>Assembly	Motility-Flagellum<br>Type-III Secretion	fliI|lgiI->PGPT0015340	1

Indirect Effects	ColonizingPlant System	Colonization-Motility|Chemotaxis	Motility-Flagellar Assembly	Motility-Flagellum Type-III Secretion	fliOZ->PGPT0015345	1
Indirect Effects	ColonizingPlant System	Colonization-Motility|Chemotaxis	Motility-Flagellar Assembly	Motility-Flagellum Type-III Secretion	fliP|rhcR->PGPT0015350	1
Indirect Effects	ColonizingPlant System	Colonization-Motility|Chemotaxis	Motility-Flagellar Assembly	Motility-Flagellum Type-III Secretion	fliQ|lfiQ->PGPT0015355	1
Indirect Effects	ColonizingPlant System	Colonization-Motility|Chemotaxis	Motility-Flagellar Assembly	Motility-Flagellum Type-III Secretion	fliR|lfiR->PGPT0015360	1
Indirect Effects	ColonizingPlant System	Colonization-Motility|Chemotaxis	Motility-Flagellum Control	Motility-Flagellum Chaperones	fliA|sigD|whiG->PGPT0015610	1
Indirect Effects	ColonizingPlant System	Colonization-Motility|Chemotaxis	Motility-Flagellum Control	Motility-Flagellum Chaperones	fliS->PGPT0015620	2
Indirect Effects	ColonizingPlant System	Colonization-Motility|Chemotaxis	Motility-Flagellum Control	Motility-Flagellum Chaperones	fliW->PGPT0015630	1
Indirect Effects	ColonizingPlant System	Colonization-Motility|Chemotaxis	Motility-Flagellum Control	Motility-Flagellum Chaperones	fliY|tcyA|yckK->PGPT0015635	1
Indirect Effects	ColonizingPlant System	Colonization-Motility|Chemotaxis	Motility-Flagellum Control	Motility-Flagellum Regulation	dnaA->PGPT0014800	1
Indirect Effects	ColonizingPlant System	Colonization-Motility|Chemotaxis	Motility-Flagellum Control	Motility-Flagellum Regulation	flgM->PGPT0015530	1
Indirect Effects	ColonizingPlant	Colonization-Motility|Chemotaxis	Motility-Flagellum Control	Motility-Flagellum Regulation	flhF->PGPT0015550	1

	System					
Indirect Effects	ColonizingPlant System	Colonization-Motility∣Chemotaxis	Motility-Flagellum Control	Motility-Flagellum Regulation	flhG->PGPT0015555	2
Indirect Effects	ColonizingPlant System	Colonization-Motility∣Chemotaxis	Motility-Flagellum Control	Motility-Flagellum Regulation	ntrA∣rpoN->PGPT0000795	2
Indirect Effects	ColonizingPlant System	Colonization-Motility∣Chemotaxis	Motility-GAS Vesicle Formation	Motility-GAS Vesicle Biosynthesis	gvpA->PGPT0016275	6
Indirect Effects	ColonizingPlant System	Colonization-Motility∣Chemotaxis	Motility-Pilus∣Fimbriae System	Motility-Chemosensory Pili System	chpD->PGPT0015800	1
Indirect Effects	ColonizingPlant System	Colonization-Motility∣Chemotaxis	Motility-Pilus∣Fimbriae System	Motility-Pilus System	dnaA->PGPT0014800	1
Indirect Effects	ColonizingPlant System	Colonization-Motility∣Chemotaxis	Motility-Pilus∣Fimbriae System	Motility-Pilus System	pleD->PGPT0015900	2
Indirect Effects	ColonizingPlant System	Colonization-Motility∣Chemotaxis	Motility-Pilus∣Fimbriae System	Motility-Pilus System	rseP->PGPT0011685	1
Indirect Effects	ColonizingPlant System	Colonization-Motility∣Chemotaxis	Motility-Pilus∣Fimbriae System	Motility-Pilus System-Twitching Motility	ntrA∣rpoN->PGPT0000795	2
Indirect Effects	ColonizingPlant System	Colonization-Motility∣Chemotaxis	Motility-Pilus∣Fimbriae System	Motility-Type IVa Pilus Homologous System	yggT∣ylmG->PGPT0013730	1
Indirect Effects	ColonizingPlant System	Colonization-Motility∣Chemotaxis	Other Motility Regulating Functions	Motility-QuorumSensing Related Regulation	acgA->PGPT0016250	1
Indirect	Colonizin	Colonization-	Other Motility	Motility-	sylA->PGPT0016255	3

Effects	gPlant System	Motility|Chemotaxis	Regulating Functions	QuorumSensing Related Regulation		
Indirect Effects	Colonizin gPlant System	Colonization-Motility|Chemotaxis	Other Motility Regulating Functions	Motility-Swarming Regulator	efp->PGPT0016205	1
Indirect Effects	Colonizin gPlant System	Colonization-Motility|Chemotaxis	Other Motility Regulating Functions	Motility-Swarming Regulator	swrC|yerP->PGPT0016195	3
Indirect Effects	Colonizin gPlant System	Colonization-Motility|Chemotaxis	Other Motility Regulating Functions	Motility-Swarming Regulator	ycdX->PGPT0016200	2
Indirect Effects	Colonizin gPlant System	Colonization-Motility|Chemotaxis	Other Motility Regulating Functions	Motility-Swarming Regulator	ycgR->PGPT0016210	1
Indirect Effects	Colonizin gPlant System	Colonization-Motility|Chemotaxis	Other Motility Regulating Functions	Motility-Temperature Dependent Regulation	hosA->PGPT0016185	1
Indirect Effects	Colonizin gPlant System	Colonization-Motility|Chemotaxis	Other Motility Regulating Functions	Other Motility Regulation	codY->PGPT0016270	1
Indirect Effects	Colonizin gPlant System	Colonization-Motility|Chemotaxis	Other Motility Regulating Functions	Other Motility Regulation	luxS->PGPT0016265	1
Indirect Effects	Colonizin gPlant System	Colonization-Motility|Chemotaxis	Other Motility Regulating Functions	Other Motility Regulation	sigM|rpoE->PGPT0015025	1
Indirect Effects	Colonizin gPlant System	Colonization-Plant Cell Wall|Membrane Degradation	Plant Degradative Glycosidases|Glycosyl Hydrolases	Plant Degradative GS|GH-Amylase	amyA|malS->PGPT0018575	3
Indirect Effects	Colonizin gPlant System	Colonization-Plant Cell Wall|Membrane Degradation	Plant Degradative Glycosidases|Glycosyl Hydrolases	Plant Degradative GS|GH-Amylase	Beta Amylase->PGPT0018580	1

Indirect Effects	ColonizingPlant System	Colonization-Plant Cell Wall|Membrane Degradation	Plant Degradative Glycosidases|Glycosyl Hydrolases	Plant Degradative GS|GH-Carrageenase	celF|licH|chbF->PGPT0019105	3
Indirect Effects	ColonizingPlant System	Colonization-Plant Cell Wall|Membrane Degradation	Plant Degradative Glycosidases|Glycosyl Hydrolases	Plant Degradative GS|GH-Fructofuranosidase	sacA->PGPT0018815	2
Indirect Effects	ColonizingPlant System	Colonization-Plant Cell Wall|Membrane Degradation	Plant Degradative Glycosidases|Glycosyl Hydrolases	Plant Degradative GS|GH-Fructosidase	fruA->PGPT0018745	1
Indirect Effects	ColonizingPlant System	Colonization-Plant Cell Wall|Membrane Degradation	Plant Degradative Glycosidases|Glycosyl Hydrolases	Plant Degradative GS|GH-Galactosidase	bgaB|lacA->PGPT0017815	1
Indirect Effects	ColonizingPlant System	Colonization-Plant Cell Wall|Membrane Degradation	Plant Degradative Glycosidases|Glycosyl Hydrolases	Plant Degradative GS|GH-Galactosidase	bglA->PGPT0019255	2
Indirect Effects	ColonizingPlant System	Colonization-Plant Cell Wall|Membrane Degradation	Plant Degradative Glycosidases|Glycosyl Hydrolases	Plant Degradative GS|GH-Galactosidase	lacZ->PGPT0017810	1
Indirect Effects	ColonizingPlant System	Colonization-Plant Cell Wall|Membrane Degradation	Plant Degradative Glycosidases|Glycosyl Hydrolases	Plant Degradative GS|GH-Galactosidase	melA->PGPT0017850	1
Indirect Effects	ColonizingPlant System	Colonization-Plant Cell Wall|Membrane Degradation	Plant Degradative Glycosidases|Glycosyl Hydrolases	Plant Degradative GS|GH-Galactosidase	rafA|galA->PGPT0018835	1
Indirect Effects	ColonizingPlant System	Colonization-Plant Cell Wall|Membrane Degradation	Plant Degradative Glycosidases|Glycosyl Hydrolases	Plant Degradative GS|GH-Hydrolase	yteR|yesR->PGPT0018255	1
Indirect Effects	ColonizingPlant System	Colonization-Plant Cell Wall|Membrane Degradation	Plant Degradative Glycosidases|Glycosyl Hydrolases	Plant Degradative GS|GH-Lysozyme	acm->PGPT0019160	3
Indirect Effects	ColonizingPlant	Colonization-Plant Cell Wall|Membrane	Plant Degradative Glycosidases|Glycosyl	Plant Degradative GS|GH-	treC->PGPT0014095	1

	System	Degradation	Hydrolases	Phosphotrehalase		
Indirect Effects	ColonizingPlant System	Colonization-Plant Cell Wall|Membrane Degradation	Plant Degradative Glycosidases|Glycosyl Hydrolases	Plant Degradative GS|GH-Pullulanase	pulA->PGPT0012195	2
Indirect Effects	ColonizingPlant System	Colonization-Plant Cell Wall|Membrane Degradation	Plant Lignin Degradation|Ligninases	Plant Lignin Degradation-Polyphenol Oxidase	yfiH->PGPT0019965	1
Indirect Effects	ColonizingPlant System	Colonization-Plant Cell Wall|Membrane Degradation	Plant Membrane Lipid Degradation	Plant Phospho-|Lipid Degradation	pldB->PGPT0023520	4
Indirect Effects	ColonizingPlant System	Colonization-Plant Cell Wall|Membrane Degradation	Plant Membrane Lipid Degradation	Plant Phospho-|Lipid Degradation	ysiA|fadR->PGPT0023505	9
Indirect Effects	ColonizingPlant System	Colonization-Plant Derived Substrate Usage	Plant Derived Acrobic Acid|Vitamin C Utilization	Plant Derived Vitamin C transport	ulaA|sgaT->PGPT0017105	1
Indirect Effects	ColonizingPlant System	Colonization-Plant Derived Substrate Usage	Plant Derived Acrobic Acid|Vitamin C Utilization	Plant Derived Vitamin C transport	ulaB|sgaB->PGPT0017110	1
Indirect Effects	ColonizingPlant System	Colonization-Plant Derived Substrate Usage	Plant Derived Acrobic Acid|Vitamin C Utilization	Plant Derived Vitamin C transport	ulaC|sgaA->PGPT0017115	1
Indirect Effects	ColonizingPlant System	Colonization-Plant Derived Substrate Usage	Plant Derived Acrobic Acid|Vitamin C Utilization	Plant Derived Vitamin C Utilization	araD|ulaF|sgaE|sgbE->PGPT0017420	1
Indirect Effects	ColonizingPlant System	Colonization-Plant Derived Substrate Usage	Plant Derived Acrobic Acid|Vitamin C Utilization	Plant Derived Vitamin C Utilization	ulaG->PGPT0019580	1
Indirect Effects	ColonizingPlant System	Colonization-Plant Derived Substrate Usage	Plant Derived Amine transport	Plant Derived Putrescine transport	puuP->PGPT0007795	4
Indirect	Colonizin	Colonization-Plant	Plant Derived Amine	Plant Derived	eutA->PGPT0000600	1

Effects	gPlant System	Derived Substrate Usage	Usage	EthanolAmine Degradation		
Indirect Effects	Colonizin gPlant System	Colonization-Plant Derived Substrate Usage	Plant Derived Amine Usage	Plant Derived EthanolAmine Degradation	eutB->PGPT0000605	1
Indirect Effects	Colonizin gPlant System	Colonization-Plant Derived Substrate Usage	Plant Derived Amine Usage	Plant Derived EthanolAmine Degradation	eutC->PGPT0000610	1
Indirect Effects	Colonizin gPlant System	Colonization-Plant Derived Substrate Usage	Plant Derived Amine Usage	Plant Derived Putrescine Degradation	aldH|dhaS->PGPT0006875	5
Indirect Effects	Colonizin gPlant System	Colonization-Plant Derived Substrate Usage	Plant Derived Amine Usage	Plant Derived Putrescine Degradation	aOfH->PGPT0007641	1
Indirect Effects	Colonizin gPlant System	Colonization-Plant Derived Substrate Usage	Plant Derived Amine Usage	Plant Derived Putrescine Degradation	patA1->PGPT0007155	1
Indirect Effects	Colonizin gPlant System	Colonization-Plant Derived Substrate Usage	Plant Derived Amine Usage	Plant Derived Putrescine Degradation	puuB|ordL->PGPT0007637	1
Indirect Effects	Colonizin gPlant System	Colonization-Plant Derived Substrate Usage	Plant Derived Amine Usage	Plant Derived Putrescine Degradation	puuR->PGPT0019781	3
Indirect Effects	Colonizin gPlant System	Colonization-Plant Derived Substrate Usage	Plant Derived Amino Acid Dependent Pathways	Plant Derived Opine Metabolism	iolD->PGPT0018485	2
Indirect Effects	Colonizin gPlant System	Colonization-Plant Derived Substrate Usage	Plant Derived Amino Acid Dependent Pathways	Plant Derived Opine Metabolism	iolH->PGPT0020845	1
Indirect Effects	Colonizin gPlant System	Colonization-Plant Derived Substrate Usage	Plant Derived Amino Acid Dependent Pathways	Plant Derived Opine Metabolism	iolI->PGPT0018495	1

Indirect Effects	ColonizingPlant System	Colonization-Plant Derived Substrate Usage	Plant Derived Amino Acid Dependent Pathways	Plant Derived Opine Metabolism	iolW->PGPT0018530	1
Indirect Effects	ColonizingPlant System	Colonization-Plant Derived Substrate Usage	Plant Derived Amino Acid Dependent Pathways	Plant Derived Opine Metabolism	mocC->PGPT0016785	3
Indirect Effects	ColonizingPlant System	Colonization-Plant Derived Substrate Usage	Plant Derived Amino Acid Dependent Pathways	Plant Derived Opine Metabolism	mocR->PGPT0016790	3
Indirect Effects	ColonizingPlant System	Colonization-Plant Derived Substrate Usage	Plant Derived Amino Acid Dependent Pathways	Plant Derived Opine Metabolism	Putative saccharOpine Dehydrogenase->PGPT0020900	4
Indirect Effects	ColonizingPlant System	Colonization-Plant Derived Substrate Usage	Plant Derived Amino Acid Dependent Pathways	Plant Derived Opine Metabolism	sOxa->PGPT0013515	2
Indirect Effects	ColonizingPlant System	Colonization-Plant Derived Substrate Usage	Plant Derived Amino Acid Dependent Pathways	Plant Derived Peptide Metabolism	Putative PROTEASE->PGPT0021005	2
Indirect Effects	ColonizingPlant System	Colonization-Plant Derived Substrate Usage	Plant Derived Amino Acid Dependent Pathways	Plant Derived Peptide Metabolism	TC POT->PGPT0021010	2
Indirect Effects	ColonizingPlant System	Colonization-Plant Derived Substrate Usage	Plant Derived Amino Acid Dependent Pathways	Plant Derived Peptide Metabolism	acdP->PGPT0020950	1
Indirect Effects	ColonizingPlant System	Colonization-Plant Derived Substrate Usage	Plant Derived Amino Acid Dependent Pathways	Plant Derived Peptide Metabolism	bpr->PGPT0020925	1
Indirect Effects	ColonizingPlant System	Colonization-Plant Derived Substrate Usage	Plant Derived Amino Acid Dependent Pathways	Plant Derived Peptide Metabolism	ddpA|ABC PE S->PGPT0004430	6
Indirect Effects	ColonizingPlant	Colonization-Plant Derived Substrate	Plant Derived Amino Acid Dependent	Plant Derived Peptide Metabolism	ddpB|appB->PGPT0004445	4

	System	Usage	Pathways			
Indirect Effects	ColonizingPlant System	Colonization-Plant Derived Substrate Usage	Plant Derived Amino Acid Dependent Pathways	Plant Derived Peptide Metabolism	ddpC|appC->PGPT0004450	4
Indirect Effects	ColonizingPlant System	Colonization-Plant Derived Substrate Usage	Plant Derived Amino Acid Dependent Pathways	Plant Derived Peptide Metabolism	ddpD->PGPT0004435	4
Indirect Effects	ColonizingPlant System	Colonization-Plant Derived Substrate Usage	Plant Derived Amino Acid Dependent Pathways	Plant Derived Peptide Metabolism	ddpF->PGPT0004440	4
Indirect Effects	ColonizingPlant System	Colonization-Plant Derived Substrate Usage	Plant Derived Amino Acid Dependent Pathways	Plant Derived Peptide Metabolism	dppA1->PGPT0020935	1
Indirect Effects	ColonizingPlant System	Colonization-Plant Derived Substrate Usage	Plant Derived Amino Acid Dependent Pathways	Plant Derived Peptide Metabolism	dppB1->PGPT0004530	1
Indirect Effects	ColonizingPlant System	Colonization-Plant Derived Substrate Usage	Plant Derived Amino Acid Dependent Pathways	Plant Derived Peptide Metabolism	dppC1->PGPT0004535	1
Indirect Effects	ColonizingPlant System	Colonization-Plant Derived Substrate Usage	Plant Derived Amino Acid Dependent Pathways	Plant Derived Peptide Metabolism	dppD1->PGPT0004540	1
Indirect Effects	ColonizingPlant System	Colonization-Plant Derived Substrate Usage	Plant Derived Amino Acid Dependent Pathways	Plant Derived Peptide Metabolism	dppE->PGPT0004525	1
Indirect Effects	ColonizingPlant System	Colonization-Plant Derived Substrate Usage	Plant Derived Amino Acid Dependent Pathways	Plant Derived Peptide Metabolism	map->PGPT0020010	3
Indirect Effects	ColonizingPlant System	Colonization-Plant Derived Substrate Usage	Plant Derived Amino Acid Dependent Pathways	Plant Derived Peptide Metabolism	nprM->PGPT0020995	1
Indirect	Colonizin	Colonization-Plant	Plant Derived Amino	Plant Derived Peptide	opaA|pepQ-	1

Effects	gPlant System	Derived Substrate Usage	Acid Dependent Pathways	Metabolism	>PGPT0006760	
Indirect Effects	Colonizin gPlant System	Colonization-Plant Derived Substrate Usage	Plant Derived Amino Acid Dependent Pathways	Plant Derived Peptide Metabolism	oppA|mppA->PGPT0021015	1
Indirect Effects	Colonizin gPlant System	Colonization-Plant Derived Substrate Usage	Plant Derived Amino Acid Dependent Pathways	Plant Derived Peptide Metabolism	oppB->PGPT0021020	1
Indirect Effects	Colonizin gPlant System	Colonization-Plant Derived Substrate Usage	Plant Derived Amino Acid Dependent Pathways	Plant Derived Peptide Metabolism	oppC->PGPT0021025	1
Indirect Effects	Colonizin gPlant System	Colonization-Plant Derived Substrate Usage	Plant Derived Amino Acid Dependent Pathways	Plant Derived Peptide Metabolism	oppD->PGPT0021030	1
Indirect Effects	Colonizin gPlant System	Colonization-Plant Derived Substrate Usage	Plant Derived Amino Acid Dependent Pathways	Plant Derived Peptide Metabolism	oppF->PGPT0021035	2
Indirect Effects	Colonizin gPlant System	Colonization-Plant Derived Substrate Usage	Plant Derived Amino Acid Dependent Pathways	Plant Derived Peptide Metabolism	pepD->PGPT0020955	2
Indirect Effects	Colonizin gPlant System	Colonization-Plant Derived Substrate Usage	Plant Derived Amino Acid Dependent Pathways	Plant Derived Peptide Metabolism	pepF|pepB->PGPT0020965	1
Indirect Effects	Colonizin gPlant System	Colonization-Plant Derived Substrate Usage	Plant Derived Amino Acid Dependent Pathways	Plant Derived Peptide Metabolism	pepP->PGPT0006770	1
Indirect Effects	Colonizin gPlant System	Colonization-Plant Derived Substrate Usage	Plant Derived Amino Acid Dependent Pathways	Plant Derived Peptide Metabolism	pepS|ampP|ampT->PGPT0020940	2
Indirect Effects	Colonizin gPlant System	Colonization-Plant Derived Substrate Usage	Plant Derived Amino Acid Dependent Pathways	Plant Derived Peptide Metabolism	pepT->PGPT0020915	1

<table>
<tr><td>Indirect Effects</td><td>ColonizingPlant System</td><td>Colonization-Plant Derived Substrate Usage</td><td>Plant Derived Amino Acid Dependent Pathways</td><td>Plant Derived Peptide Metabolism</td><td>vpr->PGPT0020990</td><td>2</td></tr>
<tr><td>Indirect Effects</td><td>ColonizingPlant System</td><td>Colonization-Plant Derived Substrate Usage</td><td>Plant Derived Amino Acid Dependent Pathways</td><td>Plant Derived Peptide Metabolism</td><td>ykfC->PGPT0020945</td><td>2</td></tr>
<tr><td>Indirect Effects</td><td>ColonizingPlant System</td><td>Colonization-Plant Derived Substrate Usage</td><td>Plant Derived Amino Acid Dependent Pathways</td><td>Plant Derived Peptide Metabolism</td><td>ywaD->PGPT0020920</td><td>1</td></tr>
<tr><td>Indirect Effects</td><td>ColonizingPlant System</td><td>Colonization-Plant Derived Substrate Usage</td><td>Plant Derived Amino Acid transport</td><td>Plant Derived Alanine transport</td><td>cycA|ydgF->PGPT0020615</td><td>1</td></tr>
<tr><td>Indirect Effects</td><td>ColonizingPlant System</td><td>Colonization-Plant Derived Substrate Usage</td><td>Plant Derived Amino Acid transport</td><td>Plant Derived Alanine transport</td><td>yflA|TC AGCS->PGPT0014405</td><td>5</td></tr>
<tr><td>Indirect Effects</td><td>ColonizingPlant System</td><td>Colonization-Plant Derived Substrate Usage</td><td>Plant Derived Amino Acid transport</td><td>Plant Derived Arginine transport</td><td>arcD|lysl|lysP->PGPT0020620</td><td>1</td></tr>
<tr><td>Indirect Effects</td><td>ColonizingPlant System</td><td>Colonization-Plant Derived Substrate Usage</td><td>Plant Derived Amino Acid transport</td><td>Plant Derived Arginine transport</td><td>artP|artI->PGPT0020560</td><td>1</td></tr>
<tr><td>Indirect Effects</td><td>ColonizingPlant System</td><td>Colonization-Plant Derived Substrate Usage</td><td>Plant Derived Amino Acid transport</td><td>Plant Derived Arginine transport</td><td>artQ->PGPT0020550</td><td>1</td></tr>
<tr><td>Indirect Effects</td><td>ColonizingPlant System</td><td>Colonization-Plant Derived Substrate Usage</td><td>Plant Derived Amino Acid transport</td><td>Plant Derived Arginine transport</td><td>artR|artM->PGPT0020555</td><td>1</td></tr>
<tr><td>Indirect Effects</td><td>ColonizingPlant System</td><td>Colonization-Plant Derived Substrate Usage</td><td>Plant Derived Amino Acid transport</td><td>Plant Derived Arginine transport</td><td>lysE|argO->PGPT0020651</td><td>6</td></tr>
<tr><td>Indirect Effects</td><td>ColonizingPlant</td><td>Colonization-Plant Derived Substrate</td><td>Plant Derived Amino Acid transport</td><td>Plant Derived Aspartate transport</td><td>dctA->PGPT0001450</td><td>2</td></tr>
</table>

	System	Usage				
Indirect Effects	Colonizin gPlant System	Colonization-Plant Derived Substrate Usage	Plant Derived Amino Acid transport	Plant Derived Aspartate transport	peb1B|glnP|glnM->PGPT0000705	2
Indirect Effects	Colonizin gPlant System	Colonization-Plant Derived Substrate Usage	Plant Derived Amino Acid transport	Plant Derived Aspartate transport	peb1C|glnQ->PGPT0000710	1
Indirect Effects	Colonizin gPlant System	Colonization-Plant Derived Substrate Usage	Plant Derived Amino Acid transport	Plant Derived Aspartate transport	yveA->PGPT0000711	5
Indirect Effects	Colonizin gPlant System	Colonization-Plant Derived Substrate Usage	Plant Derived Amino Acid transport	Plant Derived Branched-Chain Amino Acid transport	TC LIVCS|brnQ->PGPT0014385	3
Indirect Effects	Colonizin gPlant System	Colonization-Plant Derived Substrate Usage	Plant Derived Amino Acid transport	Plant Derived Branched-Chain Amino Acid transport	livF->PGPT0020785	1
Indirect Effects	Colonizin gPlant System	Colonization-Plant Derived Substrate Usage	Plant Derived Amino Acid transport	Plant Derived Branched-Chain Amino Acid transport	livG->PGPT0020780	1
Indirect Effects	Colonizin gPlant System	Colonization-Plant Derived Substrate Usage	Plant Derived Amino Acid transport	Plant Derived Branched-Chain Amino Acid transport	livH->PGPT0020770	1
Indirect Effects	Colonizin gPlant System	Colonization-Plant Derived Substrate Usage	Plant Derived Amino Acid transport	Plant Derived Branched-Chain Amino Acid transport	livK->PGPT0020765	1
Indirect Effects	Colonizin gPlant System	Colonization-Plant Derived Substrate Usage	Plant Derived Amino Acid transport	Plant Derived Branched-Chain Amino Acid transport	livM->PGPT0020775	1
Indirect Effects	Colonizin gPlant System	Colonization-Plant Derived Substrate Usage	Plant Derived Amino Acid transport	Plant Derived Branched-Chain Amino Acid transport	ycsG->PGPT0020786	2
Indirect	Colonizin	Colonization-Plant	Plant Derived Amino	Plant Derived	fliY|tcyA|yckK-	1

Effects	gPlant System	Derived Substrate Usage	Acid transport	Cysteine transport	>PGPT0015635	
Indirect Effects	Colonizin gPlant System	Colonization-Plant Derived Substrate Usage	Plant Derived Amino Acid transport	Plant Derived Cysteine transport	tcyB|yecS->PGPT0020715	1
Indirect Effects	Colonizin gPlant System	Colonization-Plant Derived Substrate Usage	Plant Derived Amino Acid transport	Plant Derived Cysteine transport	tcyC|yecC->PGPT0020720	1
Indirect Effects	Colonizin gPlant System	Colonization-Plant Derived Substrate Usage	Plant Derived Amino Acid transport	Plant Derived Cysteine transport	tcyJ->PGPT0020725	1
Indirect Effects	Colonizin gPlant System	Colonization-Plant Derived Substrate Usage	Plant Derived Amino Acid transport	Plant Derived Cysteine transport	tcyK->PGPT0020730	2
Indirect Effects	Colonizin gPlant System	Colonization-Plant Derived Substrate Usage	Plant Derived Amino Acid transport	Plant Derived Cysteine transport	tcyL->PGPT0020735	2
Indirect Effects	Colonizin gPlant System	Colonization-Plant Derived Substrate Usage	Plant Derived Amino Acid transport	Plant Derived Cysteine transport	tcyM->PGPT0020740	2
Indirect Effects	Colonizin gPlant System	Colonization-Plant Derived Substrate Usage	Plant Derived Amino Acid transport	Plant Derived Cysteine transport	tcyN->PGPT0020745	2
Indirect Effects	Colonizin gPlant System	Colonization-Plant Derived Substrate Usage	Plant Derived Amino Acid transport	Plant Derived General Amino Acid transport	TC AAT|yifK->PGPT0000815	9
Indirect Effects	Colonizin gPlant System	Colonization-Plant Derived Substrate Usage	Plant Derived Amino Acid transport	Plant Derived General Amino Acid transport	TC APA|yhdG->PGPT0020810	4
Indirect Effects	Colonizin gPlant System	Colonization-Plant Derived Substrate Usage	Plant Derived Amino Acid transport	Plant Derived General Amino Acid transport	TC DAACS->PGPT0013965	2

Indirect Effects	ColonizingPlant System	Colonization-Plant Derived Substrate Usage	Plant Derived Amino Acid transport	Plant Derived General Amino Acid transport	ycgH->PGPT0020806	1
Indirect Effects	ColonizingPlant System	Colonization-Plant Derived Substrate Usage	Plant Derived Amino Acid transport	Plant Derived General Amino Acid transport	yuiF->PGPT0020805	1
Indirect Effects	ColonizingPlant System	Colonization-Plant Derived Substrate Usage	Plant Derived Amino Acid transport	Plant Derived Glutamate transport	gltS->PGPT0000810	1
Indirect Effects	ColonizingPlant System	Colonization-Plant Derived Substrate Usage	Plant Derived Amino Acid transport	Plant Derived Glutamate transport	ntrA|rpoN->PGPT0000795	2
Indirect Effects	ColonizingPlant System	Colonization-Plant Derived Substrate Usage	Plant Derived Amino Acid transport	Plant Derived GlutAmine transport	glnT->PGPT0000745	2
Indirect Effects	ColonizingPlant System	Colonization-Plant Derived Substrate Usage	Plant Derived Amino Acid transport	Plant Derived GlutAmine transport	peb1A|glnH->PGPT0000670	1
Indirect Effects	ColonizingPlant System	Colonization-Plant Derived Substrate Usage	Plant Derived Amino Acid transport	Plant Derived GlutAmine transport	peb1B|glnP|glnM->PGPT0000705	2
Indirect Effects	ColonizingPlant System	Colonization-Plant Derived Substrate Usage	Plant Derived Amino Acid transport	Plant Derived GlutAmine transport	peb1C|glnQ->PGPT0000710	1
Indirect Effects	ColonizingPlant System	Colonization-Plant Derived Substrate Usage	Plant Derived Amino Acid transport	Plant Derived GlutAmine transport	ycbA|glnK->PGPT0000735	2
Indirect Effects	ColonizingPlant System	Colonization-Plant Derived Substrate Usage	Plant Derived Amino Acid transport	Plant Derived GlutAmine transport	ycbB|glnL->PGPT0000740	2
Indirect Effects	ColonizingPlant	Colonization-Plant Derived Substrate	Plant Derived Amino Acid transport	Plant Derived Glycine transport	cycA|ydgF->PGPT0020615	1

	System	Usage				
Indirect Effects	ColonizingPlant System	Colonization-Plant Derived Substrate Usage	Plant Derived Amino Acid transport	Plant Derived Glycine transport	yflA|TC AGCS->PGPT0014405	5
Indirect Effects	ColonizingPlant System	Colonization-Plant Derived Substrate Usage	Plant Derived Amino Acid transport	Plant Derived Histidine transport	artP|artI->PGPT0020560	1
Indirect Effects	ColonizingPlant System	Colonization-Plant Derived Substrate Usage	Plant Derived Amino Acid transport	Plant Derived Histidine transport	artQ->PGPT0020550	1
Indirect Effects	ColonizingPlant System	Colonization-Plant Derived Substrate Usage	Plant Derived Amino Acid transport	Plant Derived Histidine transport	artR|artM->PGPT0020555	1
Indirect Effects	ColonizingPlant System	Colonization-Plant Derived Substrate Usage	Plant Derived Amino Acid transport	Plant Derived Histidine transport	hutM->PGPT0020590	1
Indirect Effects	ColonizingPlant System	Colonization-Plant Derived Substrate Usage	Plant Derived Amino Acid transport	Plant Derived Lysine transport	arcD|lysI|lysP->PGPT0020620	1
Indirect Effects	ColonizingPlant System	Colonization-Plant Derived Substrate Usage	Plant Derived Amino Acid transport	Plant Derived Lysine transport	artP|artI->PGPT0020560	1
Indirect Effects	ColonizingPlant System	Colonization-Plant Derived Substrate Usage	Plant Derived Amino Acid transport	Plant Derived Lysine transport	artQ->PGPT0020550	1
Indirect Effects	ColonizingPlant System	Colonization-Plant Derived Substrate Usage	Plant Derived Amino Acid transport	Plant Derived Lysine transport	artR|artM->PGPT0020555	1
Indirect Effects	ColonizingPlant System	Colonization-Plant Derived Substrate Usage	Plant Derived Amino Acid transport	Plant Derived Lysine transport	lysE|argO->PGPT0020651	6
Indirect	Colonizin	Colonization-Plant	Plant Derived Amino	Plant Derived Lysine	lysP->PGPT0020630	2

Effects	gPlant System	Derived Substrate Usage	Acid transport	transport		
Indirect Effects	Colonizin gPlant System	Colonization-Plant Derived Substrate Usage	Plant Derived Amino Acid transport	Plant Derived Methionine transport	metI->PGPT0020515	1
Indirect Effects	Colonizin gPlant System	Colonization-Plant Derived Substrate Usage	Plant Derived Amino Acid transport	Plant Derived Methionine transport	metN->PGPT0020520	1
Indirect Effects	Colonizin gPlant System	Colonization-Plant Derived Substrate Usage	Plant Derived Amino Acid transport	Plant Derived Methionine transport	metQ->PGPT0020510	1
Indirect Effects	Colonizin gPlant System	Colonization-Plant Derived Substrate Usage	Plant Derived Amino Acid transport	Plant Derived Methionine transport	mmuP->PGPT0020525	2
Indirect Effects	Colonizin gPlant System	Colonization-Plant Derived Substrate Usage	Plant Derived Amino Acid transport	Plant Derived Polar Amino Acid transport	artP|artI->PGPT0020560	1
Indirect Effects	Colonizin gPlant System	Colonization-Plant Derived Substrate Usage	Plant Derived Amino Acid transport	Plant Derived Polar Amino Acid transport	artQ->PGPT0020550	1
Indirect Effects	Colonizin gPlant System	Colonization-Plant Derived Substrate Usage	Plant Derived Amino Acid transport	Plant Derived Polar Amino Acid transport	artR|artM->PGPT0020555	1
Indirect Effects	Colonizin gPlant System	Colonization-Plant Derived Substrate Usage	Plant Derived Amino Acid transport	Plant Derived Proline transport	TC SSS|yerK|opuE->PGPT0013955	7
Indirect Effects	Colonizin gPlant System	Colonization-Plant Derived Substrate Usage	Plant Derived Amino Acid transport	Plant Derived Proline transport	gbuA|proV->PGPT0013630	2
Indirect Effects	Colonizin gPlant System	Colonization-Plant Derived Substrate Usage	Plant Derived Amino Acid transport	Plant Derived Proline transport	gbuB|proW->PGPT0013635	2

Indirect Effects	ColonizingPlant System	Colonization-Plant Derived Substrate Usage	Plant Derived Amino Acid transport	Plant Derived Proline transport	gbuC|proX->PGPT0013640	2
Indirect Effects	ColonizingPlant System	Colonization-Plant Derived Substrate Usage	Plant Derived Amino Acid transport	Plant Derived Proline transport	proP->PGPT0013645	1
Indirect Effects	ColonizingPlant System	Colonization-Plant Derived Substrate Usage	Plant Derived Amino Acid transport	Plant Derived Serine transport	cycA|ydgF->PGPT0020615	1
Indirect Effects	ColonizingPlant System	Colonization-Plant Derived Substrate Usage	Plant Derived Amino Acid Utilization	Plant Derived Alanine Degradation	ald->PGPT0020070	4
Indirect Effects	ColonizingPlant System	Colonization-Plant Derived Substrate Usage	Plant Derived Amino Acid Utilization	Plant Derived Alanine Degradation	alr->PGPT0020040	1
Indirect Effects	ColonizingPlant System	Colonization-Plant Derived Substrate Usage	Plant Derived Amino Acid Utilization	Plant Derived Alanine Degradation	dat->PGPT0001915	1
Indirect Effects	ColonizingPlant System	Colonization-Plant Derived Substrate Usage	Plant Derived Amino Acid Utilization	Plant Derived Alanine Degradation	ddl->PGPT0020050	2
Indirect Effects	ColonizingPlant System	Colonization-Plant Derived Substrate Usage	Plant Derived Amino Acid Utilization	Plant Derived Alanine Degradation	ycjG->PGPT0020045	1
Indirect Effects	ColonizingPlant System	Colonization-Plant Derived Substrate Usage	Plant Derived Amino Acid Utilization	Plant Derived Arginine Degradation	ahrC->PGPT0020105	1
Indirect Effects	ColonizingPlant System	Colonization-Plant Derived Substrate Usage	Plant Derived Amino Acid Utilization	Plant Derived Arginine Degradation	arcB|argF|argI->PGPT0020080	1
Indirect Effects	ColonizingPlant	Colonization-Plant Derived Substrate	Plant Derived Amino Acid Utilization	Plant Derived Arginine Degradation	nos|nosA->PGPT0020095	1

	System	Usage				
Indirect Effects	ColonizingPlant System	Colonization-Plant Derived Substrate Usage	Plant Derived Amino Acid Utilization	Plant Derived Arginine Degradation	rocF->PGPT0020100	2
Indirect Effects	ColonizingPlant System	Colonization-Plant Derived Substrate Usage	Plant Derived Amino Acid Utilization	Plant Derived Asparagine Degradation	EC 3 5 1 1|ansA|ansB->PGPT0020180	3
Indirect Effects	ColonizingPlant System	Colonization-Plant Derived Substrate Usage	Plant Derived Amino Acid Utilization	Plant Derived Asparagine Degradation	asnB->PGPT0020150	1
Indirect Effects	ColonizingPlant System	Colonization-Plant Derived Substrate Usage	Plant Derived Amino Acid Utilization	Plant Derived Aspartate Degradation	argG->PGPT0020130	1
Indirect Effects	ColonizingPlant System	Colonization-Plant Derived Substrate Usage	Plant Derived Amino Acid Utilization	Plant Derived Aspartate Degradation	asnB->PGPT0020150	1
Indirect Effects	ColonizingPlant System	Colonization-Plant Derived Substrate Usage	Plant Derived Amino Acid Utilization	Plant Derived Aspartate Degradation	aspA->PGPT0020125	2
Indirect Effects	ColonizingPlant System	Colonization-Plant Derived Substrate Usage	Plant Derived Amino Acid Utilization	Plant Derived Aspartate Degradation	aspB->PGPT0020110	1
Indirect Effects	ColonizingPlant System	Colonization-Plant Derived Substrate Usage	Plant Derived Amino Acid Utilization	Plant Derived Aspartate Degradation	hom->PGPT0020155	1
Indirect Effects	ColonizingPlant System	Colonization-Plant Derived Substrate Usage	Plant Derived Amino Acid Utilization	Plant Derived Aspartate Degradation	lysC->PGPT0014040	3
Indirect Effects	ColonizingPlant System	Colonization-Plant Derived Substrate Usage	Plant Derived Amino Acid Utilization	Plant Derived Aspartate Degradation	nadB->PGPT0013355	1
Indirect	Colonizin	Colonization-Plant	Plant Derived Amino	Plant Derived	panD->PGPT0008890	1

Effects	gPlant System	Derived Substrate Usage	Acid Utilization	Aspartate Degradation		
Indirect Effects	Colonizin gPlant System	Colonization-Plant Derived Substrate Usage	Plant Derived Amino Acid Utilization	Plant Derived Aspartate Degradation	purA->PGPT0020140	1
Indirect Effects	Colonizin gPlant System	Colonization-Plant Derived Substrate Usage	Plant Derived Amino Acid Utilization	Plant Derived Cysteine Degradation|Conversi on	aspB->PGPT0020110	1
Indirect Effects	Colonizin gPlant System	Colonization-Plant Derived Substrate Usage	Plant Derived Amino Acid Utilization	Plant Derived Cysteine Degradation|Conversi on	cysK->PGPT0002810	3
Indirect Effects	Colonizin gPlant System	Colonization-Plant Derived Substrate Usage	Plant Derived Amino Acid Utilization	Plant Derived Cysteine Degradation|Conversi on	cysK2->PGPT0002815	1
Indirect Effects	Colonizin gPlant System	Colonization-Plant Derived Substrate Usage	Plant Derived Amino Acid Utilization	Plant Derived Cysteine Degradation|Conversi on	mccB->PGPT0001985	2
Indirect Effects	Colonizin gPlant System	Colonization-Plant Derived Substrate Usage	Plant Derived Amino Acid Utilization	Plant Derived Cysteine Degradation|Conversi on	metC->PGPT0001995	2
Indirect Effects	Colonizin gPlant System	Colonization-Plant Derived Substrate Usage	Plant Derived Amino Acid Utilization	Plant Derived Cysteine Degradation|Conversi on	nifS|iscS->PGPT0000065	4
Indirect Effects	Colonizin gPlant System	Colonization-Plant Derived Substrate Usage	Plant Derived Amino Acid Utilization	Plant Derived Cysteine Degradation|Conversi	patB|malY->PGPT0001990	1

				on		
Indirect Effects	ColonizingPlant System	Colonization-Plant Derived Substrate Usage	Plant Derived Amino Acid Utilization	Plant Derived Cysteine Degradation|Conversion	sseA->PGPT0002045	2
Indirect Effects	ColonizingPlant System	Colonization-Plant Derived Substrate Usage	Plant Derived Amino Acid Utilization	Plant Derived Cysteine Degradation|Conversion	sufS->PGPT0020195	1
Indirect Effects	ColonizingPlant System	Colonization-Plant Derived Substrate Usage	Plant Derived Amino Acid Utilization	Plant Derived D-Amino Acid Degradation	dadA->PGPT0020315	1
Indirect Effects	ColonizingPlant System	Colonization-Plant Derived Substrate Usage	Plant Derived Amino Acid Utilization	Plant Derived Glutamate Degradation|Conversion	gadA|gadB->PGPT0007630	1
Indirect Effects	ColonizingPlant System	Colonization-Plant Derived Substrate Usage	Plant Derived Amino Acid Utilization	Plant Derived Glutamate Degradation|Conversion	glnA->PGPT0000645	1
Indirect Effects	ColonizingPlant System	Colonization-Plant Derived Substrate Usage	Plant Derived Amino Acid Utilization	Plant Derived Glutamate Degradation|Conversion	gudB|rocG->PGPT0020205	3
Indirect Effects	ColonizingPlant System	Colonization-Plant Derived Substrate Usage	Plant Derived Amino Acid Utilization	Plant Derived Glutamate Degradation|Conversion	murI->PGPT0020200	1
Indirect Effects	ColonizingPlant System	Colonization-Plant Derived Substrate Usage	Plant Derived Amino Acid Utilization	Plant Derived Glutamate Degradation|Conversion	putA->PGPT0020210	4

Indirect Effects	Colonizin gPlant System	Colonization-Plant Derived Substrate Usage	Plant Derived Amino Acid Utilization	Plant Derived GlutAmine Degradation	glmS|nodM->PGPT0017630	1
Indirect Effects	Colonizin gPlant System	Colonization-Plant Derived Substrate Usage	Plant Derived Amino Acid Utilization	Plant Derived GlutAmine Degradation	glsA->PGPT0020215	1
Indirect Effects	Colonizin gPlant System	Colonization-Plant Derived Substrate Usage	Plant Derived Amino Acid Utilization	Plant Derived GlutAmine Degradation	gltB->PGPT0000635	1
Indirect Effects	Colonizin gPlant System	Colonization-Plant Derived Substrate Usage	Plant Derived Amino Acid Utilization	Plant Derived GlutAmine Degradation	gltD->PGPT0000640	1
Indirect Effects	Colonizin gPlant System	Colonization-Plant Derived Substrate Usage	Plant Derived Amino Acid Utilization	Plant Derived GlutAmine Degradation	purF->PGPT0020225	1
Indirect Effects	Colonizin gPlant System	Colonization-Plant Derived Substrate Usage	Plant Derived Amino Acid Utilization	Plant Derived GlutAmine Degradation	purQ->PGPT0020220	1
Indirect Effects	Colonizin gPlant System	Colonization-Plant Derived Substrate Usage	Plant Derived Amino Acid Utilization	Plant Derived GlutAmine Degradation	ycbA|glnK->PGPT0000735	2
Indirect Effects	Colonizin gPlant System	Colonization-Plant Derived Substrate Usage	Plant Derived Amino Acid Utilization	Plant Derived GlutAmine Degradation	ycbB|glnL->PGPT0000740	2
Indirect Effects	Colonizin gPlant System	Colonization-Plant Derived Substrate Usage	Plant Derived Amino Acid Utilization	Plant Derived Glycine Degradation	gcvPA->PGPT0020485	1
Indirect Effects	Colonizin gPlant System	Colonization-Plant Derived Substrate Usage	Plant Derived Amino Acid Utilization	Plant Derived Glycine Degradation	gcvPB->PGPT0020490	1
Indirect Effects	Colonizin gPlant	Colonization-Plant Derived Substrate	Plant Derived Amino Acid Utilization	Plant Derived Glycine Degradation	gcvT->PGPT0008130	1

	System	Usage				
Indirect Effects	ColonizingPlant System	Colonization-Plant Derived Substrate Usage	Plant Derived Amino Acid Utilization	Plant Derived Glycine Degradation	glyA->PGPT0008090	1
Indirect Effects	ColonizingPlant System	Colonization-Plant Derived Substrate Usage	Plant Derived Amino Acid Utilization	Plant Derived Glycine Degradation	kbl->PGPT0020495	1
Indirect Effects	ColonizingPlant System	Colonization-Plant Derived Substrate Usage	Plant Derived Amino Acid Utilization	Plant Derived Glycine Degradation	thiG->PGPT0008965	1
Indirect Effects	ColonizingPlant System	Colonization-Plant Derived Substrate Usage	Plant Derived Amino Acid Utilization	Plant Derived Glycine Degradation	thiO->PGPT0008955	2
Indirect Effects	ColonizingPlant System	Colonization-Plant Derived Substrate Usage	Plant Derived Amino Acid Utilization	Plant Derived Histidine Degradation	hutG->PGPT0020240	2
Indirect Effects	ColonizingPlant System	Colonization-Plant Derived Substrate Usage	Plant Derived Amino Acid Utilization	Plant Derived Histidine Degradation	hutH->PGPT0020230	2
Indirect Effects	ColonizingPlant System	Colonization-Plant Derived Substrate Usage	Plant Derived Amino Acid Utilization	Plant Derived Histidine Degradation	hutI->PGPT0020235	4
Indirect Effects	ColonizingPlant System	Colonization-Plant Derived Substrate Usage	Plant Derived Amino Acid Utilization	Plant Derived Histidine Degradation	hutU->PGPT0020250	2
Indirect Effects	ColonizingPlant System	Colonization-Plant Derived Substrate Usage	Plant Derived Amino Acid Utilization	Plant Derived Leucine Degradation	bkdA1->PGPT0008861	1
Indirect Effects	ColonizingPlant System	Colonization-Plant Derived Substrate Usage	Plant Derived Amino Acid Utilization	Plant Derived Leucine Degradation	bkdA2|bfmBAB->PGPT0008862	1
Indirect	Colonizin	Colonization-Plant	Plant Derived Amino	Plant Derived Leucine	ilvE->PGPT0008860	1

Effects	gPlant System	Derived Substrate Usage	Acid Utilization	Degradation		
Indirect Effects	Colonizin gPlant System	Colonization-Plant Derived Substrate Usage	Plant Derived Amino Acid Utilization	Plant Derived Leucine Degradation	ldh|leu->PGPT0020320	1
Indirect Effects	Colonizin gPlant System	Colonization-Plant Derived Substrate Usage	Plant Derived Amino Acid Utilization	Plant Derived Lysine Degradation	ablA->PGPT0014370	2
Indirect Effects	Colonizin gPlant System	Colonization-Plant Derived Substrate Usage	Plant Derived Amino Acid Utilization	Plant Derived Lysine Degradation	dat->PGPT0001915	1
Indirect Effects	Colonizin gPlant System	Colonization-Plant Derived Substrate Usage	Plant Derived Amino Acid Utilization	Plant Derived Methionine Degradation	map->PGPT0020010	3
Indirect Effects	Colonizin gPlant System	Colonization-Plant Derived Substrate Usage	Plant Derived Amino Acid Utilization	Plant Derived Methionine Degradation	mdeA->PGPT0020020	2
Indirect Effects	Colonizin gPlant System	Colonization-Plant Derived Substrate Usage	Plant Derived Amino Acid Utilization	Plant Derived Methionine Degradation	metK->PGPT0020000	1
Indirect Effects	Colonizin gPlant System	Colonization-Plant Derived Substrate Usage	Plant Derived Amino Acid Utilization	Plant Derived Methionine Degradation	ridA|tdcF->PGPT0020030	1
Indirect Effects	Colonizin gPlant System	Colonization-Plant Derived Substrate Usage	Plant Derived Amino Acid Utilization	Plant Derived Phenylalanine Degradation	aaaT->PGPT0020290	7
Indirect Effects	Colonizin gPlant System	Colonization-Plant Derived Substrate Usage	Plant Derived Amino Acid Utilization	Plant Derived Phenylalanine Degradation	aspB->PGPT0020110	1
Indirect Effects	Colonizin gPlant System	Colonization-Plant Derived Substrate Usage	Plant Derived Amino Acid Utilization	Plant Derived Phenylalanine Degradation	dadA->PGPT0020315	1

Indirect Effects	ColonizingPlant System	Colonization-Plant Derived Substrate Usage	Plant Derived Amino Acid Utilization	Plant Derived Phenylalanine Degradation	dat->PGPT0001915	1
Indirect Effects	ColonizingPlant System	Colonization-Plant Derived Substrate Usage	Plant Derived Amino Acid Utilization	Plant Derived Phenylalanine Degradation	hisC->PGPT0020305	1
Indirect Effects	ColonizingPlant System	Colonization-Plant Derived Substrate Usage	Plant Derived Amino Acid Utilization	Plant Derived Phenylalanine Degradation	ilvE->PGPT0008860	1
Indirect Effects	ColonizingPlant System	Colonization-Plant Derived Substrate Usage	Plant Derived Amino Acid Utilization	Plant Derived Phenylalanine Degradation	tyrB->PGPT0020310	2
Indirect Effects	ColonizingPlant System	Colonization-Plant Derived Substrate Usage	Plant Derived Amino Acid Utilization	Plant Derived Proline Degradation	P4HA Like->PGPT0020375	1
Indirect Effects	ColonizingPlant System	Colonization-Plant Derived Substrate Usage	Plant Derived Amino Acid Utilization	Plant Derived Proline Degradation	lhpB2->PGPT0020390	1
Indirect Effects	ColonizingPlant System	Colonization-Plant Derived Substrate Usage	Plant Derived Amino Acid Utilization	Plant Derived Proline Degradation	prdF->PGPT0014240	1
Indirect Effects	ColonizingPlant System	Colonization-Plant Derived Substrate Usage	Plant Derived Amino Acid Utilization	Plant Derived Proline Degradation	proC->PGPT0014225	3
Indirect Effects	ColonizingPlant System	Colonization-Plant Derived Substrate Usage	Plant Derived Amino Acid Utilization	Plant Derived Proline Degradation	putA->PGPT0020210	4
Indirect Effects	ColonizingPlant System	Colonization-Plant Derived Substrate Usage	Plant Derived Amino Acid Utilization	Plant Derived Serine Degradation	cysE->PGPT0002970	1
Indirect Effects	ColonizingPlant	Colonization-Plant Derived Substrate	Plant Derived Amino Acid Utilization	Plant Derived Serine Degradation	cysE->PGPT0020265	1

	System	Usage				
Indirect Effects	Colonizin gPlant System	Colonization-Plant Derived Substrate Usage	Plant Derived Amino Acid Utilization	Plant Derived Serine Degradation	dsdA->PGPT0001970	1
Indirect Effects	Colonizin gPlant System	Colonization-Plant Derived Substrate Usage	Plant Derived Amino Acid Utilization	Plant Derived Serine Degradation	hom->PGPT0020155	1
Indirect Effects	Colonizin gPlant System	Colonization-Plant Derived Substrate Usage	Plant Derived Amino Acid Utilization	Plant Derived Serine Degradation	thrB->PGPT0020275	1
Indirect Effects	Colonizin gPlant System	Colonization-Plant Derived Substrate Usage	Plant Derived Amino Acid Utilization	Plant Derived Serine Degradation	thrC->PGPT0009175	5
Indirect Effects	Colonizin gPlant System	Colonization-Plant Derived Substrate Usage	Plant Derived Amino Acid Utilization	Plant Derived Threonine Degradation	ilvA|tdcB->PGPT0001960	1
Indirect Effects	Colonizin gPlant System	Colonization-Plant Derived Substrate Usage	Plant Derived Amino Acid Utilization	Plant Derived Tyrosine Degradation	aspB->PGPT0020110	1
Indirect Effects	Colonizin gPlant System	Colonization-Plant Derived Substrate Usage	Plant Derived Amino Acid Utilization	Plant Derived Tyrosine Degradation	hisC->PGPT0020305	1
Indirect Effects	Colonizin gPlant System	Colonization-Plant Derived Substrate Usage	Plant Derived Amino Acid Utilization	Plant Derived Tyrosine Degradation	hpaB->PGPT0020455	2
Indirect Effects	Colonizin gPlant System	Colonization-Plant Derived Substrate Usage	Plant Derived Amino Acid Utilization	Plant Derived Tyrosine Degradation	tyr->PGPT0020425	1
Indirect Effects	Colonizin gPlant System	Colonization-Plant Derived Substrate Usage	Plant Derived Amino Acid Utilization	Plant Derived Tyrosine Degradation	tyrB->PGPT0020310	2
Indirect	Colonizin	Colonization-Plant	Plant Derived Amino	Plant Derived Valine	actA->PGPT0001885	1

Effects	gPlant System	Derived Substrate Usage	Acid Utilization	Degradation		
Indirect Effects	Colonizin gPlant System	Colonization-Plant Derived Substrate Usage	Plant Derived Amino Acid Utilization	Plant Derived Valine Degradation	ilvE->PGPT0008860	1
Indirect Effects	Colonizin gPlant System	Colonization-Plant Derived Substrate Usage	Plant Derived Amino|Nucleotide Sugar Utilization	Plant Derived Fructoselysine Biosynthesis	frlB|yurP->PGPT0018970	1
Indirect Effects	Colonizin gPlant System	Colonization-Plant Derived Substrate Usage	Plant Derived Amino|Nucleotide Sugar Utilization	Plant Derived Fructoselysine Biosynthesis	frlD->PGPT0018980	1
Indirect Effects	Colonizin gPlant System	Colonization-Plant Derived Substrate Usage	Plant Derived Amino|Nucleotide Sugar Utilization	Plant Derived Glucosamine|Glucosaminate Degradation	glmM->PGPT0018950	1
Indirect Effects	Colonizin gPlant System	Colonization-Plant Derived Substrate Usage	Plant Derived Amino|Nucleotide Sugar Utilization	Plant Derived Glucosamine|Glucosaminate Degradation	glmU->PGPT0018955	1
Indirect Effects	Colonizin gPlant System	Colonization-Plant Derived Substrate Usage	Plant Derived Amino|Nucleotide Sugar Utilization	Plant Derived Glucosamine|Glucosaminate Degradation	gspK->PGPT0018875	1
Indirect Effects	Colonizin gPlant System	Colonization-Plant Derived Substrate Usage	Plant Derived Amino|Nucleotide Sugar Utilization	Plant Derived Glucosamine|Glucosaminate Degradation	nagA->PGPT0018860	1
Indirect Effects	Colonizin gPlant System	Colonization-Plant Derived Substrate Usage	Plant Derived Amino|Nucleotide Sugar Utilization	Plant Derived Glucosamine|Glucosaminate Degradation	nagB->PGPT0018865	1
Indirect Effects	Colonizin gPlant System	Colonization-Plant Derived Substrate Usage	Plant Derived Amino|Nucleotide Sugar Utilization	Plant Derived Glucosamine|Glucosaminate Degradation	nagC->PGPT0018870	2
Indirect Effects	Colonizin gPlant System	Colonization-Plant Derived Substrate Usage	Plant Derived Amino|Nucleotide Sugar Utilization	Plant Derived Glucosamine|Glucosaminate Degradation	pseB|wbjB->PGPT0018935	1

Indirect Effects	ColonizingPlant System	Colonization-Plant Derived Substrate Usage	Plant Derived Amino|Nucleotide Sugar Utilization	Plant Derived Glucosamine|Glucosaminate Degradation	wbpA->PGPT0018920	1
Indirect Effects	ColonizingPlant System	Colonization-Plant Derived Substrate Usage	Plant Derived Amino|Nucleotide Sugar Utilization	Plant Derived Glucosamine|Glucosaminate Degradation	wecB->PGPT0018905	2
Indirect Effects	ColonizingPlant System	Colonization-Plant Derived Substrate Usage	Plant Derived Amino|Nucleotide Sugar Utilization	Plant Derived Muramate Degradation	mupP->PGPT0019045	1
Indirect Effects	ColonizingPlant System	Colonization-Plant Derived Substrate Usage	Plant Derived Amino|Nucleotide Sugar Utilization	Plant Derived Muramate Degradation	murQ->PGPT0019040	1
Indirect Effects	ColonizingPlant System	Colonization-Plant Derived Substrate Usage	Plant Derived Amino|Nucleotide Sugar Utilization	Plant Derived UDP-Galactose-Glucose Pool	galT->PGPT0017835	1
Indirect Effects	ColonizingPlant System	Colonization-Plant Derived Substrate Usage	Plant Derived Amino|Nucleotide Sugar Utilization	Plant Derived UDP-Galactose-Glucuronate Pool	cap1J|wbgU->PGPT0019020	1
Indirect Effects	ColonizingPlant System	Colonization-Plant Derived Substrate Usage	Plant Derived Amino|Nucleotide Sugar Utilization	Plant Derived UDP-Galactose-xylose Pool	uxs->PGPT0019015	1
Indirect Effects	ColonizingPlant System	Colonization-Plant Derived Substrate Usage	Plant Derived Aromatic|PhenolIC Compound Utilization	Plant Derived Benzene|Phenol Degradation	bsdA->PGPT0019770	4
Indirect Effects	ColonizingPlant System	Colonization-Plant Derived Substrate Usage	Plant Derived Aromatic|PhenolIC Compound Utilization	Plant Derived Benzene|Phenol Degradation	bsdC->PGPT0005435	1
Indirect Effects	ColonizingPlant System	Colonization-Plant Derived Substrate Usage	Plant Derived Aromatic|PhenolIC Compound Utilization	Plant Derived Benzene|Phenol Degradation	pdc->PGPT0019910	1
Indirect Effects	ColonizingPlant	Colonization-Plant Derived Substrate	Plant Derived Aromatic|PhenolIC	Plant Derived Benzene|Phenol	ubiX|bsdB->PGPT0009565	1

	System	Usage	Compound Utilization	Degradation		
Indirect Effects	Colonizin gPlant System	Colonization-Plant Derived Substrate Usage	Plant Derived Aromatic|PhenolIC Compound Utilization	Plant Derived Beta-Ketoadipate Pathway	pcaC->PGPT0005005	6
Indirect Effects	Colonizin gPlant System	Colonization-Plant Derived Substrate Usage	Plant Derived Aromatic|PhenolIC Compound Utilization	Plant Derived Beta-Ketoadipate Pathway	pcaD|catD->PGPT0004995	3
Indirect Effects	Colonizin gPlant System	Colonization-Plant Derived Substrate Usage	Plant Derived Aromatic|PhenolIC Compound Utilization	Plant Derived Catechol Derivate Utilization	catE->PGPT0005050	1
Indirect Effects	Colonizin gPlant System	Colonization-Plant Derived Substrate Usage	Plant Derived Aromatic|PhenolIC Compound Utilization	Plant Derived Catechol Derivate Utilization	mhpE->PGPT0002050	1
Indirect Effects	Colonizin gPlant System	Colonization-Plant Derived Substrate Usage	Plant Derived Aromatic|PhenolIC Compound Utilization	Plant Derived Gallate Catabolism	ligK|galC->PGPT0002085	5
Indirect Effects	Colonizin gPlant System	Colonization-Plant Derived Substrate Usage	Plant Derived Aromatic|PhenolIC Compound Utilization	Plant Derived HCA Degradation|Vanillin Intermediate	paaF|echA->PGPT0001860	1
Indirect Effects	Colonizin gPlant System	Colonization-Plant Derived Substrate Usage	Plant Derived Aromatic|PhenolIC Compound Utilization	Plant Derived Lignin Utilization	yfiH->PGPT0019965	1
Indirect Effects	Colonizin gPlant System	Colonization-Plant Derived Substrate Usage	Plant Derived Aromatic|PhenolIC Compound Utilization	Plant Derived Nitro-|Aminobenzoate Degradation	namA->PGPT0005575	1
Indirect Effects	Colonizin gPlant System	Colonization-Plant Derived Substrate Usage	Plant Derived Aromatic|PhenolIC Compound Utilization	Plant Derived Nitro-|Aminobenzoate Degradation	nbaA->PGPT0005560	1
Indirect Effects	Colonizin gPlant System	Colonization-Plant Derived Substrate Usage	Plant Derived Aromatic|PhenolIC Compound Utilization	Plant Derived Nitro-|Aminobenzoate Degradation	nhoA|yddI->PGPT0019890	1
Indirect	Colonizin	Colonization-Plant	Plant Derived	Plant Derived	padA->PGPT0019920	1

Effects	gPlant System	Derived Substrate Usage	Aromatic|PhenolIC Compound Utilization	PhenylAcetate Degradation		
Indirect Effects	Colonizin gPlant System	Colonization-Plant Derived Substrate Usage	Plant Derived Aromatic|PhenolIC Compound Utilization	Plant Derived Polyphenol Metabolism	yfiH->PGPT0019965	1
Indirect Effects	Colonizin gPlant System	Colonization-Plant Derived Substrate Usage	Plant Derived Aromatic|PhenolIC Compound Utilization	Plant Derived Protocatechuic Acid Degradation	pcaC->PGPT0005005	6
Indirect Effects	Colonizin gPlant System	Colonization-Plant Derived Substrate Usage	Plant Derived Aromatic|PhenolIC Compound Utilization	Plant Derived Protocatechuic Acid Degradation	pcaD|catD->PGPT0004995	3
Indirect Effects	Colonizin gPlant System	Colonization-Plant Derived Substrate Usage	Plant Derived Aromatic|PhenolIC Compound Utilization	Plant Derived Quinate Catabolism	aRod->PGPT0012885	1
Indirect Effects	Colonizin gPlant System	Colonization-Plant Derived Substrate Usage	Plant Derived Aromatic|PhenolIC Compound Utilization	Plant Derived Quinate Catabolism	aroQ|qutE->PGPT0012905	1
Indirect Effects	Colonizin gPlant System	Colonization-Plant Derived Substrate Usage	Plant Derived Aromatic|PhenolIC Compound Utilization	Plant Derived Quinate Catabolism	ydiB->PGPT0019725	1
Indirect Effects	Colonizin gPlant System	Colonization-Plant Derived Substrate Usage	Plant Derived Carbohydrate-General Usage	Plant Derived CarbohydrateS-Glycolysis|GluconeoGenesis	eno->PGPT0018050	1
Indirect Effects	Colonizin gPlant System	Colonization-Plant Derived Substrate Usage	Plant Derived Carbohydrate-General Usage	Plant Derived CarbohydrateS-Glycolysis|GluconeoGenesis	fbaA|cbbA->PGPT0017615	1
Indirect Effects	Colonizin gPlant System	Colonization-Plant Derived Substrate Usage	Plant Derived Carbohydrate-General Usage	Plant Derived CarbohydrateS-Glycolysis|GluconeoGenesis	fruK|fpk->PGPT0017580	1

Indirect Effects	ColonizingPlant System	Colonization-Plant Derived Substrate Usage	Plant Derived Carbohydrate-General Usage	Plant Derived CarbohydrateS-Glycolysis|GluconeoGenesis	gapA->PGPT0018000	2
Indirect Effects	ColonizingPlant System	Colonization-Plant Derived Substrate Usage	Plant Derived Carbohydrate-General Usage	Plant Derived CarbohydrateS-Glycolysis|GluconeoGenesis	gapN->PGPT0018005	2
Indirect Effects	ColonizingPlant System	Colonization-Plant Derived Substrate Usage	Plant Derived Carbohydrate-General Usage	Plant Derived CarbohydrateS-Glycolysis|GluconeoGenesis	gpmA->PGPT0018030	1
Indirect Effects	ColonizingPlant System	Colonization-Plant Derived Substrate Usage	Plant Derived Carbohydrate-General Usage	Plant Derived CarbohydrateS-Glycolysis|GluconeoGenesis	gpmI->PGPT0018040	1
Indirect Effects	ColonizingPlant System	Colonization-Plant Derived Substrate Usage	Plant Derived Carbohydrate-General Usage	Plant Derived CarbohydrateS-Glycolysis|GluconeoGenesis	pdhA->PGPT0018025	1
Indirect Effects	ColonizingPlant System	Colonization-Plant Derived Substrate Usage	Plant Derived Carbohydrate-General Usage	Plant Derived CarbohydrateS-Glycolysis|GluconeoGenesis	pgi->PGPT0017735	1
Indirect Effects	ColonizingPlant System	Colonization-Plant Derived Substrate Usage	Plant Derived Carbohydrate-General Usage	Plant Derived CarbohydrateS-Glycolysis|GluconeoGenesis	pgk->PGPT0018015	1
Indirect Effects	ColonizingPlant System	Colonization-Plant Derived Substrate Usage	Plant Derived Carbohydrate-General Usage	Plant Derived CarbohydrateS-Glycolysis|GluconeoGenesis	pgm->PGPT0017710	2

Indirect Effects	ColonizingPlant System	Colonization-Plant Derived Substrate Usage	Plant Derived Carbohydrate-General Usage	Plant Derived CarbohydrateS-Glycolysis|GluconeoGenesis	pps|ppsA->PGPT0002035	2
Indirect Effects	ColonizingPlant System	Colonization-Plant Derived Substrate Usage	Plant Derived Carbohydrate-General Usage	Plant Derived CarbohydrateS-Glycolysis|GluconeoGenesis	pyk->PGPT0002020	2
Indirect Effects	ColonizingPlant System	Colonization-Plant Derived Substrate Usage	Plant Derived Carbohydrate-General Usage	Plant Derived CarbohydrateS-Glycolysis|GluconeoGenesis	tpiA->PGPT0017995	1
Indirect Effects	ColonizingPlant System	Colonization-Plant Derived Substrate Usage	Plant Derived Carbohydrate-General Usage	Plant Derived Carbohydrate Utilization Regulation	hexR|yebK->PGPT0017985	4
Indirect Effects	ColonizingPlant System	Colonization-Plant Derived Substrate Usage	Plant Derived Carbohydrate-General Usage	Plant Derived Carbohydrate Utilization Regulation	lacI|galR->PGPT0017992	10
Indirect Effects	ColonizingPlant System	Colonization-Plant Derived Substrate Usage	Plant Derived Carbohydrate-Hexoses Utilization	Plant Derived Fructose Metabolism|Degradation	fbaA|cbbA->PGPT0017615	1
Indirect Effects	ColonizingPlant System	Colonization-Plant Derived Substrate Usage	Plant Derived Carbohydrate-Hexoses Utilization	Plant Derived Fructose Metabolism|Degradation	fbp3->PGPT0017660	1
Indirect Effects	ColonizingPlant System	Colonization-Plant Derived Substrate Usage	Plant Derived Carbohydrate-Hexoses Utilization	Plant Derived Fructose Metabolism|Degradation	fruK|fpk->PGPT0017580	1
Indirect Effects	ColonizingPlant System	Colonization-Plant Derived Substrate Usage	Plant Derived Carbohydrate-Hexoses Utilization	Plant Derived Fructose Metabolism|Degradation	fruR2->PGPT0017590	3
Indirect Effects	ColonizingPlant	Colonization-Plant Derived Substrate	Plant Derived Carbohydrate-Hexoses	Plant Derived Fructose Metabolism|Degradati	glmS|nodM->PGPT0017630	1

	System	Usage	Utilization	on		
Indirect Effects	ColonizingPlant System	Colonization-Plant Derived Substrate Usage	Plant Derived Carbohydrate-Hexoses Utilization	Plant Derived Fructose Metabolism|Degradation	scrK->PGPT0017625	3
Indirect Effects	ColonizingPlant System	Colonization-Plant Derived Substrate Usage	Plant Derived Carbohydrate-Hexoses Utilization	Plant Derived Fucose Degradation	fucA->PGPT0017430	2
Indirect Effects	ColonizingPlant System	Colonization-Plant Derived Substrate Usage	Plant Derived Carbohydrate-Hexoses Utilization	Plant Derived Fucose Degradation	pld->PGPT0017915	1
Indirect Effects	ColonizingPlant System	Colonization-Plant Derived Substrate Usage	Plant Derived Carbohydrate-Hexoses Utilization	Plant Derived Galacotose Metabolism|Degradation	galM->PGPT0017840	1
Indirect Effects	ColonizingPlant System	Colonization-Plant Derived Substrate Usage	Plant Derived Carbohydrate-Hexoses Utilization	Plant Derived Galacotose Metabolism|Degradation	galT->PGPT0017835	1
Indirect Effects	ColonizingPlant System	Colonization-Plant Derived Substrate Usage	Plant Derived Carbohydrate-Hexoses Utilization	Plant Derived Galacotose Metabolism|Degradation	melA->PGPT0017850	1
Indirect Effects	ColonizingPlant System	Colonization-Plant Derived Substrate Usage	Plant Derived Carbohydrate-Hexoses Utilization	Plant Derived Glucose Degradation	pgi->PGPT0017735	1
Indirect Effects	ColonizingPlant System	Colonization-Plant Derived Substrate Usage	Plant Derived Carbohydrate-Hexoses Utilization	Plant Derived Glucose Degradation	pgm->PGPT0017710	2
Indirect Effects	ColonizingPlant System	Colonization-Plant Derived Substrate Usage	Plant Derived Carbohydrate-Hexoses Utilization	Plant Derived Glucose Degradation	pgmB->PGPT0017720	1
Indirect	Colonizin	Colonization-Plant	Plant Derived	Plant Derived Glucose	ptsG|glcA|glcB-	1

Effects	gPlant System	Derived Substrate Usage	Carbohydrate-Hexoses Utilization	Degradation	>PGPT0016870	
Indirect Effects	Colonizin gPlant System	Colonization-Plant Derived Substrate Usage	Plant Derived Carbohydrate-Hexoses Utilization	Plant Derived Glucose Degradation	yidA->PGPT0017727	4
Indirect Effects	Colonizin gPlant System	Colonization-Plant Derived Substrate Usage	Plant Derived Carbohydrate-Hexoses Utilization	Plant Derived Glucose Degradation	yigL->PGPT0017726	1
Indirect Effects	Colonizin gPlant System	Colonization-Plant Derived Substrate Usage	Plant Derived Carbohydrate-Hexoses Utilization	Plant Derived Lactose Degradation	bgaB|lacA->PGPT0017815	1
Indirect Effects	Colonizin gPlant System	Colonization-Plant Derived Substrate Usage	Plant Derived Carbohydrate-Hexoses Utilization	Plant Derived Lactose Degradation	lacZ->PGPT0017810	1
Indirect Effects	Colonizin gPlant System	Colonization-Plant Derived Substrate Usage	Plant Derived Carbohydrate-Hexoses Utilization	Plant Derived Mannose Metabolism|Degradation	gmhB|mpg|gmpP|glmM|hddC|manB->PGPT0017870	1
Indirect Effects	Colonizin gPlant System	Colonization-Plant Derived Substrate Usage	Plant Derived Carbohydrate-Hexoses Utilization	Plant Derived Mannose Metabolism|Degradation	manA->PGPT0017860	1
Indirect Effects	Colonizin gPlant System	Colonization-Plant Derived Substrate Usage	Plant Derived Carbohydrate-Hexoses Utilization	Plant Derived Mannose Metabolism|Degradation	mgtA->PGPT0017895	1
Indirect Effects	Colonizin gPlant System	Colonization-Plant Derived Substrate Usage	Plant Derived Carbohydrate-Hexoses Utilization	Plant Derived Rhamnose Degradation	rhaS->PGPT0017780	1
Indirect Effects	Colonizin gPlant System	Colonization-Plant Derived Substrate Usage	Plant Derived Carbohydrate-Hexoses Utilization	Plant Derived Tagatose Degradation	agaR->PGPT0017955	1

Indirect Effects	ColonizingPlant System	Colonization-Plant Derived Substrate Usage	Plant Derived Carbohydrate-Hexoses Utilization	Plant Derived Tagatose Degradation	dpe|lre->PGPT0017960	1
Indirect Effects	ColonizingPlant System	Colonization-Plant Derived Substrate Usage	Plant Derived Carbohydrate-Hexoses Utilization	Plant Derived Trehalose Metabolism	crr->PGPT0014090	1
Indirect Effects	ColonizingPlant System	Colonization-Plant Derived Substrate Usage	Plant Derived Carbohydrate-Hexoses Utilization	Plant Derived Trehalose Metabolism	treB->PGPT0014075	1
Indirect Effects	ColonizingPlant System	Colonization-Plant Derived Substrate Usage	Plant Derived Carbohydrate-Hexoses Utilization	Plant Derived Trehalose Metabolism	treC->PGPT0014095	1
Indirect Effects	ColonizingPlant System	Colonization-Plant Derived Substrate Usage	Plant Derived Carbohydrate-Hexoses Utilization	Plant Derived Trehalose Metabolism	treR2->PGPT0014110	2
Indirect Effects	ColonizingPlant System	Colonization-Plant Derived Substrate Usage	Plant Derived Carbohydrate-Pentoses Utilization	Plant Derived Arabinose Metabolism	araA->PGPT0017450	1
Indirect Effects	ColonizingPlant System	Colonization-Plant Derived Substrate Usage	Plant Derived Carbohydrate-Pentoses Utilization	Plant Derived Arabinose Metabolism	araB|L ribuloKinase->PGPT0017410	1
Indirect Effects	ColonizingPlant System	Colonization-Plant Derived Substrate Usage	Plant Derived Carbohydrate-Pentoses Utilization	Plant Derived Arabinose Metabolism	araC->PGPT0017485	4
Indirect Effects	ColonizingPlant System	Colonization-Plant Derived Substrate Usage	Plant Derived Carbohydrate-Pentoses Utilization	Plant Derived Arabinose Metabolism	araD|ulaF|sgaE|sgbE->PGPT0017420	1
Indirect Effects	ColonizingPlant System	Colonization-Plant Derived Substrate Usage	Plant Derived Carbohydrate-Pentoses Utilization	Plant Derived Arabinose Metabolism	araR->PGPT0017490	2
Indirect Effects	ColonizingPlant	Colonization-Plant Derived Substrate	Plant Derived Carbohydrate-	Plant Derived Lyxose Degradation	araD|ulaF|sgaE|sgbE->PGPT0017420	1

	System	Usage	Pentoses Utilization			
Indirect Effects	ColonizingPlant System	Colonization-Plant Derived Substrate Usage	Plant Derived Carbohydrate-Pentoses Utilization	Plant Derived Pentose Metabolism	gnd|gntZ->PGPT0017385	4
Indirect Effects	ColonizingPlant System	Colonization-Plant Derived Substrate Usage	Plant Derived Carbohydrate-Pentoses Utilization	Plant Derived Pentose Metabolism	talA|talB->PGPT0017375	4
Indirect Effects	ColonizingPlant System	Colonization-Plant Derived Substrate Usage	Plant Derived Carbohydrate-Pentoses Utilization	Plant Derived Pentose Metabolism	tktA|tktB->PGPT0011200	4
Indirect Effects	ColonizingPlant System	Colonization-Plant Derived Substrate Usage	Plant Derived Carbohydrate-Pentoses Utilization	Plant Derived Pentose Metabolism	ykgB|pgl->PGPT0014975	2
Indirect Effects	ColonizingPlant System	Colonization-Plant Derived Substrate Usage	Plant Derived Carbohydrate-Pentoses Utilization	Plant Derived Pentose Metabolism	zwf->PGPT0017380	4
Indirect Effects	ColonizingPlant System	Colonization-Plant Derived Substrate Usage	Plant Derived Carbohydrate-Pentoses Utilization	Plant Derived Ribose Metabolism|Degradation	araB|L ribuloKinase->PGPT0017410	1
Indirect Effects	ColonizingPlant System	Colonization-Plant Derived Substrate Usage	Plant Derived Carbohydrate-Pentoses Utilization	Plant Derived Ribose Metabolism|Degradation	araD|ulaF|sgaE|sgbE->PGPT0017420	1
Indirect Effects	ColonizingPlant System	Colonization-Plant Derived Substrate Usage	Plant Derived Carbohydrate-Pentoses Utilization	Plant Derived Ribose Metabolism|Degradation	deoB->PGPT0017440	1
Indirect Effects	ColonizingPlant System	Colonization-Plant Derived Substrate Usage	Plant Derived Carbohydrate-Pentoses Utilization	Plant Derived Ribose Metabolism|Degradation	deoC->PGPT0017445	1
Indirect Effects	ColonizingPlant System	Colonization-Plant Derived Substrate Usage	Plant Derived Carbohydrate-Pentoses Utilization	Plant Derived Ribose Metabolism|Degradation	fucA->PGPT0017430	2
Indirect	Colonizin	Colonization-Plant	Plant Derived	Plant Derived Ribose	hisG->PGPT0017400	2

Effects	gPlant System	Derived Substrate Usage	Carbohydrate-Pentoses Utilization	Metabolism|Degradation		
Indirect Effects	ColonizingPlant System	Colonization-Plant Derived Substrate Usage	Plant Derived Carbohydrate-Pentoses Utilization	Plant Derived Ribose Metabolism|Degradation	rbsD->PGPT0016595	1
Indirect Effects	ColonizingPlant System	Colonization-Plant Derived Substrate Usage	Plant Derived Carbohydrate-Pentoses Utilization	Plant Derived Ribose Metabolism|Degradation	rbsK->PGPT0017405	1
Indirect Effects	ColonizingPlant System	Colonization-Plant Derived Substrate Usage	Plant Derived Carbohydrate-Pentoses Utilization	Plant Derived Ribose Metabolism|Degradation	rpe|cbbE->PGPT0017425	1
Indirect Effects	ColonizingPlant System	Colonization-Plant Derived Substrate Usage	Plant Derived Carbohydrate-Pentoses Utilization	Plant Derived Ribose Metabolism|Degradation	rpiA->PGPT0017390	1
Indirect Effects	ColonizingPlant System	Colonization-Plant Derived Substrate Usage	Plant Derived Carbohydrate-Pentoses Utilization	Plant Derived Ribose Metabolism|Degradation	rpiB->PGPT0017395	2
Indirect Effects	ColonizingPlant System	Colonization-Plant Derived Substrate Usage	Plant Derived Carbohydrate-Pentoses Utilization	Plant Derived xylose|Xylulose Degradation	araD|ulaF|sgaE|sgbE->PGPT0017420	1
Indirect Effects	ColonizingPlant System	Colonization-Plant Derived Substrate Usage	Plant Derived Carbohydrate-Pentoses Utilization	Plant Derived xylose|Xylulose Degradation	gutB->PGPT0017541	1
Indirect Effects	ColonizingPlant System	Colonization-Plant Derived Substrate Usage	Plant Derived Carbohydrate-Pentoses Utilization	Plant Derived xylose|Xylulose Degradation	rpe|cbbE->PGPT0017425	1
Indirect Effects	ColonizingPlant System	Colonization-Plant Derived Substrate Usage	Plant Derived Carbohydrate-Pentoses Utilization	Plant Derived xylose|Xylulose Degradation	xdh->PGPT0017545	2
Indirect Effects	ColonizingPlant System	Colonization-Plant Derived Substrate Usage	Plant Derived Carbohydrate-Pentoses Utilization	Plant Derived xylose|Xylulose Degradation	xylA->PGPT0017550	1

Indirect Effects	ColonizingPlant System	Colonization-Plant Derived Substrate Usage	Plant Derived Carbohydrate-Pentoses Utilization	Plant Derived xylose|Xylulose Degradation	xylB->PGPT0017535	2
Indirect Effects	ColonizingPlant System	Colonization-Plant Derived Substrate Usage	Plant Derived Carbohydrate-Pentoses Utilization	Plant Derived xylose|Xylulose Degradation	yajO|iolS->PGPT0017540	3
Indirect Effects	ColonizingPlant System	Colonization-Plant Derived Substrate Usage	Plant Derived Carbohydrate transport	Plant Derived Alpha-Glucoside transport	msmX|msmK|malK|sugC|ggtA|msiK->PGPT0016310	3
Indirect Effects	ColonizingPlant System	Colonization-Plant Derived Substrate Usage	Plant Derived Carbohydrate transport	Plant Derived ArabinoGalactan|Galactose oligomer|maltooligosaccharide transport	msmX|msmK|malK|sugC|ggtA|msiK->PGPT0016310	3
Indirect Effects	ColonizingPlant System	Colonization-Plant Derived Substrate Usage	Plant Derived Carbohydrate transport	Plant Derived Arabinooligosaccharide transport	msmX|msmK|malK|sugC|ggtA|msiK->PGPT0016310	3
Indirect Effects	ColonizingPlant System	Colonization-Plant Derived Substrate Usage	Plant Derived Carbohydrate transport	Plant Derived Ascorbate PTS System	ulaA|sgaT->PGPT0017105	1
Indirect Effects	ColonizingPlant System	Colonization-Plant Derived Substrate Usage	Plant Derived Carbohydrate transport	Plant Derived Ascorbate PTS System	ulaB|sgaB->PGPT0017110	1
Indirect Effects	ColonizingPlant System	Colonization-Plant Derived Substrate Usage	Plant Derived Carbohydrate transport	Plant Derived Ascorbate PTS System	ulaC|sgaA->PGPT0017115	1
Indirect Effects	ColonizingPlant System	Colonization-Plant Derived Substrate Usage	Plant Derived Carbohydrate transport	Plant Derived Beta-Glucoside PTS System I	bglF->PGPT0016920	2
Indirect Effects	ColonizingPlant System	Colonization-Plant Derived Substrate Usage	Plant Derived Carbohydrate transport	Plant Derived Cellobiose transport	msmX|msmK|malK|sugC|ggtA|msiK->PGPT0016310	3

Indirect Effects	ColonizingPlant System	Colonization-Plant Derived Substrate Usage	Plant Derived Carbohydrate transport	Plant Derived Cellobiose|DiAcetylChitobiose PTS System	celA|chbB->PGPT0016975	3
Indirect Effects	ColonizingPlant System	Colonization-Plant Derived Substrate Usage	Plant Derived Carbohydrate transport	Plant Derived Cellobiose|DiAcetylChitobiose PTS System	celB|chbC->PGPT0016970	3
Indirect Effects	ColonizingPlant System	Colonization-Plant Derived Substrate Usage	Plant Derived Carbohydrate transport	Plant Derived Cellobiose|DiAcetylChitobiose PTS System	celC|chbA->PGPT0016980	2
Indirect Effects	ColonizingPlant System	Colonization-Plant Derived Substrate Usage	Plant Derived Carbohydrate transport	Plant Derived Chitobiose transport I	msmX|msmK|malK|sugC|ggtA|msiK->PGPT0016310	3
Indirect Effects	ColonizingPlant System	Colonization-Plant Derived Substrate Usage	Plant Derived Carbohydrate transport	Plant Derived Fructooligosaccharide transport	msmX|msmK|malK|sugC|ggtA|msiK->PGPT0016310	3
Indirect Effects	ColonizingPlant System	Colonization-Plant Derived Substrate Usage	Plant Derived Carbohydrate transport	Plant Derived Fructose PTS System I	fruA->PGPT0017120	1
Indirect Effects	ColonizingPlant System	Colonization-Plant Derived Substrate Usage	Plant Derived Carbohydrate transport	Plant Derived Fructose PTS System II	levE->PGPT0017140	1
Indirect Effects	ColonizingPlant System	Colonization-Plant Derived Substrate Usage	Plant Derived Carbohydrate transport	Plant Derived Fructose PTS System II	levF->PGPT0017145	1
Indirect Effects	ColonizingPlant System	Colonization-Plant Derived Substrate Usage	Plant Derived Carbohydrate transport	Plant Derived Fructose PTS System II	levG->PGPT0017150	1
Indirect Effects	ColonizingPlant System	Colonization-Plant Derived Substrate Usage	Plant Derived Carbohydrate transport	Plant Derived Glucarate transport	gudP->PGPT0016475	2
Indirect Effects	ColonizingPlant	Colonization-Plant Derived Substrate	Plant Derived Carbohydrate	Plant Derived Glucose PTS System I	crr->PGPT0014090	1

	System	Usage	transport			
Indirect Effects	Colonizin gPlant System	Colonization-Plant Derived Substrate Usage	Plant Derived Carbohydrate transport	Plant Derived Glucose PTS System I	ptsG|glcA|glcB->PGPT0016870	1
Indirect Effects	Colonizin gPlant System	Colonization-Plant Derived Substrate Usage	Plant Derived Carbohydrate transport	Plant Derived Glucose PTS System I	ptsI->PGPT0002025	1
Indirect Effects	Colonizin gPlant System	Colonization-Plant Derived Substrate Usage	Plant Derived Carbohydrate transport	Plant Derived Glucose|Mannose transport I	msmX|msmK|malK|sugC|ggtA|msiK->PGPT0016310	3
Indirect Effects	Colonizin gPlant System	Colonization-Plant Derived Substrate Usage	Plant Derived Carbohydrate transport	Plant Derived Glycerol transport	ycaD->PGPT0014291	1
Indirect Effects	Colonizin gPlant System	Colonization-Plant Derived Substrate Usage	Plant Derived Carbohydrate transport	Plant Derived Glycerol Uptake	glpF|pduF->PGPT0004760	1
Indirect Effects	Colonizin gPlant System	Colonization-Plant Derived Substrate Usage	Plant Derived Carbohydrate transport	Plant Derived Glycoside|Pentoside|Hexuronide transport	TC GPH|yihO|xynP->PGPT0014410	3
Indirect Effects	Colonizin gPlant System	Colonization-Plant Derived Substrate Usage	Plant Derived Carbohydrate transport	Plant Derived Lactose transport	lacY|MFS transporter|OHS Family|Lactose permease->PGPT0016855	1
Indirect Effects	Colonizin gPlant System	Colonization-Plant Derived Substrate Usage	Plant Derived Carbohydrate transport	Plant Derived Lactose|Arabinose transport	lacE|araN->PGPT0016360	1
Indirect Effects	Colonizin gPlant System	Colonization-Plant Derived Substrate Usage	Plant Derived Carbohydrate transport	Plant Derived Lactose|Arabinose transport	lacF|araP->PGPT0016365	1
Indirect Effects	Colonizin gPlant	Colonization-Plant Derived Substrate	Plant Derived Carbohydrate	Plant Derived Lactose|Arabinose	lacG|araQ->PGPT0016370	1

	System	Usage	transport	transport		
Indirect Effects	ColonizingPlant System	Colonization-Plant Derived Substrate Usage	Plant Derived Carbohydrate transport	Plant Derived Maltose PTS System	malT->PGPT0016910	1
Indirect Effects	ColonizingPlant System	Colonization-Plant Derived Substrate Usage	Plant Derived Carbohydrate transport	Plant Derived Maltose|Glucose PTS System	crr->PGPT0014090	1
Indirect Effects	ColonizingPlant System	Colonization-Plant Derived Substrate Usage	Plant Derived Carbohydrate transport	Plant Derived Maltose|Maltodextrin transport	msmX|msmK|malK|sugC|ggtA|msiK->PGPT0016310	3
Indirect Effects	ColonizingPlant System	Colonization-Plant Derived Substrate Usage	Plant Derived Carbohydrate transport	Plant Derived Mannitol PTS System	mtlA|cmtA->PGPT0016945	1
Indirect Effects	ColonizingPlant System	Colonization-Plant Derived Substrate Usage	Plant Derived Carbohydrate transport	Plant Derived Mannitol|Sorbitol transport	msmX|msmK|malK|sugC|ggtA|msiK->PGPT0016310	3
Indirect Effects	ColonizingPlant System	Colonization-Plant Derived Substrate Usage	Plant Derived Carbohydrate transport	Plant Derived Mannosyl-Glycerate PTS System	mngR|farR->PGPT0016955	2
Indirect Effects	ColonizingPlant System	Colonization-Plant Derived Substrate Usage	Plant Derived Carbohydrate transport	Plant Derived Multiple Sugar transport III	msmX|msmK|malK|sugC|ggtA|msiK->PGPT0016310	3
Indirect Effects	ColonizingPlant System	Colonization-Plant Derived Substrate Usage	Plant Derived Carbohydrate transport	Plant Derived N Acetylglucosamine PTS System	nagE|nagP->PGPT0016860	1
Indirect Effects	ColonizingPlant System	Colonization-Plant Derived Substrate Usage	Plant Derived Carbohydrate transport	Plant Derived Other Sugar transport Related Proteins	MFS SET->PGPT0017195	1
Indirect Effects	ColonizingPlant System	Colonization-Plant Derived Substrate Usage	Plant Derived Carbohydrate transport	Plant Derived Other Sugar transport Related Proteins	TC PST->PGPT0017190	3
Indirect	Colonizin	Colonization-Plant	Plant Derived	Plant Derived Other	hxt->PGPT0014440	2

Effects	gPlant System	Derived Substrate Usage	Carbohydrate transport	Sugar transport Related Proteins		
Indirect Effects	Colonizin gPlant System	Colonization-Plant Derived Substrate Usage	Plant Derived Carbohydrate transport	Plant Derived Other Sugar transport Related Proteins	ynfM->PGPT0017201	2
Indirect Effects	Colonizin gPlant System	Colonization-Plant Derived Substrate Usage	Plant Derived Carbohydrate transport	Plant Derived Reaffinose|Stachyose|Melibiose transport	msmE->PGPT0016330	5
Indirect Effects	Colonizin gPlant System	Colonization-Plant Derived Substrate Usage	Plant Derived Carbohydrate transport	Plant Derived Reaffinose|Stachyose|Melibiose transport	msmF->PGPT0016335	4
Indirect Effects	Colonizin gPlant System	Colonization-Plant Derived Substrate Usage	Plant Derived Carbohydrate transport	Plant Derived Reaffinose|Stachyose|Melibiose transport	msmG->PGPT0016340	4
Indirect Effects	Colonizin gPlant System	Colonization-Plant Derived Substrate Usage	Plant Derived Carbohydrate transport	Plant Derived Reaffinose|Stachyose|Melibiose transport	msmX|msmK|malK|sugC|ggtA|msiK->PGPT0016310	3
Indirect Effects	Colonizin gPlant System	Colonization-Plant Derived Substrate Usage	Plant Derived Carbohydrate transport	Plant Derived Ribose|Autoinducer|xylose transport	rbsA->PGPT0016600	1
Indirect Effects	Colonizin gPlant System	Colonization-Plant Derived Substrate Usage	Plant Derived Carbohydrate transport	Plant Derived Ribose|Autoinducer|xylose transport	rbsB->PGPT0015740	1
Indirect Effects	Colonizin gPlant System	Colonization-Plant Derived Substrate Usage	Plant Derived Carbohydrate transport	Plant Derived Ribose|Autoinducer|xylose transport	rbsC->PGPT0016590	1
Indirect Effects	Colonizin gPlant System	Colonization-Plant Derived Substrate Usage	Plant Derived Carbohydrate transport	Plant Derived Ribose|Autoinducer|xylose transport	rbsD->PGPT0016595	1
Indirect Effects	Colonizin gPlant System	Colonization-Plant Derived Substrate Usage	Plant Derived Carbohydrate transport	Plant Derived Simple Sugar transport	ABC SS A->PGPT0017155	1

Indirect Effects	ColonizingPlant System	Colonization-Plant Derived Substrate Usage	Plant Derived Carbohydrate transport	Plant Derived Simple Sugar transport	ABC SS P->PGPT0017160	1
Indirect Effects	ColonizingPlant System	Colonization-Plant Derived Substrate Usage	Plant Derived Carbohydrate transport	Plant Derived Simple Sugar transport	ABC SS S->PGPT0017165	1
Indirect Effects	ColonizingPlant System	Colonization-Plant Derived Substrate Usage	Plant Derived Carbohydrate transport	Plant Derived Sucrose PTS System	scrA|sacP|sacX|ptsS->PGPT0014190	3
Indirect Effects	ColonizingPlant System	Colonization-Plant Derived Substrate Usage	Plant Derived Carbohydrate transport	Plant Derived xylose transport II	xylE->PGPT0014450	1
Indirect Effects	ColonizingPlant System	Colonization-Plant Derived Substrate Usage	Plant Derived Carbohydrate transport	Plant Derived lnsitol transport 2	iolT->PGPT0016801	1
Indirect Effects	ColonizingPlant System	Colonization-Plant Derived Substrate Usage	Plant Derived Carbohydrate transport	Plant Derived sn-Glycerol-3P transport	ugpA->PGPT0016840	1
Indirect Effects	ColonizingPlant System	Colonization-Plant Derived Substrate Usage	Plant Derived Carbohydrate transport	Plant Derived sn-Glycerol-3P transport	ugpB->PGPT0016835	1
Indirect Effects	ColonizingPlant System	Colonization-Plant Derived Substrate Usage	Plant Derived Carbohydrate transport	Plant Derived sn-Glycerol-3P transport	ugpC->PGPT0016850	1
Indirect Effects	ColonizingPlant System	Colonization-Plant Derived Substrate Usage	Plant Derived Carbohydrate transport	Plant Derived sn-Glycerol-3P transport	ugpE->PGPT0016845	1
Indirect Effects	ColonizingPlant System	Colonization-Plant Derived Substrate Usage	Plant Derived Carbohydrate Usage-Others	Plant Derived Carbon Catebolite Regulation	ccpA->PGPT0019459	2
Indirect Effects	ColonizingPlant	Colonization-Plant Derived Substrate	Plant Derived Carbohydrate Usage-	Plant Derived Carbon Catebolite Regulation	cggR->PGPT0019460	1

	System	Usage	Others			
Indirect Effects	ColonizingPlant System	Colonization-Plant Derived Substrate Usage	Plant Derived Carbohydrate Usage-Others	Plant Derived Levan Degradation	sacC|levB->PGPT0019220	1
Indirect Effects	ColonizingPlant System	Colonization-Plant Derived Substrate Usage	Plant Derived Carbohydrate Usage-Others	Plant Derived Lichenan Degradation	licR->PGPT0019430	1
Indirect Effects	ColonizingPlant System	Colonization-Plant Derived Substrate Usage	Plant Derived Carbohydrate Usage-Others	Plant Derived propionate|Propanoate Utilization	bkdA1->PGPT0008861	1
Indirect Effects	ColonizingPlant System	Colonization-Plant Derived Substrate Usage	Plant Derived Carbohydrate Usage-Others	Plant Derived propionate|Propanoate Utilization	bkdA2|bfmBAB->PGPT0008862	1
Indirect Effects	ColonizingPlant System	Colonization-Plant Derived Substrate Usage	Plant Derived Carbohydrate Usage-Others	Plant Derived propionate|Propanoate Utilization	pccB->PGPT0001712	1
Indirect Effects	ColonizingPlant System	Colonization-Plant Derived Substrate Usage	Plant Derived Carbohydrate Usage-Others	Plant Derived propionate|Propanoate Utilization	prpR->PGPT0019455	1
Indirect Effects	ColonizingPlant System	Colonization-Plant Derived Substrate Usage	Plant Derived Carnithine Usage	Plant Derived Carnithine Degradation	dhcR->PGPT0021950	2
Indirect Effects	ColonizingPlant System	Colonization-Plant Derived Substrate Usage	Plant Derived Complex Sugar Utilization	Plant Derived Cellulose|HemiCellulose Degradation	bgaB|lacA->PGPT0017815	1
Indirect Effects	ColonizingPlant System	Colonization-Plant Derived Substrate Usage	Plant Derived Complex Sugar Utilization	Plant Derived Cellulose|HemiCellulose Degradation	pda|pgdA->PGPT0018645	8
Indirect Effects	ColonizingPlant System	Colonization-Plant Derived Substrate Usage	Plant Derived Complex Sugar Utilization	Plant Derived Fructan|Mannan Breakdown	fruA->PGPT0018745	1
Indirect	Colonizin	Colonization-Plant	Plant Derived	Plant Derived	lacZ->PGPT0017810	1

Effects	gPlant System	Derived Substrate Usage	Complex Sugar Utilization	Glucan|Glycan Breakdown		
Indirect Effects	Colonizing Plant System	Colonization-Plant Derived Substrate Usage	Plant Derived Complex Sugar Utilization	Plant Derived Maltose Degradation	crr->PGPT0014090	1
Indirect Effects	Colonizing Plant System	Colonization-Plant Derived Substrate Usage	Plant Derived Complex Sugar Utilization	Plant Derived Maltose Degradation	maa|nodL->PGPT0018611	1
Indirect Effects	Colonizing Plant System	Colonization-Plant Derived Substrate Usage	Plant Derived Complex Sugar Utilization	Plant Derived Maltose Degradation	mapP->PGPT0018615	1
Indirect Effects	Colonizing Plant System	Colonization-Plant Derived Substrate Usage	Plant Derived Complex Sugar Utilization	Plant Derived Pectin Degradation	yteR|yesR->PGPT0018255	1
Indirect Effects	Colonizing Plant System	Colonization-Plant Derived Substrate Usage	Plant Derived Complex Sugar Utilization	Plant Derived Pullulan Degradation	pulA->PGPT0012195	2
Indirect Effects	Colonizing Plant System	Colonization-Plant Derived Substrate Usage	Plant Derived Complex Sugar Utilization	Plant Derived Raffinose|Stachyose Degradation	melA->PGPT0017850	1
Indirect Effects	Colonizing Plant System	Colonization-Plant Derived Substrate Usage	Plant Derived Complex Sugar Utilization	Plant Derived Raffinose|Stachyose Degradation	rafA|galA->PGPT0018835	1
Indirect Effects	Colonizing Plant System	Colonization-Plant Derived Substrate Usage	Plant Derived Complex Sugar Utilization	Plant Derived Raffinose|Stachyose Degradation	sacA->PGPT0018815	2
Indirect Effects	Colonizing Plant System	Colonization-Plant Derived Substrate Usage	Plant Derived Complex Sugar Utilization	Plant Derived Starch|Glycogen Degradation	amyA|malS->PGPT0018575	3
Indirect Effects	Colonizing Plant System	Colonization-Plant Derived Substrate Usage	Plant Derived Complex Sugar Utilization	Plant Derived Starch|Glycogen Degradation	Beta Amylase->PGPT0018580	1

Indirect Effects	ColonizingPlant System	Colonization-Plant Derived Substrate Usage	Plant Derived Complex Sugar Utilization	Plant Derived Starch|Glycogen Degradation	glgP->PGPT0018545	1
Indirect Effects	ColonizingPlant System	Colonization-Plant Derived Substrate Usage	Plant Derived Complex Sugar Utilization	Plant Derived Sucrose Metabolism|Degradation	sacA->PGPT0018815	2
Indirect Effects	ColonizingPlant System	Colonization-Plant Derived Substrate Usage	Plant Derived Complex Sugar Utilization	Plant Derived Sucrose Metabolism|Degradation	sacB->PGPT0013775	2
Indirect Effects	ColonizingPlant System	Colonization-Plant Derived Substrate Usage	Plant Derived Complex Sugar Utilization	Plant Derived Sucrose Metabolism|Degradation	scrR->PGPT0018830	3
Indirect Effects	ColonizingPlant System	Colonization-Plant Derived Substrate Usage	Plant Derived Glycoside Utilization	Plant Derived Beta-Glycoside Degradation	celA|chbB->PGPT0016975	3
Indirect Effects	ColonizingPlant System	Colonization-Plant Derived Substrate Usage	Plant Derived Glycoside Utilization	Plant Derived Beta-Glycoside Degradation	celB|chbC->PGPT0016970	3
Indirect Effects	ColonizingPlant System	Colonization-Plant Derived Substrate Usage	Plant Derived Glycoside Utilization	Plant Derived Beta-Glycoside Degradation	celC|chbA->PGPT0016980	2
Indirect Effects	ColonizingPlant System	Colonization-Plant Derived Substrate Usage	Plant Derived Glycoside Utilization	Plant Derived Beta-Glycoside Degradation	celF|licH|chbF->PGPT0019105	3
Indirect Effects	ColonizingPlant System	Colonization-Plant Derived Substrate Usage	Plant Derived Glycoside Utilization	Plant Derived Beta-Glycoside Degradation	crr->PGPT0014090	1
Indirect Effects	ColonizingPlant System	Colonization-Plant Derived Substrate Usage	Plant Derived Glycoside Utilization	Plant Derived Glycoside-Glycosidases|Glycosyl Hydrolases	acm->PGPT0019160	3
Indirect	Colonizin	Colonization-Plant	Plant Derived	Plant Derived	amyA|malS-	3

Effects	gPlant System	Derived Substrate Usage	Glycoside Utilization	Glycoside-Glycosidases|Glycosyl Hydrolases	>PGPT0018575	
Indirect Effects	Colonizin gPlant System	Colonization-Plant Derived Substrate Usage	Plant Derived Glycoside Utilization	Plant Derived Glycoside-Glycosidases|Glycosyl Hydrolases	Beta Amylase->PGPT0018580	1
Indirect Effects	Colonizin gPlant System	Colonization-Plant Derived Substrate Usage	Plant Derived Glycoside Utilization	Plant Derived Glycoside-Glycosidases|Glycosyl Hydrolases	bgaB|lacA->PGPT0017815	1
Indirect Effects	Colonizin gPlant System	Colonization-Plant Derived Substrate Usage	Plant Derived Glycoside Utilization	Plant Derived Glycoside-Glycosidases|Glycosyl Hydrolases	bglA->PGPT0019255	2
Indirect Effects	Colonizin gPlant System	Colonization-Plant Derived Substrate Usage	Plant Derived Glycoside Utilization	Plant Derived Glycoside-Glycosidases|Glycosyl Hydrolases	celF|licH|chbF->PGPT0019105	3
Indirect Effects	Colonizin gPlant System	Colonization-Plant Derived Substrate Usage	Plant Derived Glycoside Utilization	Plant Derived Glycoside-Glycosidases|Glycosyl Hydrolases	fruA->PGPT0018745	1
Indirect Effects	Colonizin gPlant System	Colonization-Plant Derived Substrate Usage	Plant Derived Glycoside Utilization	Plant Derived Glycoside-Glycosidases|Glycosyl Hydrolases	lacZ->PGPT0017810	1
Indirect Effects	Colonizin gPlant System	Colonization-Plant Derived Substrate Usage	Plant Derived Glycoside Utilization	Plant Derived Glycoside-Glycosidases|Glycosyl Hydrolases	melA->PGPT0017850	1
Indirect	Colonizin	Colonization-Plant	Plant Derived	Plant Derived	pulA->PGPT0012195	2

Effects	gPlant System	Derived Substrate Usage	Glycoside Utilization	Glycoside-Glycosidases|Glycosyl Hydrolases		
Indirect Effects	Colonizin gPlant System	Colonization-Plant Derived Substrate Usage	Plant Derived Glycoside Utilization	Plant Derived Glycoside-Glycosidases|Glycosyl Hydrolases	rafA|galA->PGPT0018835	1
Indirect Effects	Colonizin gPlant System	Colonization-Plant Derived Substrate Usage	Plant Derived Glycoside Utilization	Plant Derived Glycoside-Glycosidases|Glycosyl Hydrolases	sacA->PGPT0018815	2
Indirect Effects	Colonizin gPlant System	Colonization-Plant Derived Substrate Usage	Plant Derived Glycoside Utilization	Plant Derived Glycoside-Glycosidases|Glycosyl Hydrolases	sacC|levB->PGPT0019220	1
Indirect Effects	Colonizin gPlant System	Colonization-Plant Derived Substrate Usage	Plant Derived Glycoside Utilization	Plant Derived Glycoside-Glycosidases|Glycosyl Hydrolases	treC->PGPT0014095	1
Indirect Effects	Colonizin gPlant System	Colonization-Plant Derived Substrate Usage	Plant Derived Glycoside Utilization	Plant Derived Glycoside-Glycosidases|Glycosyl Hydrolases	yteR|yesR->PGPT0018255	1
Indirect Effects	Colonizin gPlant System	Colonization-Plant Derived Substrate Usage	Plant Derived Glycoside Utilization	Plant Derived Glycoside Usage Regulation	licT|bglG->PGPT0014761	3
Indirect Effects	Colonizin gPlant System	Colonization-Plant Derived Substrate Usage	Plant Derived Glycoside Utilization	Plant Derived Glycoside Usage Regulation	paiB|yumE->PGPT0014760	1
Indirect Effects	Colonizin gPlant System	Colonization-Plant Derived Substrate Usage	Plant Derived Glycoside Utilization	Plant Derived Non-Peptide Carbon-Nitrogen Bonds	pda|pgdA->PGPT0018645	8

				Cleavage		
Indirect Effects	ColonizingPlant System	Colonization-Plant Derived Substrate Usage	Plant Derived Glycoside Utilization	Plant Derived Non-Peptide Carbon-Nitrogen Bonds Cleavage	sle1|yocH->PGPT0019120	3
Indirect Effects	ColonizingPlant System	Colonization-Plant Derived Substrate Usage	Plant Derived Glycoside Utilization	Plant Derived Non-Peptide Carbon-Nitrogen Bonds Cleavage	xlyAB->PGPT0019115	1
Indirect Effects	ColonizingPlant System	Colonization-Plant Derived Substrate Usage	Plant Derived Lipid Metabolism	Plant Derived Fatty Acid Degradation	ECHS1->PGPT0021790	2
Indirect Effects	ColonizingPlant System	Colonization-Plant Derived Substrate Usage	Plant Derived Lipid Metabolism	Plant Derived Fatty Acid Degradation	EC 1 1 1 1|adh->PGPT0006375	1
Indirect Effects	ColonizingPlant System	Colonization-Plant Derived Substrate Usage	Plant Derived Lipid Metabolism	Plant Derived Fatty Acid Degradation	acd->PGPT0008385	2
Indirect Effects	ColonizingPlant System	Colonization-Plant Derived Substrate Usage	Plant Derived Lipid Metabolism	Plant Derived Fatty Acid Degradation	aldH|dhaS->PGPT0006875	5
Indirect Effects	ColonizingPlant System	Colonization-Plant Derived Substrate Usage	Plant Derived Lipid Metabolism	Plant Derived Fatty Acid Degradation	aToA->PGPT0021750	1
Indirect Effects	ColonizingPlant System	Colonization-Plant Derived Substrate Usage	Plant Derived Lipid Metabolism	Plant Derived Fatty Acid Degradation	aToD->PGPT0021755	1
Indirect Effects	ColonizingPlant System	Colonization-Plant Derived Substrate Usage	Plant Derived Lipid Metabolism	Plant Derived Fatty Acid Degradation	aToE->PGPT0021760	1
Indirect Effects	ColonizingPlant	Colonization-Plant Derived Substrate	Plant Derived Lipid Metabolism	Plant Derived Fatty Acid Degradation	aToS->PGPT0021740	1

	System	Usage				
Indirect Effects	ColonizingPlant System	Colonization-Plant Derived Substrate Usage	Plant Derived Lipid Metabolism	Plant Derived Fatty Acid Degradation	bcd->PGPT0002285	8
Indirect Effects	ColonizingPlant System	Colonization-Plant Derived Substrate Usage	Plant Derived Lipid Metabolism	Plant Derived Fatty Acid Degradation	cpxB->PGPT0006740	1
Indirect Effects	ColonizingPlant System	Colonization-Plant Derived Substrate Usage	Plant Derived Lipid Metabolism	Plant Derived Fatty Acid Degradation	fadB->PGPT0008390	2
Indirect Effects	ColonizingPlant System	Colonization-Plant Derived Substrate Usage	Plant Derived Lipid Metabolism	Plant Derived Fatty Acid Degradation	fadD->PGPT0008380	6
Indirect Effects	ColonizingPlant System	Colonization-Plant Derived Substrate Usage	Plant Derived Lipid Metabolism	Plant Derived Fatty Acid Degradation	fadN->PGPT0008395	1
Indirect Effects	ColonizingPlant System	Colonization-Plant Derived Substrate Usage	Plant Derived Lipid Metabolism	Plant Derived Fatty Acid Degradation	frmA|ADH5|adhC->PGPT0006355	2
Indirect Effects	ColonizingPlant System	Colonization-Plant Derived Substrate Usage	Plant Derived Lipid Metabolism	Plant Derived Fatty Acid Degradation	paaF|echA->PGPT0001860	1
Indirect Effects	ColonizingPlant System	Colonization-Plant Derived Substrate Usage	Plant Derived Lipid Metabolism	Plant Derived Fatty Acid Degradation	yiaY->PGPT0006370	1
Indirect Effects	ColonizingPlant System	Colonization-Plant Derived Substrate Usage	Plant Derived Lipid Metabolism	Plant Derived GlycerophosphoLipid Degradation	glpA|glpD->PGPT0006775	1
Indirect Effects	ColonizingPlant System	Colonization-Plant Derived Substrate Usage	Plant Derived Lipid Metabolism	Plant Derived GlycerophosphoLipid Degradation	glpQ|ugpQ->PGPT0018470	5
Indirect	Colonizin	Colonization-Plant	Plant Derived	Plant Derived	bmpA->PGPT0021055	3

Effects	gPlant System	Derived Substrate Usage	Nucleoside Metabolism	Nucleoside transport		
Indirect Effects	Colonizin gPlant System	Colonization-Plant Derived Substrate Usage	Plant Derived Nucleoside Metabolism	Plant Derived Nucleoside transport	cytR->PGPT0021075	3
Indirect Effects	Colonizin gPlant System	Colonization-Plant Derived Substrate Usage	Plant Derived Nucleoside Metabolism	Plant Derived Nucleoside transport	nucp->PGPT0021065	2
Indirect Effects	Colonizin gPlant System	Colonization-Plant Derived Substrate Usage	Plant Derived Nucleoside Metabolism	Plant Derived Nucleoside transport	nupA|yufO->PGPT0021040	2
Indirect Effects	Colonizin gPlant System	Colonization-Plant Derived Substrate Usage	Plant Derived Nucleoside Metabolism	Plant Derived Nucleoside transport	nupB|yufP->PGPT0021045	2
Indirect Effects	Colonizin gPlant System	Colonization-Plant Derived Substrate Usage	Plant Derived Nucleoside Metabolism	Plant Derived Nucleoside transport	nupC|yufQ->PGPT0021050	2
Indirect Effects	Colonizin gPlant System	Colonization-Plant Derived Substrate Usage	Plant Derived Nucleoside Metabolism	Plant Derived Nucleoside transport	pbuE->PGPT0021080	3
Indirect Effects	Colonizin gPlant System	Colonization-Plant Derived Substrate Usage	Plant Derived Nucleoside Metabolism	Plant Derived Nucleoside transport	yxjA|nupG->PGPT0021085	1
Indirect Effects	Colonizin gPlant System	Colonization-Plant Derived Substrate Usage	Plant Derived Nucleoside Metabolism	Plant Derived Purine Degradation	allB->PGPT0000870	1
Indirect Effects	Colonizin gPlant System	Colonization-Plant Derived Substrate Usage	Plant Derived Nucleoside Metabolism	Plant Derived Purine Degradation	hpyO->PGPT0021120	1
Indirect Effects	Colonizin gPlant System	Colonization-Plant Derived Substrate Usage	Plant Derived Nucleoside Metabolism	Plant Derived Purine Degradation	uraD->PGPT0021130	1

Indirect Effects	ColonizingPlant System	Colonization-Plant Derived Substrate Usage	Plant Derived Nucleoside Metabolism	Plant Derived Purine Degradation	uraH|pucM|hiuH->PGPT0021125	1
Indirect Effects	ColonizingPlant System	Colonization-Plant Derived Substrate Usage	Plant Derived Nucleoside Metabolism	Plant Derived Purine Degradation	xdhB|pucD->PGPT0007265	1
Indirect Effects	ColonizingPlant System	Colonization-Plant Derived Substrate Usage	Plant Derived Nucleoside Metabolism	Plant Derived Purine Degradation	yagT->PGPT0007290	1
Indirect Effects	ColonizingPlant System	Colonization-Plant Derived Substrate Usage	Plant Derived Nucleoside Metabolism	Plant Derived Purine Degradation	ygeT|xdhB->PGPT0007270	1
Indirect Effects	ColonizingPlant System	Colonization-Plant Derived Substrate Usage	Plant Derived Nucleoside Metabolism	Plant Derived Purine Metabolism	ade->PGPT0021510	2
Indirect Effects	ColonizingPlant System	Colonization-Plant Derived Substrate Usage	Plant Derived Nucleoside Metabolism	Plant Derived Purine Metabolism	adk|AK->PGPT0009040	1
Indirect Effects	ColonizingPlant System	Colonization-Plant Derived Substrate Usage	Plant Derived Nucleoside Metabolism	Plant Derived Purine Metabolism	allC->PGPT0000875	1
Indirect Effects	ColonizingPlant System	Colonization-Plant Derived Substrate Usage	Plant Derived Nucleoside Metabolism	Plant Derived Purine Metabolism	apt->PGPT0021455	1
Indirect Effects	ColonizingPlant System	Colonization-Plant Derived Substrate Usage	Plant Derived Nucleoside Metabolism	Plant Derived Purine Metabolism	cpdA->PGPT0021595	1
Indirect Effects	ColonizingPlant System	Colonization-Plant Derived Substrate Usage	Plant Derived Nucleoside Metabolism	Plant Derived Purine Metabolism	cysC->PGPT0002780	2
Indirect Effects	ColonizingPlant	Colonization-Plant Derived Substrate	Plant Derived Nucleoside	Plant Derived Purine Metabolism	deoB->PGPT0017440	1

	System	Usage	Metabolism			
Indirect Effects	ColonizingPlant System	Colonization-Plant Derived Substrate Usage	Plant Derived Nucleoside Metabolism	Plant Derived Purine Metabolism	dgk->PGPT0021655	1
Indirect Effects	ColonizingPlant System	Colonization-Plant Derived Substrate Usage	Plant Derived Nucleoside Metabolism	Plant Derived Purine Metabolism	gmk->PGPT0021475	1
Indirect Effects	ColonizingPlant System	Colonization-Plant Derived Substrate Usage	Plant Derived Nucleoside Metabolism	Plant Derived Purine Metabolism	guaA->PGPT0021580	1
Indirect Effects	ColonizingPlant System	Colonization-Plant Derived Substrate Usage	Plant Derived Nucleoside Metabolism	Plant Derived Purine Metabolism	guaB->PGPT0021445	2
Indirect Effects	ColonizingPlant System	Colonization-Plant Derived Substrate Usage	Plant Derived Nucleoside Metabolism	Plant Derived Purine Metabolism	guaC->PGPT0021450	2
Indirect Effects	ColonizingPlant System	Colonization-Plant Derived Substrate Usage	Plant Derived Nucleoside Metabolism	Plant Derived Purine Metabolism	hit->PGPT0021690	2
Indirect Effects	ColonizingPlant System	Colonization-Plant Derived Substrate Usage	Plant Derived Nucleoside Metabolism	Plant Derived Purine Metabolism	hprT|hpt->PGPT0007295	1
Indirect Effects	ColonizingPlant System	Colonization-Plant Derived Substrate Usage	Plant Derived Nucleoside Metabolism	Plant Derived Purine Metabolism	iunH->PGPT0013465	2
Indirect Effects	ColonizingPlant System	Colonization-Plant Derived Substrate Usage	Plant Derived Nucleoside Metabolism	Plant Derived Purine Metabolism	nudF->PGPT0021525	2
Indirect Effects	ColonizingPlant System	Colonization-Plant Derived Substrate Usage	Plant Derived Nucleoside Metabolism	Plant Derived Purine Metabolism	pgm->PGPT0017710	2
Indirect	Colonizin	Colonization-Plant	Plant Derived	Plant Derived Purine	ppx|ppx gppA-	1

Effects	gPlant System	Derived Substrate Usage	Nucleoside Metabolism	Metabolism	>PGPT0002595	
Indirect Effects	Colonizin gPlant System	Colonization-Plant Derived Substrate Usage	Plant Derived Nucleoside Metabolism	Plant Derived Purine Metabolism	prsA->PGPT0021480	1
Indirect Effects	Colonizin gPlant System	Colonization-Plant Derived Substrate Usage	Plant Derived Nucleoside Metabolism	Plant Derived Purine Metabolism	pucG->PGPT0021460	1
Indirect Effects	Colonizin gPlant System	Colonization-Plant Derived Substrate Usage	Plant Derived Nucleoside Metabolism	Plant Derived Purine Metabolism	purA->PGPT0020140	1
Indirect Effects	Colonizin gPlant System	Colonization-Plant Derived Substrate Usage	Plant Derived Nucleoside Metabolism	Plant Derived Purine Metabolism	purB->PGPT0021555	1
Indirect Effects	Colonizin gPlant System	Colonization-Plant Derived Substrate Usage	Plant Derived Nucleoside Metabolism	Plant Derived Purine Metabolism	purC->PGPT0021565	1
Indirect Effects	Colonizin gPlant System	Colonization-Plant Derived Substrate Usage	Plant Derived Nucleoside Metabolism	Plant Derived Purine Metabolism	purD->PGPT0021575	1
Indirect Effects	Colonizin gPlant System	Colonization-Plant Derived Substrate Usage	Plant Derived Nucleoside Metabolism	Plant Derived Purine Metabolism	purE->PGPT0021545	1
Indirect Effects	Colonizin gPlant System	Colonization-Plant Derived Substrate Usage	Plant Derived Nucleoside Metabolism	Plant Derived Purine Metabolism	purF->PGPT0020225	1
Indirect Effects	Colonizin gPlant System	Colonization-Plant Derived Substrate Usage	Plant Derived Nucleoside Metabolism	Plant Derived Purine Metabolism	purH->PGPT0008110	2
Indirect Effects	Colonizin gPlant System	Colonization-Plant Derived Substrate Usage	Plant Derived Nucleoside Metabolism	Plant Derived Purine Metabolism	purK->PGPT0021550	1

Indirect Effects	ColonizingPlant System	Colonization-Plant Derived Substrate Usage	Plant Derived Nucleoside Metabolism	Plant Derived Purine Metabolism	purL->PGPT0021730	1
Indirect Effects	ColonizingPlant System	Colonization-Plant Derived Substrate Usage	Plant Derived Nucleoside Metabolism	Plant Derived Purine Metabolism	purM->PGPT0021570	1
Indirect Effects	ColonizingPlant System	Colonization-Plant Derived Substrate Usage	Plant Derived Nucleoside Metabolism	Plant Derived Purine Metabolism	purN->PGPT0008095	1
Indirect Effects	ColonizingPlant System	Colonization-Plant Derived Substrate Usage	Plant Derived Nucleoside Metabolism	Plant Derived Purine Metabolism	purQ->PGPT0020220	1
Indirect Effects	ColonizingPlant System	Colonization-Plant Derived Substrate Usage	Plant Derived Nucleoside Metabolism	Plant Derived Purine Metabolism	purS->PGPT0021725	1
Indirect Effects	ColonizingPlant System	Colonization-Plant Derived Substrate Usage	Plant Derived Nucleoside Metabolism	Plant Derived Purine Metabolism	pyk->PGPT0002020	2
Indirect Effects	ColonizingPlant System	Colonization-Plant Derived Substrate Usage	Plant Derived Nucleoside Metabolism	Plant Derived Purine Metabolism	sat|met3->PGPT0002410	1
Indirect Effects	ColonizingPlant System	Colonization-Plant Derived Substrate Usage	Plant Derived Nucleoside Metabolism	Plant Derived Purine Metabolism	spoT->PGPT0014310	1
Indirect Effects	ColonizingPlant System	Colonization-Plant Derived Substrate Usage	Plant Derived Nucleoside Metabolism	Plant Derived Purine Metabolism	spoT|relA->PGPT0021700	1
Indirect Effects	ColonizingPlant System	Colonization-Plant Derived Substrate Usage	Plant Derived Nucleoside Metabolism	Plant Derived Purine Metabolism	tilS hprT->PGPT0021660	1
Indirect Effects	ColonizingPlant	Colonization-Plant Derived Substrate	Plant Derived Nucleoside	Plant Derived Purine Metabolism	Urea->PGPT0000955	1

	System	Usage	Metabolism			
Indirect Effects	ColonizingPlant System	Colonization-Plant Derived Substrate Usage	Plant Derived Nucleoside Metabolism	Plant Derived Purine Metabolism	ureB->PGPT0000960	1
Indirect Effects	ColonizingPlant System	Colonization-Plant Derived Substrate Usage	Plant Derived Nucleoside Metabolism	Plant Derived Purine Metabolism	ureC->PGPT0000965	1
Indirect Effects	ColonizingPlant System	Colonization-Plant Derived Substrate Usage	Plant Derived Nucleoside Metabolism	Plant Derived Purine Metabolism	xpt->PGPT0007305	1
Indirect Effects	ColonizingPlant System	Colonization-Plant Derived Substrate Usage	Plant Derived Nucleoside Metabolism	Plant Derived Purine Metabolism	yjjX->PGPT0021540	1
Indirect Effects	ColonizingPlant System	Colonization-Plant Derived Substrate Usage	Plant Derived Nucleoside Metabolism	Plant Derived Purine Metabolism	ywaC|yjbM->PGPT0014825	1
Indirect Effects	ColonizingPlant System	Colonization-Plant Derived Substrate Usage	Plant Derived Nucleoside Metabolism	Plant Derived Pyrimidine Degradation	UPB1 Like|pydC->PGPT0008885	5
Indirect Effects	ColonizingPlant System	Colonization-Plant Derived Substrate Usage	Plant Derived Nucleoside Metabolism	Plant Derived Pyrimidine Degradation	dht|hydA->PGPT0008880	1
Indirect Effects	ColonizingPlant System	Colonization-Plant Derived Substrate Usage	Plant Derived Nucleoside Metabolism	Plant Derived Pyrimidine Degradation	preA->PGPT0008875	1
Indirect Effects	ColonizingPlant System	Colonization-Plant Derived Substrate Usage	Plant Derived Nucleoside Metabolism	Plant Derived Pyrimidine Degradation	preT->PGPT0008870	1
Indirect Effects	ColonizingPlant System	Colonization-Plant Derived Substrate Usage	Plant Derived Nucleoside Metabolism	Plant Derived Pyrimidine Degradation	pydC->PGPT0021095	1
Indirect	Colonizin	Colonization-Plant	Plant Derived	Plant Derived	carA->PGPT0021155	2

Effects	gPlant System	Derived Substrate Usage	Nucleoside Metabolism	Pyrimidine Metabolism		
Indirect Effects	Colonizin gPlant System	Colonization-Plant Derived Substrate Usage	Plant Derived Nucleoside Metabolism	Plant Derived Pyrimidine Metabolism	carB->PGPT0021150	2
Indirect Effects	Colonizin gPlant System	Colonization-Plant Derived Substrate Usage	Plant Derived Nucleoside Metabolism	Plant Derived Pyrimidine Metabolism	cdd->PGPT0021360	2
Indirect Effects	Colonizin gPlant System	Colonization-Plant Derived Substrate Usage	Plant Derived Nucleoside Metabolism	Plant Derived Pyrimidine Metabolism	cmk->PGPT0021220	1
Indirect Effects	Colonizin gPlant System	Colonization-Plant Derived Substrate Usage	Plant Derived Nucleoside Metabolism	Plant Derived Pyrimidine Metabolism	codA->PGPT0021315	1
Indirect Effects	Colonizin gPlant System	Colonization-Plant Derived Substrate Usage	Plant Derived Nucleoside Metabolism	Plant Derived Pyrimidine Metabolism	comEB|tadA->PGPT0021380	1
Indirect Effects	Colonizin gPlant System	Colonization-Plant Derived Substrate Usage	Plant Derived Nucleoside Metabolism	Plant Derived Pyrimidine Metabolism	dcd->PGPT0021225	1
Indirect Effects	Colonizin gPlant System	Colonization-Plant Derived Substrate Usage	Plant Derived Nucleoside Metabolism	Plant Derived Pyrimidine Metabolism	dck->PGPT0021370	1
Indirect Effects	Colonizin gPlant System	Colonization-Plant Derived Substrate Usage	Plant Derived Nucleoside Metabolism	Plant Derived Pyrimidine Metabolism	deoD->PGPT0013385	1
Indirect Effects	Colonizin gPlant System	Colonization-Plant Derived Substrate Usage	Plant Derived Nucleoside Metabolism	Plant Derived Pyrimidine Metabolism	ndk->PGPT0021210	1
Indirect Effects	Colonizin gPlant System	Colonization-Plant Derived Substrate Usage	Plant Derived Nucleoside Metabolism	Plant Derived Pyrimidine Metabolism	nrdA|nrdE->PGPT0021340	3

Indirect Effects	ColonizingPlant System	Colonization-Plant Derived Substrate Usage	Plant Derived Nucleoside Metabolism	Plant Derived Pyrimidine Metabolism	nrdB|nrdF->PGPT0021345	1
Indirect Effects	ColonizingPlant System	Colonization-Plant Derived Substrate Usage	Plant Derived Nucleoside Metabolism	Plant Derived Pyrimidine Metabolism	nudG->PGPT0021430	1
Indirect Effects	ColonizingPlant System	Colonization-Plant Derived Substrate Usage	Plant Derived Nucleoside Metabolism	Plant Derived Pyrimidine Metabolism	pdp|deoA->PGPT0021295	1
Indirect Effects	ColonizingPlant System	Colonization-Plant Derived Substrate Usage	Plant Derived Nucleoside Metabolism	Plant Derived Pyrimidine Metabolism	punA->PGPT0013380	1
Indirect Effects	ColonizingPlant System	Colonization-Plant Derived Substrate Usage	Plant Derived Nucleoside Metabolism	Plant Derived Pyrimidine Metabolism	pyrB->PGPT0021160	1
Indirect Effects	ColonizingPlant System	Colonization-Plant Derived Substrate Usage	Plant Derived Nucleoside Metabolism	Plant Derived Pyrimidine Metabolism	pyrC->PGPT0021145	1
Indirect Effects	ColonizingPlant System	Colonization-Plant Derived Substrate Usage	Plant Derived Nucleoside Metabolism	Plant Derived Pyrimidine Metabolism	pyrDI->PGPT0021175	1
Indirect Effects	ColonizingPlant System	Colonization-Plant Derived Substrate Usage	Plant Derived Nucleoside Metabolism	Plant Derived Pyrimidine Metabolism	pyrDII->PGPT0021180	1
Indirect Effects	ColonizingPlant System	Colonization-Plant Derived Substrate Usage	Plant Derived Nucleoside Metabolism	Plant Derived Pyrimidine Metabolism	pyrE->PGPT0021200	1
Indirect Effects	ColonizingPlant System	Colonization-Plant Derived Substrate Usage	Plant Derived Nucleoside Metabolism	Plant Derived Pyrimidine Metabolism	pyrF->PGPT0014965	1
Indirect Effects	ColonizingPlant	Colonization-Plant Derived Substrate	Plant Derived Nucleoside	Plant Derived Pyrimidine	pyrG->PGPT0021215	1

	System	Usage	Metabolism	Metabolism		
Indirect Effects	ColonizingPlant System	Colonization-Plant Derived Substrate Usage	Plant Derived Nucleoside Metabolism	Plant Derived Pyrimidine Metabolism	pyrH->PGPT0021205	1
Indirect Effects	ColonizingPlant System	Colonization-Plant Derived Substrate Usage	Plant Derived Nucleoside Metabolism	Plant Derived Pyrimidine Metabolism	rutB->PGPT0021270	1
Indirect Effects	ColonizingPlant System	Colonization-Plant Derived Substrate Usage	Plant Derived Nucleoside Metabolism	Plant Derived Pyrimidine Metabolism	tdk->PGPT0021390	1
Indirect Effects	ColonizingPlant System	Colonization-Plant Derived Substrate Usage	Plant Derived Nucleoside Metabolism	Plant Derived Pyrimidine Metabolism	thyA->PGPT0008145	1
Indirect Effects	ColonizingPlant System	Colonization-Plant Derived Substrate Usage	Plant Derived Nucleoside Metabolism	Plant Derived Pyrimidine Metabolism	tmk->PGPT0021395	1
Indirect Effects	ColonizingPlant System	Colonization-Plant Derived Substrate Usage	Plant Derived Nucleoside Metabolism	Plant Derived Pyrimidine Metabolism	udk->PGPT0021230	1
Indirect Effects	ColonizingPlant System	Colonization-Plant Derived Substrate Usage	Plant Derived Nucleoside Metabolism	Plant Derived Pyrimidine Metabolism	udp->PGPT0021235	1
Indirect Effects	ColonizingPlant System	Colonization-Plant Derived Substrate Usage	Plant Derived Nucleoside Metabolism	Plant Derived Pyrimidine Metabolism	upp->PGPT0021240	1
Indirect Effects	ColonizingPlant System	Colonization-Plant Derived Substrate Usage	Plant Derived Nucleoside Metabolism	Plant Derived Pyrimidine Metabolism	ydfG->PGPT0021285	1
Indirect Effects	ColonizingPlant System	Colonization-Plant Derived Substrate Usage	Plant Derived Nucleoside Metabolism	Plant Derived Pyrimidine Metabolism	yfkN->PGPT0013420	1
Indirect	Colonizin	Colonization-Plant	Plant Derived	Plant Derived	ymdB->PGPT0021405	1

Effects	gPlant System	Derived Substrate Usage	Nucleoside Metabolism	Pyrimidine Metabolism		
Indirect Effects	Colonizin gPlant System	Colonization-Plant Derived Substrate Usage	Plant Derived Organic Acid transport	Plant Derived C4-Dicarboxyrate transport	dctA->PGPT0001450	2
Indirect Effects	Colonizin gPlant System	Colonization-Plant Derived Substrate Usage	Plant Derived Organic Acid transport	Plant Derived C4-Dicarboxyrate transport	dctP->PGPT0017325	1
Indirect Effects	Colonizin gPlant System	Colonization-Plant Derived Substrate Usage	Plant Derived Organic Acid transport	Plant Derived Citrate transport	TC CITMHS|CitMHS Family|citN->PGPT0001500	6
Indirect Effects	Colonizin gPlant System	Colonization-Plant Derived Substrate Usage	Plant Derived Organic Acid transport	Plant Derived Citrate transport	citS->PGPT0001490	2
Indirect Effects	Colonizin gPlant System	Colonization-Plant Derived Substrate Usage	Plant Derived Organic Acid transport	Plant Derived Citrate transport	fecB|htsA->PGPT0003800	1
Indirect Effects	Colonizin gPlant System	Colonization-Plant Derived Substrate Usage	Plant Derived Organic Acid transport	Plant Derived Citrate transport	fecC|htsB->PGPT0003805	2
Indirect Effects	Colonizin gPlant System	Colonization-Plant Derived Substrate Usage	Plant Derived Organic Acid transport	Plant Derived Citrate transport	fecD|htsC->PGPT0003810	2
Indirect Effects	Colonizin gPlant System	Colonization-Plant Derived Substrate Usage	Plant Derived Organic Acid transport	Plant Derived Citrate transport	fecE->PGPT0003815	1
Indirect Effects	Colonizin gPlant System	Colonization-Plant Derived Substrate Usage	Plant Derived Organic Acid transport	Plant Derived Formate transport	fdhC->PGPT0017240	1
Indirect Effects	Colonizin gPlant System	Colonization-Plant Derived Substrate Usage	Plant Derived Organic Acid transport	Plant Derived Formate transport	oxlT->PGPT0017235	1

Indirect Effects	ColonizingPlant System	Colonization-Plant Derived Substrate Usage	Plant Derived Organic Acid transport	Plant Derived Galactonate transport	dgoT->PGPT0002190	3
Indirect Effects	ColonizingPlant System	Colonization-Plant Derived Substrate Usage	Plant Derived Organic Acid transport	Plant Derived Gluconate transport	TC GNTP->PGPT0001340	4
Indirect Effects	ColonizingPlant System	Colonization-Plant Derived Substrate Usage	Plant Derived Organic Acid transport	Plant Derived Hexuronate transport	exuT->PGPT0017205	2
Indirect Effects	ColonizingPlant System	Colonization-Plant Derived Substrate Usage	Plant Derived Organic Acid transport	Plant Derived Keto-|OxoGlutarate transport	TC DASS|yflS->PGPT0013960	1
Indirect Effects	ColonizingPlant System	Colonization-Plant Derived Substrate Usage	Plant Derived Organic Acid transport	Plant Derived Keto-|OxoGlutarate transport	kgtP->PGPT0001525	1
Indirect Effects	ColonizingPlant System	Colonization-Plant Derived Substrate Usage	Plant Derived Organic Acid transport	Plant Derived KetoDeoxygluconate transport	kdgT->PGPT0017210	1
Indirect Effects	ColonizingPlant System	Colonization-Plant Derived Substrate Usage	Plant Derived Organic Acid transport	Plant Derived Lactate transport	lctP->PGPT0001800	2
Indirect Effects	ColonizingPlant System	Colonization-Plant Derived Substrate Usage	Plant Derived Organic Acid transport	Plant Derived Lactate transport	lldP|lctP->PGPT0001805	1
Indirect Effects	ColonizingPlant System	Colonization-Plant Derived Substrate Usage	Plant Derived Organic Acid transport	Plant Derived Malate transport	TC DASS|yflS->PGPT0013960	1
Indirect Effects	ColonizingPlant System	Colonization-Plant Derived Substrate Usage	Plant Derived Organic Acid transport	Plant Derived Malate transport	dctA->PGPT0001450	2
Indirect Effects	ColonizingPlant	Colonization-Plant Derived Substrate	Plant Derived Organic Acid transport	Plant Derived Malate transport	maeN->PGPT0001690	2

	System	Usage				
Indirect Effects	ColonizingPlant System	Colonization-Plant Derived Substrate Usage	Plant Derived Organic Acid transport	Plant Derived Malonate transport	mdcF->PGPT0001720	2
Indirect Effects	ColonizingPlant System	Colonization-Plant Derived Substrate Usage	Plant Derived Organic Acid transport	Plant Derived OligogalacTuronide transport I	TogT|rhiT->PGPT0017280	1
Indirect Effects	ColonizingPlant System	Colonization-Plant Derived Substrate Usage	Plant Derived Organic Acid transport	Plant Derived Oxalate transport	oxlT->PGPT0017235	1
Indirect Effects	ColonizingPlant System	Colonization-Plant Derived Substrate Usage	Plant Derived Organic Acid transport	Plant Derived Tricarboxylate transport	tctC->PGPT0017360	1
Indirect Effects	ColonizingPlant System	Colonization-Plant Derived Substrate Usage	Plant Derived Organic Acid Utilization	Plant Derived Acetate Utilization	acs->PGPT0002265	2
Indirect Effects	ColonizingPlant System	Colonization-Plant Derived Substrate Usage	Plant Derived Organic Acid Utilization	Plant Derived Aconitate Utilization	acnA->PGPT0001465	2
Indirect Effects	ColonizingPlant System	Colonization-Plant Derived Substrate Usage	Plant Derived Organic Acid Utilization	Plant Derived Ascorbate Utilization	araD|ulaF|sgaE|sgbE->PGPT0017420	1
Indirect Effects	ColonizingPlant System	Colonization-Plant Derived Substrate Usage	Plant Derived Organic Acid Utilization	Plant Derived Ascorbate Utilization	ulaG->PGPT0019580	1
Indirect Effects	ColonizingPlant System	Colonization-Plant Derived Substrate Usage	Plant Derived Organic Acid Utilization	Plant Derived Cinnamate Degradation	mhpC->PGPT0019765	2
Indirect Effects	ColonizingPlant System	Colonization-Plant Derived Substrate Usage	Plant Derived Organic Acid Utilization	Plant Derived Cinnamate Degradation	mhpE->PGPT0002050	1
Indirect	Colonizin	Colonization-Plant	Plant Derived Organic	Plant Derived Citrate	citR->PGPT0019505	1

Effects	gPlant System	Derived Substrate Usage	Acid Utilization	Sensing|Utilization		
Indirect Effects	Colonizin gPlant System	Colonization-Plant Derived Substrate Usage	Plant Derived Organic Acid Utilization	Plant Derived Citrate Sensing|Utilization	citS->PGPT0001490	2
Indirect Effects	Colonizin gPlant System	Colonization-Plant Derived Substrate Usage	Plant Derived Organic Acid Utilization	Plant Derived Citrate Sensing|Utilization	icd->PGPT0001170	1
Indirect Effects	Colonizin gPlant System	Colonization-Plant Derived Substrate Usage	Plant Derived Organic Acid Utilization	Plant Derived Erythronate Utilization	denD->PGPT0019550	1
Indirect Effects	Colonizin gPlant System	Colonization-Plant Derived Substrate Usage	Plant Derived Organic Acid Utilization	Plant Derived Erythronate Utilization	dtnK|denK->PGPT0018125	2
Indirect Effects	Colonizin gPlant System	Colonization-Plant Derived Substrate Usage	Plant Derived Organic Acid Utilization	Plant Derived Formate Utilization	fdoG|fdhF|fdwA->PGPT0019635	1
Indirect Effects	Colonizin gPlant System	Colonization-Plant Derived Substrate Usage	Plant Derived Organic Acid Utilization	Plant Derived Fumarate Sensing|Utilization	fumA|fumB->PGPT0001635	1
Indirect Effects	Colonizin gPlant System	Colonization-Plant Derived Substrate Usage	Plant Derived Organic Acid Utilization	Plant Derived Fumarate Sensing|Utilization	fumC->PGPT0001650	1
Indirect Effects	Colonizin gPlant System	Colonization-Plant Derived Substrate Usage	Plant Derived Organic Acid Utilization	Plant Derived Fumarate Sensing|Utilization	sdhA|frdA->PGPT0001605	1
Indirect Effects	Colonizin gPlant System	Colonization-Plant Derived Substrate Usage	Plant Derived Organic Acid Utilization	Plant Derived Fumarate Sensing|Utilization	sdhB|frdB->PGPT0001600	1
Indirect Effects	Colonizin gPlant System	Colonization-Plant Derived Substrate Usage	Plant Derived Organic Acid Utilization	Plant Derived Fumarate Sensing|Utilization	sdhC|frdC->PGPT0001595	1

Indirect Effects	ColonizingPlant System	Colonization-Plant Derived Substrate Usage	Plant Derived Organic Acid Utilization	Plant Derived Glutarate Utilization	korA|oorA|OforA->PGPT0001175	1
Indirect Effects	ColonizingPlant System	Colonization-Plant Derived Substrate Usage	Plant Derived Organic Acid Utilization	Plant Derived Glutarate Utilization	korB|oorB|OforB->PGPT0001180	1
Indirect Effects	ColonizingPlant System	Colonization-Plant Derived Substrate Usage	Plant Derived Organic Acid Utilization	Plant Derived Glutarate Utilization	sucA->PGPT0001530	1
Indirect Effects	ColonizingPlant System	Colonization-Plant Derived Substrate Usage	Plant Derived Organic Acid Utilization	Plant Derived Glutarate Utilization	sucB->PGPT0001535	2
Indirect Effects	ColonizingPlant System	Colonization-Plant Derived Substrate Usage	Plant Derived Organic Acid Utilization	Plant Derived Glyoxulate Utilization	ghrA->PGPT0019465	1
Indirect Effects	ColonizingPlant System	Colonization-Plant Derived Substrate Usage	Plant Derived Organic Acid Utilization	Plant Derived Indole-3-Acetate Degradation	aldH|dhaS->PGPT0006875	5
Indirect Effects	ColonizingPlant System	Colonization-Plant Derived Substrate Usage	Plant Derived Organic Acid Utilization	Plant Derived Malate Utilization	bshA->PGPT0019565	3
Indirect Effects	ColonizingPlant System	Colonization-Plant Derived Substrate Usage	Plant Derived Organic Acid Utilization	Plant Derived Malate Utilization	bshB1->PGPT0019570	2
Indirect Effects	ColonizingPlant System	Colonization-Plant Derived Substrate Usage	Plant Derived Organic Acid Utilization	Plant Derived Malate Utilization	bshB2->PGPT0019575	1
Indirect Effects	ColonizingPlant System	Colonization-Plant Derived Substrate Usage	Plant Derived Organic Acid Utilization	Plant Derived Malate Utilization	leuB->PGPT0001441	3
Indirect Effects	ColonizingPlant	Colonization-Plant Derived Substrate	Plant Derived Organic Acid Utilization	Plant Derived Malate Utilization	leuC->PGPT0001442	1

	System	Usage				
Indirect Effects	ColonizingPlant System	Colonization-Plant Derived Substrate Usage	Plant Derived Organic Acid Utilization	Plant Derived Malate Utilization	leuD->PGPT0001443	1
Indirect Effects	ColonizingPlant System	Colonization-Plant Derived Substrate Usage	Plant Derived Organic Acid Utilization	Plant Derived Malate Utilization	maeA|sfcA|ywkA->PGPT0001350	7
Indirect Effects	ColonizingPlant System	Colonization-Plant Derived Substrate Usage	Plant Derived Organic Acid Utilization	Plant Derived Malate Utilization	maeN->PGPT0001690	2
Indirect Effects	ColonizingPlant System	Colonization-Plant Derived Substrate Usage	Plant Derived Organic Acid Utilization	Plant Derived Malate Utilization	malK|yufL->PGPT0019555	4
Indirect Effects	ColonizingPlant System	Colonization-Plant Derived Substrate Usage	Plant Derived Organic Acid Utilization	Plant Derived Malate Utilization	malR->PGPT0019560	2
Indirect Effects	ColonizingPlant System	Colonization-Plant Derived Substrate Usage	Plant Derived Organic Acid Utilization	Plant Derived Malate Utilization	mdh->PGPT0001435	1
Indirect Effects	ColonizingPlant System	Colonization-Plant Derived Substrate Usage	Plant Derived Organic Acid Utilization	Plant Derived Malate Utilization	mqo->PGPT0001440	2
Indirect Effects	ColonizingPlant System	Colonization-Plant Derived Substrate Usage	Plant Derived Organic Acid Utilization	Plant Derived Malate Utilization	ttuC|dmlA->PGPT0001905	1
Indirect Effects	ColonizingPlant System	Colonization-Plant Derived Substrate Usage	Plant Derived Organic Acid Utilization	Plant Derived Malonate Utilization	mdcA->PGPT0019600	1
Indirect Effects	ColonizingPlant System	Colonization-Plant Derived Substrate Usage	Plant Derived Organic Acid Utilization	Plant Derived Malonate Utilization	mdcB->PGPT0019605	1
Indirect	Colonizin	Colonization-Plant	Plant Derived Organic	Plant Derived	mdcC->PGPT0019610	1

Effects	gPlant System	Derived Substrate Usage	Acid Utilization	Malonate Utilization		
Indirect Effects	Colonizin gPlant System	Colonization-Plant Derived Substrate Usage	Plant Derived Organic Acid Utilization	Plant Derived Malonate Utilization	mdcD->PGPT0019615	1
Indirect Effects	Colonizin gPlant System	Colonization-Plant Derived Substrate Usage	Plant Derived Organic Acid Utilization	Plant Derived Malonate Utilization	mdcG->PGPT0019625	1
Indirect Effects	Colonizin gPlant System	Colonization-Plant Derived Substrate Usage	Plant Derived Organic Acid Utilization	Plant Derived Malonate Utilization	mdcH->PGPT0019630	1
Indirect Effects	Colonizin gPlant System	Colonization-Plant Derived Substrate Usage	Plant Derived Organic Acid Utilization	Plant Derived Nicotinate Degradation	nicF->PGPT0019705	1
Indirect Effects	Colonizin gPlant System	Colonization-Plant Derived Substrate Usage	Plant Derived Organic Acid Utilization	Plant Derived Organic Acid Usage-TriCarboxylic Acid Cycle	CS|gltA->PGPT0001455	2
Indirect Effects	Colonizin gPlant System	Colonization-Plant Derived Substrate Usage	Plant Derived Organic Acid Utilization	Plant Derived Organic Acid Usage-TriCarboxylic Acid Cycle	aceF|pdhC->PGPT0001390	2
Indirect Effects	Colonizin gPlant System	Colonization-Plant Derived Substrate Usage	Plant Derived Organic Acid Utilization	Plant Derived Organic Acid Usage-TriCarboxylic Acid Cycle	acnA->PGPT0001465	2
Indirect Effects	Colonizin gPlant System	Colonization-Plant Derived Substrate Usage	Plant Derived Organic Acid Utilization	Plant Derived Organic Acid Usage-TriCarboxylic Acid Cycle	frdA->PGPT0001115	3
Indirect Effects	Colonizin gPlant	Colonization-Plant Derived Substrate	Plant Derived Organic Acid Utilization	Plant Derived Organic Acid Usage-	fumA|fumB->PGPT0001635	1

	System	Usage		TriCarboxylic Acid Cycle		
Indirect Effects	ColonizingPlant System	Colonization-Plant Derived Substrate Usage	Plant Derived Organic Acid Utilization	Plant Derived Organic Acid Usage-TriCarboxylic Acid Cycle	fumC->PGPT0001650	1
Indirect Effects	ColonizingPlant System	Colonization-Plant Derived Substrate Usage	Plant Derived Organic Acid Utilization	Plant Derived Organic Acid Usage-TriCarboxylic Acid Cycle	icd->PGPT0001170	1
Indirect Effects	ColonizingPlant System	Colonization-Plant Derived Substrate Usage	Plant Derived Organic Acid Utilization	Plant Derived Organic Acid Usage-TriCarboxylic Acid Cycle	korA|oorA|OforA->PGPT0001175	1
Indirect Effects	ColonizingPlant System	Colonization-Plant Derived Substrate Usage	Plant Derived Organic Acid Utilization	Plant Derived Organic Acid Usage-TriCarboxylic Acid Cycle	korB|oorB|OforB->PGPT0001180	1
Indirect Effects	ColonizingPlant System	Colonization-Plant Derived Substrate Usage	Plant Derived Organic Acid Utilization	Plant Derived Organic Acid Usage-TriCarboxylic Acid Cycle	lpd|pdhD->PGPT0001380	3
Indirect Effects	ColonizingPlant System	Colonization-Plant Derived Substrate Usage	Plant Derived Organic Acid Utilization	Plant Derived Organic Acid Usage-TriCarboxylic Acid Cycle	mdh->PGPT0001435	1
Indirect Effects	ColonizingPlant System	Colonization-Plant Derived Substrate Usage	Plant Derived Organic Acid Utilization	Plant Derived Organic Acid Usage-TriCarboxylic Acid Cycle	mqo->PGPT0001440	2
Indirect Effects	ColonizingPlant	Colonization-Plant Derived Substrate	Plant Derived Organic Acid Utilization	Plant Derived Organic Acid Usage-	pckA->PGPT0001405	1

	System	Usage		TriCarboxylic Acid Cycle		
Indirect Effects	ColonizingPlant System	Colonization-Plant Derived Substrate Usage	Plant Derived Organic Acid Utilization	Plant Derived Organic Acid Usage-TriCarboxylic Acid Cycle	pdhA->PGPT0018025	1
Indirect Effects	ColonizingPlant System	Colonization-Plant Derived Substrate Usage	Plant Derived Organic Acid Utilization	Plant Derived Organic Acid Usage-TriCarboxylic Acid Cycle	pdhB->PGPT0019590	1
Indirect Effects	ColonizingPlant System	Colonization-Plant Derived Substrate Usage	Plant Derived Organic Acid Utilization	Plant Derived Organic Acid Usage-TriCarboxylic Acid Cycle	pyc->PGPT0001420	1
Indirect Effects	ColonizingPlant System	Colonization-Plant Derived Substrate Usage	Plant Derived Organic Acid Utilization	Plant Derived Organic Acid Usage-TriCarboxylic Acid Cycle	sdhA|frdA->PGPT0001605	1
Indirect Effects	ColonizingPlant System	Colonization-Plant Derived Substrate Usage	Plant Derived Organic Acid Utilization	Plant Derived Organic Acid Usage-TriCarboxylic Acid Cycle	sdhB|frdB->PGPT0001600	1
Indirect Effects	ColonizingPlant System	Colonization-Plant Derived Substrate Usage	Plant Derived Organic Acid Utilization	Plant Derived Organic Acid Usage-TriCarboxylic Acid Cycle	sdhC|frdC->PGPT0001595	1
Indirect Effects	ColonizingPlant System	Colonization-Plant Derived Substrate Usage	Plant Derived Organic Acid Utilization	Plant Derived Organic Acid Usage-TriCarboxylic Acid Cycle	sucA->PGPT0001530	1
Indirect Effects	ColonizingPlant	Colonization-Plant Derived Substrate	Plant Derived Organic Acid Utilization	Plant Derived Organic Acid Usage-	sucB->PGPT0001535	2

	System	Usage		TriCarboxylic Acid Cycle		
Indirect Effects	ColonizingPlant System	Colonization-Plant Derived Substrate Usage	Plant Derived Organic Acid Utilization	Plant Derived Organic Acid Usage-TriCarboxylic Acid Cycle	sucC->PGPT0019515	1
Indirect Effects	ColonizingPlant System	Colonization-Plant Derived Substrate Usage	Plant Derived Organic Acid Utilization	Plant Derived Organic Acid Usage-TriCarboxylic Acid Cycle	sucD->PGPT0001540	1
Indirect Effects	ColonizingPlant System	Colonization-Plant Derived Substrate Usage	Plant Derived Organic Acid Utilization	Plant Derived Oxalic Acid Derivate Utilization	frc|yfdW->PGPT0002130	2
Indirect Effects	ColonizingPlant System	Colonization-Plant Derived Substrate Usage	Plant Derived Organic Acid Utilization	Plant Derived Oxalic Acid Derivate Utilization	gyaR->PGPT0019780	2
Indirect Effects	ColonizingPlant System	Colonization-Plant Derived Substrate Usage	Plant Derived Organic Acid Utilization	Plant Derived Salicylic Acid Degradation	bsdA->PGPT0019770	4
Indirect Effects	ColonizingPlant System	Colonization-Plant Derived Substrate Usage	Plant Derived Organic Acid Utilization	Plant Derived Salicylic Acid Degradation	bsdC->PGPT0005435	1
Indirect Effects	ColonizingPlant System	Colonization-Plant Derived Substrate Usage	Plant Derived Organic Acid Utilization	Plant Derived Salicylic Acid Degradation	ubiX|bsdB->PGPT0009565	1
Indirect Effects	ColonizingPlant System	Colonization-Plant Derived Substrate Usage	Plant Derived Organic Acid Utilization	Plant Derived Shikimate Degradation	aroB->PGPT0012875	2
Indirect Effects	ColonizingPlant System	Colonization-Plant Derived Substrate Usage	Plant Derived Organic Acid Utilization	Plant Derived Shikimate Degradation	aroL|aroK->PGPT0012900	1
Indirect	Colonizin	Colonization-Plant	Plant Derived Organic	Plant Derived	cyclohexadieny|preph	1

Effects	gPlant System	Derived Substrate Usage	Acid Utilization	Shikimate Degradation	enate Dehydrogenase|3 PhosphoShikimate 1 carboxyvinylTransferase->PGPT0012870	
Indirect Effects	Colonizin gPlant System	Colonization-Plant Derived Substrate Usage	Plant Derived Organic Acid Utilization	Plant Derived Shikimate Degradation	yddE->PGPT0012850	2
Indirect Effects	Colonizin gPlant System	Colonization-Plant Derived Substrate Usage	Plant Derived Organic Acid Utilization	Plant Derived Shikimate Degradation	ydiB->PGPT0019725	1
Indirect Effects	Colonizin gPlant System	Colonization-Plant Derived Substrate Usage	Plant Derived Organic Acid Utilization	Plant Derived Succinate Utilization	sdhA|frdA->PGPT0001605	1
Indirect Effects	Colonizin gPlant System	Colonization-Plant Derived Substrate Usage	Plant Derived Organic Acid Utilization	Plant Derived Succinate Utilization	sdhB|frdB->PGPT0001600	1
Indirect Effects	Colonizin gPlant System	Colonization-Plant Derived Substrate Usage	Plant Derived Organic Acid Utilization	Plant Derived Succinate Utilization	sdhC|frdC->PGPT0001595	1
Indirect Effects	Colonizin gPlant System	Colonization-Plant Derived Substrate Usage	Plant Derived Organic Acid Utilization	Plant Derived Succinate Utilization	sucA->PGPT0001530	1
Indirect Effects	Colonizin gPlant System	Colonization-Plant Derived Substrate Usage	Plant Derived Organic Acid Utilization	Plant Derived Succinate Utilization	sucC->PGPT0019515	1
Indirect Effects	Colonizin gPlant System	Colonization-Plant Derived Substrate Usage	Plant Derived Organic Acid Utilization	Plant Derived Succinate Utilization	sucD->PGPT0001540	1
Indirect Effects	Colonizin gPlant System	Colonization-Plant Derived Substrate Usage	Plant Derived Organic Acid Utilization	Plant Derived Tartrate Utilization	ttuC|dmlA->PGPT0001905	1

Indirect Effects	ColonizingPlant System	Colonization-Plant Derived Substrate Usage	Plant Derived Organic Acid Utilization	Plant Derived Taurine Utilization	ggt->PGPT0002935	6
Indirect Effects	ColonizingPlant System	Colonization-Plant Derived Substrate Usage	Plant Derived Sugar Acid Utilization	Plant Derived Galactarate|Glucarate Degradation	garD->PGPT0018155	1
Indirect Effects	ColonizingPlant System	Colonization-Plant Derived Substrate Usage	Plant Derived Sugar Acid Utilization	Plant Derived Galactarate|Glucarate Degradation	garK|glxK->PGPT0018175	1
Indirect Effects	ColonizingPlant System	Colonization-Plant Derived Substrate Usage	Plant Derived Sugar Acid Utilization	Plant Derived Galactarate|Glucarate Degradation	garR|glxR->PGPT0018170	4
Indirect Effects	ColonizingPlant System	Colonization-Plant Derived Substrate Usage	Plant Derived Sugar Acid Utilization	Plant Derived Galactarate|Glucarate Degradation	gudD->PGPT0018180	1
Indirect Effects	ColonizingPlant System	Colonization-Plant Derived Substrate Usage	Plant Derived Sugar Acid Utilization	Plant Derived Galactarate|Glucarate Degradation	kdgD|ycbC->PGPT0018160	1
Indirect Effects	ColonizingPlant System	Colonization-Plant Derived Substrate Usage	Plant Derived Sugar Acid Utilization	Plant Derived Galactonate Degradation	kdgK->PGPT0018060	1
Indirect Effects	ColonizingPlant System	Colonization-Plant Derived Substrate Usage	Plant Derived Sugar Acid Utilization	Plant Derived Galacturonate|Tagaturonate|Altronate Degradation	eda->PGPT0002060	1
Indirect Effects	ColonizingPlant System	Colonization-Plant Derived Substrate Usage	Plant Derived Sugar Acid Utilization	Plant Derived Galacturonate|Tagaturonate|Altronate Degradation	kdgK->PGPT0018060	1
Indirect Effects	ColonizingPlant System	Colonization-Plant Derived Substrate Usage	Plant Derived Sugar Acid Utilization	Plant Derived Galacturonate|Tagaturonate|Altronate	uxaA->PGPT0018305	1

				Degradation		
Indirect Effects	ColonizingPlant System	Colonization-Plant Derived Substrate Usage	Plant Derived Sugar Acid Utilization	Plant Derived Galacturonate|Tagaturonate|Altronate Degradation	uxaB->PGPT0018320	1
Indirect Effects	ColonizingPlant System	Colonization-Plant Derived Substrate Usage	Plant Derived Sugar Acid Utilization	Plant Derived Galacturonate|Tagaturonate|Altronate Degradation	uxaC->PGPT0018210	1
Indirect Effects	ColonizingPlant System	Colonization-Plant Derived Substrate Usage	Plant Derived Sugar Acid Utilization	Plant Derived Glacturonate|Glucuronate Degradation	kdgD|ycbC->PGPT0018160	1
Indirect Effects	ColonizingPlant System	Colonization-Plant Derived Substrate Usage	Plant Derived Sugar Acid Utilization	Plant Derived Gluconate Metabolism	eda->PGPT0002060	1
Indirect Effects	ColonizingPlant System	Colonization-Plant Derived Substrate Usage	Plant Derived Sugar Acid Utilization	Plant Derived Gluconate Metabolism	gdh|ycdF->PGPT0014705	7
Indirect Effects	ColonizingPlant System	Colonization-Plant Derived Substrate Usage	Plant Derived Sugar Acid Utilization	Plant Derived Gluconate Metabolism	gnd|gntZ->PGPT0017385	4
Indirect Effects	ColonizingPlant System	Colonization-Plant Derived Substrate Usage	Plant Derived Sugar Acid Utilization	Plant Derived Gluconate Metabolism	idnO->PGPT0018070	1
Indirect Effects	ColonizingPlant System	Colonization-Plant Derived Substrate Usage	Plant Derived Sugar Acid Utilization	Plant Derived Gluconate Metabolism	kdgK->PGPT0018060	1
Indirect Effects	ColonizingPlant System	Colonization-Plant Derived Substrate Usage	Plant Derived Sugar Acid Utilization	Plant Derived Gluconate Metabolism	kduD->PGPT0018065	1
Indirect Effects	ColonizingPlant	Colonization-Plant Derived Substrate	Plant Derived Sugar Acid Utilization	Plant Derived Gluconate Metabolism	ykgB|pgl->PGPT0014975	2

	System	Usage				
Indirect Effects	ColonizingPlant System	Colonization-Plant Derived Substrate Usage	Plant Derived Sugar Acid Utilization	Plant Derived Gluconate Metabolism	zwf->PGPT0017380	4
Indirect Effects	ColonizingPlant System	Colonization-Plant Derived Substrate Usage	Plant Derived Sugar Acid Utilization	Plant Derived Glucuronate Degradation	uxaC->PGPT0018210	1
Indirect Effects	ColonizingPlant System	Colonization-Plant Derived Substrate Usage	Plant Derived Sugar Acid Utilization	Plant Derived Gulonate Degradation	araD|ulaF|sgaE|sgbE->PGPT0017420	1
Indirect Effects	ColonizingPlant System	Colonization-Plant Derived Substrate Usage	Plant Derived Sugar Acid Utilization	Plant Derived Gulonate|Fructuronate|Mannonate Degradation	uxaC->PGPT0018210	1
Indirect Effects	ColonizingPlant System	Colonization-Plant Derived Substrate Usage	Plant Derived Sugar Acid Utilization	Plant Derived Hexonate|Hexuronate|Hexuronide Utilization	exuR->PGPT0018214	1
Indirect Effects	ColonizingPlant System	Colonization-Plant Derived Substrate Usage	Plant Derived Sugar Acid Utilization	Plant Derived Hexonate|Hexuronate|Hexuronide Utilization	kdgR->PGPT0018213	3
Indirect Effects	ColonizingPlant System	Colonization-Plant Derived Substrate Usage	Plant Derived Sugar Acid Utilization	Plant Derived Idonate Degradation	idnO->PGPT0018070	1
Indirect Effects	ColonizingPlant System	Colonization-Plant Derived Substrate Usage	Plant Derived Sugar Acid Utilization	Plant Derived Lactate Degradation	lldE|ykgE|lutA->PGPT0018090	1
Indirect Effects	ColonizingPlant System	Colonization-Plant Derived Substrate Usage	Plant Derived Sugar Acid Utilization	Plant Derived Lactate Degradation	lldF|ykgF|lutB->PGPT0018095	3
Indirect	Colonizin	Colonization-Plant	Plant Derived Sugar	Plant Derived Lactate	lldG|ykgG|lutC-	1

Effects	gPlant System	Derived Substrate Usage	Acid Utilization	Degradation	>PGPT0018100	
Indirect Effects	Colonizin gPlant System	Colonization-Plant Derived Substrate Usage	Plant Derived Sugar Acid Utilization	Plant Derived Lactate Degradation	lldR->PGPT0018120	2
Indirect Effects	Colonizin gPlant System	Colonization-Plant Derived Substrate Usage	Plant Derived Sugar Acid Utilization	Plant Derived OligogalacTuronide Degradation	kduD->PGPT0018065	1
Indirect Effects	Colonizin gPlant System	Colonization-Plant Derived Substrate Usage	Plant Derived Sugar Acid Utilization	Plant Derived OligogalacTuronide Degradation	yteR|yesR->PGPT0018255	1
Indirect Effects	Colonizin gPlant System	Colonization-Plant Derived Substrate Usage	Plant Derived Sugar Acid Utilization	Plant Derived Threonate Degradation	dtnK|denK->PGPT0018125	2
Indirect Effects	Colonizin gPlant System	Colonization-Plant Derived Substrate Usage	Plant Derived Sugar Alcohol Utilization	Plant Derived D-Erythrulose|D-Threitol Degradation	rpiB->PGPT0017395	2
Indirect Effects	Colonizin gPlant System	Colonization-Plant Derived Substrate Usage	Plant Derived Sugar Alcohol Utilization	Plant Derived ErythritoL Degradation	rpiB->PGPT0017395	2
Indirect Effects	Colonizin gPlant System	Colonization-Plant Derived Substrate Usage	Plant Derived Sugar Alcohol Utilization	Plant Derived Glycerol|Glycerophos phodiester Metabolism|Degradati on	dhaK 2->PGPT0018465	1
Indirect Effects	Colonizin gPlant System	Colonization-Plant Derived Substrate Usage	Plant Derived Sugar Alcohol Utilization	Plant Derived Glycerol|Glycerophos phodiester Metabolism|Degradati on	gldA|dhaD->PGPT0008270	1
Indirect Effects	Colonizin gPlant	Colonization-Plant Derived Substrate	Plant Derived Sugar Alcohol Utilization	Plant Derived Glycerol|Glycerophos	glpA|glpD->PGPT0006775	1

	System	Usage		phodiester Metabolism|Degradation		
Indirect Effects	ColonizingPlant System	Colonization-Plant Derived Substrate Usage	Plant Derived Sugar Alcohol Utilization	Plant Derived Glycerol|Glycerophosphodiester Metabolism|Degradation	glpK->PGPT0018435	1
Indirect Effects	ColonizingPlant System	Colonization-Plant Derived Substrate Usage	Plant Derived Sugar Alcohol Utilization	Plant Derived Glycerol|Glycerophosphodiester Metabolism|Degradation	glpQ|ugpQ->PGPT0018470	5
Indirect Effects	ColonizingPlant System	Colonization-Plant Derived Substrate Usage	Plant Derived Sugar Alcohol Utilization	Plant Derived Inositol Derivate Degradation	iolB->PGPT0018475	2
Indirect Effects	ColonizingPlant System	Colonization-Plant Derived Substrate Usage	Plant Derived Sugar Alcohol Utilization	Plant Derived Inositol Derivate Degradation	iolC->PGPT0018480	2
Indirect Effects	ColonizingPlant System	Colonization-Plant Derived Substrate Usage	Plant Derived Sugar Alcohol Utilization	Plant Derived Inositol Derivate Degradation	iolD->PGPT0018485	2
Indirect Effects	ColonizingPlant System	Colonization-Plant Derived Substrate Usage	Plant Derived Sugar Alcohol Utilization	Plant Derived Inositol Derivate Degradation	iolI->PGPT0018495	1
Indirect Effects	ColonizingPlant System	Colonization-Plant Derived Substrate Usage	Plant Derived Sugar Alcohol Utilization	Plant Derived Inositol Derivate Degradation	iolJ->PGPT0018500	1
Indirect Effects	ColonizingPlant System	Colonization-Plant Derived Substrate Usage	Plant Derived Sugar Alcohol Utilization	Plant Derived Inositol Derivate Degradation	iolS->PGPT0018520	2
Indirect	Colonizin	Colonization-Plant	Plant Derived Sugar	Plant Derived Inositol	iolW->PGPT0018530	1

Effects	gPlant System	Derived Substrate Usage	Alcohol Utilization	Derivate Degradation		
Indirect Effects	Colonizin gPlant System	Colonization-Plant Derived Substrate Usage	Plant Derived Sugar Alcohol Utilization	Plant Derived Inositol Derivate Degradation	iolX->PGPT0018525	3
Indirect Effects	Colonizin gPlant System	Colonization-Plant Derived Substrate Usage	Plant Derived Sugar Alcohol Utilization	Plant Derived Inositol Derivate Degradation	mmsA|iolA->PGPT0002295	4
Indirect Effects	Colonizin gPlant System	Colonization-Plant Derived Substrate Usage	Plant Derived Sugar Alcohol Utilization	Plant Derived Inositol Derivate Degradation	mocC->PGPT0016785	3
Indirect Effects	Colonizin gPlant System	Colonization-Plant Derived Substrate Usage	Plant Derived Sugar Alcohol Utilization	Plant Derived Inositol Derivate Degradation	mocR->PGPT0016790	3
Indirect Effects	Colonizin gPlant System	Colonization-Plant Derived Substrate Usage	Plant Derived Sugar Alcohol Utilization	Plant Derived Inositol Derivate Degradation	suhB->PGPT0018535	1
Indirect Effects	Colonizin gPlant System	Colonization-Plant Derived Substrate Usage	Plant Derived Sugar Alcohol Utilization	Plant Derived Mannitol Degradation	mtlA|cmtA->PGPT0016945	1
Indirect Effects	Colonizin gPlant System	Colonization-Plant Derived Substrate Usage	Plant Derived Sugar Alcohol Utilization	Plant Derived Mannitol Degradation	mtlD->PGPT0013700	1
Indirect Effects	Colonizin gPlant System	Colonization-Plant Derived Substrate Usage	Plant Derived Sugar Alcohol Utilization	Plant Derived Sorbitol Degradation	hxpB->PGPT0018430	1
Indirect Effects	Colonizin gPlant System	Colonization-Plant Derived Substrate Usage	Plant Derived Terpene Utilization	Plant Derived Citronellol-Citronellal-Citronellate Degradation	atuB|atuG->PGPT0019825	1
Indirect Effects	Colonizin gPlant	Colonization-Plant Derived Substrate	Plant Derived Terpene Utilization	Plant Derived Citronellol-Citronellal-	hmgL->PGPT0008315	2

	System	Usage		Citronellate Degradation		
Indirect Effects	ColonizingPlant System	Colonization-Plant Derived Substrate Usage	Plant Derived trigonelline Usage	Plant Derived trigonelline Degradation	gabD->PGPT0001580	5
Indirect Effects	ColonizingPlant System	Colonization-Plant Derived Substrate Usage	Plant Derived trigonelline Usage	Plant Derived trigonelline Degradation	tgnB->PGPT0000910	1
Indirect Effects	ColonizingPlant System	Colonization-Plant Derived Substrate Usage	Plant Derived trigonelline Usage	Plant Derived trigonelline Degradation	tgnR->PGPT0000900	3
Indirect Effects	ColonizingPlant System	Colonization-Surface Attachment	Surface Adhesion	Other Surface Adhesion Proteins	yfiQ->PGPT0022170	1
Indirect Effects	ColonizingPlant System	Colonization-Surface Attachment	Surface Adhesion	Other Surface Adhesion Proteins	ywsB->PGPT0022161	1
Indirect Effects	ColonizingPlant System	Colonization-Surface Attachment	Surface Attachment-Lipo-|Colanic Acid Metabolism	Surface Attachment-Capsular Metabolism	TC GPH|yihO|xynP->PGPT0014410	3
Indirect Effects	ColonizingPlant System	Colonization-Surface Attachment	Surface Attachment-Lipo-|Colanic Acid Metabolism	Surface Attachment-Colanic Acid Metabolism	manC|cpsB->PGPT0022660	1
Indirect Effects	ColonizingPlant System	Colonization-Surface Attachment	Surface Attachment-Lipo-|Teichoic Acid Metabolism	Surface Attachment-Lipoteichoic Acid Metabolism	dltE->PGPT0013220	1
Indirect Effects	ColonizingPlant System	Colonization-Surface Attachment	Surface Attachment-Lipo-|Teichoic Acid Metabolism	Surface Attachment-Lipoteichoic Acid Metabolism	ltaS->PGPT0023375	3
Indirect Effects	ColonizingPlant System	Colonization-Surface Attachment	Surface Attachment-Lipo-|Teichoic Acid Metabolism	Surface Attachment-Lipoteichoic Acid Metabolism	ugtP->PGPT0023400	2

Indirect Effects	ColonizingPlant System	Colonization-Surface Attachment	Surface Attachment-Lipo-|Teichoic Acid Metabolism	Surface Attachment-Teichoic Acid Metabolism	fmtA->PGPT0023445	1
Indirect Effects	ColonizingPlant System	Colonization-Surface Attachment	Surface Attachment-Lipo-|Teichoic Acid Metabolism	Surface Attachment-Teichoic Acid Metabolism	gtaC->PGPT0023460	2
Indirect Effects	ColonizingPlant System	Colonization-Surface Attachment	Surface Attachment-Lipo-|Teichoic Acid Metabolism	Surface Attachment-Teichoic Acid Metabolism	Taga|tarA->PGPT0023455	2
Indirect Effects	ColonizingPlant System	Colonization-Surface Attachment	Surface Attachment-Lipo-|Teichoic Acid Metabolism	Surface Attachment-Teichoic Acid Metabolism	tagT|tagU|tagV->PGPT0023440	7
Indirect Effects	ColonizingPlant System	Colonization-Surface Attachment	Surface Attachment-Lipo-|Teichoic Acid Metabolism	Surface Attachment-Teichoic Acid Metabolism	wecB->PGPT0018905	2
Indirect Effects	ColonizingPlant System	Colonization-Surface Attachment	Surface Attachment-Lipo-|Teichuronic Acid Metabolism	Surface Attachment-Teichuronic Acid Metabolism	tuaG|ggaB->PGPT0015205	2
Indirect Effects	ColonizingPlant System	Colonization-Surface Attachment	Surface Attachment-Lipo-|Teichuronic Acid Metabolism	Surface Attachment-Teichuronic Acid Metabolism	wecA|tagO|rfe->PGPT0015240	1
Indirect Effects	ColonizingPlant System	Colonization-Surface Attachment	Surface Attachment-LPS|Lipid|IVA Metabolism	Surface Attachment-Lipid A Metabolism	lpxG->PGPT0022365	3
Indirect Effects	ColonizingPlant System	Colonization-Surface Attachment	Surface Attachment-LPS|Lipid|IVA Metabolism	Surface Attachment-LPS-GDP D-Rhamnose|Deoxy D Mannose Modification	rmd->PGPT0022780	1
Indirect Effects	ColonizingPlant System	Colonization-Surface Attachment	Surface Attachment-LPS|Lipid|IVA Metabolism	Surface Attachment-LPS-GDP Mannose Modification	manC|cpsB->PGPT0022660	1
Indirect	Colonizin	Colonization-Surface	Surface Attachment-	Surface Attachment-	glmU->PGPT0018955	1

Effects	gPlant System	Attachment	LPS|Lipid|IVA Metabolism	LPS-Glucosamine Modification		
Indirect Effects	Colonizin gPlant System	Colonization-Surface Attachment	Surface Attachment-LPS|Lipid|IVA Metabolism	Surface Attachment-LPS-Glucose-Mannose-Frucose Conversion	manA->PGPT0017860	1
Indirect Effects	Colonizin gPlant System	Colonization-Surface Attachment	Surface Attachment-LPS|Lipid|IVA Metabolism	Surface Attachment-LPS-Manno-Heptose|-Phosphate Modification	gmhB|yaeD->PGPT0023035	1
Indirect Effects	Colonizin gPlant System	Colonization-Surface Attachment	Surface Attachment-LPS|Lipid|IVA Metabolism	Surface Attachment-LPS-O-Antiogen Biosynthesis	csbB|gtrB|yfdH->PGPT0014540	4
Indirect Effects	Colonizin gPlant System	Colonization-Surface Attachment	Surface Attachment-LPS|Lipid|IVA Metabolism	Surface Attachment-LPS-O-Antiogen Biosynthesis	wecA|tagO|rfe->PGPT0015240	1
Indirect Effects	Colonizin gPlant System	Colonization-Surface Attachment	Surface Attachment-LPS|Lipid|IVA Metabolism	Surface Attachment-LPS-O-Antiogen Biosynthesis	wecB->PGPT0018905	2
Indirect Effects	Colonizin gPlant System	Colonization-Surface Attachment	Surface Attachment-LPS|Lipid|IVA Metabolism	Surface Attachment-LPS-O-Antiogen Biosynthesis	wecC->PGPT0022670	1
Indirect Effects	Colonizin gPlant System	Colonization-Surface Attachment	Surface Attachment-LPS|Lipid|IVA Metabolism	Surface Attachment-LPS-Pseudaminic Acid Modification	pseB|wbjB->PGPT0018935	1
Indirect Effects	Colonizin gPlant System	Colonization-Surface Attachment	Surface Attachment-LPS|Lipid|IVA Metabolism	Surface Attachment-LPS-Putative Trylose Modification	rfbD|rmlD->PGPT0022630	1
Indirect Effects	Colonizin gPlant System	Colonization-Surface Attachment	Surface Attachment-LPS|Lipid|IVA Metabolism	Surface Attachment-LPS-UDP-Galactose-Glucuronate Modification	cap1J|wbgU->PGPT0019020	1

Indirect Effects	ColonizingPlant System	Colonization-Surface Attachment	Surface Attachment-LPS|Lipid|IVA Metabolism	Surface Attachment-LPS D Galactofuranose Modification	galT->PGPT0017835	1
Indirect Effects	ColonizingPlant System	Colonization-Surface Attachment	Surface Attachment-LPS|Lipid|IVA Metabolism	Surface Attachment-LPS D Mannosaminuronic Acid Modification	wecB->PGPT0018905	2
Indirect Effects	ColonizingPlant System	Colonization-Surface Attachment	Surface Attachment-LPS|Lipid|IVA Metabolism	Surface Attachment-LPS D Mannosaminuronic Acid Modification	wecC->PGPT0022670	1
Indirect Effects	ColonizingPlant System	Colonization-Surface Attachment	Surface Attachment-LPS|Lipid|IVA Metabolism	Surface Attachment-LPS D Mannuronic Acid Modification	wbpA->PGPT0018920	1
Indirect Effects	ColonizingPlant System	Colonization-Surface Attachment	Surface Attachment-LPS|Lipid|IVA Metabolism	Surface Attachment-LPS D Mannuronic Acid Modification	wlbA|bplA->PGPT0022715	3
Indirect Effects	ColonizingPlant System	Colonization-Surface Attachment	Surface Attachment-LPS|Lipid|IVA Metabolism	Surface Attachment-LPS L Ara4N Modification	arnE->PGPT0022825	1
Indirect Effects	ColonizingPlant System	Colonization-Surface Attachment	Surface Attachment-Poly-N-Acetyl-Glucosamine Metabolism	Surface Attachment-Poly-N Acetyl-Glucosamine Biosynthesis	pgaC|icaA->PGPT0023485	3
Indirect Effects	ColonizingPlant System	Other Colonization Related Proteins	Colonization-Glycogen Metabolism	Colonization-Glycogen Biosynthesis	glgA->PGPT0025880	1
Indirect Effects	ColonizingPlant System	Other Colonization Related Proteins	Colonization-Glycogen Metabolism	Colonization-Glycogen Biosynthesis	glgC->PGPT0025885	2
Indirect	Colonizin	Other Colonization	Colonization-	Colonization-	glgP->PGPT0018545	1

Effects	gPlant System	Related Proteins	Glycogen Metabolism	Glycogen Biosynthesis		
Indirect Effects	Colonizin gPlant System	Other Colonization Related Proteins	Colonization-Glycogen Metabolism	Colonization-Glycogen Biosynthesis	yqgM->PGPT0018547	2
Indirect Effects	Colonizin gPlant System	Other Colonization Related Proteins	Colonization-Host Invasion Factors	Host Invasion-GGDEF|EAL|PAC|PAS-Domain-Containing Protein	GGDEF|EAL|PAC|PAS Domain Containing Protein->PGPT0023624	4
Indirect Effects	Colonizin gPlant System	Other Colonization Related Proteins	Colonization-Host Invasion Factors	Host Invasion-Host Infection Mediator	miaA|ipt->PGPT0007210	1
Indirect Effects	Colonizin gPlant System	Other Colonization Related Proteins	Colonization-Host Invasion Factors	Host Invasion-Host Infection Mediator	typA|bipA->PGPT0023720	1
Indirect Effects	Colonizin gPlant System	Other Colonization Related Proteins	Colonization-Host Invasion Factors	Host Invasion-Host Infection Mediator	yajQ->PGPT0012210	1
Indirect Effects	Colonizin gPlant System	Other Colonization Related Proteins	Colonization-Host Invasion Factors	Host Invasion-Phosphatidylcholine Biosynthesis	pmtA->PGPT0007740	3
Indirect Effects	Colonizin gPlant System	Other Colonization Related Proteins	Colonization-Host Invasion Factors	Host Invasion-SAM|Queuosine|Lipid Metabolism	mtaD->PGPT0023665	1
Indirect Effects	Colonizin gPlant System	Other Colonization Related Proteins	Colonization-Host Invasion Factors	Host Invasion-SAM|Queuosine|Lipid Metabolism	pmtA->PGPT0007740	3
Indirect Effects	Colonizin gPlant System	Other Colonization Related Proteins	Colonization-Host Invasion Factors	Host Invasion-SAM|Queuosine|Lipid Metabolism	queA->PGPT0023660	1
Indirect Effects	Colonizin gPlant	Other Colonization Related Proteins	Colonization-Host Invasion Factors	Host Invasion-SAM|Queuosine|Lipi	smtA->PGPT0023655	2

	System			d Metabolism		
Indirect Effects	Colonizin gPlant System	Root Colonization	Root Colonization-Vitamin B3|Niacin Metabolism	Root Colonization-Vitamin B3|Niacin Biosynthesis	deoD->PGPT0013385	1
Indirect Effects	Colonizin gPlant System	Root Colonization	Root Colonization-Vitamin B3|Niacin Metabolism	Root Colonization-Vitamin B3|Niacin Biosynthesis	iunH->PGPT0013465	2
Indirect Effects	Colonizin gPlant System	Root Colonization	Root Colonization-Vitamin B3|Niacin Metabolism	Root Colonization-Vitamin B3|Niacin Biosynthesis	nadA->PGPT0013365	1
Indirect Effects	Colonizin gPlant System	Root Colonization	Root Colonization-Vitamin B3|Niacin Metabolism	Root Colonization-Vitamin B3|Niacin Biosynthesis	nadB->PGPT0013355	1
Indirect Effects	Colonizin gPlant System	Root Colonization	Root Colonization-Vitamin B3|Niacin Metabolism	Root Colonization-Vitamin B3|Niacin Biosynthesis	nadC->PGPT0013370	1
Indirect Effects	Colonizin gPlant System	Root Colonization	Root Colonization-Vitamin B3|Niacin Metabolism	Root Colonization-Vitamin B3|Niacin Biosynthesis	nadD->PGPT0013435	3
Indirect Effects	Colonizin gPlant System	Root Colonization	Root Colonization-Vitamin B3|Niacin Metabolism	Root Colonization-Vitamin B3|Niacin Biosynthesis	nadE->PGPT0013445	2
Indirect Effects	Colonizin gPlant System	Root Colonization	Root Colonization-Vitamin B3|Niacin Metabolism	Root Colonization-Vitamin B3|Niacin Biosynthesis	npdA->PGPT0013485	1
Indirect Effects	Colonizin gPlant System	Root Colonization	Root Colonization-Vitamin B3|Niacin Metabolism	Root Colonization-Vitamin B3|Niacin Biosynthesis	pncA->PGPT0013470	2
Indirect Effects	Colonizin gPlant System	Root Colonization	Root Colonization-Vitamin B3|Niacin Metabolism	Root Colonization-Vitamin B3|Niacin Biosynthesis	pncB->PGPT0013375	2
Indirect	Colonizin	Root Colonization	Root Colonization-	Root Colonization-	pncC->PGPT0013455	1

Effects	gPlant System		Vitamin B3|Niacin Metabolism	Vitamin B3|Niacin Biosynthesis		
Indirect Effects	Colonizin gPlant System	Root Colonization	Root Colonization-Vitamin B3|Niacin Metabolism	Root Colonization-Vitamin B3|Niacin Biosynthesis	punA->PGPT0013380	1
Indirect Effects	Colonizin gPlant System	Root Colonization	Root Colonization-Vitamin B3|Niacin Metabolism	Root Colonization-Vitamin B3|Niacin Biosynthesis	yfkN->PGPT0013420	1
Indirect Effects	Colonizin gPlant System	Root Colonization	Root Colonization-Vitamin B7|iotin Metabolism	Root Colonization-Vitamin B7|iotin Biosynthesis	accA->PGPT0001695	1
Indirect Effects	Colonizin gPlant System	Root Colonization	Root Colonization-Vitamin B7|iotin Metabolism	Root Colonization-Vitamin B7|iotin Biosynthesis	accB|bccP->PGPT0001700	3
Indirect Effects	Colonizin gPlant System	Root Colonization	Root Colonization-Vitamin B7|iotin Metabolism	Root Colonization-Vitamin B7|iotin Biosynthesis	bioA->PGPT0022100	1
Indirect Effects	Colonizin gPlant System	Root Colonization	Root Colonization-Vitamin B7|iotin Metabolism	Root Colonization-Vitamin B7|iotin Biosynthesis	bioB->PGPT0022120	1
Indirect Effects	Colonizin gPlant System	Root Colonization	Root Colonization-Vitamin B7|iotin Metabolism	Root Colonization-Vitamin B7|iotin Biosynthesis	bioC->PGPT0022060	1
Indirect Effects	Colonizin gPlant System	Root Colonization	Root Colonization-Vitamin B7|iotin Metabolism	Root Colonization-Vitamin B7|iotin Biosynthesis	biOf->PGPT0022095	1
Indirect Effects	Colonizin gPlant System	Root Colonization	Root Colonization-Vitamin B7|iotin Metabolism	Root Colonization-Vitamin B7|iotin Biosynthesis	bioH->PGPT0022065	1
Indirect Effects	Colonizin gPlant System	Root Colonization	Root Colonization-Vitamin B7|iotin Metabolism	Root Colonization-Vitamin B7|iotin Biosynthesis	birA|bpr->PGPT0022055	1

Indirect Effects	Colonizin gPlant System	Root Colonization	Root Colonization-Vitamin B7∣iotin Metabolism	Root Colonization-Vitamin B7∣iotin Biosynthesis	fabF->PGPT0008360	1
Indirect Effects	Colonizin gPlant System	Root Colonization	Root Colonization-Vitamin B7∣iotin Metabolism	Root Colonization-Vitamin B7∣iotin Biosynthesis	fabH->PGPT0008355	4
Indirect Effects	Colonizin gPlant System	Root Colonization	Root Colonization-Vitamin B7∣iotin Metabolism	Root Colonization-Vitamin B7∣iotin Biosynthesis	fabI->PGPT0008370	1
Indirect Effects	Colonizin gPlant System	Root Colonization	Root Colonization-Vitamin B7∣iotin Metabolism	Root Colonization-Vitamin B7∣iotin Biosynthesis	fabZ->PGPT0008365	3
Indirect Effects	Colonizin gPlant System	Root Colonization	Root Colonization-Vitamin B7∣iotin Metabolism	Root Colonization-Vitamin B7∣iotin Biosynthesis	fadD->PGPT0008380	6
Indirect Effects	Colonizin gPlant System	Root Colonization	Root Colonization-Vitamin B7∣iotin Metabolism	Root Colonization-Vitamin B7∣iotin Biosynthesis	ymfI∣fabG∣efpI->PGPT0003180	14
Indirect Effects	Colonizin gPlant System	Root Colonization	Root Colonization-Vitamin B7∣iotin Metabolism	Root Colonization-Vitamin B7∣iotin transport	bioY->PGPT0022050	3
Indirect Effects	Colonizin gPlant System	Root Colonization	Root Colonization By Nodulation	Root Nodulation Metabolism	glmS∣nodM->PGPT0017630	1
Indirect Effects	Colonizin gPlant System	Root Colonization	Root Colonization By Nodulation	Root Nodulation Metabolism	maa∣nodL->PGPT0018611	1
Indirect Effects	Colonizin gPlant System	Root Colonization	Root Colonization By Nodulation	Root Nodule Regulation	glpA∣glpD->PGPT0006775	1
Indirect Effects	Colonizin gPlant	Root Colonization	Root Colonization By Nodulation	Root Nodule Regulation	glpK->PGPT0018435	1

	System					
Indirect Effects	ColonizingPlant System	Root Colonization	Root Colonization By Nodulation	Root Nodule Regulation	nodX->PGPT0022030	2
Indirect Effects	ColonizingPlant System	Root Colonization	Root Colonization Facilitating Protein	Root Colonization-Levan Metabolism	sacB->PGPT0013775	2
Indirect Effects	ColonizingPlant System	Root Colonization	Root Colonization Facilitating Protein	Root Colonization-Site-Specific Recombination	xerC->PGPT0021995	1
Indirect Effects	ColonizingPlant System	Root Colonization	Root Colonization Facilitating Protein	Root Colonization-Site-Specific Recombination	xerD->PGPT0022000	8
Indirect Effects	ColonizingPlant System	Root Colonization	Root Colonization Facilitating Protein	Root Colonization-Zinc transport Lipoprotein	znuA->PGPT0004220	1
Indirect Effects	ColonizingPlant System	Root Colonization	Root Colonization Facilitating Protein	Sessile Root Colonization	abrB->PGPT0014790	6
Indirect Effects	ColonizingPlant System	Root Colonization	Root Colonization Facilitating Protein	Sessile Root Colonization	codY->PGPT0016270	1
Indirect Effects	CompetitiveExclusion|CE	CE-Bacterial Fitness	CE-Bacterial Fitness-adaptive Mutation	CE-Bacterial Fitness-Environment-Inducible DNA Polymerases	dinB->PGPT0014881	1
Indirect Effects	CompetitiveExclusion|CE	CE-Bacterial Fitness	CE-Bacterial Fitness-adaptive Mutation	CE-Bacterial Fitness-Environment-Inducible DNA Polymerases	umuC->PGPT0014882	5
Indirect Effects	CompetitiveExclusi	CE-Bacterial Fitness	CE-Bacterial Fitness-CRISPR-CAS System	CE-Bacterial Fitness-Type I CRISPR-CAS	cas6->PGPT0026955	1

	on|CE			System		
Indirect Effects	CompetitiveExclusion|CE	CE-Bacterial Fitness	CE-Bacterial Fitness-CRISPR-CAS System	CE-Bacterial Fitness-Type I CRISPR-CAS System	cst2|cas7->PGPT0026935	1
Indirect Effects	CompetitiveExclusion|CE	CE-Bacterial Fitness	CE-Bacterial Fitness-CRISPR-CAS System	CE-Bacterial Fitness-Universal CAS Proteins	cas2->PGPT0026890	1
Indirect Effects	CompetitiveExclusion|CE	CE-Bacterial Fitness	CE-Bacterial Fitness-Energy Metabolism	CE-Bacterial Fitness-Aerobic Respiration|Oxidative Phosphorylation	cydA->PGPT0026700	2
Indirect Effects	CompetitiveExclusion|CE	CE-Bacterial Fitness	CE-Bacterial Fitness-Energy Metabolism	CE-Bacterial Fitness-Aerobic Respiration|Oxidative Phosphorylation	cydB->PGPT0026705	2
Indirect Effects	CompetitiveExclusion|CE	CE-Bacterial Fitness	CE-Bacterial Fitness-Energy Metabolism	CE-Bacterial Fitness-Aerobic Respiration|Oxidative Phosphorylation	ndh->PGPT0004020	3
Indirect Effects	CompetitiveExclusion|CE	CE-Bacterial Fitness	CE-Bacterial Fitness-Energy Metabolism	CE-Bacterial Fitness-Aerobic Respiration|Oxidative Phosphorylation	ndhB->PGPT0026715	1
Indirect Effects	CompetitiveExclusion|CE	CE-Bacterial Fitness	CE-Bacterial Fitness-Energy Metabolism	CE-Bacterial Fitness-Oxygen Availability Signaling	rpoS->PGPT0014685	1
Indirect Effects	CompetitiveExclusion|CE	CE-Bacterial Fitness	CE-Bacterial Fitness-Environmental Survival	CE-Bacterial Fitness-Environmental Survival-Glycogen Biosynthesis	glgA->PGPT0025880	1
Indirect Effects	CompetitiveExclusi	CE-Bacterial Fitness	CE-Bacterial Fitness-Environmental	CE-Bacterial Fitness-Environmental	glgC->PGPT0025885	2

	on|CE		Survival	Survival-Glycogen Biosynthesis		
Indirect Effects	CompetitiveExclusion|CE	CE-Bacterial Fitness	CE-Bacterial Fitness-Environmental Survival	CE-Bacterial Fitness-Environmental Survival-Glycogen Biosynthesis	glgP->PGPT0018545	1
Indirect Effects	CompetitiveExclusion|CE	CE-Bacterial Fitness	CE-Bacterial Fitness-Environmental Survival	CE-Bacterial Fitness-Environmental Survival-Glycogen Biosynthesis	yqgM->PGPT0018547	2
Indirect Effects	CompetitiveExclusion|CE	CE-Bacterial Fitness	CE-Bacterial Fitness-Mobile Elements	CE-Bacterial Fitness-Integrases|Recombinases	int|intR|intV|intQ|intW|xerC->PGPT0021996	2
Indirect Effects	CompetitiveExclusion|CE	CE-Bacterial Fitness	CE-Bacterial Fitness-Mobile Elements	CE-Bacterial Fitness-Integrases|Recombinases	xerC->PGPT0021995	1
Indirect Effects	CompetitiveExclusion|CE	CE-Bacterial Fitness	CE-Bacterial Fitness-Mobile Elements	CE-Bacterial Fitness-Integrases|Recombinases	xerD->PGPT0022000	8
Indirect Effects	CompetitiveExclusion|CE	CE-Bacterial Fitness	CE-Bacterial Fitness-Mobile Elements	CE-Bacterial Fitness-transposases	Putative transposase->PGPT0030665	2
Indirect Effects	CompetitiveExclusion|CE	CE-Bacterial Fitness	CE-Bacterial Fitness-Mobile Elements	CE-Bacterial Fitness-transposases	tnp->PGPT0030635	1
Indirect Effects	CompetitiveExclusion|CE	CE-Bacterial Fitness	CE-Bacterial Fitness-Multidrug Resistance	CE-Bacterial Fitness-Multidrug-Cell Wall-Active Antibiotic Resistance	liaF->PGPT0028815	1
Indirect Effects	CompetitiveExclusion|CE	CE-Bacterial Fitness	CE-Bacterial Fitness-Multidrug Resistance	CE-Bacterial Fitness-Multidrug-Cell Wall-Active Antibiotic	liaG->PGPT0028810	2

				Resistance		
Indirect Effects	CompetitiveExclusion|CE	CE-Bacterial Fitness	CE-Bacterial Fitness-Multidrug Resistance	CE-Bacterial Fitness-Multidrug-Cell Wall-Active Antibiotic Resistance	liaR->PGPT0014870	4
Indirect Effects	CompetitiveExclusion|CE	CE-Bacterial Fitness	CE-Bacterial Fitness-Multidrug Resistance	CE-Bacterial Fitness-Multidrug-Cell Wall-Active Antibiotic Resistance	liaS->PGPT0014865	1
Indirect Effects	CompetitiveExclusion|CE	CE-Bacterial Fitness	CE-Bacterial Fitness-Multidrug Resistance	CE-Bacterial Fitness-Multidrug transport-Efflux Pump AbcA	abcA|bmrA->PGPT0028965	4
Indirect Effects	CompetitiveExclusion|CE	CE-Bacterial Fitness	CE-Bacterial Fitness-Multidrug Resistance	CE-Bacterial Fitness-Multidrug transport-Efflux Pump AbcA	norG->PGPT0028960	7
Indirect Effects	CompetitiveExclusion|CE	CE-Bacterial Fitness	CE-Bacterial Fitness-Multidrug Resistance	CE-Bacterial Fitness-Multidrug transport-Efflux Pump AcrEF-TolC	acrR1->PGPT0014365	1
Indirect Effects	CompetitiveExclusion|CE	CE-Bacterial Fitness	CE-Bacterial Fitness-Multidrug Resistance	CE-Bacterial Fitness-Multidrug transport-Efflux Pump MepA	mepA->PGPT0028985	1
Indirect Effects	CompetitiveExclusion|CE	CE-Bacterial Fitness	CE-Bacterial Fitness-Multidrug Resistance	CE-Bacterial Fitness-Multidrug transport-Efflux Pump MexAB-OprM	acrR|smeT->PGPT0003255	2
Indirect Effects	CompetitiveExclusion|CE	CE-Bacterial Fitness	CE-Bacterial Fitness-Multidrug Resistance	CE-Bacterial Fitness-Multidrug transport-Efflux Pump MexJK-OprM	mexL->PGPT0028895	1
Indirect Effects	CompetitiveExclusi	CE-Bacterial Fitness	CE-Bacterial Fitness-Multidrug Resistance	CE-Bacterial Fitness-Multidrug transport-	cueR->PGPT0004135	9

	on|CE			Efflux Pump MexPQ-OpmE		
Indirect Effects	CompetitiveExclusion|CE	CE-Bacterial Fitness	CE-Bacterial Fitness-Multidrug Resistance	CE-Bacterial Fitness-Multidrug transport-Efflux Pump NorB	norG->PGPT0028960	7
Indirect Effects	CompetitiveExclusion|CE	CE-Bacterial Fitness	CE-Bacterial Fitness-Multidrug Resistance	CE-Bacterial Fitness-Multidrug transport-Efflux Pump QacA	qacR->PGPT0028975	2
Indirect Effects	CompetitiveExclusion|CE	CE-Bacterial Fitness	CE-Bacterial Fitness-Multidrug Resistance	CE-Bacterial Fitness-Multidrug transport-Efflux Pump QacA	smvA|qacA|lfrA->PGPT0028045	1
Indirect Effects	CompetitiveExclusion|CE	CE-Bacterial Fitness	CE-Bacterial Fitness-Multidrug Resistance	CE-Bacterial Fitness-Multidrug Related Regulation	bmrR->PGPT0003907	2
Indirect Effects	CompetitiveExclusion|CE	CE-Bacterial Fitness	CE-Bacterial Fitness-Multidrug Resistance	CE-Bacterial Fitness-Multidrug Related Regulation	rarD->PGPT0003906	3
Indirect Effects	CompetitiveExclusion|CE	CE-Bacterial Fitness	CE-Bacterial Fitness-Multidrug Resistance	CE-Bacterial Fitness-Other Multidrug Efflux Genes	TC MATE|norM|mdtK|dinF->PGPT0029115	3
Indirect Effects	CompetitiveExclusion|CE	CE-Bacterial Fitness	CE-Bacterial Fitness-Multidrug Resistance	CE-Bacterial Fitness-Other Multidrug Efflux Genes	TC SMR3->PGPT0029120	1
Indirect Effects	CompetitiveExclusion|CE	CE-Bacterial Fitness	CE-Bacterial Fitness-Multidrug Resistance	CE-Bacterial Fitness-Other Multidrug Efflux Genes	bcr|tcaB->PGPT0029005	2
Indirect Effects	CompetitiveExclusion|CE	CE-Bacterial Fitness	CE-Bacterial Fitness-Multidrug Resistance	CE-Bacterial Fitness-Other Multidrug Efflux Genes	blt->PGPT0003185	5
Indirect Effects	CompetitiveExclusion|CE	CE-Bacterial Fitness	CE-Bacterial Fitness-Multidrug Resistance	CE-Bacterial Fitness-Other Multidrug Efflux Genes	efrA->PGPT0029025	1

Indirect Effects	CompetitiveExclusion|CE	CE-Bacterial Fitness	CE-Bacterial Fitness-Multidrug Resistance	CE-Bacterial Fitness-Other Multidrug Efflux Genes	efrB->PGPT0029030	2
Indirect Effects	CompetitiveExclusion|CE	CE-Bacterial Fitness	CE-Bacterial Fitness-Multidrug Resistance	CE-Bacterial Fitness-Other Multidrug Efflux Genes	emrB->PGPT0029220	7
Indirect Effects	CompetitiveExclusion|CE	CE-Bacterial Fitness	CE-Bacterial Fitness-Multidrug Resistance	CE-Bacterial Fitness-Other Multidrug Efflux Genes	emrE|qac|mmr|smr->PGPT0029230	4
Indirect Effects	CompetitiveExclusion|CE	CE-Bacterial Fitness	CE-Bacterial Fitness-Multidrug Resistance	CE-Bacterial Fitness-Other Multidrug Efflux Genes	lmrB->PGPT0028570	5
Indirect Effects	CompetitiveExclusion|CE	CE-Bacterial Fitness	CE-Bacterial Fitness-Multidrug Resistance	CE-Bacterial Fitness-Other Multidrug Efflux Genes	mdeA->PGPT0029035	2
Indirect Effects	CompetitiveExclusion|CE	CE-Bacterial Fitness	CE-Bacterial Fitness-Multidrug Resistance	CE-Bacterial Fitness-Other Multidrug Efflux Genes	mdlA|smdA->PGPT0029040	3
Indirect Effects	CompetitiveExclusion|CE	CE-Bacterial Fitness	CE-Bacterial Fitness-Multidrug Resistance	CE-Bacterial Fitness-Other Multidrug Efflux Genes	mdlB|smdB->PGPT0029045	3
Indirect Effects	CompetitiveExclusion|CE	CE-Bacterial Fitness	CE-Bacterial Fitness-Multidrug Resistance	CE-Bacterial Fitness-Other Multidrug Efflux Genes	mdtG->PGPT0029185	3
Indirect Effects	CompetitiveExclusion|CE	CE-Bacterial Fitness	CE-Bacterial Fitness-Multidrug Resistance	CE-Bacterial Fitness-Other Multidrug Efflux Genes	mdtH->PGPT0029190	1
Indirect Effects	CompetitiveExclusion|CE	CE-Bacterial Fitness	CE-Bacterial Fitness-Multidrug Resistance	CE-Bacterial Fitness-Other Multidrug Efflux Genes	mta|ywnD->PGPT0003175	1
Indirect Effects	CompetitiveExclusi	CE-Bacterial Fitness	CE-Bacterial Fitness-Multidrug Resistance	CE-Bacterial Fitness-Other Multidrug	sdrM->PGPT0029095	1

	on|CE			Efflux Genes		
Indirect Effects	CompetitiveExclusion|CE	CE-Bacterial Fitness	CE-Bacterial Fitness-Multidrug Resistance	CE-Bacterial Fitness-Other Multidrug Efflux Genes	ToxG|mexH->PGPT0009800	1
Indirect Effects	CompetitiveExclusion|CE	CE-Bacterial Fitness	CE-Bacterial Fitness-Multidrug Resistance	CE-Bacterial Fitness-Other Multidrug Efflux Genes	ydhP->PGPT0028991	4
Indirect Effects	CompetitiveExclusion|CE	CE-Bacterial Fitness	CE-Bacterial Fitness-Multidrug Resistance	CE-Bacterial Fitness-Other Multidrug Efflux Genes	ykkC->PGPT0029165	1
Indirect Effects	CompetitiveExclusion|CE	CE-Bacterial Fitness	CE-Bacterial Fitness-Multidrug Resistance	CE-Bacterial Fitness-Other Multidrug Efflux Genes	ymfE->PGPT0003580	3
Indirect Effects	CompetitiveExclusion|CE	CE-Bacterial Fitness	CE-Bacterial Fitness-Multidrug Resistance	CE-Bacterial Fitness-Resistance To Toxic aminoacyl Nucleotides-1	aaaT->PGPT0020290	7
Indirect Effects	CompetitiveExclusion|CE	CE-Bacterial Fitness	CE-Bacterial Fitness-Phage Defense System	CE-Bacterial Fitness-Phage Exclusion	mcp|tlpC|tlpA|dcrA->PGPT0015710	2
Indirect Effects	CompetitiveExclusion|CE	CE-Bacterial Fitness	CE-Bacterial Fitness-Phage Defense System	CE-Bacterial Fitness-Phage Shock Proteins	pspE->PGPT0014648	2
Indirect Effects	CompetitiveExclusion|CE	CE-Bacterial Fitness	CE-Bacterial Fitness-Phage Defense System	CE-Bacterial Fitness-Phage Shock Proteins	Toxin pspC->PGPT0027520	1
Indirect Effects	CompetitiveExclusion|CE	CE-Bacterial Fitness	CE-Bacterial Fitness-Resistance To Antimicrobial|Toxic Compounds	CE-Bacterial Fitness-3-Nitropropionate|3-NPA Resistance	ncd2|npd|pnoA->PGPT0006800	4
Indirect Effects	CompetitiveExclusi	CE-Bacterial Fitness	CE-Bacterial Fitness-Resistance To	CE-Bacterial Fitness-3-PhenylPropionic	mhpC->PGPT0019765	2

	on|CE		Antimicrobial|Toxic Compounds	Acid|Cinnamic Acid Resistance		
Indirect Effects	CompetitiveExclusion|CE	CE-Bacterial Fitness	CE-Bacterial Fitness-Resistance To Antimicrobial|Toxic Compounds	CE-Bacterial Fitness-3-PhenylPropionic Acid|Cinnamic Acid Resistance	mhpE->PGPT0002050	1
Indirect Effects	CompetitiveExclusion|CE	CE-Bacterial Fitness	CE-Bacterial Fitness-Resistance To Antimicrobial|Toxic Compounds	CE-Bacterial Fitness-5-NitroIMidazole Antibiotic Resistance-Azomyzin	nimD->PGPT0028780	1
Indirect Effects	CompetitiveExclusion|CE	CE-Bacterial Fitness	CE-Bacterial Fitness-Resistance To Antimicrobial|Toxic Compounds	CE-Bacterial Fitness-Aminoglycosides Resistance-Streptomycin|Kanamycin|Amikacin	aac6 I->PGPT0028630	3
Indirect Effects	CompetitiveExclusion|CE	CE-Bacterial Fitness	CE-Bacterial Fitness-Resistance To Antimicrobial|Toxic Compounds	CE-Bacterial Fitness-Aminoglycosides Resistance-Streptomycin|Kanamycin|Amikacin	aac6 I|aacA7->PGPT0028640	1
Indirect Effects	CompetitiveExclusion|CE	CE-Bacterial Fitness	CE-Bacterial Fitness-Resistance To Antimicrobial|Toxic Compounds	CE-Bacterial Fitness-Aminoglycosides Resistance-Streptomycin|Kanamycin|Amikacin	aacA->PGPT0028650	1
Indirect Effects	CompetitiveExclusion|CE	CE-Bacterial Fitness	CE-Bacterial Fitness-Resistance To Antimicrobial|Toxic Compounds	CE-Bacterial Fitness-Aminoglycosides Resistance-Streptomycin|Kanamycin|Amikacin	aacC->PGPT0028625	1
Indirect Effects	CompetitiveExclusi	CE-Bacterial Fitness	CE-Bacterial Fitness-Resistance To	CE-Bacterial Fitness-Aminoglycosides	sat4->PGPT0028670	1

	on|CE		Antimicrobial|Toxic Compounds	Resistance-Streptomycin|Kanamycin|Amikacin		
Indirect Effects	CompetitiveExclusion|CE	CE-Bacterial Fitness	CE-Bacterial Fitness-Resistance To Antimicrobial|Toxic Compounds	CE-Bacterial Fitness-Aminoglycosides Resistance-Streptomycin|Kanamycin|Amikacin	strB->PGPT0028755	1
Indirect Effects	CompetitiveExclusion|CE	CE-Bacterial Fitness	CE-Bacterial Fitness-Resistance To Antimicrobial|Toxic Compounds	CE-Bacterial Fitness-Antimicrobial Compound Resistance-Acetoin|2|3-Butanediol Synthesis	aceF|pdhC->PGPT0001390	2
Indirect Effects	CompetitiveExclusion|CE	CE-Bacterial Fitness	CE-Bacterial Fitness-Resistance To Antimicrobial|Toxic Compounds	CE-Bacterial Fitness-Antimicrobial Compound Resistance-Acetoin|2|3-Butanediol Synthesis	acoA->PGPT0008230	1
Indirect Effects	CompetitiveExclusion|CE	CE-Bacterial Fitness	CE-Bacterial Fitness-Resistance To Antimicrobial|Toxic Compounds	CE-Bacterial Fitness-Antimicrobial Compound Resistance-Acetoin|2|3-Butanediol Synthesis	acoB->PGPT0008235	1
Indirect Effects	CompetitiveExclusion|CE	CE-Bacterial Fitness	CE-Bacterial Fitness-Resistance To Antimicrobial|Toxic Compounds	CE-Bacterial Fitness-Antimicrobial Compound Resistance-Acetoin|2|3-Butanediol Synthesis	acoR->PGPT0001030	6

Indirect Effects	CompetitiveExclusion|CE	CE-Bacterial Fitness	CE-Bacterial Fitness-Resistance To Antimicrobial|Toxic Compounds	CE-Bacterial Fitness-Antimicrobial Compound Resistance-Acetoin|2|3-Butanediol Synthesis	acuB->PGPT0008220	4
Indirect Effects	CompetitiveExclusion|CE	CE-Bacterial Fitness	CE-Bacterial Fitness-Resistance To Antimicrobial|Toxic Compounds	CE-Bacterial Fitness-Antimicrobial Compound Resistance-Acetoin|2|3-Butanediol Synthesis	acuC->PGPT0008225	1
Indirect Effects	CompetitiveExclusion|CE	CE-Bacterial Fitness	CE-Bacterial Fitness-Resistance To Antimicrobial|Toxic Compounds	CE-Bacterial Fitness-Antimicrobial Compound Resistance-Acetoin|2|3-Butanediol Synthesis	budA|aldC|aldB|alsD->PGPT0008180	1
Indirect Effects	CompetitiveExclusion|CE	CE-Bacterial Fitness	CE-Bacterial Fitness-Resistance To Antimicrobial|Toxic Compounds	CE-Bacterial Fitness-Antimicrobial Compound Resistance-Acetoin|2|3-Butanediol Synthesis	budB|ilvK|alsS|ilvB|ilvG|ilvI->PGPT0008185	4
Indirect Effects	CompetitiveExclusion|CE	CE-Bacterial Fitness	CE-Bacterial Fitness-Resistance To Antimicrobial|Toxic Compounds	CE-Bacterial Fitness-Antimicrobial Compound Resistance-Acetoin|2|3-Butanediol Synthesis	budC->PGPT0008190	3
Indirect Effects	CompetitiveExclusi	CE-Bacterial Fitness	CE-Bacterial Fitness-Resistance To	CE-Bacterial Fitness-Antimicrobial	butA|ydjL|budC->PGPT0008195	1

	on|CE		Antimicrobial|Toxic Compounds	Compound Resistance-Acetoin|2|3-Butanediol Synthesis		
Indirect Effects	CompetitiveExclusion|CE	CE-Bacterial Fitness	CE-Bacterial Fitness-Resistance To Antimicrobial|Toxic Compounds	CE-Bacterial Fitness-Antimicrobial Compound Resistance-Acetoin|2|3-Butanediol Synthesis	butB->PGPT0008200	1
Indirect Effects	CompetitiveExclusion|CE	CE-Bacterial Fitness	CE-Bacterial Fitness-Resistance To Antimicrobial|Toxic Compounds	CE-Bacterial Fitness-Antimicrobial Compound Resistance-Acetoin|2|3-Butanediol Synthesis	ilvH|ilvN->PGPT0008205	1
Indirect Effects	CompetitiveExclusion|CE	CE-Bacterial Fitness	CE-Bacterial Fitness-Resistance To Antimicrobial|Toxic Compounds	CE-Bacterial Fitness-Antimicrobial Compound Resistance-Acetoin|2|3-Butanediol Synthesis	lpd|pdhD->PGPT0001380	3
Indirect Effects	CompetitiveExclusion|CE	CE-Bacterial Fitness	CE-Bacterial Fitness-Resistance To Antimicrobial|Toxic Compounds	CE-Bacterial Fitness-Bacimethrin|CF3-HMP Detoxification	nudJ|ymfB->PGPT0028060	1
Indirect Effects	CompetitiveExclusion|CE	CE-Bacterial Fitness	CE-Bacterial Fitness-Resistance To Antimicrobial|Toxic Compounds	CE-Bacterial Fitness-Bacimethrin|CF3-HMP Detoxification	thiC->PGPT0008905	1
Indirect Effects	CompetitiveExclusi	CE-Bacterial Fitness	CE-Bacterial Fitness-Resistance To	CE-Bacterial Fitness-Bacimethrin|CF3-	yjjX->PGPT0021540	1

	on|CE		Antimicrobial|Toxic Compounds	HMP Detoxification		
Indirect Effects	CompetitiveExclusion|CE	CE-Bacterial Fitness	CE-Bacterial Fitness-Resistance To Antimicrobial|Toxic Compounds	CE-Bacterial Fitness-Bacitracin Resistance	bceA|vraD->PGPT0010200	2
Indirect Effects	CompetitiveExclusion|CE	CE-Bacterial Fitness	CE-Bacterial Fitness-Resistance To Antimicrobial|Toxic Compounds	CE-Bacterial Fitness-Bacitracin Resistance	bceR->PGPT0010230	1
Indirect Effects	CompetitiveExclusion|CE	CE-Bacterial Fitness	CE-Bacterial Fitness-Resistance To Antimicrobial|Toxic Compounds	CE-Bacterial Fitness-Bacitracin Resistance	bcsS->PGPT0010225	1
Indirect Effects	CompetitiveExclusion|CE	CE-Bacterial Fitness	CE-Bacterial Fitness-Resistance To Antimicrobial|Toxic Compounds	CE-Bacterial Fitness-Beta-Lactam Resistance	ampD->PGPT0024100	1
Indirect Effects	CompetitiveExclusion|CE	CE-Bacterial Fitness	CE-Bacterial Fitness-Resistance To Antimicrobial|Toxic Compounds	CE-Bacterial Fitness-Beta-Lactam Resistance	blaI->PGPT0028105	2
Indirect Effects	CompetitiveExclusion|CE	CE-Bacterial Fitness	CE-Bacterial Fitness-Resistance To Antimicrobial|Toxic Compounds	CE-Bacterial Fitness-Beta-Lactam Resistance	cusR|copR|silR->PGPT0004105	1
Indirect Effects	CompetitiveExclusion|CE	CE-Bacterial Fitness	CE-Bacterial Fitness-Resistance To Antimicrobial|Toxic Compounds	CE-Bacterial Fitness-Beta-Lactam Resistance	cusS|copS|silS->PGPT0004980	1
Indirect Effects	CompetitiveExclusi	CE-Bacterial Fitness	CE-Bacterial Fitness-Resistance To	CE-Bacterial Fitness-Beta-Lactam	pbp2A->PGPT0023895	2

	on|CE		Antimicrobial|Toxic Compounds	Resistance		
Indirect Effects	CompetitiveExclusion|CE	CE-Bacterial Fitness	CE-Bacterial Fitness-Resistance To Antimicrobial|Toxic Compounds	CE-Bacterial Fitness-Beta-Lactam Resistance	penP->PGPT0028070	2
Indirect Effects	CompetitiveExclusion|CE	CE-Bacterial Fitness	CE-Bacterial Fitness-Resistance To Antimicrobial|Toxic Compounds	CE-Bacterial Fitness-Bleomycin Resistance	gloA|ywbC->PGPT0013300	11
Indirect Effects	CompetitiveExclusion|CE	CE-Bacterial Fitness	CE-Bacterial Fitness-Resistance To Antimicrobial|Toxic Compounds	CE-Bacterial Fitness-Catechol Resistance	catD->PGPT0028505	3
Indirect Effects	CompetitiveExclusion|CE	CE-Bacterial Fitness	CE-Bacterial Fitness-Resistance To Antimicrobial|Toxic Compounds	CE-Bacterial Fitness-Catechol Resistance	catE->PGPT0005050	1
Indirect Effects	CompetitiveExclusion|CE	CE-Bacterial Fitness	CE-Bacterial Fitness-Resistance To Antimicrobial|Toxic Compounds	CE-Bacterial Fitness-Catechol Resistance	mhqA|mhqE|mhqO|yaiA->PGPT0028510	3
Indirect Effects	CompetitiveExclusion|CE	CE-Bacterial Fitness	CE-Bacterial Fitness-Resistance To Antimicrobial|Toxic Compounds	CE-Bacterial Fitness-Catechol|Chromanon|Ganomycin Resistance	yodB->PGPT0028512	5
Indirect Effects	CompetitiveExclusion|CE	CE-Bacterial Fitness	CE-Bacterial Fitness-Resistance To Antimicrobial|Toxic Compounds	CE-Bacterial Fitness-Catechol|Chromanon|Ganomycin Resistance	yodC->PGPT0028511	3
Indirect Effects	CompetitiveExclusi	CE-Bacterial Fitness	CE-Bacterial Fitness-Resistance To	CE-Bacterial Fitness-Chromanon Resistance	nfrA2|ycnD->PGPT0000295	1

	on|CE		Antimicrobial|Toxic Compounds			
Indirect Effects	CompetitiveExclusion|CE	CE-Bacterial Fitness	CE-Bacterial Fitness-Resistance To Antimicrobial|Toxic Compounds	CE-Bacterial Fitness-Phosphomycin Resistance	fosB->PGPT0028560	1
Indirect Effects	CompetitiveExclusion|CE	CE-Bacterial Fitness	CE-Bacterial Fitness-Resistance To Antimicrobial|Toxic Compounds	CE-Bacterial Fitness-Phosphomycin Resistance	gloA|ywbC->PGPT0013300	11
Indirect Effects	CompetitiveExclusion|CE	CE-Bacterial Fitness	CE-Bacterial Fitness-Resistance To Antimicrobial|Toxic Compounds	CE-Bacterial Fitness-Gallate Resistance	ligK|galC->PGPT0002085	5
Indirect Effects	CompetitiveExclusion|CE	CE-Bacterial Fitness	CE-Bacterial Fitness-Resistance To Antimicrobial|Toxic Compounds	CE-Bacterial Fitness-Glycopeptide Resistance	vanY|yodJ->PGPT0024055	3
Indirect Effects	CompetitiveExclusion|CE	CE-Bacterial Fitness	CE-Bacterial Fitness-Resistance To Antimicrobial|Toxic Compounds	CE-Bacterial Fitness-Hydroquinone-Derivate Resistance	catD->PGPT0028505	3
Indirect Effects	CompetitiveExclusion|CE	CE-Bacterial Fitness	CE-Bacterial Fitness-Resistance To Antimicrobial|Toxic Compounds	CE-Bacterial Fitness-Hydroquinone-Derivate Resistance	mhqA|mhqE|mhqO|yaiA->PGPT0028510	3
Indirect Effects	CompetitiveExclusion|CE	CE-Bacterial Fitness	CE-Bacterial Fitness-Resistance To Antimicrobial|Toxic Compounds	CE-Bacterial Fitness-HydroxyCinnamic Acid Resistance	paaF|echA->PGPT0001860	1
Indirect Effects	CompetitiveExclusi	CE-Bacterial Fitness	CE-Bacterial Fitness-Resistance To	CE-Bacterial Fitness-HydroxyCinnamic	pcaC->PGPT0005005	6

	on|CE		Antimicrobial|Toxic Compounds	Acid Resistance		
Indirect Effects	CompetitiveExclusion|CE	CE-Bacterial Fitness	CE-Bacterial Fitness-Resistance To Antimicrobial|Toxic Compounds	CE-Bacterial Fitness-HydroxyCinnamic Acid Resistance	pcaD|catD->PGPT0004995	3
Indirect Effects	CompetitiveExclusion|CE	CE-Bacterial Fitness	CE-Bacterial Fitness-Resistance To Antimicrobial|Toxic Compounds	CE-Bacterial Fitness-Lantibiotic Resistance	nukE|mcdE|sboE->PGPT0011720	2
Indirect Effects	CompetitiveExclusion|CE	CE-Bacterial Fitness	CE-Bacterial Fitness-Resistance To Antimicrobial|Toxic Compounds	CE-Bacterial Fitness-Lantibiotic Resistance	nukF|mcdF|sbOf->PGPT0011725	2
Indirect Effects	CompetitiveExclusion|CE	CE-Bacterial Fitness	CE-Bacterial Fitness-Resistance To Antimicrobial|Toxic Compounds	CE-Bacterial Fitness-Lantibiotic Resistance	nukG|mcdG|sboG->PGPT0011715	2
Indirect Effects	CompetitiveExclusion|CE	CE-Bacterial Fitness	CE-Bacterial Fitness-Resistance To Antimicrobial|Toxic Compounds	CE-Bacterial Fitness-LINCOmycin Resistance	lmrB->PGPT0028570	5
Indirect Effects	CompetitiveExclusion|CE	CE-Bacterial Fitness	CE-Bacterial Fitness-Resistance To Antimicrobial|Toxic Compounds	CE-Bacterial Fitness-Macrolide Resistance	cfr->PGPT0027930	1
Indirect Effects	CompetitiveExclusion|CE	CE-Bacterial Fitness	CE-Bacterial Fitness-Resistance To Antimicrobial|Toxic Compounds	CE-Bacterial Fitness-Macrolide Resistance	ereA B->PGPT0027980	3
Indirect Effects	CompetitiveExclusi	CE-Bacterial Fitness	CE-Bacterial Fitness-Resistance To	CE-Bacterial Fitness-Macrolide Resistance	mef|ykuC->PGPT0027965	8

	on|CE		Antimicrobial|Toxic Compounds			
Indirect Effects	CompetitiveExclusion|CE	CE-Bacterial Fitness	CE-Bacterial Fitness-Resistance To Antimicrobial|Toxic Compounds	CE-Bacterial Fitness-Macrolide Resistance	mgt|oleD->PGPT0028005	2
Indirect Effects	CompetitiveExclusion|CE	CE-Bacterial Fitness	CE-Bacterial Fitness-Resistance To Antimicrobial|Toxic Compounds	CE-Bacterial Fitness-Macrolide Resistance	msrA|vmlR->PGPT0027940	4
Indirect Effects	CompetitiveExclusion|CE	CE-Bacterial Fitness	CE-Bacterial Fitness-Resistance To Antimicrobial|Toxic Compounds	CE-Bacterial Fitness-Macrolide Resistance	tylC|oleB|carA|srmB->PGPT0027935	1
Indirect Effects	CompetitiveExclusion|CE	CE-Bacterial Fitness	CE-Bacterial Fitness-Resistance To Antimicrobial|Toxic Compounds	CE-Bacterial Fitness-Methylglyoxal Detoxification	ghrA->PGPT0019465	1
Indirect Effects	CompetitiveExclusion|CE	CE-Bacterial Fitness	CE-Bacterial Fitness-Resistance To Antimicrobial|Toxic Compounds	CE-Bacterial Fitness-Methylglyoxal Detoxification	gloA|ywbC->PGPT0013300	11
Indirect Effects	CompetitiveExclusion|CE	CE-Bacterial Fitness	CE-Bacterial Fitness-Resistance To Antimicrobial|Toxic Compounds	CE-Bacterial Fitness-Methylglyoxal Detoxification	yfkM|pfpI|yraA->PGPT0015010	3
Indirect Effects	CompetitiveExclusion|CE	CE-Bacterial Fitness	CE-Bacterial Fitness-Resistance To Antimicrobial|Toxic Compounds	CE-Bacterial Fitness-Methylglyoxal Detoxification	yvgN->PGPT0014985	2
Indirect Effects	CompetitiveExclusi	CE-Bacterial Fitness	CE-Bacterial Fitness-Resistance To	CE-Bacterial Fitness-Phenicol Resistance	cfr->PGPT0027930	1

	on|CE		Antimicrobial|Toxic Compounds			
Indirect Effects	CompetitiveExclusion|CE	CE-Bacterial Fitness	CE-Bacterial Fitness-Resistance To Antimicrobial|Toxic Compounds	CE-Bacterial Fitness-Polymyxin Resistance	pmrA->PGPT0003915	3
Indirect Effects	CompetitiveExclusion|CE	CE-Bacterial Fitness	CE-Bacterial Fitness-Resistance To Antimicrobial|Toxic Compounds	CE-Bacterial Fitness-propionate-3-Nitrate-Derivate Resistance	ncd2|npd|pnoA->PGPT0006800	4
Indirect Effects	CompetitiveExclusion|CE	CE-Bacterial Fitness	CE-Bacterial Fitness-Resistance To Antimicrobial|Toxic Compounds	CE-Bacterial Fitness-Quaternary Ammonium Compound Resistance	sugE->PGPT0028785	3
Indirect Effects	CompetitiveExclusion|CE	CE-Bacterial Fitness	CE-Bacterial Fitness-Resistance To Antimicrobial|Toxic Compounds	CE-Bacterial Fitness-Quinoline Resistance	acrR|smeT->PGPT0003255	2
Indirect Effects	CompetitiveExclusion|CE	CE-Bacterial Fitness	CE-Bacterial Fitness-Resistance To Antimicrobial|Toxic Compounds	CE-Bacterial Fitness-Quinolone Resistance	smvA|qacA|lfrA->PGPT0028045	1
Indirect Effects	CompetitiveExclusion|CE	CE-Bacterial Fitness	CE-Bacterial Fitness-Resistance To Antimicrobial|Toxic Compounds	CE-Bacterial Fitness-Resistance To Antimicrobial Peptides	degP|htrA->PGPT0015105	3
Indirect Effects	CompetitiveExclusion|CE	CE-Bacterial Fitness	CE-Bacterial Fitness-Resistance To Antimicrobial|Toxic Compounds	CE-Bacterial Fitness-Resistance To Antimicrobial Peptides	dltE->PGPT0013220	1
Indirect Effects	CompetitiveExclusi	CE-Bacterial Fitness	CE-Bacterial Fitness-Resistance To	CE-Bacterial Fitness-Resistance To	pmrA->PGPT0003915	3

	on|CE		Antimicrobial|Toxic Compounds	Antimicrobial Peptides		
Indirect Effects	CompetitiveExclusion|CE	CE-Bacterial Fitness	CE-Bacterial Fitness-Resistance To Antimicrobial|Toxic Compounds	CE-Bacterial Fitness-Resistance To Antimicrobial Peptides	ppiB->PGPT0015111	1
Indirect Effects	CompetitiveExclusion|CE	CE-Bacterial Fitness	CE-Bacterial Fitness-Resistance To Antimicrobial|Toxic Compounds	CE-Bacterial Fitness-Resistance To Antimicrobial Peptides	yxdJ->PGPT0027850	1
Indirect Effects	CompetitiveExclusion|CE	CE-Bacterial Fitness	CE-Bacterial Fitness-Resistance To Antimicrobial|Toxic Compounds	CE-Bacterial Fitness-Resistance To Antimicrobial Peptides	yxdK->PGPT0027855	1
Indirect Effects	CompetitiveExclusion|CE	CE-Bacterial Fitness	CE-Bacterial Fitness-Resistance To Antimicrobial|Toxic Compounds	CE-Bacterial Fitness-Salicylic Acid Resistance	bsdA->PGPT0019770	4
Indirect Effects	CompetitiveExclusion|CE	CE-Bacterial Fitness	CE-Bacterial Fitness-Resistance To Antimicrobial|Toxic Compounds	CE-Bacterial Fitness-Salicylic Acid Resistance	bsdC->PGPT0005435	1
Indirect Effects	CompetitiveExclusion|CE	CE-Bacterial Fitness	CE-Bacterial Fitness-Resistance To Antimicrobial|Toxic Compounds	CE-Bacterial Fitness-Salicylic Acid Resistance	ubiX|bsdB->PGPT0009565	1
Indirect Effects	CompetitiveExclusion|CE	CE-Bacterial Fitness	CE-Bacterial Fitness-Resistance To Antimicrobial|Toxic Compounds	CE-Bacterial Fitness-Surfactin Resistance	swrC|yerP->PGPT0016195	3
Indirect Effects	CompetitiveExclusi	CE-Bacterial Fitness	CE-Bacterial Fitness-Resistance To	CE-Bacterial Fitness-Tetracycline	otrA|tetM|teTo->PGPT0010355	3

	on|CE		Antimicrobial|Toxic Compounds	Resistance		
Indirect Effects	CompetitiveExclusion|CE	CE-Bacterial Fitness	CE-Bacterial Fitness-Resistance To Antimicrobial|Toxic Compounds	CE-Bacterial Fitness-Tetracycline Resistance	tet35->PGPT0027895	1
Indirect Effects	CompetitiveExclusion|CE	CE-Bacterial Fitness	CE-Bacterial Fitness-Resistance To Antimicrobial|Toxic Compounds	CE-Bacterial Fitness-Tetracycline Resistance	tetR->PGPT0027886	1
Indirect Effects	CompetitiveExclusion|CE	CE-Bacterial Fitness	CE-Bacterial Fitness-Resistance To Antimicrobial|Toxic Compounds	CE-Bacterial Fitness-Toxines Neutralization SpdC Immunity	spdI->PGPT0028597	1
Indirect Effects	CompetitiveExclusion|CE	CE-Bacterial Fitness	CE-Bacterial Fitness-Resistance To Antimicrobial|Toxic Compounds	CE-Bacterial Fitness-Toxines Neutralization YDF System	ydfH->PGPT0028585	2
Indirect Effects	CompetitiveExclusion|CE	CE-Bacterial Fitness	CE-Bacterial Fitness-Resistance To Antimicrobial|Toxic Compounds	CE-Bacterial Fitness-Toxines Neutralization YDF System	ydfI->PGPT0028590	1
Indirect Effects	CompetitiveExclusion|CE	CE-Bacterial Fitness	CE-Bacterial Fitness-Resistance To Antimicrobial|Toxic Compounds	CE-Bacterial Fitness-Toxines Neutralization YDF System	ydfJ->PGPT0028595	2
Indirect Effects	CompetitiveExclusion|CE	CE-Bacterial Fitness	CE-Bacterial Fitness-Resistance To Antimicrobial|Toxic Compounds	CE-Bacterial Fitness-Vancomycin Resistance	vanRC|vanRE|vanRG->PGPT0028485	1
Indirect Effects	CompetitiveExclusi	CE-Bacterial Fitness	CE-Bacterial Fitness-Resistance To	CE-Bacterial Fitness-Vancomycin	vanSC|vanSE|vanSG->PGPT0028480	1

	on|CE		Antimicrobial|Toxic Compounds	Resistance		
Indirect Effects	CompetitiveExclusion|CE	CE-Bacterial Fitness	CE-Bacterial Fitness-Resistance To Antimicrobial|Toxic Compounds	CE-Bacterial Fitness-Vancomycin Resistance	vanY|yodJ->PGPT0024055	3
Indirect Effects	CompetitiveExclusion|CE	CE-Bacterial Fitness	CE-Bacterial Fitness-Resistance To Antimicrobial|Toxic Compounds	RCE-Bacterial Fitness-Resistance To Antimicrobial Peptides|Bleomycin	mhqA|mhqE|mhqO|yaiA->PGPT0028510	3
Indirect Effects	CompetitiveExclusion|CE	CE-Bacterial Fitness	CE-Bacterial Fitness-Restriction|Modification System	CE-Bacterial Fitness-Type III R-M System	mod->PGPT0027230	1
Indirect Effects	CompetitiveExclusion|CE	CE-Bacterial Fitness	CE-Bacterial Fitness-Restriction|Modification System	CE-Bacterial Fitness-Type IV R-M System	mcrB->PGPT0027240	1
Indirect Effects	CompetitiveExclusion|CE	CE-Bacterial Fitness	CE-Bacterial Fitness-Restriction|Modification System	CE-Bacterial Fitness-Type IV R-M System	mrr->PGPT0027250	3
Indirect Effects	CompetitiveExclusion|CE	CE-Bacterial Fitness	CE-Bacterial Fitness-Rhizopine Metabolism	CE-Bacterial Fitness-Rhizopine Biosynthesis	dapA|mosA->PGPT0002080	3
Indirect Effects	CompetitiveExclusion|CE	CE-Bacterial Fitness	CE-Bacterial Fitness-Rhizopine Metabolism	CE-Bacterial Fitness-Rhizopine Catabolism	mocC->PGPT0016785	3
Indirect Effects	CompetitiveExclusion|CE	CE-Bacterial Fitness	CE-Bacterial Fitness-Toxin-Antitoxin System	CE-Bacterial Fitness-AbrB Toxin-Antitoxin System	Antitoxin abrB->PGPT0027725	1
Indirect Effects	CompetitiveExclusion|CE	CE-Bacterial Fitness	CE-Bacterial Fitness-Toxin-Antitoxin System	CE-Bacterial Fitness-HipA-HipB Toxin-Antitoxin System	Regulation hipB->PGPT0027485	2
Indirect	Competiti	CE-Bacterial Fitness	CE-Bacterial Fitness-	CE-Bacterial Fitness-	Toxin	1

Effects	veExclusion|CE		Toxin-Antitoxin System	MazF-MazE Toxin-Antitoxin System	mazF|ndoA|chpApemK->PGPT0027390	
Indirect Effects	CompetitiveExclusion|CE	CE-Bacterial Fitness	CE-Bacterial Fitness-Toxin-Antitoxin System	CE-Bacterial Fitness-PspC-PspB Toxin-Antitoxin System	Toxin pspC->PGPT0027520	1
Indirect Effects	CompetitiveExclusion|CE	CE-Bacterial Fitness	CE-Bacterial Fitness-Toxin-Antitoxin System	CE-Bacterial Fitness-RatA-RatB|YfjG-YfjF|RatAB-SsrAS System	smpB->PGPT0014355	1
Indirect Effects	CompetitiveExclusion|CE	CE-Bacterial Fitness	CE-Bacterial Fitness-Toxin-Antitoxin System	CE-Bacterial Fitness-Sulfur Modification Proteins	nifS|iscS->PGPT0000065	4
Indirect Effects	CompetitiveExclusion|CE	CE-Bacterial Fitness	CE-Bacterial Fitness-Toxin-Antitoxin System	CE-Bacterial Fitness-SymR-SymE Toxin-Antitoxin System	Toxin symE->PGPT0027290	1
Indirect Effects	CompetitiveExclusion|CE	CE-Bacterial Fitness	CE-Bacterial Fitness-Toxin-Antitoxin System	CE-Bacterial Fitness-Type II Toxin-Antitoxin System Related Factors	ftsZ->PGPT0027695	2
Indirect Effects	CompetitiveExclusion|CE	CE-Bacterial Fitness	CE-Bacterial Fitness-Toxin-Antitoxin System	CE-Bacterial Fitness-Type II Toxin-Antitoxin System Related Factors	gltX->PGPT0008460	1
Indirect Effects	CompetitiveExclusion|CE	CE-Bacterial Fitness	CE-Bacterial Fitness-Toxin-Antitoxin System	CE-Bacterial Fitness-Type II Toxin-Antitoxin System Related Factors	mreB->PGPT0027700	2
Indirect Effects	CompetitiveExclusion|CE	CE-Bacterial Fitness	CE-Bacterial Fitness-Toxin-Antitoxin System	CE-Bacterial Fitness-Type II Toxin-Antitoxin System Related Factors	parC->PGPT0027705	2
Indirect	Competiti	CE-Bacterial Fitness	CE-Bacterial Fitness-	CE-Bacterial Fitness-	parE->PGPT0027710	2

Effects	veExclusi on|CE		Toxin-Antitoxin System	Type II Toxin-Antitoxin System Related Factors		
Indirect Effects	Competiti veExclusi on|CE	CE-Bacterial Fitness	CE-Bacterial Fitness-Toxin-Antitoxin System	CE-Bacterial Fitness-YobL-YobK Toxin-Antitoxin System	Antitoxin yobK->PGPT0027635	1
Indirect Effects	Competiti veExclusi on|CE	CE-Bacterial Fitness	CE-Bacterial Fitness-Toxin-Antitoxin System	CE-Bacterial Fitness-YqcG-YqcF Toxin-Antitoxin System	Antitoxin yqcF->PGPT0027655	1
Indirect Effects	Competiti veExclusi on|CE	CE-Bacterial Fitness	CE-Bacterial Fitness-Toxin-Antitoxin System	CE-Bacterial Fitness-YxiD-YxxD Toxin-Antitoxin System	Antitoxin yxxD->PGPT0027665	2
Indirect Effects	Competiti veExclusi on|CE	CE-Bacterial Fitness	Resistance To Antimicrobial|Toxic Compounds	CE-Bacterial Fitness-Carbomycin Resistance	tylC|oleB|carA|srmB->PGPT0027935	1
Indirect Effects	Competiti veExclusi on|CE	CE-Bacterial Secretion	CE-SEC-Secretion Pathway	CE-SEC-Secretion Related Proteins	syd->PGPT0029274	3
Indirect Effects	Competiti veExclusi on|CE	CE-Bacterial Secretion	CE-SEC-Secretion Pathway	CE-SEC-SRP Core Components	ffh->PGPT0025750	1
Indirect Effects	Competiti veExclusi on|CE	CE-Bacterial Secretion	CE-SEC-Secretion Pathway	CE-SEC-SRP Core Components	ftsY->PGPT0025740	1
Indirect Effects	Competiti veExclusi on|CE	CE-Bacterial Secretion	CE-SEC-Secretion Pathway	CE-SEC-SRP Core Components	secA->PGPT0025735	1
Indirect Effects	Competiti veExclusi on|CE	CE-Bacterial Secretion	CE-SEC-Secretion Pathway	CE-SEC-SRP Core Components	secDF->PGPT0025710	1
Indirect Effects	Competiti veExclusi	CE-Bacterial Secretion	CE-SEC-Secretion Pathway	CE-SEC-SRP Core Components	secE->PGPT0025715	1

	on|CE					
Indirect Effects	CompetitiveExclusion|CE	CE-Bacterial Secretion	CE-SEC-Secretion Pathway	CE-SEC-SRP Core Components	secG->PGPT0025720	1
Indirect Effects	CompetitiveExclusion|CE	CE-Bacterial Secretion	CE-SEC-Secretion Pathway	CE-SEC-SRP Core Components	secY->PGPT0025725	1
Indirect Effects	CompetitiveExclusion|CE	CE-Bacterial Secretion	CE-SEC-Secretion Pathway	CE-SEC-SRP Core Components	yajC->PGPT0025730	1
Indirect Effects	CompetitiveExclusion|CE	CE-Bacterial Secretion	CE-SEC-Secretion Pathway	CE-SEC-SRP Core Components	yidC|spoIIIJ|oxaA|ccfA->PGPT0024530	3
Indirect Effects	CompetitiveExclusion|CE	CE-Bacterial Secretion	CE-TAT-TWIN-Arginine TransloCation Pathway	CE-TAT-TWIN-Arginine TransloCation Proteins	tatA->PGPT0029290	6
Indirect Effects	CompetitiveExclusion|CE	CE-Bacterial Secretion	CE-TAT-TWIN-Arginine TransloCation Pathway	CE-TAT-TWIN-Arginine TransloCation Proteins	tatC->PGPT0029295	3
Indirect Effects	CompetitiveExclusion|CE	CE-Bacterial Secretion	CE-Type III Secretion Systems	CE-T3SS-Flagellar Export Apparatus	flhA|lfhA|fhiA|rhcV->PGPT0015320	1
Indirect Effects	CompetitiveExclusion|CE	CE-Bacterial Secretion	CE-Type III Secretion Systems	CE-T3SS-Flagellar Export Apparatus	flhB->PGPT0015325	1
Indirect Effects	CompetitiveExclusion|CE	CE-Bacterial Secretion	CE-Type III Secretion Systems	CE-T3SS-Flagellar Export Apparatus	fliF->PGPT0015430	1
Indirect Effects	CompetitiveExclusi	CE-Bacterial Secretion	CE-Type III Secretion Systems	CE-T3SS-Flagellar Export Apparatus	fliH->PGPT0015335	1

	on|CE					
Indirect Effects	CompetitiveExclusion|CE	CE-Bacterial Secretion	CE-Type III Secretion Systems	CE-T3SS-Flagellar Export Apparatus	fliI|lgiI->PGPT0015340	1
Indirect Effects	CompetitiveExclusion|CE	CE-Bacterial Secretion	CE-Type III Secretion Systems	CE-T3SS-Flagellar Export Apparatus	fliN|lfiN|fliNY|cheC|cheD->PGPT0015410	1
Indirect Effects	CompetitiveExclusion|CE	CE-Bacterial Secretion	CE-Type III Secretion Systems	CE-T3SS-Flagellar Export Apparatus	fliOZ->PGPT0015345	1
Indirect Effects	CompetitiveExclusion|CE	CE-Bacterial Secretion	CE-Type III Secretion Systems	CE-T3SS-Flagellar Export Apparatus	fliP|rhcR->PGPT0015350	1
Indirect Effects	CompetitiveExclusion|CE	CE-Bacterial Secretion	CE-Type III Secretion Systems	CE-T3SS-Flagellar Export Apparatus	fliQ|lfiQ->PGPT0015355	1
Indirect Effects	CompetitiveExclusion|CE	CE-Bacterial Secretion	CE-Type III Secretion Systems	CE-T3SS-Flagellar Export Apparatus	fliR|lfiR->PGPT0015360	1
Indirect Effects	CompetitiveExclusion|CE	CE-Bacterial Secretion	CE-Type II Secretion Systems	CE-T2SS-Competence-Related DNA Transformation transport	comC->PGPT0029470	1
Indirect Effects	CompetitiveExclusion|CE	CE-Bacterial Secretion	CE-Type II Secretion Systems	CE-T2SS-Competence-Related DNA Transformation transport	comEA->PGPT0029410	1
Indirect Effects	CompetitiveExclusion|CE	CE-Bacterial Secretion	CE-Type II Secretion Systems	CE-T2SS-Competence-Related DNA Transformation transport	comEB|tadA->PGPT0021380	1
Indirect	Competiti	CE-Bacterial Secretion	CE-Type II Secretion	CE-T2SS-Competence-	comEC->PGPT0029415	2

Effects	veExclusion|CE		Systems	Related DNA Transformation transport		
Indirect Effects	CompetitiveExclusion|CE	CE-Bacterial Secretion	CE-Type II Secretion Systems	CE-T2SS-Competence-Related DNA Transformation transport	comER->PGPT0029420	1
Indirect Effects	CompetitiveExclusion|CE	CE-Bacterial Secretion	CE-Type II Secretion Systems	CE-T2SS-Competence-Related DNA Transformation transport	comFA->PGPT0029425	1
Indirect Effects	CompetitiveExclusion|CE	CE-Bacterial Secretion	CE-Type II Secretion Systems	CE-T2SS-Competence-Related DNA Transformation transport	comGA->PGPT0029440	1
Indirect Effects	CompetitiveExclusion|CE	CE-Bacterial Secretion	CE-Type II Secretion Systems	CE-T2SS-Competence-Related DNA Transformation transport	comGB->PGPT0029445	1
Indirect Effects	CompetitiveExclusion|CE	CE-Bacterial Secretion	CE-Type II Secretion Systems	CE-T2SS-Competence-Related DNA Transformation transport	comGC->PGPT0029450	1
Indirect Effects	CompetitiveExclusion|CE	CE-Bacterial Secretion	CE-Type II Secretion Systems	CE-T2SS-Competence-Related DNA Transformation transport	comGD->PGPT0029455	1
Indirect Effects	CompetitiveExclusion|CE	CE-Bacterial Secretion	CE-Type II Secretion Systems	CE-T2SS-Competence-Related DNA Transformation transport	comGF->PGPT0029465	1
Indirect	Competiti	CE-Bacterial Secretion	CE-Type II Secretion	CE-T2SS-Competence-	comK->PGPT0025815	1

Effects	veExclusion|CE		Systems	Related DNA Transformation transport		
Indirect Effects	CompetitiveExclusion|CE	CE-Bacterial Secretion	CE-Type II Secretion Systems	CE-T2SS-Competence-Related DNA Transformation transport	comZ->PGPT0029475	1
Indirect Effects	CompetitiveExclusion|CE	CE-Bacterial Secretion	CE-Type II Secretion Systems	CE-T2SS-Type IVa Pilus Homolog Protein	yggT|ylmG->PGPT0013730	1
Indirect Effects	CompetitiveExclusion|CE	CE-Bacterial Secretion	CE-Type IV Secretion Systems	CE-T4SS-Conjugal DNA-Protein Transfer	virD4|lvhD4->PGPT0029925	1
Indirect Effects	CompetitiveExclusion|CE	CE-Bacterial Secretion	CE-Type IV Secretion Systems	CE-T4SS-Conjugal Transfer Pilus Assembly Protein	trbI->PGPT0030000	1
Indirect Effects	CompetitiveExclusion|CE	CE-Bacterial Secretion	CE-Type I Secretion Systems	CE-TSS1-Alpha-Hemolysin|Cyclolysin transport	hlyB|cyaB->PGPT0029360	1
Indirect Effects	CompetitiveExclusion|CE	CE-Bacterial Secretion	CE-Type VI Secretion Systems	CE-Type VI Secretion-ESS System	essB->PGPT0030467	1
Indirect Effects	CompetitiveExclusion|CE	CE-Bacterial Secretion	CE-Type VI Secretion Systems	CE-Type VI Secretion-ESS System	essC|eccC|ftsK|spoIIIE->PGPT0030468	8
Indirect Effects	CompetitiveExclusion|CE	CE-Bacterial Secretion	CE-Type VI Secretion Systems	CE-Type VI Secretion-ESS System	esxA->PGPT0030476	1
Indirect Effects	CompetitiveExclusion|CE	CE-Bacterial Secretion	CE-Type VI Secretion Systems	CE-Type VI Secretion Related Proteins	ppa|prpA->PGPT0030420	2
Indirect	Competiti	CE-Bacterial Secretion	CE-Type V Secretion	CE-T5a	pspA->PGPT0030320	1

Effects	veExclusion|CE		Systems	Autotransporter Secretion		
Indirect Effects	CompetitiveExclusion|CE	CE-Cell Envelope Remodelling	CE-ArabinoGalactan|Lipoarabinomannan Remodelling	CE-Remodelling Phosphate Transferase Activity	wecA|tagO|rfe->PGPT0015240	1
Indirect Effects	CompetitiveExclusion|CE	CE-Cell Envelope Remodelling	CE-Envelope Remodelling Regulation	CE-Envelope Remodelling Regulation Factor	paiB|yumE->PGPT0014760	1
Indirect Effects	CompetitiveExclusion|CE	CE-Cell Envelope Remodelling	CE-Envelope Remodelling Regulation	CE-Envelope Remodelling Regulation Factor	walR->PGPT0014763	1
Indirect Effects	CompetitiveExclusion|CE	CE-Cell Envelope Remodelling	CE-Glycerolipid Remodelling	CE-Remodelling Glycerolipid-Lipoteichoic Acid Synthase	ltaS->PGPT0023375	3
Indirect Effects	CompetitiveExclusion|CE	CE-Cell Envelope Remodelling	CE-Glycerolipid Remodelling	CE-Remodelling Glycerolipid Acyltransferase Activity	plsC->PGPT0024380	3
Indirect Effects	CompetitiveExclusion|CE	CE-Cell Envelope Remodelling	CE-Glycerolipid Remodelling	CE-Remodelling Glycerolipid Acyltransferase Activity	plsX->PGPT0024410	1
Indirect Effects	CompetitiveExclusion|CE	CE-Cell Envelope Remodelling	CE-Glycerolipid Remodelling	CE-Remodelling Glycerolipid Acyltransferase Activity	plsY->PGPT0024375	2
Indirect Effects	CompetitiveExclusion|CE	CE-Cell Envelope Remodelling	CE-Glycerolipid Remodelling	CE-Remodelling Glycerolipid Dehydrogenase|Dehydratase Activity	aldH|dhaS->PGPT0006875	5

Indirect Effects	CompetitiveExclusion|CE	CE-Cell Envelope Remodelling	CE-Glycerolipid Remodelling	CE-Remodelling Glycerolipid Dehydrogenase|Dehydratase Activity	gldA|dhaD->PGPT0008270	1
Indirect Effects	CompetitiveExclusion|CE	CE-Cell Envelope Remodelling	CE-Glycerolipid Remodelling	CE-Remodelling Glycerolipid Dehydrogenase|Dehydratase Activity	yahK->PGPT0024430	1
Indirect Effects	CompetitiveExclusion|CE	CE-Cell Envelope Remodelling	CE-Glycerolipid Remodelling	CE-Remodelling Glycerolipid Galactosidase Activity	melA->PGPT0017850	1
Indirect Effects	CompetitiveExclusion|CE	CE-Cell Envelope Remodelling	CE-Glycerolipid Remodelling	CE-Remodelling Glycerolipid Galactosidase Activity	rafA|galA->PGPT0018835	1
Indirect Effects	CompetitiveExclusion|CE	CE-Cell Envelope Remodelling	CE-Glycerolipid Remodelling	CE-Remodelling Glycerolipid GlucosylTransferase Activity	ugtP->PGPT0023400	2
Indirect Effects	CompetitiveExclusion|CE	CE-Cell Envelope Remodelling	CE-Glycerolipid Remodelling	CE-Remodelling Glycerolipid Kinase Activity	dagK->PGPT0024345	2
Indirect Effects	CompetitiveExclusion|CE	CE-Cell Envelope Remodelling	CE-Glycerolipid Remodelling	CE-Remodelling Glycerolipid Kinase Activity	dhaK 2->PGPT0018465	1
Indirect Effects	CompetitiveExclusion|CE	CE-Cell Envelope Remodelling	CE-Glycerolipid Remodelling	CE-Remodelling Glycerolipid Kinase Activity	garK|glxK->PGPT0018175	1
Indirect Effects	CompetitiveExclusion|CE	CE-Cell Envelope Remodelling	CE-Glycerolipid Remodelling	CE-Remodelling Glycerolipid Kinase Activity	glpK->PGPT0018435	1
Indirect Effects	CompetitiveExclusi	CE-Cell Envelope Remodelling	CE-Glycerolipid Remodelling	CE-Remodelling Glycerolipid Lipase	lip|lipC->PGPT0024445	1

	on|CE			Activity		
Indirect Effects	CompetitiveExclusion|CE	CE-Cell Envelope Remodelling	CE-GlycerophosphoLipid Remodelling	CE-Remodelling GPL CytidylylTransferase Activity	cdsA|ynbB->PGPT0007685	1
Indirect Effects	CompetitiveExclusion|CE	CE-Cell Envelope Remodelling	CE-GlycerophosphoLipid Remodelling	CE-Remodelling GPL Decarboxylase Activity	psd|PISD->PGPT0007700	1
Indirect Effects	CompetitiveExclusion|CE	CE-Cell Envelope Remodelling	CE-GlycerophosphoLipid Remodelling	CE-Remodelling GPL Dehydrogenase Activity	glpA|glpD->PGPT0006775	1
Indirect Effects	CompetitiveExclusion|CE	CE-Cell Envelope Remodelling	CE-GlycerophosphoLipid Remodelling	CE-Remodelling GPL Dehydrogenase Activity	gpsA->PGPT0024330	1
Indirect Effects	CompetitiveExclusion|CE	CE-Cell Envelope Remodelling	CE-GlycerophosphoLipid Remodelling	CE-Remodelling GPL Desaturase Activity	desA->PGPT0008400	2
Indirect Effects	CompetitiveExclusion|CE	CE-Cell Envelope Remodelling	CE-GlycerophosphoLipid Remodelling	CE-Remodelling GPL Diacylglicerol Kinase Activity	dagK->PGPT0024345	2
Indirect Effects	CompetitiveExclusion|CE	CE-Cell Envelope Remodelling	CE-GlycerophosphoLipid Remodelling	CE-Remodelling GPL GlycerophosphoLipid-Cardiolipin Synthase Activity	clsA B|ybhO|ywiE->PGPT0007725	5
Indirect Effects	CompetitiveExclusion|CE	CE-Cell Envelope Remodelling	CE-GlycerophosphoLipid Remodelling	CE-Remodelling GPL GlycerophosphoLipid-Cardiolipin Synthase Activity	clsC|ymdC->PGPT0007730	2
Indirect Effects	CompetitiveExclusion|CE	CE-Cell Envelope Remodelling	CE-GlycerophosphoLipid Remodelling	CE-Remodelling GPL GlycerophosphoLipid-EthanolAmine Ammonia-Lyase Activity	eutB->PGPT0000605	1

Indirect Effects	CompetitiveExclusion|CE	CE-Cell Envelope Remodelling	CE-GlycerophosphoLipid Remodelling	CE-Remodelling GPL GlycerophosphoLipid-EthanolAmine Ammonia-Lyase Activity	eutC->PGPT0000610	1
Indirect Effects	CompetitiveExclusion|CE	CE-Cell Envelope Remodelling	CE-GlycerophosphoLipid Remodelling	CE-Remodelling GPL GlycerophosphoLipid-EthanolAmine Utilization	eutA->PGPT0000600	1
Indirect Effects	CompetitiveExclusion|CE	CE-Cell Envelope Remodelling	CE-GlycerophosphoLipid Remodelling	CE-Remodelling GPL LysophosphoLipase Activity	pldB->PGPT0023520	4
Indirect Effects	CompetitiveExclusion|CE	CE-Cell Envelope Remodelling	CE-GlycerophosphoLipid Remodelling	CE-Remodelling GPL MethylTransferase Activity	pmtA->PGPT0007740	3
Indirect Effects	CompetitiveExclusion|CE	CE-Cell Envelope Remodelling	CE-GlycerophosphoLipid Remodelling	CE-Remodelling GPL Phosphatase Activity	pgpA->PGPT0007710	2
Indirect Effects	CompetitiveExclusion|CE	CE-Cell Envelope Remodelling	CE-GlycerophosphoLipid Remodelling	CE-Remodelling GPL Phosphate Acyltransferase Activity	plsC->PGPT0024380	3
Indirect Effects	CompetitiveExclusion|CE	CE-Cell Envelope Remodelling	CE-GlycerophosphoLipid Remodelling	CE-Remodelling GPL Phosphate Acyltransferase Activity	plsY->PGPT0024375	2
Indirect Effects	CompetitiveExclusion|CE	CE-Cell Envelope Remodelling	CE-GlycerophosphoLipid Remodelling	CE-Remodelling GPL Phosphate Synthase Activity	pcrB->PGPT0024385	1
Indirect Effects	CompetitiveExclusion|CE	CE-Cell Envelope Remodelling	CE-GlycerophosphoLipid Remodelling	CE-Remodelling GPL Phosphatidyltransferase Activity	CHO1|pssA->PGPT0007695	3

Indirect Effects	CompetitiveExclusion|CE	CE-Cell Envelope Remodelling	CE-GlycerophosphoLipid Remodelling	CE-Remodelling GPL Phosphodiesterase Activity	glpQ|ugpQ->PGPT0018470	5
Indirect Effects	CompetitiveExclusion|CE	CE-Cell Envelope Remodelling	CE-Other Cell Membrane Remodelling Proteins	CE-Remodelling Lipoprotein Metabolism	rlpA->PGPT0024465	1
Indirect Effects	CompetitiveExclusion|CE	CE-Cell Envelope Remodelling	CE-Other Cell Membrane Remodelling Proteins	Other Integral Membrane Remodelling Proteins	yidC|spoIIIJ|oxaA|ccfA->PGPT0024530	3
Indirect Effects	CompetitiveExclusion|CE	CE-Cell Envelope Remodelling	CE-Other Cell Membrane Remodelling Proteins	Other Integral Membrane Remodelling Proteins	yozB ->PGPT0024520	1
Indirect Effects	CompetitiveExclusion|CE	CE-Cell Envelope Remodelling	CE-Other Cell Membrane Remodelling Proteins	Other Integral Membrane Remodelling Proteins	yuiD->PGPT0024535	2
Indirect Effects	CompetitiveExclusion|CE	CE-Cell Envelope Remodelling	CE-Other Remodelling Surface Glycosylation Patterns	CE-Remodelling S-Layer Glycoprotein Decoration	aglJ->PGPT0024610	1
Indirect Effects	CompetitiveExclusion|CE	CE-Cell Envelope Remodelling	CE-Peptidoglycan Remodelling	CE-PG Remodelling-CarboxyPeptidase Activity	dacC|dacA|dacD->PGPT0024040	3
Indirect Effects	CompetitiveExclusion|CE	CE-Cell Envelope Remodelling	CE-Peptidoglycan Remodelling	CE-PG Remodelling-CarboxyPeptidase Activity	vanY|yodJ->PGPT0024055	3
Indirect Effects	CompetitiveExclusion|CE	CE-Cell Envelope Remodelling	CE-Peptidoglycan Remodelling	CE-PG Remodelling-Dipeptidyl-Peptidase Activity	ykfC->PGPT0020945	2
Indirect Effects	CompetitiveExclusion|CE	CE-Cell Envelope Remodelling	CE-Peptidoglycan Remodelling	CE-PG Remodelling-L|D transpeptidase Activity	erfK->PGPT0023745	2
Indirect Effects	CompetitiveExclusi	CE-Cell Envelope Remodelling	CE-Peptidoglycan Remodelling	CE-PG Remodelling-L|D transpeptidase	ynhG->PGPT0023765	1

	on|CE			Activity		
Indirect Effects	CompetitiveExclusion|CE	CE-Cell Envelope Remodelling	CE-Peptidoglycan Remodelling	CE-PG Remodelling-Muramoyltetrapeptide CarboxyPeptidase Activity	ldcA->PGPT0023815	2
Indirect Effects	CompetitiveExclusion|CE	CE-Cell Envelope Remodelling	CE-Peptidoglycan Remodelling	CE-PG Remodelling-N Acetylglucosamine Modification	murA->PGPT0024090	2
Indirect Effects	CompetitiveExclusion|CE	CE-Cell Envelope Remodelling	CE-Peptidoglycan Remodelling	CE-PG Remodelling-N AcetylMuramate Modification	ampD->PGPT0024100	1
Indirect Effects	CompetitiveExclusion|CE	CE-Cell Envelope Remodelling	CE-Peptidoglycan Remodelling	CE-PG Remodelling-N AcetylMuramate Modification	ddl->PGPT0020050	2
Indirect Effects	CompetitiveExclusion|CE	CE-Cell Envelope Remodelling	CE-Peptidoglycan Remodelling	CE-PG Remodelling-N AcetylMuramate Modification	mraY->PGPT0024105	1
Indirect Effects	CompetitiveExclusion|CE	CE-Cell Envelope Remodelling	CE-Peptidoglycan Remodelling	CE-PG Remodelling-N AcetylMuramate Modification	murB->PGPT0024115	1
Indirect Effects	CompetitiveExclusion|CE	CE-Cell Envelope Remodelling	CE-Peptidoglycan Remodelling	CE-PG Remodelling-N AcetylMuramate Modification	murD->PGPT0024125	1
Indirect Effects	CompetitiveExclusion|CE	CE-Cell Envelope Remodelling	CE-Peptidoglycan Remodelling	CE-PG Remodelling-N AcetylMuramate Modification	murE->PGPT0024130	2
Indirect Effects	CompetitiveExclusion|CE	CE-Cell Envelope Remodelling	CE-Peptidoglycan Remodelling	CE-PG Remodelling-N AcetylMuramate Modification	murF->PGPT0024145	2
Indirect Effects	CompetitiveExclusion|CE	CE-Cell Envelope Remodelling	CE-Peptidoglycan Remodelling	CE-PG Remodelling-N AcetylMuramate Modification	murG->PGPT0024150	1

Indirect Effects	CompetitiveExclusion|CE	CE-Cell Envelope Remodelling	CE-Peptidoglycan Remodelling	CE-PG Remodelling-Penicillin-Binding Proteins	mrcA->PGPT0023875	3
Indirect Effects	CompetitiveExclusion|CE	CE-Cell Envelope Remodelling	CE-Peptidoglycan Remodelling	CE-PG Remodelling-Penicillin-Binding Proteins	pbp2A->PGPT0023895	2
Indirect Effects	CompetitiveExclusion|CE	CE-Cell Envelope Remodelling	CE-Peptidoglycan Remodelling	CE-PG Remodelling-Penicillin-Binding Proteins	pbpA->PGPT0023930	2
Indirect Effects	CompetitiveExclusion|CE	CE-Cell Envelope Remodelling	CE-Peptidoglycan Remodelling	CE-PG Remodelling-Penicillin-Binding Proteins	pbpB->PGPT0023935	1
Indirect Effects	CompetitiveExclusion|CE	CE-Cell Envelope Remodelling	CE-Peptidoglycan Remodelling	CE-PG Remodelling-Penicillin-Binding Proteins	pbpC->PGPT0023945	1
Indirect Effects	CompetitiveExclusion|CE	CE-Cell Envelope Remodelling	CE-Peptidoglycan Remodelling	CE-PG Remodelling-Penicillin-Binding Proteins	pbpG->PGPT0023955	1
Indirect Effects	CompetitiveExclusion|CE	CE-Cell Envelope Remodelling	CE-Peptidoglycan Remodelling	CE-PG Remodelling-Penicillin-Binding Proteins	pbpI->PGPT0023965	1
Indirect Effects	CompetitiveExclusion|CE	CE-Cell Envelope Remodelling	CE-Peptidoglycan Remodelling	CE-PG Remodelling-Stage V Sporulation Related Protein	spoVD->PGPT0024085	1
Indirect Effects	CompetitiveExclusion|CE	CE-Cell Envelope Remodelling	CE-Peptidoglycan Remodelling	CE-PG Remodelling-Undecaprenol Modification	bacA->PGPT0024165	3
Indirect Effects	CompetitiveExclusion|CE	CE-Cell Envelope Remodelling	CE-Peptidoglycan Remodelling	CE-PG Remodelling-Undecaprenol Modification	bcrC->PGPT0024170	8
Indirect Effects	CompetitiveExclusi	CE-Cell Envelope Remodelling	CE-Peptidoglycan Remodelling	CE-PG Remodelling-Undecaprenol	dgkA->PGPT0024175	1

	on|CE			Modification		
Indirect Effects	CompetitiveExclusion|CE	CE-Cell Envelope Remodelling	CE-Peptidoglycan Remodelling	CE-PG Remodelling-Undecaprenol Modification	uppS|ispU->PGPT0024180	1
Indirect Effects	CompetitiveExclusion|CE	CE-Cell Envelope Remodelling	CE-Peptidoglycan Remodelling	CE-PG Remodelling Amidase Activity	amiA|amiB|amiC->PGPT0024160	9
Indirect Effects	CompetitiveExclusion|CE	CE-Cell Envelope Remodelling	CE-Peptidoglycan Remodelling	CE-PG Remodelling DL-Endopeptidase Activity	cwlO->PGPT0023995	2
Indirect Effects	CompetitiveExclusion|CE	CE-Cell Envelope Remodelling	CE-Peptidoglycan Remodelling	CE-PG Remodelling DL-Endopeptidase Activity	lytE|cwlF|yocH->PGPT0024005	8
Indirect Effects	CompetitiveExclusion|CE	CE-Cell Envelope Remodelling	CE-Peptidoglycan Remodelling	CE-PG Remodelling DL-Endopeptidase Activity	lytF|cwlE->PGPT0024010	3
Indirect Effects	CompetitiveExclusion|CE	CE-Cell Envelope Remodelling	CE-Peptidoglycan Remodelling	CE-PG Remodelling Hydrolase Activity	icaB->PGPT0023970	1
Indirect Effects	CompetitiveExclusion|CE	CE-Cell Envelope Remodelling	CE-Peptidoglycan Remodelling	CE-PG Remodelling Hydrolase Activity	pda|pgdA->PGPT0018645	8
Indirect Effects	CompetitiveExclusion|CE	CE-Cell Envelope Remodelling	CE-Peptidoglycan Remodelling	CE-PG Remodelling Hydrolase Activity	sle1|yocH->PGPT0019120	3
Indirect Effects	CompetitiveExclusion|CE	CE-Cell Envelope Remodelling	CE-Peptidoglycan Remodelling	CE-PG Remodelling Hydrolase Activity	xlyAB->PGPT0019115	1
Indirect Effects	CompetitiveExclusion|CE	CE-Cell Envelope Remodelling	CE-Peptidoglycan Remodelling	CE-PG Remodelling LD-Endopeptidase Activity	cwlK->PGPT0024025	2
Indirect	Competiti	CE-Cell Envelope	CE-Peptidoglycan	CE-PG Remodelling	lytH->PGPT0024030	1

Effects	veExclusion|CE	Remodelling	Remodelling	LD-Endopeptidase Activity		
Indirect Effects	CompetitiveExclusion|CE	CE-Cell Envelope Remodelling	CE-Peptidoglycan Remodelling	CE-PG Remodelling Lipid II Flippase Activity	murJ|mviN->PGPT0024200	1
Indirect Effects	CompetitiveExclusion|CE	CE-Cell Envelope Remodelling	CE-Peptidoglycan Remodelling	CE-PG Remodelling Related Racemases	alr->PGPT0020040	1
Indirect Effects	CompetitiveExclusion|CE	CE-Cell Envelope Remodelling	CE-Peptidoglycan Remodelling	CE-PG Remodelling Related Racemases	murI->PGPT0020200	1
Indirect Effects	CompetitiveExclusion|CE	CE-Cell Envelope Remodelling	CE-Peptidoglycan Remodelling	CE-PG Remodelling Related Sortase	srtA->PGPT0024075	4
Indirect Effects	CompetitiveExclusion|CE	CE-Cell Envelope Remodelling	CE-RemodellingLipo-|Colanic Acid Production	CE-Remodelling Lipoteichoic Acid Metabolism	ltaS->PGPT0023375	3
Indirect Effects	CompetitiveExclusion|CE	CE-Cell Envelope Remodelling	CE-RemodellingLipo-|Colanic Acid Production	CE-Remodelling Lipoteichoic Acid Metabolism	ugtP->PGPT0023400	2
Indirect Effects	CompetitiveExclusion|CE	CE-Cell Envelope Remodelling	CE-RemodellingLipo-|Colanic Acid Production	CE-Remodelling Teichoic Acid Metabolism	fmtA->PGPT0023445	1
Indirect Effects	CompetitiveExclusion|CE	CE-Cell Envelope Remodelling	CE-RemodellingLipo-|Colanic Acid Production	CE-Remodelling Teichoic Acid Metabolism	gtaC->PGPT0023460	2
Indirect Effects	CompetitiveExclusion|CE	CE-Cell Envelope Remodelling	CE-RemodellingLipo-|Colanic Acid Production	CE-Remodelling Teichoic Acid Metabolism	Taga|tarA->PGPT0023455	2
Indirect Effects	CompetitiveExclusion|CE	CE-Cell Envelope Remodelling	CE-RemodellingLipo-|Colanic Acid Production	CE-Remodelling Teichoic Acid Metabolism	tagT|tagU|tagV->PGPT0023440	7

Indirect Effects	CompetitiveExclusion|CE	CE-Cell Envelope Remodelling	CE-RemodellingLipo-|Colanic Acid Production	CE-Remodelling Teichoic Acid Metabolism	wecB->PGPT0018905	2
Indirect Effects	CompetitiveExclusion|CE	CE-Cell Envelope Remodelling	CE-RemodellingLipo-|Teichuronic Acid Metabolism	CE-Remodelling Teichuronic Acid Metabolism	tuaG|ggaB->PGPT0015205	2
Indirect Effects	CompetitiveExclusion|CE	CE-Cell Envelope Remodelling	CE-RemodellingLipo-|Teichuronic Acid Metabolism	CE-Remodelling Teichuronic Acid Metabolism	wecA|tagO|rfe->PGPT0015240	1
Indirect Effects	CompetitiveExclusion|CE	CE-Cell Envelope Remodelling	CE-RemodellingLPS|Lipid|IVA Metabolism	CE-Remodelling Lipid A Metabolism	lpxG->PGPT0022365	3
Indirect Effects	CompetitiveExclusion|CE	CE-Cell Envelope Remodelling	CE-RemodellingLPS|Lipid|IVA Metabolism	CE-Remodelling LPS-GDP D-Rhamnose|Deoxy D Mannose Modification	rmd->PGPT0022780	1
Indirect Effects	CompetitiveExclusion|CE	CE-Cell Envelope Remodelling	CE-RemodellingLPS|Lipid|IVA Metabolism	CE-Remodelling LPS-GDP Mannose Modification	manC|cpsB->PGPT0022660	1
Indirect Effects	CompetitiveExclusion|CE	CE-Cell Envelope Remodelling	CE-RemodellingLPS|Lipid|IVA Metabolism	CE-Remodelling LPS-Glucosamine Modification	glmU->PGPT0018955	1
Indirect Effects	CompetitiveExclusion|CE	CE-Cell Envelope Remodelling	CE-RemodellingLPS|Lipid|IVA Metabolism	CE-Remodelling LPS-Glucose-Mannose-Frucose Conversion Modification	manA->PGPT0017860	1
Indirect Effects	CompetitiveExclusion|CE	CE-Cell Envelope Remodelling	CE-RemodellingLPS|Lipid|IVA Metabolism	CE-Remodelling LPS-Manno-Heptose|-Phosphate Modification	gmhB|yaeD->PGPT0023035	1
Indirect Effects	CompetitiveExclusi	CE-Cell Envelope Remodelling	CE-RemodellingLPS|Lipi	CE-Remodelling LPS-O-Antiogen	csbB|gtrB|yfdH->PGPT0014540	4

	on|CE		d|IVA Metabolism	Biosynthesis		
Indirect Effects	CompetitiveExclusion|CE	CE-Cell Envelope Remodelling	CE-RemodellingLPS|Lipid|IVA Metabolism	CE-Remodelling LPS-O-Antiogen Biosynthesis	wecA|tagO|rfe->PGPT0015240	1
Indirect Effects	CompetitiveExclusion|CE	CE-Cell Envelope Remodelling	CE-RemodellingLPS|Lipid|IVA Metabolism	CE-Remodelling LPS-O-Antiogen Biosynthesis	wecB->PGPT0018905	2
Indirect Effects	CompetitiveExclusion|CE	CE-Cell Envelope Remodelling	CE-RemodellingLPS|Lipid|IVA Metabolism	CE-Remodelling LPS-O-Antiogen Biosynthesis	wecC->PGPT0022670	1
Indirect Effects	CompetitiveExclusion|CE	CE-Cell Envelope Remodelling	CE-RemodellingLPS|Lipid|IVA Metabolism	CE-Remodelling LPS-Pseudaminic Acid Modification	pseB|wbjB->PGPT0018935	1
Indirect Effects	CompetitiveExclusion|CE	CE-Cell Envelope Remodelling	CE-RemodellingLPS|Lipid|IVA Metabolism	CE-Remodelling LPS-Putative Trylose Derivates Modification	rfbD|rmlD->PGPT0022630	1
Indirect Effects	CompetitiveExclusion|CE	CE-Cell Envelope Remodelling	CE-RemodellingLPS|Lipid|IVA Metabolism	CE-Remodelling LPS-UDP-Galactose-Glucuronate Pool Modification	cap1J|wbgU->PGPT0019020	1
Indirect Effects	CompetitiveExclusion|CE	CE-Cell Envelope Remodelling	CE-RemodellingLPS|Lipid|IVA Metabolism	CE-Remodelling LPS D Galactofuranose Modification	galT->PGPT0017835	1
Indirect Effects	CompetitiveExclusion|CE	CE-Cell Envelope Remodelling	CE-RemodellingLPS|Lipid|IVA Metabolism	CE-Remodelling LPS D Mannosaminuronic Acid Modification	wecB->PGPT0018905	2
Indirect Effects	CompetitiveExclusion|CE	CE-Cell Envelope Remodelling	CE-RemodellingLPS|Lipid|IVA Metabolism	CE-Remodelling LPS D Mannosaminuronic Acid Modification	wecC->PGPT0022670	1
Indirect Effects	CompetitiveExclusion|CE	CE-Cell Envelope Remodelling	CE-RemodellingLPS|Lipid|IVA Metabolism	CE-Remodelling LPS D Mannuronic Acid Modification	wbpA->PGPT0018920	1

Indirect Effects	CompetitiveExclusion|CE	CE-Cell Envelope Remodelling	CE-RemodellingLPS|Lipid|IVA Metabolism	CE-Remodelling LPS D Mannuronic Acid Modification	wlbA|bplA->PGPT0022715	3
Indirect Effects	CompetitiveExclusion|CE	CE-Cell Envelope Remodelling	CE-RemodellingLPS|Lipid|IVA Metabolism	CE-Remodelling LPS L Ara4N Modification	arnE->PGPT0022825	1
Indirect Effects	CompetitiveExclusion|CE	CE-Exopolysaccharide Production|EPS	CE-EPS-Capsule Metabolism	CE-EPS-Capsule Biosynthesis	capA|pgsA->PGPT0026660	2
Indirect Effects	CompetitiveExclusion|CE	CE-Exopolysaccharide Production|EPS	CE-EPS-Capsule Metabolism	CE-EPS-Capsule Biosynthesis	capC|pgsC->PGPT0026670	1
Indirect Effects	CompetitiveExclusion|CE	CE-Exopolysaccharide Production|EPS	CE-EPS-Colanic Acid Metabolism	CE-EPS-Capsular Colanic Acid Biosynthesis	TC GPH|yihO|xynP->PGPT0014410	3
Indirect Effects	CompetitiveExclusion|CE	CE-Exopolysaccharide Production|EPS	CE-EPS-Colanic Acid Metabolism	CE-EPS-Colanic Acid Related Proteins	manC|cpsB->PGPT0022660	1
Indirect Effects	CompetitiveExclusion|CE	CE-Exopolysaccharide Production|EPS	CE-EPS-Exopolysaccharide transport	CE-EPS-Exopolysaccharide transport-1	Exop|vpsO->PGPT0026560	2
Indirect Effects	CompetitiveExclusion|CE	CE-Exopolysaccharide Production|EPS	CE-EPS-Glycan Metabolism	CE-EPS-GalactoGlucan Metabolic Pathway	mucR->PGPT0026010	3
Indirect Effects	CompetitiveExclusion|CE	CE-Exopolysaccharide Production|EPS	CE-EPS-Glycan Metabolism	CE-EPS-GalactoGlucan Metabolic Pathway	phoB->PGPT0002660	1
Indirect Effects	CompetitiveExclusion|CE	CE-Exopolysaccharide Production|EPS	CE-EPS-Glycan Metabolism	CE-EPS-GalactoGlucan Metabolic Pathway	phoP|phoB1->PGPT0002665	4
Indirect Effects	CompetitiveExclusi	CE-Exopolysaccharide Production|EPS	CE-EPS-Glycan Metabolism	CE-EPS-GalactoGlucan	sinI->PGPT0026295	2

	on|CE			Metabolic Pathway		
Indirect Effects	CompetitiveExclusion|CE	CE-Exopolysaccharide Production|EPS	CE-EPS-Glycan Metabolism	CE-EPS-Glycan Biosynthesis	DPM1 Like|arnC|ppm1|wcaA->PGPT0017834	2
Indirect Effects	CompetitiveExclusion|CE	CE-Exopolysaccharide Production|EPS	CE-EPS-Glycan Metabolism	CE-EPS-Glycan Biosynthesis	galT->PGPT0017835	1
Indirect Effects	CompetitiveExclusion|CE	CE-Exopolysaccharide Production|EPS	CE-EPS-Glycan Metabolism	CE-EPS-Glycan Biosynthesis	glgC->PGPT0025885	2
Indirect Effects	CompetitiveExclusion|CE	CE-Exopolysaccharide Production|EPS	CE-EPS-Glycan Metabolism	CE-EPS-Succinoglycan Metabolic Pathway	Exop|vpsO->PGPT0026560	2
Indirect Effects	CompetitiveExclusion|CE	CE-Exopolysaccharide Production|EPS	CE-EPS-Glycan Metabolism	CE-EPS-Succinoglycan Metabolic Pathway	exoY->PGPT0026575	1
Indirect Effects	CompetitiveExclusion|CE	CE-Exopolysaccharide Production|EPS	CE-EPS-Glycogen Metabolism	CE-EPS-Glycogen Biosynthesis	glgA->PGPT0025880	1
Indirect Effects	CompetitiveExclusion|CE	CE-Exopolysaccharide Production|EPS	CE-EPS-Glycogen Metabolism	CE-EPS-Glycogen Biosynthesis	glgC->PGPT0025885	2
Indirect Effects	CompetitiveExclusion|CE	CE-Exopolysaccharide Production|EPS	CE-EPS-Glycogen Metabolism	CE-EPS-Glycogen Biosynthesis	glgP->PGPT0018545	1
Indirect Effects	CompetitiveExclusion|CE	CE-Exopolysaccharide Production|EPS	CE-EPS-Glycogen Metabolism	CE-EPS-Glycogen Biosynthesis	yqgM->PGPT0018547	2
Indirect Effects	CompetitiveExclusion|CE	CE-Exopolysaccharide Production|EPS	CE-EPS-Glycosidases|Glycosyl Hydrolases	CE-EPS-Amylase	amyA|malS->PGPT0018575	3
Indirect	Competiti	CE-Exopolysaccharide	CE-EPS-	CE-EPS-Amylase	Beta Amylase-	1

Effects	veExclusion|CE	Production|EPS	Glycosidases|Glycosyl Hydrolases		>PGPT0018580	
Indirect Effects	CompetitiveExclusion|CE	CE-Exopolysaccharide Production|EPS	CE-EPS-Glycosidases|Glycosyl Hydrolases	CE-EPS-Carrageenase	celF|licH|chbF->PGPT0019105	3
Indirect Effects	CompetitiveExclusion|CE	CE-Exopolysaccharide Production|EPS	CE-EPS-Glycosidases|Glycosyl Hydrolases	CE-EPS-Fructofuranosidase	sacA->PGPT0018815	2
Indirect Effects	CompetitiveExclusion|CE	CE-Exopolysaccharide Production|EPS	CE-EPS-Glycosidases|Glycosyl Hydrolases	CE-EPS-Fructosidase	fruA->PGPT0018745	1
Indirect Effects	CompetitiveExclusion|CE	CE-Exopolysaccharide Production|EPS	CE-EPS-Glycosidases|Glycosyl Hydrolases	CE-EPS-Galactosidase	bgaB|lacA->PGPT0017815	1
Indirect Effects	CompetitiveExclusion|CE	CE-Exopolysaccharide Production|EPS	CE-EPS-Glycosidases|Glycosyl Hydrolases	CE-EPS-Galactosidase	bglA->PGPT0019255	2
Indirect Effects	CompetitiveExclusion|CE	CE-Exopolysaccharide Production|EPS	CE-EPS-Glycosidases|Glycosyl Hydrolases	CE-EPS-Galactosidase	lacZ->PGPT0017810	1
Indirect Effects	CompetitiveExclusion|CE	CE-Exopolysaccharide Production|EPS	CE-EPS-Glycosidases|Glycosyl Hydrolases	CE-EPS-Galactosidase	melA->PGPT0017850	1
Indirect Effects	CompetitiveExclusion|CE	CE-Exopolysaccharide Production|EPS	CE-EPS-Glycosidases|Glycosyl Hydrolases	CE-EPS-Galactosidase	rafA|galA->PGPT0018835	1
Indirect Effects	CompetitiveExclusion|CE	CE-Exopolysaccharide Production|EPS	CE-EPS-Glycosidases|Glycosyl Hydrolases	CE-EPS-Hydrolase	yteR|yesR->PGPT0018255	1
Indirect Effects	CompetitiveExclusion|CE	CE-Exopolysaccharide Production|EPS	CE-EPS-Glycosidases|Glycosyl Hydrolases	CE-EPS-Phosphotrehalase	treC->PGPT0014095	1

Indirect Effects	CompetitiveExclusion|CE	CE-Exopolysaccharide Production|EPS	CE-EPS-Glycosidases|Glycosyl Hydrolases	CE-EPS-Pullulanase	pulA->PGPT0012195	2
Indirect Effects	CompetitiveExclusion|CE	CE-Exopolysaccharide Production|EPS	CE-EPS-Levan Metabolism	CE-EPS-Levanbiose Biosynthesis	sacC|levB->PGPT0019220	1
Indirect Effects	CompetitiveExclusion|CE	CE-Exopolysaccharide Production|EPS	CE-EPS-Levan Metabolism	CE-EPS-Levan Biosynthesis	sacB->PGPT0013775	2
Indirect Effects	CompetitiveExclusion|CE	CE-Exopolysaccharide Production|EPS	CE-EPS-Lipo-|Teichuronic Acid Metabolism	CE-EPS-Teichuronic Acid Metabolic Pathway	tuaG|ggaB->PGPT0015205	2
Indirect Effects	CompetitiveExclusion|CE	CE-Exopolysaccharide Production|EPS	CE-EPS-Lipo-|Teichuronic Acid Metabolism	CE-EPS-Teichuronic Acid Metabolic Pathway	wecA|tagO|rfe->PGPT0015240	1
Indirect Effects	CompetitiveExclusion|CE	CE-Exopolysaccharide Production|EPS	CE-EPS-Poly-N-Acetyl-Glucosamine Metabolism	CE-EPS-Poly-N Acetyl-Glucosamine Biosynthesis	pgaC|icaA->PGPT0023485	3
Indirect Effects	CompetitiveExclusion|CE	CE-Exopolysaccharide Production|EPS	CE-EPS-PSL Polysaccharide Metabolism	CE-EPS-PSL PolysaccharideS Biosynthesis	pslH->PGPT0026045	1
Indirect Effects	CompetitiveExclusion|CE	CE-Exopolysaccharide Production|EPS	CE-EPS-PSS-Related Exopolysaccharide Metabolism	CE-EPS-PSS-Related Exopolysaccharide Biosynthesis	CHO1|pssA->PGPT0007695	3
Indirect Effects	CompetitiveExclusion|CE	CE-Exopolysaccharide Production|EPS	CE-EPS-Putative Exopolysaccharide Function	CE-EPS-Putative Exopolysaccharide Function-1	bshA->PGPT0019565	3
Indirect Effects	CompetitiveExclusion|CE	CE-Exopolysaccharide Production|EPS	CE-EPS-Putative Exopolysaccharide Function	CE-EPS-Putative Exopolysaccharide Function-1	cysE->PGPT0020265	1
Indirect Effects	CompetitiveExclusi	CE-Exopolysaccharide Production|EPS	CE-EPS-Putative Exopolysaccharide	CE-EPS-Putative Exopolysaccharide	murQ->PGPT0019040	1

	on|CE		Function	Function-1		
Indirect Effects	CompetitiveExclusion|CE	CE-Exopolysaccharide Production|EPS	CE-EPS-Teichoic Acid Metabolism	CE-EPS-Lipoteichoic Acid Metabolic Pathway	dltE->PGPT0013220	1
Indirect Effects	CompetitiveExclusion|CE	CE-Exopolysaccharide Production|EPS	CE-EPS-Teichoic Acid Metabolism	CE-EPS-Lipoteichoic Acid Metabolic Pathway	ltaS->PGPT0023375	3
Indirect Effects	CompetitiveExclusion|CE	CE-Exopolysaccharide Production|EPS	CE-EPS-Teichoic Acid Metabolism	CE-EPS-Lipoteichoic Acid Metabolic Pathway	ugtP->PGPT0023400	2
Indirect Effects	CompetitiveExclusion|CE	CE-Exopolysaccharide Production|EPS	CE-EPS-Teichoic Acid Metabolism	CE-EPS-Teichoic Acid Metabolic Pathway	fmtA->PGPT0023445	1
Indirect Effects	CompetitiveExclusion|CE	CE-Exopolysaccharide Production|EPS	CE-EPS-Teichoic Acid Metabolism	CE-EPS-Teichoic Acid Metabolic Pathway	gtaC->PGPT0023460	2
Indirect Effects	CompetitiveExclusion|CE	CE-Exopolysaccharide Production|EPS	CE-EPS-Teichoic Acid Metabolism	CE-EPS-Teichoic Acid Metabolic Pathway	Taga|tarA->PGPT0023455	2
Indirect Effects	CompetitiveExclusion|CE	CE-Exopolysaccharide Production|EPS	CE-EPS-Teichoic Acid Metabolism	CE-EPS-Teichoic Acid Metabolic Pathway	tagT|tagU|tagV->PGPT0023440	7
Indirect Effects	CompetitiveExclusion|CE	CE-Exopolysaccharide Production|EPS	CE-EPS-Teichoic Acid Metabolism	CE-EPS-Teichoic Acid Metabolic Pathway	wecB->PGPT0018905	2
Indirect Effects	CompetitiveExclusion|CE	CE-Exopolysaccharide Production|EPS	CE-EPS-UDP-N-Acetyl-D-Mannosamine Metabolism	CE-EPS-UDP-N Acetyl D Mannosamine Modification	wecB->PGPT0018905	2
Indirect Effects	CompetitiveExclusion|CE	CE-Exopolysaccharide Production|EPS	CE-EPS-UDP-N-Acetyl-D-Mannosamine	CE-EPS-UDP-N Acetyl D Mannosamine Modification	wecC->PGPT0022670	1

			Metabolism			
Indirect Effects	CompetitiveExclusion|CE	CE-Exopolysaccharide Production|EPS	CE-EPS-WZA-WZC-WZB-WEC Metabolism	CE-EPS-EPS-WZA-WZC-WZB-WEC System	wecB->PGPT0018905	2
Indirect Effects	CompetitiveExclusion|CE	CE-Exopolysaccharide Production|EPS	CE-EPS-WZA-WZC-WZB-WEC Metabolism	CE-EPS-EPS-WZA-WZC-WZB-WEC System	wecC->PGPT0022670	1
Indirect Effects	CompetitiveExclusion|CE	CE-Quorum Sensing Response|Biofilm Formation	CE-Biofilm Regulators	CE-BF-Related-AI-2 transport	tqsA|ydgG->PGPT0025496	6
Indirect Effects	CompetitiveExclusion|CE	CE-Quorum Sensing Response|Biofilm Formation	CE-Biofilm Regulators	CE-Other Biofilm Regulators	abrB->PGPT0014790	6
Indirect Effects	CompetitiveExclusion|CE	CE-Quorum Sensing Response|Biofilm Formation	CE-Biofilm Regulators	CE-Other Biofilm Regulators	acgA->PGPT0016250	1
Indirect Effects	CompetitiveExclusion|CE	CE-Quorum Sensing Response|Biofilm Formation	CE-Biofilm Regulators	CE-Other Biofilm Regulators	flgM->PGPT0015530	1
Indirect Effects	CompetitiveExclusion|CE	CE-Quorum Sensing Response|Biofilm Formation	CE-Biofilm Regulators	CE-Other Biofilm Regulators	fliA|sigD|whiG->PGPT0015610	1
Indirect Effects	CompetitiveExclusion|CE	CE-Quorum Sensing Response|Biofilm Formation	CE-Biofilm Regulators	CE-Other Biofilm Regulators	icaR->PGPT0026330	2
Indirect Effects	CompetitiveExclusion|CE	CE-Quorum Sensing Response|Biofilm Formation	CE-Biofilm Regulators	CE-Other Biofilm Regulators	luxS->PGPT0016265	1
Indirect Effects	CompetitiveExclusion|CE	CE-Quorum Sensing Response|Biofilm Formation	CE-Biofilm Regulators	CE-Other Biofilm Regulators	lytR|ypdB|yehT->PGPT0026304	2
Indirect	Competiti	CE-Quorum Sensing	CE-Biofilm Regulators	CE-Other Biofilm	lytS|ypdA|yehU-	1

Effects	veExclusi on|CE	Response|Biofilm Formation		Regulators	>PGPT0026305	
Indirect Effects	Competiti veExclusi on|CE	CE-Quorum Sensing Response|Biofilm Formation	CE-Biofilm Regulators	CE-Other Biofilm Regulators	resE->PGPT0026265	1
Indirect Effects	Competiti veExclusi on|CE	CE-Quorum Sensing Response|Biofilm Formation	CE-Biofilm Regulators	CE-Other Biofilm Regulators	rpoE|sigW->PGPT0014960	10
Indirect Effects	Competiti veExclusi on|CE	CE-Quorum Sensing Response|Biofilm Formation	CE-Biofilm Regulators	CE-Other Biofilm Regulators	sigH|sigG|sigF|sigE->PGPT0014795	8
Indirect Effects	Competiti veExclusi on|CE	CE-Quorum Sensing Response|Biofilm Formation	CE-Biofilm Regulators	CE-Other Biofilm Regulators	sinI->PGPT0026295	2
Indirect Effects	Competiti veExclusi on|CE	CE-Quorum Sensing Response|Biofilm Formation	CE-Biofilm Regulators	CE-Other Biofilm Regulators	sinR->PGPT0014755	2
Indirect Effects	Competiti veExclusi on|CE	CE-Quorum Sensing Response|Biofilm Formation	CE-Biofilm Regulators	CE-Other Biofilm Regulators	spo0A->PGPT0014750	1
Indirect Effects	Competiti veExclusi on|CE	CE-Quorum Sensing Response|Biofilm Formation	CE-Biofilm Regulators	CE-Other Biofilm Regulators	spoT->PGPT0014310	1
Indirect Effects	Competiti veExclusi on|CE	CE-Quorum Sensing Response|Biofilm Formation	CE-Biofilm Regulators	CE-Other Biofilm Regulators	sylA->PGPT0016255	3
Indirect Effects	Competiti veExclusi on|CE	CE-Quorum Sensing Response|Biofilm Formation	CE-Biofilm Regulators	CE-Other Biofilm Regulators	ycbA|glnK->PGPT0000735	2
Indirect Effects	Competiti veExclusi on|CE	CE-Quorum Sensing Response|Biofilm Formation	CE-Biofilm Related Genes	CE-Biofilm-Polyhydroxybutyrate Production	phaJ|croR->PGPT0014741	2

Indirect Effects	CompetitiveExclusion|CE	CE-Quorum Sensing Response|Biofilm Formation	CE-Biofilm Related Genes	CE-Biofilm-Polyhydroxybutyrate Production	phbB|phaB->PGPT0014735	1
Indirect Effects	CompetitiveExclusion|CE	CE-Quorum Sensing Response|Biofilm Formation	CE-Biofilm Related Genes	CE-Biofilm-Polyhydroxybutyrate Production	phbC|phaC->PGPT0014740	1
Indirect Effects	CompetitiveExclusion|CE	CE-Quorum Sensing Response|Biofilm Formation	CE-Biofilm Related Genes	CE-Biofilm-Teichoic Acid Metabolism	wecB->PGPT0018905	2
Indirect Effects	CompetitiveExclusion|CE	CE-Quorum Sensing Response|Biofilm Formation	CE-Biofilm Related Genes	CE-Biofilm-Teichoic Acid Metabolism	wecC->PGPT0022670	1
Indirect Effects	CompetitiveExclusion|CE	CE-Quorum Sensing Response|Biofilm Formation	CE-Environmental QSR|BF Signaling	CE-QSR|BF-Acidic Stress Signaling	frc|yfdW->PGPT0002130	2
Indirect Effects	CompetitiveExclusion|CE	CE-Quorum Sensing Response|Biofilm Formation	CE-Environmental QSR|BF Signaling	CE-QSR|BF-Acidic Stress Signaling	gadA|gadB->PGPT0007630	1
Indirect Effects	CompetitiveExclusion|CE	CE-Quorum Sensing Response|Biofilm Formation	CE-Environmental QSR|BF Signaling	CE-QSR|BF-Acidic Stress Signaling	gsp->PGPT0007755	1
Indirect Effects	CompetitiveExclusion|CE	CE-Quorum Sensing Response|Biofilm Formation	CE-Environmental QSR|BF Signaling	CE-QSR|BF-Acidic Stress Signaling	mdcF->PGPT0001720	2
Indirect Effects	CompetitiveExclusion|CE	CE-Quorum Sensing Response|Biofilm Formation	CE-Environmental QSR|BF Signaling	CE-QSR|BF-Acidic Stress Signaling	speE|SRM|SPEC3|SPSD->PGPT0007750	3
Indirect Effects	CompetitiveExclusion|CE	CE-Quorum Sensing Response|Biofilm Formation	CE-Environmental QSR|BF Signaling	CE-QSR|BF-Carbohydrate Limitation Signaling	clp|crp->PGPT0015075	2
Indirect Effects	CompetitiveExclusi	CE-Quorum Sensing Response|Biofilm	CE-Environmental QSR|BF Signaling	CE-QSR|BF-Carbohydrate	crr->PGPT0014090	1

	on|CE	Formation		Limitation Signaling		
Indirect Effects	CompetitiveExclusion|CE	CE-Quorum Sensing Response|Biofilm Formation	CE-Environmental QSR|BF Signaling	CE-QSR|BF-Cell-To-Cell Signaling	cysE->PGPT0020265	1
Indirect Effects	CompetitiveExclusion|CE	CE-Quorum Sensing Response|Biofilm Formation	CE-Environmental QSR|BF Signaling	CE-QSR|BF-Envelope Stress Signaling	liaR->PGPT0014870	4
Indirect Effects	CompetitiveExclusion|CE	CE-Quorum Sensing Response|Biofilm Formation	CE-Environmental QSR|BF Signaling	CE-QSR|BF-Envelope Stress Signaling	liaS->PGPT0014865	1
Indirect Effects	CompetitiveExclusion|CE	CE-Quorum Sensing Response|Biofilm Formation	CE-Environmental QSR|BF Signaling	CE-QSR|BF-Low Temperature|AcidIfication Signaling	rpoS->PGPT0014685	1
Indirect Effects	CompetitiveExclusion|CE	CE-Quorum Sensing Response|Biofilm Formation	CE-Environmental QSR|BF Signaling	CE-QSR|BF-Oxidative Stress Signaling	gsp->PGPT0007755	1
Indirect Effects	CompetitiveExclusion|CE	CE-Quorum Sensing Response|Biofilm Formation	CE-Environmental QSR|BF Signaling	CE-QSR|BF-Oxidative Stress Signaling	oxyR->PGPT0012965	13
Indirect Effects	CompetitiveExclusion|CE	CE-Quorum Sensing Response|Biofilm Formation	CE-Environmental QSR|BF Signaling	CE-QSR|BF-Oxidative Stress Signaling	paiA->PGPT0007785	10
Indirect Effects	CompetitiveExclusion|CE	CE-Quorum Sensing Response|Biofilm Formation	CE-Environmental QSR|BF Signaling	CE-QSR|BF-Oxidative Stress Signaling	patA1->PGPT0007155	1
Indirect Effects	CompetitiveExclusion|CE	CE-Quorum Sensing Response|Biofilm Formation	CE-Environmental QSR|BF Signaling	CE-QSR|BF-Oxidative Stress Signaling	potA->PGPT0007840	1
Indirect Effects	CompetitiveExclusion|CE	CE-Quorum Sensing Response|Biofilm Formation	CE-Environmental QSR|BF Signaling	CE-QSR|BF-Oxidative Stress Signaling	potB->PGPT0007835	1
Indirect	Competiti	CE-Quorum Sensing	CE-Environmental	CE-QSR|BF-Oxidative	potC->PGPT0007830	1

Effects	veExclusi on|CE	Response|Biofilm Formation	QSR|BF Signaling	Stress Signaling		
Indirect Effects	Competiti veExclusi on|CE	CE-Quorum Sensing Response|Biofilm Formation	CE-Environmental QSR|BF Signaling	CE-QSR|BF-Oxidative Stress Signaling	potD->PGPT0007825	1
Indirect Effects	Competiti veExclusi on|CE	CE-Quorum Sensing Response|Biofilm Formation	CE-Environmental QSR|BF Signaling	CE-QSR|BF-Oxidative Stress Signaling	puuP->PGPT0007795	4
Indirect Effects	Competiti veExclusi on|CE	CE-Quorum Sensing Response|Biofilm Formation	CE-Environmental QSR|BF Signaling	CE-QSR|BF-Oxidative Stress Signaling	speB->PGPT0007775	3
Indirect Effects	Competiti veExclusi on|CE	CE-Quorum Sensing Response|Biofilm Formation	CE-Environmental QSR|BF Signaling	CE-QSR|BF-Oxidative Stress Signaling	speE|SRM|SPEC 3|SPSD- >PGPT0007750	3
Indirect Effects	Competiti veExclusi on|CE	CE-Quorum Sensing Response|Biofilm Formation	CE-Environmental QSR|BF Signaling	CE-QSR|BF-Oxidative Stress Signaling	speG->PGPT0007790	6
Indirect Effects	Competiti veExclusi on|CE	CE-Quorum Sensing Response|Biofilm Formation	CE-Environmental QSR|BF Signaling	CE-QSR|BF-Oxygen Availability Signaling	rpoS->PGPT0014685	1
Indirect Effects	Competiti veExclusi on|CE	CE-Quorum Sensing Response|Biofilm Formation	CE-Other QSR|BF Related Systems	CE-QSR Virulence Regulatory System	paaK->PGPT0026240	1
Indirect Effects	Competiti veExclusi on|CE	CE-Quorum Sensing Response|Biofilm Formation	CE-Other QSR|BF Related Systems	CE-QSR|BF- BarA|UvrY-CSR System	glgA->PGPT0025880	1
Indirect Effects	Competiti veExclusi on|CE	CE-Quorum Sensing Response|Biofilm Formation	CE-Other QSR|BF Related Systems	CE-QSR|BF- BarA|UvrY-CSR System	glgC->PGPT0025885	2
Indirect Effects	Competiti veExclusi on|CE	CE-Quorum Sensing Response|Biofilm Formation	CE-Other QSR|BF Related Systems	CE-QSR|BF- BarA|UvrY-CSR System	glgP->PGPT0018545	1

Indirect Effects	CompetitiveExclusion|CE	CE-Quorum Sensing Response|Biofilm Formation	CE-Other QSR|BF Related Systems	CE-QSR|BF-BarA|UvrY-CSR System	pgaC|icaA->PGPT0023485	3
Indirect Effects	CompetitiveExclusion|CE	CE-Quorum Sensing Response|Biofilm Formation	CE-Other QSR|BF Related Systems	CE-QSR|BF-c-di-GMP Signaling Pathway	acgA->PGPT0016250	1
Indirect Effects	CompetitiveExclusion|CE	CE-Quorum Sensing Response|Biofilm Formation	CE-Other QSR|BF Related Systems	CE-QSR|BF-c-di-GMP Signaling Pathway	mucR->PGPT0026010	3
Indirect Effects	CompetitiveExclusion|CE	CE-Quorum Sensing Response|Biofilm Formation	CE-Other QSR|BF Related Systems	CE-QSR|BF-c-di-GMP Signaling Pathway	pslH->PGPT0026045	1
Indirect Effects	CompetitiveExclusion|CE	CE-Quorum Sensing Response|Biofilm Formation	CE-Other QSR|BF Related Systems	CE-QSR|BF-cAMP|CRP Signaling Pathway	clp|crp->PGPT0015075	2
Indirect Effects	CompetitiveExclusion|CE	CE-Quorum Sensing Response|Biofilm Formation	CE-Other QSR|BF Related Systems	CE-QSR|BF-cAMP|CRP Signaling Pathway	crr->PGPT0014090	1
Indirect Effects	CompetitiveExclusion|CE	CE-Quorum Sensing Response|Biofilm Formation	CE-Other QSR|BF Related Systems	CE-QSR|BF-cAMP|CRP Signaling Pathway	flgM->PGPT0015530	1
Indirect Effects	CompetitiveExclusion|CE	CE-Quorum Sensing Response|Biofilm Formation	CE-Other QSR|BF Related Systems	CE-QSR|BF-cAMP|CRP Signaling Pathway	fliA|sigD|whiG->PGPT0015610	1
Indirect Effects	CompetitiveExclusion|CE	CE-Quorum Sensing Response|Biofilm Formation	CE-Other QSR|BF Related Systems	CE-QSR|BF-cAMP|CRP Signaling Pathway	rpoS->PGPT0014685	1
Indirect Effects	CompetitiveExclusion|CE	CE-Quorum Sensing Response|Biofilm Formation	CE-Other QSR|BF Related Systems	CE-QSR|BF-cAMP|CRP Signaling Pathway	ycgR->PGPT0016210	1
Indirect Effects	CompetitiveExclusi	CE-Quorum Sensing Response|Biofilm	CE-Other QSR|BF Related Systems	CE-QSR|BF-cAMP|VFR Signaling	clp|crp->PGPT0015075	2

	on|CE	Formation		Pathway		
Indirect Effects	CompetitiveExclusion|CE	CE-Quorum Sensing Response|Biofilm Formation	CE-Other QSR|BF Related Systems	CE-QSR|BF-cAMP|VFR Signaling Pathway	cpdA->PGPT0021595	1
Indirect Effects	CompetitiveExclusion|CE	CE-Quorum Sensing Response|Biofilm Formation	CE-QSR-Autoinducer Perception	CE-QSR-3-OH PAME Percipitation|Signaling	wecB->PGPT0018905	2
Indirect Effects	CompetitiveExclusion|CE	CE-Quorum Sensing Response|Biofilm Formation	CE-QSR-Autoinducer Perception	CE-QSR-3-OH PAME Percipitation|Signaling	wecC->PGPT0022670	1
Indirect Effects	CompetitiveExclusion|CE	CE-Quorum Sensing Response|Biofilm Formation	CE-QSR-Autoinducer Perception	CE-QSR-AI-1 LasI Percipitation|Signaling|AHL|NAHL	phzF|yddE->PGPT0012805	2
Indirect Effects	CompetitiveExclusion|CE	CE-Quorum Sensing Response|Biofilm Formation	CE-QSR-Autoinducer Perception	CE-QSR-AI-1 LasI Percipitation|Signaling|AHL|NAHL	trpE|phnA->PGPT0007095	3
Indirect Effects	CompetitiveExclusion|CE	CE-Quorum Sensing Response|Biofilm Formation	CE-QSR-Autoinducer Perception	CE-QSR-AI-1 Percipitation|AHL|NAHL	luxE->PGPT0025325	2
Indirect Effects	CompetitiveExclusion|CE	CE-Quorum Sensing Response|Biofilm Formation	CE-QSR-Autoinducer Perception	CE-QSR-AI-1 RhlI|CepI|SolI Percipitation|Signaling|AHL|NAHL	gadA|gadB->PGPT0007630	1
Indirect Effects	CompetitiveExclusion|CE	CE-Quorum Sensing Response|Biofilm Formation	CE-QSR-Autoinducer Perception	CE-QSR-AI-1 RhlI|CepI|SolI Percipitation|Signaling|AHL|NAHL	phzF|yddE->PGPT0012805	2
Indirect Effects	CompetitiveExclusion|CE	CE-Quorum Sensing Response|Biofilm Formation	CE-QSR-Autoinducer Perception	CE-QSR-AI-1 RhlI|CepI|SolI Percipitation|Signaling|AHL|NAHL	rhlB->PGPT0006835	2
Indirect	Competiti	CE-Quorum Sensing	CE-QSR-Autoinducer	CE-QSR-AI-1	trpE|phnA-	3

Effects	veExclusion|CE	Response|Biofilm Formation	Perception	RhlI|CepI|SolI Percipitation|Signaling|AHL|NAHL	>PGPT0007095	
Indirect Effects	CompetitiveExclusion|CE	CE-Quorum Sensing Response|Biofilm Formation	CE-QSR-Autoinducer Perception	CE-QSR-AI-2 Biosynthesis	luxS->PGPT0016265	1
Indirect Effects	CompetitiveExclusion|CE	CE-Quorum Sensing Response|Biofilm Formation	CE-QSR-Autoinducer Perception	CE-QSR-AI-2 Biosynthesis	metK->PGPT0020000	1
Indirect Effects	CompetitiveExclusion|CE	CE-Quorum Sensing Response|Biofilm Formation	CE-QSR-Autoinducer Perception	CE-QSR-AI-2 Biosynthesis	mtnN|pfs|yadA->PGPT0014320	2
Indirect Effects	CompetitiveExclusion|CE	CE-Quorum Sensing Response|Biofilm Formation	CE-QSR-Autoinducer Perception	CE-QSR-AI-2 Percipitation|Signaling	lsrG->PGPT0025515	2
Indirect Effects	CompetitiveExclusion|CE	CE-Quorum Sensing Response|Biofilm Formation	CE-QSR-Autoinducer Perception	CE-QSR-AI-2 Percipitation|Signaling	lsrR->PGPT0025525	1
Indirect Effects	CompetitiveExclusion|CE	CE-Quorum Sensing Response|Biofilm Formation	CE-QSR-Autoinducer Perception	CE-QSR-AI-2 transport	tqsA|ydgG->PGPT0025496	6
Indirect Effects	CompetitiveExclusion|CE	CE-Quorum Sensing Response|Biofilm Formation	CE-QSR-Autoinducer Perception	CE-QSR-AI-3 Perception|Signaling	pmrA->PGPT0003915	3
Indirect Effects	CompetitiveExclusion|CE	CE-Quorum Sensing Response|Biofilm Formation	CE-QSR-Autoinducer Perception	CE-QSR-AI1|AI-2|CAI-1 Perception|Signaling	aphA->PGPT0025575	4
Indirect Effects	CompetitiveExclusion|CE	CE-Quorum Sensing Response|Biofilm Formation	CE-QSR-Autoinducer Perception	CE-QSR-AI1|AI-2|CAI-1 Perception|Signaling	hfq->PGPT0025560	1
Indirect Effects	CompetitiveExclusi	CE-Quorum Sensing Response|Biofilm	CE-QSR-Autoinducer Perception	CE-QSR-AI transport	cycA|ydgF->PGPT0020615	1

	on|CE	Formation				
Indirect Effects	CompetitiveExclusion|CE	CE-Quorum Sensing Response|Biofilm Formation	CE-QSR-Autoinducer Perception	CE-QSR-ComX Pheromone Percipitation|Signaling	comK->PGPT0025815	1
Indirect Effects	CompetitiveExclusion|CE	CE-Quorum Sensing Response|Biofilm Formation	CE-QSR-Autoinducer Perception	CE-QSR-DSF Biosynthesis	fadD->PGPT0008380	6
Indirect Effects	CompetitiveExclusion|CE	CE-Quorum Sensing Response|Biofilm Formation	CE-QSR-Autoinducer Perception	CE-QSR-DSF Percipitation|Signaling	clp|crp->PGPT0015075	2
Indirect Effects	CompetitiveExclusion|CE	CE-Quorum Sensing Response|Biofilm Formation	CE-QSR-Autoinducer Perception	CE-QSR-GABA Percipitation|Signaling	livF->PGPT0020785	1
Indirect Effects	CompetitiveExclusion|CE	CE-Quorum Sensing Response|Biofilm Formation	CE-QSR-Autoinducer Perception	CE-QSR-GABA Percipitation|Signaling	livG->PGPT0020780	1
Indirect Effects	CompetitiveExclusion|CE	CE-Quorum Sensing Response|Biofilm Formation	CE-QSR-Autoinducer Perception	CE-QSR-GABA Percipitation|Signaling	livH->PGPT0020770	1
Indirect Effects	CompetitiveExclusion|CE	CE-Quorum Sensing Response|Biofilm Formation	CE-QSR-Autoinducer Perception	CE-QSR-GABA Percipitation|Signaling	livK->PGPT0020765	1
Indirect Effects	CompetitiveExclusion|CE	CE-Quorum Sensing Response|Biofilm Formation	CE-QSR-Autoinducer Perception	CE-QSR-GABA Percipitation|Signaling	livM->PGPT0020775	1
Indirect Effects	CompetitiveExclusion|CE	CE-Quorum Sensing Response|Biofilm Formation	CE-QSR-Autoinducer Perception	CE-QSR-HHQ|PQS Percipitation|Signaling	phzF|yddE->PGPT0012805	2
Indirect Effects	CompetitiveExclusion|CE	CE-Quorum Sensing Response|Biofilm Formation	CE-QSR-Autoinducer Perception	CE-QSR-HQQ|PQS Biosynthesis	trpE|phnA->PGPT0007095	3

Indirect Effects	CompetitiveExclusion|CE	CE-Quorum Sensing Response|Biofilm Formation	CE-QSR-Autoinducer Perception	CE-QSR-NOR-|Adrenaline Percipitation|Signaling	pmrA->PGPT0003915	3
Indirect Effects	CompetitiveExclusion|CE	CE-Quorum Sensing Response|Biofilm Formation	CE-QSR-Autoinducer Perception	CE-QSR-PentaPeptide Percipitation|Signaling	degU->PGPT0012750	2
Indirect Effects	CompetitiveExclusion|CE	CE-Quorum Sensing Response|Biofilm Formation	CE-QSR-Autoinducer Perception	CE-QSR-PentaPeptide Percipitation|Signaling	oppA|mppA->PGPT0021015	1
Indirect Effects	CompetitiveExclusion|CE	CE-Quorum Sensing Response|Biofilm Formation	CE-QSR-Autoinducer Perception	CE-QSR-PentaPeptide Percipitation|Signaling	oppB->PGPT0021020	1
Indirect Effects	CompetitiveExclusion|CE	CE-Quorum Sensing Response|Biofilm Formation	CE-QSR-Autoinducer Perception	CE-QSR-PentaPeptide Percipitation|Signaling	oppC->PGPT0021025	1
Indirect Effects	CompetitiveExclusion|CE	CE-Quorum Sensing Response|Biofilm Formation	CE-QSR-Autoinducer Perception	CE-QSR-PentaPeptide Percipitation|Signaling	oppD->PGPT0021030	1
Indirect Effects	CompetitiveExclusion|CE	CE-Quorum Sensing Response|Biofilm Formation	CE-QSR-Autoinducer Perception	CE-QSR-PentaPeptide Percipitation|Signaling	oppF->PGPT0021035	2
Indirect Effects	CompetitiveExclusion|CE	CE-Quorum Sensing Response|Biofilm Formation	CE-QSR-Autoinducer Perception	CE-QSR-PentaPeptide Percipitation|Signaling	spo0A->PGPT0014750	1
Indirect Effects	CompetitiveExclusion|CE	CE-Quorum Sensing Response|Biofilm Formation	CE-QSR-Autoinducer Perception	CE-QSR-PentaPeptide Percipitation|Signaling	spo0B->PGPT0024635	1
Indirect Effects	CompetitiveExclusion|CE	CE-Quorum Sensing Response|Biofilm Formation	CE-QSR-Autoinducer Perception	CE-QSR-PentaPeptide Percipitation|Signaling	spo0F->PGPT0024650	1
Indirect	Competiti	CE-Quorum Sensing	CE-QSR-Autoinducer	CE-QSR-PentaPeptide	spoVT->PGPT0024790	1

Effects	veExclusion|CE	Response|Biofilm Formation	Perception	Percipitation|Signaling		
Indirect Effects	CompetitiveExclusion|CE	CE-Quorum Sensing Response|Biofilm Formation	CE-QSR-Autoinducer Perception	CE-QSR-PentaPeptide transport	ffh->PGPT0025750	1
Indirect Effects	CompetitiveExclusion|CE	CE-Quorum Sensing Response|Biofilm Formation	CE-QSR-Autoinducer Perception	CE-QSR-PentaPeptide transport	ftsY->PGPT0025740	1
Indirect Effects	CompetitiveExclusion|CE	CE-Quorum Sensing Response|Biofilm Formation	CE-QSR-Autoinducer Perception	CE-QSR-PentaPeptide transport	secA->PGPT0025735	1
Indirect Effects	CompetitiveExclusion|CE	CE-Quorum Sensing Response|Biofilm Formation	CE-QSR-Autoinducer Perception	CE-QSR-PentaPeptide transport	secDF->PGPT0025710	1
Indirect Effects	CompetitiveExclusion|CE	CE-Quorum Sensing Response|Biofilm Formation	CE-QSR-Autoinducer Perception	CE-QSR-PentaPeptide transport	secE->PGPT0025715	1
Indirect Effects	CompetitiveExclusion|CE	CE-Quorum Sensing Response|Biofilm Formation	CE-QSR-Autoinducer Perception	CE-QSR-PentaPeptide transport	secG->PGPT0025720	1
Indirect Effects	CompetitiveExclusion|CE	CE-Quorum Sensing Response|Biofilm Formation	CE-QSR-Autoinducer Perception	CE-QSR-PentaPeptide transport	secY->PGPT0025725	1
Indirect Effects	CompetitiveExclusion|CE	CE-Quorum Sensing Response|Biofilm Formation	CE-QSR-Autoinducer Perception	CE-QSR-PentaPeptide transport	yajC->PGPT0025730	1
Indirect Effects	CompetitiveExclusion|CE	CE-Quorum Sensing Response|Biofilm Formation	CE-QSR-Autoinducer Perception	CE-QSR-PentaPeptide transport	yidC|spoIIIJ|oxaA|ccfA->PGPT0024530	3
Indirect Effects	CompetitiveExclusion|CE	CE-Quorum Sensing Response|Biofilm Formation	CE-QSR-Autoinducer Perception	CE-QSR-Unknown AI Percipitation-DPP-Mediated Pathway	ddpA|ABC PE S->PGPT0004430	6

Indirect Effects	CompetitiveExclusion|CE	CE-Quorum Sensing Response|Biofilm Formation	CE-QSR-Autoinducer Perception	CE-QSR-Unknown AI Percipitation-DPP-Mediated Pathway	ddpB|appB->PGPT0004445	4
Indirect Effects	CompetitiveExclusion|CE	CE-Quorum Sensing Response|Biofilm Formation	CE-QSR-Autoinducer Perception	CE-QSR-Unknown AI Percipitation-DPP-Mediated Pathway	ddpC|appC->PGPT0004450	4
Indirect Effects	CompetitiveExclusion|CE	CE-Quorum Sensing Response|Biofilm Formation	CE-QSR-Autoinducer Perception	CE-QSR-Unknown AI Percipitation-DPP-Mediated Pathway	ddpD->PGPT0004435	4
Indirect Effects	CompetitiveExclusion|CE	CE-Quorum Sensing Response|Biofilm Formation	CE-QSR-Autoinducer Perception	CE-QSR-Unknown AI Percipitation-DPP-Mediated Pathway	ddpF->PGPT0004440	4
Indirect Effects	CompetitiveExclusion|CE	CE-Quorum Sensing Response|Biofilm Formation	CE-QSR|BF-Cell Density Regulation	CE-QSR|BF-Biofilm-Low Cell Density Regulator	aphA->PGPT0025575	4
Indirect Effects	CompetitiveExclusion|CE	CE-Quorum Sensing Response|Biofilm Formation	CE-Quorum Quenching	CE-Quorum Quenching-AHL-Degradation	cpxB->PGPT0006740	1
Indirect Effects	CompetitiveExclusion|CE	CE-Quorum Sensing Response|Biofilm Formation	CE-Quorum Sensing Regulators	CE-QSR-Other Quorum Sensing Regulated Genes	nprR|nprA->PGPT0011700	3
Indirect Effects	CompetitiveExclusion|CE	CE-Quorum Sensing Response|Biofilm Formation	CE-Quorum Sensing Regulators	CE-QSR-Quorum Sensing Regulated FLS	flgM->PGPT0015530	1
Indirect Effects	CompetitiveExclusion|CE	CE-Quorum Sensing Response|Biofilm Formation	CE-Quorum Sensing Regulators	CE-QSR-Quorum Sensing Regulated FLS	fliA|sigD|whiG->PGPT0015610	1
Indirect Effects	CompetitiveExclusion|CE	CE-Quorum Sensing Response|Biofilm Formation	CE-Quorum Sensing Related Genes	CE-QSR-Cardiolipin Synthesis	clsA B|ybhO|ywiE->PGPT0007725	5
Indirect Effects	CompetitiveExclusi	CE-Quorum Sensing Response|Biofilm	CE-Quorum Sensing Related Genes	CE-QSR-Putative transporter	rhtB->PGPT0021036	4

	on|CE	Formation				
Indirect Effects	CompetitiveExclusion|CE	CE-Spore Production	CE-Spore Formation|Germination	CE-Other Sporulation Related Proteins	tgl->PGPT0024948	1
Indirect Effects	CompetitiveExclusion|CE	CE-Spore Production	CE-Spore Formation|Germination	CE-Other Sporulation Related Proteins	yabP->PGPT0024990	1
Indirect Effects	CompetitiveExclusion|CE	CE-Spore Production	CE-Spore Formation|Germination	CE-Other Sporulation Related Proteins	yhbH->PGPT0025005	1
Indirect Effects	CompetitiveExclusion|CE	CE-Spore Production	CE-Spore Formation|Germination	CE-Other Sporulation Related Proteins	ytfJ->PGPT0024995	1
Indirect Effects	CompetitiveExclusion|CE	CE-Spore Production	CE-Spore Formation|Germination	CE-Other Sporulation Related Proteins	yunB->PGPT0025000	1
Indirect Effects	CompetitiveExclusion|CE	CE-Spore Production	CE-Spore Formation|Germination	CE-Small Acid-Soluble Spore Proteins	SASP A|sspA->PGPT0024860	13
Indirect Effects	CompetitiveExclusion|CE	CE-Spore Production	CE-Spore Formation|Germination	CE-Small Acid-Soluble Spore Proteins	SASP B|sspB->PGPT0024865	2
Indirect Effects	CompetitiveExclusion|CE	CE-Spore Production	CE-Spore Formation|Germination	CE-Small Acid-Soluble Spore Proteins	sspD->PGPT0024875	1
Indirect Effects	CompetitiveExclusion|CE	CE-Spore Production	CE-Spore Formation|Germination	CE-Small Acid-Soluble Spore Proteins	sspE->PGPT0024880	1
Indirect Effects	CompetitiveExclusion|CE	CE-Spore Production	CE-Spore Formation|Germination	CE-Small Acid-Soluble Spore Proteins	sspH->PGPT0024895	4
Indirect	Competiti	CE-Spore Production	CE-Spore	CE-Small Acid-Soluble	sspI->PGPT0024900	1

Effects	veExclusion|CE		Formation|Germination	Spore Proteins		
Indirect Effects	CompetitiveExclusion|CE	CE-Spore Production	CE-Spore Formation|Germination	CE-Small Acid-Soluble Spore Proteins	sspK->PGPT0024910	1
Indirect Effects	CompetitiveExclusion|CE	CE-Spore Production	CE-Spore Formation|Germination	CE-Small Acid-Soluble Spore Proteins	sspN->PGPT0024925	1
Indirect Effects	CompetitiveExclusion|CE	CE-Spore Production	CE-Spore Formation|Germination	CE-Small Acid-Soluble Spore Proteins	sspO|cotK->PGPT0024930	1
Indirect Effects	CompetitiveExclusion|CE	CE-Spore Production	CE-Spore Formation|Germination	CE-Small Acid-Soluble Spore Proteins	sspP|cotL->PGPT0024935	1
Indirect Effects	CompetitiveExclusion|CE	CE-Spore Production	CE-Spore Formation|Germination	CE-Small Acid-Soluble Spore Proteins	tlp->PGPT0024940	1
Indirect Effects	CompetitiveExclusion|CE	CE-Spore Production	CE-Spore Formation|Germination	CE-Spore Coat Protein	cotD->PGPT0025150	2
Indirect Effects	CompetitiveExclusion|CE	CE-Spore Production	CE-Spore Formation|Germination	CE-Spore Coat Protein	cotE->PGPT0025155	1
Indirect Effects	CompetitiveExclusion|CE	CE-Spore Production	CE-Spore Formation|Germination	CE-Spore Coat Protein	cotF->PGPT0025160	1
Indirect Effects	CompetitiveExclusion|CE	CE-Spore Production	CE-Spore Formation|Germination	CE-Spore Coat Protein	cotH->PGPT0025165	1
Indirect Effects	CompetitiveExclusion|CE	CE-Spore Production	CE-Spore Formation|Germination	CE-Spore Coat Protein	cotJA->PGPT0025175	1

Indirect Effects	CompetitiveExclusion|CE	CE-Spore Production	CE-Spore Formation|Germination	CE-Spore Coat Protein	cotJB->PGPT0025180	1
Indirect Effects	CompetitiveExclusion|CE	CE-Spore Production	CE-Spore Formation|Germination	CE-Spore Coat Protein	cotJC->PGPT0025185	1
Indirect Effects	CompetitiveExclusion|CE	CE-Spore Production	CE-Spore Formation|Germination	CE-Spore Coat Protein	cotX->PGPT0025225	2
Indirect Effects	CompetitiveExclusion|CE	CE-Spore Production	CE-Spore Formation|Germination	CE-Spore Coat Protein	spsD->PGPT0025264	2
Indirect Effects	CompetitiveExclusion|CE	CE-Spore Production	CE-Spore Formation|Germination	CE-Spore Coat Protein	yabG->PGPT0025240	1
Indirect Effects	CompetitiveExclusion|CE	CE-Spore Production	CE-Spore Formation|Germination	CE-Spore Coat Protein	yraD->PGPT0025245	3
Indirect Effects	CompetitiveExclusion|CE	CE-Spore Production	CE-Spore Formation|Germination	CE-Spore Germination Protein	gerAA->PGPT0025010	1
Indirect Effects	CompetitiveExclusion|CE	CE-Spore Production	CE-Spore Formation|Germination	CE-Spore Germination Protein	gerAB->PGPT0025015	2
Indirect Effects	CompetitiveExclusion|CE	CE-Spore Production	CE-Spore Formation|Germination	CE-Spore Germination Protein	gerAC->PGPT0025020	1
Indirect Effects	CompetitiveExclusion|CE	CE-Spore Production	CE-Spore Formation|Germination	CE-Spore Germination Protein	gerC->PGPT0025040	1
Indirect Effects	CompetitiveExclusi	CE-Spore Production	CE-Spore Formation|Germinatio	CE-Spore Germination Protein	gerD->PGPT0025045	1

	on|CE		n			
Indirect Effects	CompetitiveExclusion|CE	CE-Spore Production	CE-Spore Formation|Germination	CE-Spore Germination Protein	gerKA->PGPT0025050	5
Indirect Effects	CompetitiveExclusion|CE	CE-Spore Production	CE-Spore Formation|Germination	CE-Spore Germination Protein	gerKB->PGPT0025055	10
Indirect Effects	CompetitiveExclusion|CE	CE-Spore Production	CE-Spore Formation|Germination	CE-Spore Germination Protein	gerKC->PGPT0025060	3
Indirect Effects	CompetitiveExclusion|CE	CE-Spore Production	CE-Spore Formation|Germination	CE-Spore Germination Protein	gerPA->PGPT0025065	1
Indirect Effects	CompetitiveExclusion|CE	CE-Spore Production	CE-Spore Formation|Germination	CE-Spore Germination Protein	gerPB->PGPT0025070	1
Indirect Effects	CompetitiveExclusion|CE	CE-Spore Production	CE-Spore Formation|Germination	CE-Spore Germination Protein	gerPC->PGPT0025075	1
Indirect Effects	CompetitiveExclusion|CE	CE-Spore Production	CE-Spore Formation|Germination	CE-Spore Germination Protein	gerPE->PGPT0025085	1
Indirect Effects	CompetitiveExclusion|CE	CE-Spore Production	CE-Spore Formation|Germination	CE-Spore Germination Protein	gerPF->PGPT0025090	4
Indirect Effects	CompetitiveExclusion|CE	CE-Spore Production	CE-Spore Formation|Germination	CE-Spore Germination Protein	gerQ->PGPT0025095	1
Indirect Effects	CompetitiveExclusion|CE	CE-Spore Production	CE-Spore Formation|Germination	CE-Spore Germination Protein	gerXB->PGPT0025105	1
Indirect	Competiti	CE-Spore Production	CE-Spore	CE-Spore Germination	yaaH->PGPT0025110	3

Effects	veExclusion|CE		Formation|Germination	Protein		
Indirect Effects	CompetitiveExclusion|CE	CE-Spore Production	CE-Spore Formation|Germination	CE-Spore Germination Protein	yndD->PGPT0025115	2
Indirect Effects	CompetitiveExclusion|CE	CE-Spore Production	CE-Spore Formation|Germination	CE-Spore Germination Protein	yndE->PGPT0025120	2
Indirect Effects	CompetitiveExclusion|CE	CE-Spore Production	CE-Spore Formation|Germination	CE-Spore Germination Protein	yndF->PGPT0025125	2
Indirect Effects	CompetitiveExclusion|CE	CE-Spore Production	CE-Spore Formation|Germination	CE-Spore Germination Protein	ypeB->PGPT0025130	1
Indirect Effects	CompetitiveExclusion|CE	CE-Spore Production	CE-Spore Formation|Germination	CE-Sporulation Regulation	gerE->PGPT0025270	1
Indirect Effects	CompetitiveExclusion|CE	CE-Spore Production	CE-Spore Formation|Germination	CE-Sporulation Regulation	kapB->PGPT0025295	1
Indirect Effects	CompetitiveExclusion|CE	CE-Spore Production	CE-Spore Formation|Germination	CE-Sporulation Regulation	kapD->PGPT0025294	1
Indirect Effects	CompetitiveExclusion|CE	CE-Spore Production	CE-Spore Formation|Germination	CE-Sporulation Regulation	kinA->PGPT0025280	5
Indirect Effects	CompetitiveExclusion|CE	CE-Spore Production	CE-Spore Formation|Germination	CE-Sporulation Regulation	kinB->PGPT0025285	3
Indirect Effects	CompetitiveExclusion|CE	CE-Spore Production	CE-Spore Formation|Germination	CE-Sporulation Regulation	kinC->PGPT0025286	1

Indirect Effects	CompetitiveExclusion|CE	CE-Spore Production	CE-Spore Formation|Germination	CE-Sporulation Regulation	kinE->PGPT0025290	3
Indirect Effects	CompetitiveExclusion|CE	CE-Spore Production	CE-Spore Formation|Germination	CE-Sporulation Regulation	sigH|sigG|sigF|sigE->PGPT0014795	8
Indirect Effects	CompetitiveExclusion|CE	CE-Spore Production	CE-Spore Formation|Germination	CE-Stage 0 Sporulation	spo0A->PGPT0014750	1
Indirect Effects	CompetitiveExclusion|CE	CE-Spore Production	CE-Spore Formation|Germination	CE-Stage 0 Sporulation	spo0B->PGPT0024635	1
Indirect Effects	CompetitiveExclusion|CE	CE-Spore Production	CE-Spore Formation|Germination	CE-Stage 0 Sporulation	spo0E->PGPT0024640	8
Indirect Effects	CompetitiveExclusion|CE	CE-Spore Production	CE-Spore Formation|Germination	CE-Stage 0 Sporulation	spo0F->PGPT0024650	1
Indirect Effects	CompetitiveExclusion|CE	CE-Spore Production	CE-Spore Formation|Germination	CE-Stage 0 Sporulation	spo0M->PGPT0024630	2
Indirect Effects	CompetitiveExclusion|CE	CE-Spore Production	CE-Spore Formation|Germination	CE-Stage III Sporulation	spoIIIAA->PGPT0024720	1
Indirect Effects	CompetitiveExclusion|CE	CE-Spore Production	CE-Spore Formation|Germination	CE-Stage III Sporulation	spoIIIAB->PGPT0024725	1
Indirect Effects	CompetitiveExclusion|CE	CE-Spore Production	CE-Spore Formation|Germination	CE-Stage III Sporulation	spoIIIAC->PGPT0024730	1
Indirect Effects	CompetitiveExclusi	CE-Spore Production	CE-Spore Formation|Germinatio	CE-Stage III Sporulation	spoIIIAD->PGPT0024735	1

	on|CE		n			
Indirect Effects	CompetitiveExclusion|CE	CE-Spore Production	CE-Spore Formation|Germination	CE-Stage III Sporulation	spoIIIAE->PGPT0024740	1
Indirect Effects	CompetitiveExclusion|CE	CE-Spore Production	CE-Spore Formation|Germination	CE-Stage III Sporulation	spoIIIAF->PGPT0024745	1
Indirect Effects	CompetitiveExclusion|CE	CE-Spore Production	CE-Spore Formation|Germination	CE-Stage III Sporulation	spoIIIAH->PGPT0024755	1
Indirect Effects	CompetitiveExclusion|CE	CE-Spore Production	CE-Spore Formation|Germination	CE-Stage III Sporulation	spoIIID->PGPT0024715	1
Indirect Effects	CompetitiveExclusion|CE	CE-Spore Production	CE-Spore Formation|Germination	CE-Stage II Sporulation	spoIIAA->PGPT0024655	1
Indirect Effects	CompetitiveExclusion|CE	CE-Spore Production	CE-Spore Formation|Germination	CE-Stage II Sporulation	spoIIAB->PGPT0024660	2
Indirect Effects	CompetitiveExclusion|CE	CE-Spore Production	CE-Spore Formation|Germination	CE-Stage II Sporulation	spoIIB->PGPT0024665	1
Indirect Effects	CompetitiveExclusion|CE	CE-Spore Production	CE-Spore Formation|Germination	CE-Stage II Sporulation	spoIID->PGPT0024670	1
Indirect Effects	CompetitiveExclusion|CE	CE-Spore Production	CE-Spore Formation|Germination	CE-Stage II Sporulation	spoIIE->PGPT0024675	1
Indirect Effects	CompetitiveExclusion|CE	CE-Spore Production	CE-Spore Formation|Germination	CE-Stage II Sporulation	spoIIGA->PGPT0024680	1
Indirect	Competiti	CE-Spore Production	CE-Spore	CE-Stage II	spoIIM->PGPT0024685	1

Effects	veExclusion|CE		Formation|Germination	Sporulation		
Indirect Effects	CompetitiveExclusion|CE	CE-Spore Production	CE-Spore Formation|Germination	CE-Stage II Sporulation	spoIIP->PGPT0024690	2
Indirect Effects	CompetitiveExclusion|CE	CE-Spore Production	CE-Spore Formation|Germination	CE-Stage II Sporulation	spoIIQ->PGPT0024695	1
Indirect Effects	CompetitiveExclusion|CE	CE-Spore Production	CE-Spore Formation|Germination	CE-Stage II Sporulation	spoIIR->PGPT0024700	1
Indirect Effects	CompetitiveExclusion|CE	CE-Spore Production	CE-Spore Formation|Germination	CE-Stage IV Sporulation	spoIVA->PGPT0024770	1
Indirect Effects	CompetitiveExclusion|CE	CE-Spore Production	CE-Spore Formation|Germination	CE-Stage IV Sporulation	spoIVB->PGPT0024775	1
Indirect Effects	CompetitiveExclusion|CE	CE-Spore Production	CE-Spore Formation|Germination	CE-Stage IV Sporulation	spoIVFA->PGPT0024780	1
Indirect Effects	CompetitiveExclusion|CE	CE-Spore Production	CE-Spore Formation|Germination	CE-Stage IV Sporulation	spoIVFB->PGPT0024785	1
Indirect Effects	CompetitiveExclusion|CE	CE-Spore Production	CE-Spore Formation|Germination	CE-Stage IV Sporulation	yqfC->PGPT0024765	1
Indirect Effects	CompetitiveExclusion|CE	CE-Spore Production	CE-Spore Formation|Germination	CE-Stage IV Sporulation	yqfD->PGPT0024760	1
Indirect Effects	CompetitiveExclusion|CE	CE-Spore Production	CE-Spore Formation|Germination	CE-Stage VI Sporulation	spoVID->PGPT0024855	1

Indirect Effects	CompetitiveExclusion|CE	CE-Spore Production	CE-Spore Formation|Germination	CE-Stage V Sporulation	spoVAA->PGPT0024795	1
Indirect Effects	CompetitiveExclusion|CE	CE-Spore Production	CE-Spore Formation|Germination	CE-Stage V Sporulation	spoVAB->PGPT0024800	1
Indirect Effects	CompetitiveExclusion|CE	CE-Spore Production	CE-Spore Formation|Germination	CE-Stage V Sporulation	spoVAC->PGPT0024805	1
Indirect Effects	CompetitiveExclusion|CE	CE-Spore Production	CE-Spore Formation|Germination	CE-Stage V Sporulation	spoVAD->PGPT0024810	1
Indirect Effects	CompetitiveExclusion|CE	CE-Spore Production	CE-Spore Formation|Germination	CE-Stage V Sporulation	spoVAE->PGPT0024815	1
Indirect Effects	CompetitiveExclusion|CE	CE-Spore Production	CE-Spore Formation|Germination	CE-Stage V Sporulation	spoVAF->PGPT0024820	1
Indirect Effects	CompetitiveExclusion|CE	CE-Spore Production	CE-Spore Formation|Germination	CE-Stage V Sporulation	spoVB->PGPT0024825	2
Indirect Effects	CompetitiveExclusion|CE	CE-Spore Production	CE-Spore Formation|Germination	CE-Stage V Sporulation	spoVD->PGPT0024085	1
Indirect Effects	CompetitiveExclusion|CE	CE-Spore Production	CE-Spore Formation|Germination	CE-Stage V Sporulation	spoVG->PGPT0024830	1
Indirect Effects	CompetitiveExclusion|CE	CE-Spore Production	CE-Spore Formation|Germination	CE-Stage V Sporulation	spoVK->PGPT0024835	1
Indirect Effects	CompetitiveExclusi	CE-Spore Production	CE-Spore Formation|Germinatio	CE-Stage V Sporulation	spoVR->PGPT0024845	1

	on|CE		n			
Indirect Effects	CompetitiveExclusion|CE	CE-Spore Production	CE-Spore Formation|Germination	CE-Stage V Sporulation	spoVS->PGPT0024850	1
Indirect Effects	CompetitiveExclusion|CE	CE-Spore Production	CE-Spore Formation|Germination	CE-Stage V Sporulation	spoVT->PGPT0024790	1
Indirect Effects	PlantImmune Response Stimulation	Induction Of Systemic Resistance|ISR	ISR-Vitamin B1|Thiamine Metabolism	ISR-Vitamin B1|Thiamine Biosynthesis	adk|AK->PGPT0009040	1
Indirect Effects	PlantImmune Response Stimulation	Induction Of Systemic Resistance|ISR	ISR-Vitamin B1|Thiamine Metabolism	ISR-Vitamin B1|Thiamine Biosynthesis	dxs->PGPT0008960	1
Indirect Effects	PlantImmune Response Stimulation	Induction Of Systemic Resistance|ISR	ISR-Vitamin B1|Thiamine Metabolism	ISR-Vitamin B1|Thiamine Biosynthesis	nifS|iscS->PGPT0000065	4
Indirect Effects	PlantImmune Response Stimulation	Induction Of Systemic Resistance|ISR	ISR-Vitamin B1|Thiamine Metabolism	ISR-Vitamin B1|Thiamine Biosynthesis	phoA->PGPT0002570	2
Indirect Effects	PlantImmune Response Stimulation	Induction Of Systemic Resistance|ISR	ISR-Vitamin B1|Thiamine Metabolism	ISR-Vitamin B1|Thiamine Biosynthesis	rsgA|engC->PGPT0009020	1

Indirect Effects	PlantImmune Response Stimulation	Induction Of Systemic Resistance|ISR	ISR-Vitamin B1|Thiamine Metabolism	ISR-Vitamin B1|Thiamine Biosynthesis	tenA->PGPT0009055	2
Indirect Effects	PlantImmune Response Stimulation	Induction Of Systemic Resistance|ISR	ISR-Vitamin B1|Thiamine Metabolism	ISR-Vitamin B1|Thiamine Biosynthesis	tenI->PGPT0008975	1
Indirect Effects	PlantImmune Response Stimulation	Induction Of Systemic Resistance|ISR	ISR-Vitamin B1|Thiamine Metabolism	ISR-Vitamin B1|Thiamine Biosynthesis	thiC->PGPT0008905	1
Indirect Effects	PlantImmune Response Stimulation	Induction Of Systemic Resistance|ISR	ISR-Vitamin B1|Thiamine Metabolism	ISR-Vitamin B1|Thiamine Biosynthesis	thiD->PGPT0008915	1
Indirect Effects	PlantImmune Response Stimulation	Induction Of Systemic Resistance|ISR	ISR-Vitamin B1|Thiamine Metabolism	ISR-Vitamin B1|Thiamine Biosynthesis	thiE->PGPT0008995	1
Indirect Effects	PlantImmune Response Stimulation	Induction Of Systemic Resistance|ISR	ISR-Vitamin B1|Thiamine Metabolism	ISR-Vitamin B1|Thiamine Biosynthesis	thiF->PGPT0008935	2
Indirect Effects	PlantImmune	Induction Of Systemic Resistance|ISR	ISR-Vitamin B1|Thiamine	ISR-Vitamin B1|Thiamine	thiG->PGPT0008965	1

	Response Stimulation		Metabolism	Biosynthesis		
Indirect Effects	PlantImmune Response Stimulation	Induction Of Systemic Resistance|ISR	ISR-Vitamin B1|Thiamine Metabolism	ISR-Vitamin B1|Thiamine Biosynthesis	thiI->PGPT0008940	1
Indirect Effects	PlantImmune Response Stimulation	Induction Of Systemic Resistance|ISR	ISR-Vitamin B1|Thiamine Metabolism	ISR-Vitamin B1|Thiamine Biosynthesis	thiM->PGPT0008990	1
Indirect Effects	PlantImmune Response Stimulation	Induction Of Systemic Resistance|ISR	ISR-Vitamin B1|Thiamine Metabolism	ISR-Vitamin B1|Thiamine Biosynthesis	thiN|TPK1|THI80->PGPT0009035	1
Indirect Effects	PlantImmune Response Stimulation	Induction Of Systemic Resistance|ISR	ISR-Vitamin B1|Thiamine Metabolism	ISR-Vitamin B1|Thiamine Biosynthesis	thiO->PGPT0008955	2
Indirect Effects	PlantImmune Response Stimulation	Induction Of Systemic Resistance|ISR	ISR-Vitamin B1|Thiamine Metabolism	ISR-Vitamin B1|Thiamine Biosynthesis	ylmB->PGPT0009060	1
Indirect Effects	PlantImmune Response Stimulatio	Induction Of Systemic Resistance|ISR	ISR-Vitamin B1|Thiamine Metabolism	ISR-Vitamin B1|Thiamine transport	cytX->PGPT0009075	3

	n					
Indirect Effects	PlantImmune Response Stimulation	Induction Of Systemic Resistance|ISR	ISR-Vitamin B1|Thiamine Metabolism	ISR-Vitamin B1|Thiamine transport	thiT->PGPT0009100	1
Indirect Effects	PlantImmune Response Stimulation	Induction Of Systemic Resistance|ISR	ISR-Vitamin B2|Riboflavin Metabolism	ISR-Vitamin B2|Riboflavin Biosynthesis	RFK|FMN1->PGPT0008615	1
Indirect Effects	PlantImmune Response Stimulation	Induction Of Systemic Resistance|ISR	ISR-Vitamin B2|Riboflavin Metabolism	ISR-Vitamin B2|Riboflavin Biosynthesis	bluB|drgA->PGPT0006810	1
Indirect Effects	PlantImmune Response Stimulation	Induction Of Systemic Resistance|ISR	ISR-Vitamin B2|Riboflavin Metabolism	ISR-Vitamin B2|Riboflavin Biosynthesis	nfrA1|ywcG->PGPT0000290	1
Indirect Effects	PlantImmune Response Stimulation	Induction Of Systemic Resistance|ISR	ISR-Vitamin B2|Riboflavin Metabolism	ISR-Vitamin B2|Riboflavin Biosynthesis	nfrA2|ycnD->PGPT0000295	1
Indirect Effects	PlantImmune Response Stimulation	Induction Of Systemic Resistance|ISR	ISR-Vitamin B2|Riboflavin Metabolism	ISR-Vitamin B2|Riboflavin Biosynthesis	ribA->PGPT0007985	1
Indirect	PlantImm	Induction Of Systemic	ISR-Vitamin	ISR-Vitamin	ribBA->PGPT0007990	1

Effects	une Response Stimulation	Resistance|ISR	B2|Riboflavin Metabolism	B2|Riboflavin Biosynthesis		
Indirect Effects	PlantImmune Response Stimulation	Induction Of Systemic Resistance|ISR	ISR-Vitamin B2|Riboflavin Metabolism	ISR-Vitamin B2|Riboflavin Biosynthesis	ribD->PGPT0008555	2
Indirect Effects	PlantImmune Response Stimulation	Induction Of Systemic Resistance|ISR	ISR-Vitamin B2|Riboflavin Metabolism	ISR-Vitamin B2|Riboflavin Biosynthesis	ribE|RIB5|ribC->PGPT0008610	1
Indirect Effects	PlantImmune Response Stimulation	Induction Of Systemic Resistance|ISR	ISR-Vitamin B2|Riboflavin Metabolism	ISR-Vitamin B2|Riboflavin Biosynthesis	ribF->PGPT0008625	1
Indirect Effects	PlantImmune Response Stimulation	Induction Of Systemic Resistance|ISR	ISR-Vitamin B2|Riboflavin Metabolism	ISR-Vitamin B2|Riboflavin Biosynthesis	ribH|RIB4->PGPT0008605	1
Indirect Effects	PlantImmune Response Stimulation	Induction Of Systemic Resistance|ISR	ISR-Vitamin B2|Riboflavin Metabolism	ISR-Vitamin B2|Riboflavin Biosynthesis	ssuE->PGPT0003045	1
Indirect Effects	PlantImmune Response	Induction Of Systemic Resistance|ISR	ISR-Vitamin B2|Riboflavin Metabolism	ISR-Vitamin B2|Riboflavin Biosynthesis	ycsE|yitU|ywtE->PGPT0008595	5

	Stimulation					
Indirect Effects	PlantImmune Response Stimulation	Induction Of Systemic Resistance|ISR	ISR-Vitamin B2|Riboflavin Metabolism	ISR-Vitamin B2|Riboflavin Biosynthesis	yigB->PGPT0008585	1
Indirect Effects	PlantImmune Response Stimulation	Induction Of Systemic Resistance|ISR	ISR-Volatiles	ISR-Acetoin|2|3-Butanediol Biosynthesis	aceF|pdhC->PGPT0001390	2
Indirect Effects	PlantImmune Response Stimulation	Induction Of Systemic Resistance|ISR	ISR-Volatiles	ISR-Acetoin|2|3-Butanediol Biosynthesis	acoA->PGPT0008230	1
Indirect Effects	PlantImmune Response Stimulation	Induction Of Systemic Resistance|ISR	ISR-Volatiles	ISR-Acetoin|2|3-Butanediol Biosynthesis	acoB->PGPT0008235	1
Indirect Effects	PlantImmune Response Stimulation	Induction Of Systemic Resistance|ISR	ISR-Volatiles	ISR-Acetoin|2|3-Butanediol Biosynthesis	acoR->PGPT0001030	6
Indirect Effects	PlantImmune Response Stimulation	Induction Of Systemic Resistance|ISR	ISR-Volatiles	ISR-Acetoin|2|3-Butanediol Biosynthesis	acuB->PGPT0008220	4

Indirect Effects	PlantImmune Response Stimulation	Induction Of Systemic Resistance|ISR	ISR-Volatiles	ISR-Acetoin|2|3-Butanediol Biosynthesis	acuC->PGPT0008225	1
Indirect Effects	PlantImmune Response Stimulation	Induction Of Systemic Resistance|ISR	ISR-Volatiles	ISR-Acetoin|2|3-Butanediol Biosynthesis	budA|aldC|aldB|alsD->PGPT0008180	1
Indirect Effects	PlantImmune Response Stimulation	Induction Of Systemic Resistance|ISR	ISR-Volatiles	ISR-Acetoin|2|3-Butanediol Biosynthesis	budB|ilvK|alsS|ilvB|ilvG|ilvI->PGPT0008185	4
Indirect Effects	PlantImmune Response Stimulation	Induction Of Systemic Resistance|ISR	ISR-Volatiles	ISR-Acetoin|2|3-Butanediol Biosynthesis	budC->PGPT0008190	3
Indirect Effects	PlantImmune Response Stimulation	Induction Of Systemic Resistance|ISR	ISR-Volatiles	ISR-Acetoin|2|3-Butanediol Biosynthesis	butA|ydjL|budC->PGPT0008195	1
Indirect Effects	PlantImmune Response Stimulation	Induction Of Systemic Resistance|ISR	ISR-Volatiles	ISR-Acetoin|2|3-Butanediol Biosynthesis	butB->PGPT0008200	1
Indirect Effects	PlantImmune	Induction Of Systemic Resistance|ISR	ISR-Volatiles	ISR-Acetoin|2|3-Butanediol	ilvH|ilvN->PGPT0008205	1

	Response Stimulation			Biosynthesis		
Indirect Effects	PlantImmune Response Stimulation	Induction Of Systemic Resistance|ISR	ISR-Volatiles	ISR-Acetoin|2|3-Butanediol Biosynthesis	lpd|pdhD->PGPT0001380	3
Indirect Effects	PlantImmune Response Stimulation	Triggered Immunity	PAMP|Effector Triggered Immunity|PTI	PTI-Bacterial EF-TU	elf18|tuf|tufA->PGPT0015245	1
Indirect Effects	PlantImmune Response Stimulation	Triggered Immunity	PAMP|Effector Triggered Immunity|PTI	PTI-Flagellin Triggered Immunity	flgK->PGPT0015195	1
Indirect Effects	PlantImmune Response Stimulation	Triggered Immunity	PAMP|Effector Triggered Immunity|PTI	PTI-Flagellin Triggered Immunity	fliC|laf1|lafA|fla1|hag->PGPT0015190	1
Indirect Effects	PlantImmune Response Stimulation	Triggered Immunity	PAMP|Effector Triggered Immunity|PTI	PTI-Flagellin Triggered Immunity	fliD|flaB->PGPT0015200	1
Indirect Effects	PlantImmune Response Stimulatio	Triggered Immunity	PAMP|Effector Triggered Immunity|PTI	PTI-PR1 Like Proteins	hptG->PGPT0014640	1

	n					
Indirect Effects	PlantImmune Response Stimulation	Triggered Immunity	PAMP|Effector Triggered Immunity|PTI	PTI-Teichuronic Acid|LPS Elicitor	tuaG|ggaB->PGPT0015205	2
Indirect Effects	PlantImmune Response Stimulation	Triggered Immunity	PAMP|Effector Triggered Immunity|PTI	PTI-Teichuronic Acid|LPS Elicitor	wecA|tagO|rfe->PGPT0015240	1
Indirect Effects	StressControl|Biocontrol	Neutralizing ABiotic Stress	High Temperature Tolerance	Drought Stress Regulation	yciG|ymdF|gsiB->PGPT0002680	3
Indirect Effects	StressControl|Biocontrol	Neutralizing ABiotic Stress	High Temperature Tolerance	Heat Affected Cell Wall|Membrane Proteins	sacB->PGPT0013775	2
Indirect Effects	StressControl|Biocontrol	Neutralizing ABiotic Stress	High Temperature Tolerance	Heat Affected Lipopolysaccharide Assembly	lapA->PGPT0014665	1
Indirect Effects	StressControl|Biocontrol	Neutralizing ABiotic Stress	High Temperature Tolerance	Heat Inducible Proteins	dps|dpsA->PGPT0004055	2
Indirect Effects	StressControl|Biocontrol	Neutralizing ABiotic Stress	High Temperature Tolerance	Heat Inducible Proteins	pspE->PGPT0014648	2
Indirect Effects	StressControl|Biocontrol	Neutralizing ABiotic Stress	High Temperature Tolerance	Heat Inducible Proteins	Toxin pspC->PGPT0027520	1
Indirect Effects	StressControl|Biocontrol	Neutralizing ABiotic Stress	High Temperature Tolerance	Heat Inducible Proteins	yciG|ymdF|gsiB->PGPT0002680	3

Indirect Effects	StressControl|Biocontrol	Neutralizing ABiotic Stress	High Temperature Tolerance	Heat Inducible Proteins	yflT->PGPT0014649	10
Indirect Effects	StressControl|Biocontrol	Neutralizing ABiotic Stress	High Temperature Tolerance	Heat Shock Proteins	clpB->PGPT0014580	1
Indirect Effects	StressControl|Biocontrol	Neutralizing ABiotic Stress	High Temperature Tolerance	Heat Shock Proteins	clpC->PGPT0014585	1
Indirect Effects	StressControl|Biocontrol	Neutralizing ABiotic Stress	High Temperature Tolerance	Heat Shock Proteins	clpE->PGPT0014590	1
Indirect Effects	StressControl|Biocontrol	Neutralizing ABiotic Stress	High Temperature Tolerance	Heat Shock Proteins	clpP->PGPT0014595	4
Indirect Effects	StressControl|Biocontrol	Neutralizing ABiotic Stress	High Temperature Tolerance	Heat Shock Proteins	clpX->PGPT0014600	1
Indirect Effects	StressControl|Biocontrol	Neutralizing ABiotic Stress	High Temperature Tolerance	Heat Shock Proteins	ctsR->PGPT0014610	1
Indirect Effects	StressControl|Biocontrol	Neutralizing ABiotic Stress	High Temperature Tolerance	Heat Shock Proteins	dnaJ->PGPT0014545	1
Indirect Effects	StressControl|Biocontrol	Neutralizing ABiotic Stress	High Temperature Tolerance	Heat Shock Proteins	dnaK->PGPT0014555	1
Indirect Effects	StressControl|Biocontrol	Neutralizing ABiotic Stress	High Temperature Tolerance	Heat Shock Proteins	groEL|mopA->PGPT0014570	1
Indirect Effects	StressControl|Bioco	Neutralizing ABiotic Stress	High Temperature Tolerance	Heat Shock Proteins	groES|mopB->PGPT0014565	1

	ntrol					
Indirect Effects	StressControl|Biocontrol	Neutralizing ABiotic Stress	High Temperature Tolerance	Heat Shock Proteins	hptG->PGPT0014640	1
Indirect Effects	StressControl|Biocontrol	Neutralizing ABiotic Stress	High Temperature Tolerance	Heat Shock Proteins	hsp20->PGPT0014635	4
Indirect Effects	StressControl|Biocontrol	Neutralizing ABiotic Stress	High Temperature Tolerance	Heat Shock Proteins	hsp33|hslO->PGPT0014645	1
Indirect Effects	StressControl|Biocontrol	Neutralizing ABiotic Stress	High Temperature Tolerance	Heat Shock Proteins	htpX|ykrL->PGPT0014605	2
Indirect Effects	StressControl|Biocontrol	Neutralizing ABiotic Stress	High Temperature Tolerance	High Temperature Regulation	arsC->PGPT0004715	2
Indirect Effects	StressControl|Biocontrol	Neutralizing ABiotic Stress	High Temperature Tolerance	High Temperature Regulation	arsC1->PGPT0004720	1
Indirect Effects	StressControl|Biocontrol	Neutralizing ABiotic Stress	High Temperature Tolerance	High Temperature Regulation	grpE->PGPT0014650	1
Indirect Effects	StressControl|Biocontrol	Neutralizing ABiotic Stress	High Temperature Tolerance	High Temperature Regulation	vicK|walK|yycG|micB->PGPT0013760	1
Indirect Effects	StressControl|Biocontrol	Neutralizing ABiotic Stress	High Temperature Tolerance	High Temperature Regulation	vicR|walR|yycF|micA->PGPT0013765	3
Indirect Effects	StressControl|Biocontrol	Neutralizing ABiotic Stress	Low Temperature Tolerance	Cold Affected Biofilm|Quorum Sensing	rpoS->PGPT0014685	1
Indirect	StressCon	Neutralizing ABiotic	Low Temperature	Cold Shock Proteins	cspA->PGPT0014675	21

Effects	trol|Biocontrol	Stress	Tolerance			
Indirect Effects	StressControl|Biocontrol	Neutralizing ABiotic Stress	Low Temperature Tolerance	Low Temperature-Related Enzymes	gdh|ycdF->PGPT0014705	7
Indirect Effects	StressControl|Biocontrol	Neutralizing ABiotic Stress	Low Temperature Tolerance	Low Temperature-Related Enzymes	terD|terE|terF->PGPT0004910	3
Indirect Effects	StressControl|Biocontrol	Neutralizing ABiotic Stress	Low Temperature Tolerance	Low Temperature-Related Enzymes	yfhE->PGPT0014725	1
Indirect Effects	StressControl|Biocontrol	Neutralizing ABiotic Stress	Low Temperature Tolerance	Low Temperature-Related Enzymes	yfhF->PGPT0014730	1
Indirect Effects	StressControl|Biocontrol	Neutralizing ABiotic Stress	Neutralizing Acidic Stress	Acidic Stress-Cell Envelope Modification Protein	sacB->PGPT0013775	2
Indirect Effects	StressControl|Biocontrol	Neutralizing ABiotic Stress	Neutralizing Acidic Stress	Acidic Stress-Low Acid Signaling	vicK|walK|yycG|micB->PGPT0013760	1
Indirect Effects	StressControl|Biocontrol	Neutralizing ABiotic Stress	Neutralizing Acidic Stress	Acidic Stress-Low Acid Signaling	vicR|walR|yycF|micA->PGPT0013765	3
Indirect Effects	StressControl|Biocontrol	Neutralizing ABiotic Stress	Neutralizing Acidic Stress	Acidic Stress-Quorum Sensing Mediated Tolerance	gadA|gadB->PGPT0007630	1
Indirect Effects	StressControl|Biocontrol	Neutralizing ABiotic Stress	Neutralizing Acidic Stress	Acidic Stress-SOxalate-Induced Acid Tolerance	frc|yfdW->PGPT0002130	2
Indirect Effects	StressControl|Biocontrol	Neutralizing ABiotic Stress	Neutralizing Acidic Stress	Acidic Stress-SOxalate-Induced Acid Tolerance	mdcF->PGPT0001720	2

Indirect Effects	StressControl|Biocontrol	Neutralizing ABiotic Stress	Neutralizing Acidic Stress	Acidic Stress-Spermidine|Putrescine Metabolism	gsp->PGPT0007755	1
Indirect Effects	StressControl|Biocontrol	Neutralizing ABiotic Stress	Neutralizing Acidic Stress	Acidic Stress-Spermidine|Putrescine Metabolism	speE|SRM|SPEC3|SPSD->PGPT0007750	3
Indirect Effects	StressControl|Biocontrol	Neutralizing ABiotic Stress	Neutralizing Acidic Stress	Acidic Stress Regulation	rpoS->PGPT0014685	1
Indirect Effects	StressControl|Biocontrol	Neutralizing ABiotic Stress	Neutralizing Acidic Stress	Acidic Stress Regulation	yciG|ymdF|gsiB->PGPT0002680	3
Indirect Effects	StressControl|Biocontrol	Neutralizing ABiotic Stress	Neutralizing Herbicidal Stress	Herbicidal Stress-OrganoPhosphate Degradation	glpA|glpD->PGPT0006775	1
Indirect Effects	StressControl|Biocontrol	Neutralizing ABiotic Stress	Neutralizing Herbicidal Stress	Herbicidal Stress-OrganoPhosphate Degradation	opaA|pepQ->PGPT0006760	1
Indirect Effects	StressControl|Biocontrol	Neutralizing ABiotic Stress	Neutralizing Herbicidal Stress	Herbicidal Stress-OrganoPhosphate Degradation	pepP->PGPT0006770	1
Indirect Effects	StressControl|Biocontrol	Neutralizing ABiotic Stress	Neutralizing Herbicidal Stress	Herbicidal Stress-OrganoPhosphate Degradation	php|opd|adpB->PGPT0006755	1
Indirect Effects	StressControl|Biocontrol	Neutralizing ABiotic Stress	Neutralizing Herbicidal Stress	Herbicidal Stress-Paraquat Stress Reduction	aldH|dhaS->PGPT0006875	5
Indirect Effects	StressControl|Biocontrol	Neutralizing ABiotic Stress	Neutralizing Herbicidal Stress	Herbicidal Stress-Paraquat Stress Reduction	clsA B|ybhO|ywiE->PGPT0007725	5
Indirect Effects	StressControl|Bioco	Neutralizing ABiotic Stress	Neutralizing Herbicidal Stress	Herbicidal Stress-Paraquat Stress	clsC|ymdC->PGPT0007730	2

	ntrol			Reduction		
Indirect Effects	StressControl|Biocontrol	Neutralizing ABiotic Stress	Neutralizing Herbicidal Stress	Herbicidal Stress-Paraquat Stress Reduction	csbA->PGPT0015022	1
Indirect Effects	StressControl|Biocontrol	Neutralizing ABiotic Stress	Neutralizing Herbicidal Stress	Herbicidal Stress-Paraquat Stress Reduction	ydaG->PGPT0015019	2
Indirect Effects	StressControl|Biocontrol	Neutralizing ABiotic Stress	Neutralizing Herbicidal Stress	Herbicidal Stress-Paraquat Stress Reduction	ydhK->PGPT0015018	1
Indirect Effects	StressControl|Biocontrol	Neutralizing ABiotic Stress	Neutralizing Herbicidal Stress	Herbicidal Stress-Paraquat Stress Reduction	yerD->PGPT0014521	1
Indirect Effects	StressControl|Biocontrol	Neutralizing ABiotic Stress	Neutralizing Herbicidal Stress	Herbicidal Stress-Paraquat Stress Reduction	yfkH->PGPT0014525	3
Indirect Effects	StressControl|Biocontrol	Neutralizing ABiotic Stress	Neutralizing Herbicidal Stress	Herbicidal Stress-Paraquat Stress Reduction	yflA|TC AGCS->PGPT0014405	5
Indirect Effects	StressControl|Biocontrol	Neutralizing ABiotic Stress	Neutralizing Herbicidal Stress	Herbicidal Stress-Paraquat Stress Reduction	yitT->PGPT0014526	15
Indirect Effects	StressControl|Biocontrol	Neutralizing ABiotic Stress	Neutralizing Herbicidal Stress	Herbicidal Stress-Paraquat Stress Reduction	yjbC->PGPT0014311	1
Indirect Effects	StressControl|Biocontrol	Neutralizing ABiotic Stress	Neutralizing Herbicidal Stress	Herbicidal Stress-Paraquat Stress Reduction	yjgD->PGPT0015032	1
Indirect Effects	StressControl|Biocontrol	Neutralizing ABiotic Stress	Neutralizing Herbicidal Stress	Herbicidal Stress-Paraquat Stress Reduction	ylxP->PGPT0014535	1
Indirect	StressCon	Neutralizing ABiotic	Neutralizing	Herbicidal Stress-	yqhQ->PGPT0015034	1

Effects	trol|Biocontrol	Stress	Herbicidal Stress	Paraquat Stress Reduction		
Indirect Effects	StressControl|Biocontrol	Neutralizing ABiotic Stress	Neutralizing Herbicidal Stress	Herbicidal Stress-Paraquat Stress Reduction	yugU->PGPT0015021	1
Indirect Effects	StressControl|Biocontrol	Neutralizing ABiotic Stress	Neutralizing Herbicidal Stress	Herbicidal Stress-Toxoflavin Metabolism	ribA->PGPT0007985	1
Indirect Effects	StressControl|Biocontrol	Neutralizing ABiotic Stress	Neutralizing Herbicidal Stress	Herbicidal Stress-Toxoflavin Metabolism	ribD->PGPT0008555	2
Indirect Effects	StressControl|Biocontrol	Neutralizing ABiotic Stress	Neutralizing Herbicidal Stress	Herbicidal Stress-Toxoflavin Metabolism	ToxF->PGPT0009795	3
Indirect Effects	StressControl|Biocontrol	Neutralizing ABiotic Stress	Neutralizing Herbicidal Stress	Herbicidal Stress-Toxoflavin Metabolism	ToxG|mexH->PGPT0009800	1
Indirect Effects	StressControl|Biocontrol	Neutralizing ABiotic Stress	Neutralizing Osmotic Stress	Osmotic Stress-1|3-Diaminopropane Biosynthesis	ddc|dfoJ|desA->PGPT0013725	1
Indirect Effects	StressControl|Biocontrol	Neutralizing ABiotic Stress	Neutralizing Osmotic Stress	Osmotic Stress-Cardiolipin Biosynthesis	clsA B|ybhO|ywiE->PGPT0007725	5
Indirect Effects	StressControl|Biocontrol	Neutralizing ABiotic Stress	Neutralizing Osmotic Stress	Osmotic Stress-Cardiolipin Biosynthesis	clsC|ymdC->PGPT0007730	2
Indirect Effects	StressControl|Biocontrol	Neutralizing ABiotic Stress	Neutralizing Osmotic Stress	Osmotic Stress-Glycine-Betaine Metabolism	betB Homologous->PGPT0007165	4
Indirect Effects	StressControl|Biocontrol	Neutralizing ABiotic Stress	Neutralizing Osmotic Stress	Osmotic Stress-Glycine-Betaine Metabolism	dmg Like->PGPT0013545	1

Indirect Effects	StressControl|Biocontrol	Neutralizing ABiotic Stress	Neutralizing Osmotic Stress	Osmotic Stress-Glycine-Betaine Metabolism	gbcA->PGPT0013680	1
Indirect Effects	StressControl|Biocontrol	Neutralizing ABiotic Stress	Neutralizing Osmotic Stress	Osmotic Stress-Glycine-Betaine Metabolism	gbsB->PGPT0008300	1
Indirect Effects	StressControl|Biocontrol	Neutralizing ABiotic Stress	Neutralizing Osmotic Stress	Osmotic Stress-Glycine-Betaine Metabolism	gbuA|proV->PGPT0013630	2
Indirect Effects	StressControl|Biocontrol	Neutralizing ABiotic Stress	Neutralizing Osmotic Stress	Osmotic Stress-Glycine-Betaine Metabolism	gbuB|proW->PGPT0013635	2
Indirect Effects	StressControl|Biocontrol	Neutralizing ABiotic Stress	Neutralizing Osmotic Stress	Osmotic Stress-Glycine-Betaine Metabolism	gbuC|proX->PGPT0013640	2
Indirect Effects	StressControl|Biocontrol	Neutralizing ABiotic Stress	Neutralizing Osmotic Stress	Osmotic Stress-Glycine-Betaine Metabolism	opuA|osmV|yehX->PGPT0013585	1
Indirect Effects	StressControl|Biocontrol	Neutralizing ABiotic Stress	Neutralizing Osmotic Stress	Osmotic Stress-Glycine-Betaine Metabolism	opuC|yehZ->PGPT0013595	1
Indirect Effects	StressControl|Biocontrol	Neutralizing ABiotic Stress	Neutralizing Osmotic Stress	Osmotic Stress-Glycine-Betaine Metabolism	opuD|ytfQ->PGPT0013600	3
Indirect Effects	StressControl|Biocontrol	Neutralizing ABiotic Stress	Neutralizing Osmotic Stress	Osmotic Stress-Glycine-Betaine Metabolism	proP->PGPT0013645	1
Indirect Effects	StressControl|Biocontrol	Neutralizing ABiotic Stress	Neutralizing Osmotic Stress	Osmotic Stress-Glycine-Betaine Metabolism	sOxa->PGPT0013515	2
Indirect Effects	StressControl|Bioco	Neutralizing ABiotic Stress	Neutralizing Osmotic Stress	Osmotic Stress-Glycine-Betaine	soxA|solA->PGPT0013530	1

	ntrol			Metabolism		
Indirect Effects	StressControl|Biocontrol	Neutralizing ABiotic Stress	Neutralizing Osmotic Stress	Osmotic Stress-Mannitol Biosynthesis	mtlD->PGPT0013700	1
Indirect Effects	StressControl|Biocontrol	Neutralizing ABiotic Stress	Neutralizing Osmotic Stress	Osmotic Stress-Mechanosensitive Ion Channel	mscL->PGPT0013771	1
Indirect Effects	StressControl|Biocontrol	Neutralizing ABiotic Stress	Neutralizing Osmotic Stress	Osmotic Stress-Mechanosensitive Ion Channel	ykuT|ybiO->PGPT0013695	4
Indirect Effects	StressControl|Biocontrol	Neutralizing ABiotic Stress	Neutralizing Osmotic Stress	Osmotic Stress-Other Regulators	nth->PGPT0013735	1
Indirect Effects	StressControl|Biocontrol	Neutralizing ABiotic Stress	Neutralizing Osmotic Stress	Osmotic Stress-Other Regulators	rhlE->PGPT0013740	5
Indirect Effects	StressControl|Biocontrol	Neutralizing ABiotic Stress	Neutralizing Osmotic Stress	Osmotic Stress-Potassium transport	cvrA|nhaP2->PGPT0013860	1
Indirect Effects	StressControl|Biocontrol	Neutralizing ABiotic Stress	Neutralizing Osmotic Stress	Osmotic Stress-Potassium transport	kch|trkA|mthK|pch->PGPT0002715	1
Indirect Effects	StressControl|Biocontrol	Neutralizing ABiotic Stress	Neutralizing Osmotic Stress	Osmotic Stress-Potassium transport	kefG->PGPT0013810	3
Indirect Effects	StressControl|Biocontrol	Neutralizing ABiotic Stress	Neutralizing Osmotic Stress	Osmotic Stress-Potassium transport	lctB->PGPT0013815	1
Indirect Effects	StressControl|Biocontrol	Neutralizing ABiotic Stress	Neutralizing Osmotic Stress	Osmotic Stress-Potassium transport	trkA|ktrA->PGPT0002710	2
Indirect	StressCon	Neutralizing ABiotic	Neutralizing Osmotic	Osmotic Stress-	trkG|trkH|ktrB-	3

<table>
<tr><td>Effects</td><td>trol|Biocontrol</td><td>Stress</td><td>Stress</td><td>Potassium transport</td><td>>PGPT0002735</td><td></td></tr>
<tr><td>Indirect Effects</td><td>StressControl|Biocontrol</td><td>Neutralizing ABiotic Stress</td><td>Neutralizing Osmotic Stress</td><td>Osmotic Stress-Secretion System</td><td>yggT|ylmG->PGPT0013730</td><td>1</td></tr>
<tr><td>Indirect Effects</td><td>StressControl|Biocontrol</td><td>Neutralizing ABiotic Stress</td><td>Neutralizing Osmotic Stress</td><td>Osmotic Stress-Spermidine|Putrescine Metabolism</td><td>gsp->PGPT0007755</td><td>1</td></tr>
<tr><td>Indirect Effects</td><td>StressControl|Biocontrol</td><td>Neutralizing ABiotic Stress</td><td>Neutralizing Osmotic Stress</td><td>Osmotic Stress-Spermidine|Putrescine Metabolism</td><td>paiA->PGPT0007785</td><td>10</td></tr>
<tr><td>Indirect Effects</td><td>StressControl|Biocontrol</td><td>Neutralizing ABiotic Stress</td><td>Neutralizing Osmotic Stress</td><td>Osmotic Stress-Spermidine|Putrescine Metabolism</td><td>patA1->PGPT0007155</td><td>1</td></tr>
<tr><td>Indirect Effects</td><td>StressControl|Biocontrol</td><td>Neutralizing ABiotic Stress</td><td>Neutralizing Osmotic Stress</td><td>Osmotic Stress-Spermidine|Putrescine Metabolism</td><td>potA->PGPT0007840</td><td>1</td></tr>
<tr><td>Indirect Effects</td><td>StressControl|Biocontrol</td><td>Neutralizing ABiotic Stress</td><td>Neutralizing Osmotic Stress</td><td>Osmotic Stress-Spermidine|Putrescine Metabolism</td><td>potB->PGPT0007835</td><td>1</td></tr>
<tr><td>Indirect Effects</td><td>StressControl|Biocontrol</td><td>Neutralizing ABiotic Stress</td><td>Neutralizing Osmotic Stress</td><td>Osmotic Stress-Spermidine|Putrescine Metabolism</td><td>potC->PGPT0007830</td><td>1</td></tr>
<tr><td>Indirect Effects</td><td>StressControl|Biocontrol</td><td>Neutralizing ABiotic Stress</td><td>Neutralizing Osmotic Stress</td><td>Osmotic Stress-Spermidine|Putrescine Metabolism</td><td>potD->PGPT0007825</td><td>1</td></tr>
<tr><td>Indirect Effects</td><td>StressControl|Biocontrol</td><td>Neutralizing ABiotic Stress</td><td>Neutralizing Osmotic Stress</td><td>Osmotic Stress-Spermidine|Putrescine Metabolism</td><td>puuP->PGPT0007795</td><td>4</td></tr>
<tr><td>Indirect Effects</td><td>StressControl|Biocontrol</td><td>Neutralizing ABiotic Stress</td><td>Neutralizing Osmotic Stress</td><td>Osmotic Stress-Spermidine|Putrescine Metabolism</td><td>speB->PGPT0007775</td><td>3</td></tr>
</table>

Indirect Effects	StressControl|Biocontrol	Neutralizing ABiotic Stress	Neutralizing Osmotic Stress	Osmotic Stress-Spermidine|Putrescine Metabolism	speE|SRM|SPEC3|SPSD->PGPT0007750	3
Indirect Effects	StressControl|Biocontrol	Neutralizing ABiotic Stress	Neutralizing Osmotic Stress	Osmotic Stress-Spermidine|Putrescine Metabolism	speG->PGPT0007790	6
Indirect Effects	StressControl|Biocontrol	Neutralizing ABiotic Stress	Neutralizing Osmotic Stress	Osmotic Stress-trigonelline Biosynthesis	nadA->PGPT0013365	1
Indirect Effects	StressControl|Biocontrol	Neutralizing ABiotic Stress	Neutralizing Osmotic Stress	Osmotic Stress-Vitamin B3|Niacin Biosynthesis	deoD->PGPT0013385	1
Indirect Effects	StressControl|Biocontrol	Neutralizing ABiotic Stress	Neutralizing Osmotic Stress	Osmotic Stress-Vitamin B3|Niacin Biosynthesis	iunH->PGPT0013465	2
Indirect Effects	StressControl|Biocontrol	Neutralizing ABiotic Stress	Neutralizing Osmotic Stress	Osmotic Stress-Vitamin B3|Niacin Biosynthesis	nadA->PGPT0013365	1
Indirect Effects	StressControl|Biocontrol	Neutralizing ABiotic Stress	Neutralizing Osmotic Stress	Osmotic Stress-Vitamin B3|Niacin Biosynthesis	nadB->PGPT0013355	1
Indirect Effects	StressControl|Biocontrol	Neutralizing ABiotic Stress	Neutralizing Osmotic Stress	Osmotic Stress-Vitamin B3|Niacin Biosynthesis	nadC->PGPT0013370	1
Indirect Effects	StressControl|Biocontrol	Neutralizing ABiotic Stress	Neutralizing Osmotic Stress	Osmotic Stress-Vitamin B3|Niacin Biosynthesis	nadD->PGPT0013435	3
Indirect Effects	StressControl|Biocontrol	Neutralizing ABiotic Stress	Neutralizing Osmotic Stress	Osmotic Stress-Vitamin B3|Niacin Biosynthesis	nadE->PGPT0013445	2
Indirect Effects	StressControl|Bioco	Neutralizing ABiotic Stress	Neutralizing Osmotic Stress	Osmotic Stress-Vitamin B3|Niacin	pncB->PGPT0013375	2

	ntrol			Biosynthesis		
Indirect Effects	StressControl|Biocontrol	Neutralizing ABiotic Stress	Neutralizing Osmotic Stress	Osmotic Stress-Vitamin B3|Niacin Biosynthesis	pncC->PGPT0013455	1
Indirect Effects	StressControl|Biocontrol	Neutralizing ABiotic Stress	Neutralizing Osmotic Stress	Osmotic Stress-Vitamin B3|Niacin Biosynthesis	punA->PGPT0013380	1
Indirect Effects	StressControl|Biocontrol	Neutralizing ABiotic Stress	Neutralizing Osmotic Stress	Osmotic Stress-Vitamin B3|Niacin Biosynthesis	yfkN->PGPT0013420	1
Indirect Effects	StressControl|Biocontrol	Neutralizing ABiotic Stress	Neutralizing Osmotic Stress	Osmotic Stress-Vitamin B6|Pyridoxine|Pyridoxal|PyridoxAmine Biosynthesis	pdxA->PGPT0009165	1
Indirect Effects	StressControl|Biocontrol	Neutralizing ABiotic Stress	Neutralizing Osmotic Stress	Osmotic Stress-Vitamin B6|Pyridoxine|Pyridoxal|PyridoxAmine Biosynthesis	pdxK|pdxY->PGPT0009125	1
Indirect Effects	StressControl|Biocontrol	Neutralizing ABiotic Stress	Neutralizing Osmotic Stress	Osmotic Stress-Vitamin B6|Pyridoxine|Pyridoxal|PyridoxAmine Biosynthesis	pdxS|pdx1|yaaD->PGPT0009180	2
Indirect Effects	StressControl|Biocontrol	Neutralizing ABiotic Stress	Neutralizing Osmotic Stress	Osmotic Stress-Vitamin B6|Pyridoxine|Pyridoxal|PyridoxAmine Biosynthesis	pdxT|pdx2|yaaE->PGPT0009185	2
Indirect Effects	StressControl|Bioco	Neutralizing ABiotic Stress	Neutralizing Osmotic Stress	Osmotic Stress-Vitamin	serA->PGPT0009155	2

	ntrol			B6|Pyridoxine|Pyridoxal|PyridoxAmine Biosynthesis		
Indirect Effects	StressControl|Biocontrol	Neutralizing ABiotic Stress	Neutralizing Osmotic Stress	Osmotic Stress-Vitamin B6|Pyridoxine|Pyridoxal|PyridoxAmine Biosynthesis	serC|pdxF->PGPT0009160	1
Indirect Effects	StressControl|Biocontrol	Neutralizing ABiotic Stress	Neutralizing Osmotic Stress	Osmotic Stress-Vitamin B6|Pyridoxine|Pyridoxal|PyridoxAmine Biosynthesis	thrC->PGPT0009175	5
Indirect Effects	StressControl|Biocontrol	Neutralizing ABiotic Stress	Neutralizing Osmotic Stress	Osmotic Stress Signaling	pspE->PGPT0014648	2
Indirect Effects	StressControl|Biocontrol	Neutralizing ABiotic Stress	Neutralizing Osmotic Stress	Osmotic Stress Signaling	sacB->PGPT0013775	2
Indirect Effects	StressControl|Biocontrol	Neutralizing ABiotic Stress	Neutralizing Osmotic Stress	Osmotic Stress Signaling	Toxin pspC->PGPT0027520	1
Indirect Effects	StressControl|Biocontrol	Neutralizing ABiotic Stress	Neutralizing Osmotic Stress	Osmotic Stress Signaling	vicK|walK|yycG|micB->PGPT0013760	1
Indirect Effects	StressControl|Biocontrol	Neutralizing ABiotic Stress	Neutralizing Osmotic Stress	Osmotic Stress Signaling	vicR|walR|yycF|micA->PGPT0013765	3
Indirect Effects	StressControl|Biocontrol	Neutralizing ABiotic Stress	Neutralizing Salinity Stress	Salinity Stress-Calcium transport	TC CITMHS|CitMHS Family|citN->PGPT0001500	6
Indirect	StressCon	Neutralizing ABiotic	Neutralizing Salinity	Salinity Stress-	chaA->PGPT0013985	1

Effects	trol|Biocontrol	Stress	Stress	Calcium transport		
Indirect Effects	StressControl|Biocontrol	Neutralizing ABiotic Stress	Neutralizing Salinity Stress	Salinity Stress-Calcium transport	yloB|ctpA->PGPT0013990	1
Indirect Effects	StressControl|Biocontrol	Neutralizing ABiotic Stress	Neutralizing Salinity Stress	Salinity Stress-Cardiolipin Biosynthesis	clsA B|ybhO|ywiE->PGPT0007725	5
Indirect Effects	StressControl|Biocontrol	Neutralizing ABiotic Stress	Neutralizing Salinity Stress	Salinity Stress-Cardiolipin Biosynthesis	clsC|ymdC->PGPT0007730	2
Indirect Effects	StressControl|Biocontrol	Neutralizing ABiotic Stress	Neutralizing Salinity Stress	Salinity Stress-Cation transport	TC CITMHS|CitMHS Family|citN->PGPT0001500	6
Indirect Effects	StressControl|Biocontrol	Neutralizing ABiotic Stress	Neutralizing Salinity Stress	Salinity Stress-Cation transport	TC GPH|yihO|xynP->PGPT0014410	3
Indirect Effects	StressControl|Biocontrol	Neutralizing ABiotic Stress	Neutralizing Salinity Stress	Salinity Stress-Cation transport	TC LIVCS|brnQ->PGPT0014385	3
Indirect Effects	StressControl|Biocontrol	Neutralizing ABiotic Stress	Neutralizing Salinity Stress	Salinity Stress-Cation transport	actP->PGPT0001400	1
Indirect Effects	StressControl|Biocontrol	Neutralizing ABiotic Stress	Neutralizing Salinity Stress	Salinity Stress-Cation transport	chaB->PGPT0014390	1
Indirect Effects	StressControl|Biocontrol	Neutralizing ABiotic Stress	Neutralizing Salinity Stress	Salinity Stress-Cation transport	nhaK|TC CPA1->PGPT0013930	2
Indirect Effects	StressControl|Biocontrol	Neutralizing ABiotic Stress	Neutralizing Salinity Stress	Salinity Stress-Cation transport	ybaL|TC KEF->PGPT0014395	2

Indirect Effects	StressControl|Biocontrol	Neutralizing ABiotic Stress	Neutralizing Salinity Stress	Salinity Stress-Cation transport	yflA|TC AGCS->PGPT0014405	5
Indirect Effects	StressControl|Biocontrol	Neutralizing ABiotic Stress	Neutralizing Salinity Stress	Salinity Stress-Chloride transport	TC CIC|eriC->PGPT0004990	1
Indirect Effects	StressControl|Biocontrol	Neutralizing ABiotic Stress	Neutralizing Salinity Stress	Salinity Stress-Chloride transport	yfbK->PGPT0014015	1
Indirect Effects	StressControl|Biocontrol	Neutralizing ABiotic Stress	Neutralizing Salinity Stress	Salinity Stress-Ectoine Metabolism	asd->PGPT0014045	2
Indirect Effects	StressControl|Biocontrol	Neutralizing ABiotic Stress	Neutralizing Salinity Stress	Salinity Stress-Ectoine Metabolism	doeB->PGPT0014047	1
Indirect Effects	StressControl|Biocontrol	Neutralizing ABiotic Stress	Neutralizing Salinity Stress	Salinity Stress-Ectoine Metabolism	doeX->PGPT0014049	3
Indirect Effects	StressControl|Biocontrol	Neutralizing ABiotic Stress	Neutralizing Salinity Stress	Salinity Stress-Ectoine Metabolism	ectB|dat->PGPT0014050	1
Indirect Effects	StressControl|Biocontrol	Neutralizing ABiotic Stress	Neutralizing Salinity Stress	Salinity Stress-Ectoine Metabolism	lysC->PGPT0014040	3
Indirect Effects	StressControl|Biocontrol	Neutralizing ABiotic Stress	Neutralizing Salinity Stress	Salinity Stress-Glutamate|GlutAmine Synthase|GLTS Pathway	glnA->PGPT0000645	1
Indirect Effects	StressControl|Biocontrol	Neutralizing ABiotic Stress	Neutralizing Salinity Stress	Salinity Stress-Glutamate|GlutAmine Synthase|GLTS Pathway	glnB|glnY->PGPT0000650	2

Indirect Effects	StressControl|Biocontrol	Neutralizing ABiotic Stress	Neutralizing Salinity Stress	Salinity Stress-Glutamate|GlutAmine Synthase|GLTS Pathway	gltB->PGPT0000635	1
Indirect Effects	StressControl|Biocontrol	Neutralizing ABiotic Stress	Neutralizing Salinity Stress	Salinity Stress-Glutamate|GlutAmine Synthase|GLTS Pathway	gltD->PGPT0000640	1
Indirect Effects	StressControl|Biocontrol	Neutralizing ABiotic Stress	Neutralizing Salinity Stress	Salinity Stress-H+ Antiporter	chaA->PGPT0013985	1
Indirect Effects	StressControl|Biocontrol	Neutralizing ABiotic Stress	Neutralizing Salinity Stress	Salinity Stress-H+ Antiporter	cvrA|nhaP2->PGPT0013860	1
Indirect Effects	StressControl|Biocontrol	Neutralizing ABiotic Stress	Neutralizing Salinity Stress	Salinity Stress-H+ Antiporter	mnhA->PGPT0013895	1
Indirect Effects	StressControl|Biocontrol	Neutralizing ABiotic Stress	Neutralizing Salinity Stress	Salinity Stress-H+ Antiporter	mnhB->PGPT0013900	1
Indirect Effects	StressControl|Biocontrol	Neutralizing ABiotic Stress	Neutralizing Salinity Stress	Salinity Stress-H+ Antiporter	mnhC->PGPT0013905	1
Indirect Effects	StressControl|Biocontrol	Neutralizing ABiotic Stress	Neutralizing Salinity Stress	Salinity Stress-H+ Antiporter	mnhD->PGPT0013910	1
Indirect Effects	StressControl|Biocontrol	Neutralizing ABiotic Stress	Neutralizing Salinity Stress	Salinity Stress-H+ Antiporter	mnhE->PGPT0013915	1
Indirect Effects	StressControl|Biocontrol	Neutralizing ABiotic Stress	Neutralizing Salinity Stress	Salinity Stress-H+ Antiporter	mnhF->PGPT0013920	1

Indirect Effects	StressControl|Biocontrol	Neutralizing ABiotic Stress	Neutralizing Salinity Stress	Salinity Stress-H+ Antiporter	mnhG->PGPT0013925	1
Indirect Effects	StressControl|Biocontrol	Neutralizing ABiotic Stress	Neutralizing Salinity Stress	Salinity Stress-H+ Antiporter	nhaC->PGPT0013945	4
Indirect Effects	StressControl|Biocontrol	Neutralizing ABiotic Stress	Neutralizing Salinity Stress	Salinity Stress-H+ Antiporter	nhaK|TC CPA1->PGPT0013930	2
Indirect Effects	StressControl|Biocontrol	Neutralizing ABiotic Stress	Neutralizing Salinity Stress	Salinity Stress-H+ Antiporter	ybaL|TC KEF->PGPT0014395	2
Indirect Effects	StressControl|Biocontrol	Neutralizing ABiotic Stress	Neutralizing Salinity Stress	Salinity Stress-H+ Symporter	TC CITMHS|CitMHS Family|citN->PGPT0001500	6
Indirect Effects	StressControl|Biocontrol	Neutralizing ABiotic Stress	Neutralizing Salinity Stress	Salinity Stress-H+ Symporter	TC DAACS->PGPT0013965	2
Indirect Effects	StressControl|Biocontrol	Neutralizing ABiotic Stress	Neutralizing Salinity Stress	Salinity Stress-H+ Symporter	TC GNTP->PGPT0001340	4
Indirect Effects	StressControl|Biocontrol	Neutralizing ABiotic Stress	Neutralizing Salinity Stress	Salinity Stress-H+ Symporter	araE->PGPT0014420	1
Indirect Effects	StressControl|Biocontrol	Neutralizing ABiotic Stress	Neutralizing Salinity Stress	Salinity Stress-H+ Symporter	glcP->PGPT0014430	3
Indirect Effects	StressControl|Biocontrol	Neutralizing ABiotic Stress	Neutralizing Salinity Stress	Salinity Stress-H+ Symporter	hxt->PGPT0014440	2
Indirect Effects	StressControl|Bioco	Neutralizing ABiotic Stress	Neutralizing Salinity Stress	Salinity Stress-H+ Symporter	xylE->PGPT0014450	1

	ntrol					
Indirect Effects	StressControl|Biocontrol	Neutralizing ABiotic Stress	Neutralizing Salinity Stress	Salinity Stress-H+ Symporter	ydjE->PGPT0014435	2
Indirect Effects	StressControl|Biocontrol	Neutralizing ABiotic Stress	Neutralizing Salinity Stress	Salinity Stress-H2S-Volatile Pathway	cysA->PGPT0002990	1
Indirect Effects	StressControl|Biocontrol	Neutralizing ABiotic Stress	Neutralizing Salinity Stress	Salinity Stress-H2S-Volatile Pathway	cysC->PGPT0002780	2
Indirect Effects	StressControl|Biocontrol	Neutralizing ABiotic Stress	Neutralizing Salinity Stress	Salinity Stress-H2S-Volatile Pathway	cysE->PGPT0002970	1
Indirect Effects	StressControl|Biocontrol	Neutralizing ABiotic Stress	Neutralizing Salinity Stress	Salinity Stress-H2S-Volatile Pathway	cysH->PGPT0002785	1
Indirect Effects	StressControl|Biocontrol	Neutralizing ABiotic Stress	Neutralizing Salinity Stress	Salinity Stress-H2S-Volatile Pathway	cysI->PGPT0002790	1
Indirect Effects	StressControl|Biocontrol	Neutralizing ABiotic Stress	Neutralizing Salinity Stress	Salinity Stress-H2S-Volatile Pathway	cysJ->PGPT0002795	1
Indirect Effects	StressControl|Biocontrol	Neutralizing ABiotic Stress	Neutralizing Salinity Stress	Salinity Stress-H2S-Volatile Pathway	cysK->PGPT0002810	3
Indirect Effects	StressControl|Biocontrol	Neutralizing ABiotic Stress	Neutralizing Salinity Stress	Salinity Stress-H2S-Volatile Pathway	cysK2->PGPT0002815	1
Indirect Effects	StressControl|Biocontrol	Neutralizing ABiotic Stress	Neutralizing Salinity Stress	Salinity Stress-H2S-Volatile Pathway	cysS->PGPT0002985	1
Indirect	StressCon	Neutralizing ABiotic	Neutralizing Salinity	Salinity Stress-H2S-	cysT|cysU-	1

Effects	trol|Biocontrol	Stress	Stress	Volatile Pathway	>PGPT0003000	
Indirect Effects	StressControl|Biocontrol	Neutralizing ABiotic Stress	Neutralizing Salinity Stress	Salinity Stress-H2S-Volatile Pathway	cysW->PGPT0003005	1
Indirect Effects	StressControl|Biocontrol	Neutralizing ABiotic Stress	Neutralizing Salinity Stress	Salinity Stress-H2S-Volatile Pathway	sat|met3->PGPT0002410	1
Indirect Effects	StressControl|Biocontrol	Neutralizing ABiotic Stress	Neutralizing Salinity Stress	Salinity Stress-Halotolerance Related Enzymes	ablA->PGPT0014370	2
Indirect Effects	StressControl|Biocontrol	Neutralizing ABiotic Stress	Neutralizing Salinity Stress	Salinity Stress-Halotolerance Related Enzymes	acrR1->PGPT0014365	1
Indirect Effects	StressControl|Biocontrol	Neutralizing ABiotic Stress	Neutralizing Salinity Stress	Salinity Stress-Halotolerance Related Enzymes	arsC->PGPT0004715	2
Indirect Effects	StressControl|Biocontrol	Neutralizing ABiotic Stress	Neutralizing Salinity Stress	Salinity Stress-Halotolerance Related Enzymes	arsC1->PGPT0004720	1
Indirect Effects	StressControl|Biocontrol	Neutralizing ABiotic Stress	Neutralizing Salinity Stress	Salinity Stress-Halotolerance Related Enzymes	atpA->PGPT0014298	1
Indirect Effects	StressControl|Biocontrol	Neutralizing ABiotic Stress	Neutralizing Salinity Stress	Salinity Stress-Halotolerance Related Enzymes	atpB->PGPT0014303	1
Indirect Effects	StressControl|Biocontrol	Neutralizing ABiotic Stress	Neutralizing Salinity Stress	Salinity Stress-Halotolerance Related Enzymes	atpC->PGPT0014295	1
Indirect Effects	StressControl|Biocontrol	Neutralizing ABiotic Stress	Neutralizing Salinity Stress	Salinity Stress-Halotolerance Related Enzymes	atpD->PGPT0014296	1

Indirect Effects	StressControl|Biocontrol	Neutralizing ABiotic Stress	Neutralizing Salinity Stress	Salinity Stress-Halotolerance Related Enzymes	atpE->PGPT0014302	1
Indirect Effects	StressControl|Biocontrol	Neutralizing ABiotic Stress	Neutralizing Salinity Stress	Salinity Stress-Halotolerance Related Enzymes	atpF->PGPT0014301	1
Indirect Effects	StressControl|Biocontrol	Neutralizing ABiotic Stress	Neutralizing Salinity Stress	Salinity Stress-Halotolerance Related Enzymes	atpG->PGPT0014297	1
Indirect Effects	StressControl|Biocontrol	Neutralizing ABiotic Stress	Neutralizing Salinity Stress	Salinity Stress-Halotolerance Related Enzymes	atpH->PGPT0014299	1
Indirect Effects	StressControl|Biocontrol	Neutralizing ABiotic Stress	Neutralizing Salinity Stress	Salinity Stress-Halotolerance Related Enzymes	dps|dpsA->PGPT0004055	2
Indirect Effects	StressControl|Biocontrol	Neutralizing ABiotic Stress	Neutralizing Salinity Stress	Salinity Stress-Halotolerance Related Enzymes	lacA->PGPT0014340	1
Indirect Effects	StressControl|Biocontrol	Neutralizing ABiotic Stress	Neutralizing Salinity Stress	Salinity Stress-Halotolerance Related Enzymes	lon->PGPT0014315	2
Indirect Effects	StressControl|Biocontrol	Neutralizing ABiotic Stress	Neutralizing Salinity Stress	Salinity Stress-Halotolerance Related Enzymes	mtnN|pfs|yadA->PGPT0014320	2
Indirect Effects	StressControl|Biocontrol	Neutralizing ABiotic Stress	Neutralizing Salinity Stress	Salinity Stress-Halotolerance Related Enzymes	nadA->PGPT0013365	1
Indirect Effects	StressControl|Biocontrol	Neutralizing ABiotic Stress	Neutralizing Salinity Stress	Salinity Stress-Halotolerance Related Enzymes	prkA|yeaG->PGPT0014345	1
Indirect Effects	StressControl|Bioco	Neutralizing ABiotic Stress	Neutralizing Salinity Stress	Salinity Stress-Halotolerance Related	rpmEB->PGPT0014325	2

	ntrol			Enzymes		
Indirect Effects	StressControl|Biocontrol	Neutralizing ABiotic Stress	Neutralizing Salinity Stress	Salinity Stress-Halotolerance Related Enzymes	rsbV->PGPT0014350	1
Indirect Effects	StressControl|Biocontrol	Neutralizing ABiotic Stress	Neutralizing Salinity Stress	Salinity Stress-Halotolerance Related Enzymes	rseP->PGPT0011685	1
Indirect Effects	StressControl|Biocontrol	Neutralizing ABiotic Stress	Neutralizing Salinity Stress	Salinity Stress-Halotolerance Related Enzymes	smpB->PGPT0014355	1
Indirect Effects	StressControl|Biocontrol	Neutralizing ABiotic Stress	Neutralizing Salinity Stress	Salinity Stress-Halotolerance Related Enzymes	spoT->PGPT0014310	1
Indirect Effects	StressControl|Biocontrol	Neutralizing ABiotic Stress	Neutralizing Salinity Stress	Salinity Stress-Halotolerance Related Enzymes	ycaD->PGPT0014291	1
Indirect Effects	StressControl|Biocontrol	Neutralizing ABiotic Stress	Neutralizing Salinity Stress	Salinity Stress-Halotolerance Related Enzymes	yciG|ymdF|gsiB->PGPT0002680	3
Indirect Effects	StressControl|Biocontrol	Neutralizing ABiotic Stress	Neutralizing Salinity Stress	Salinity Stress-Halotolerance Related Enzymes	yjbC->PGPT0014311	1
Indirect Effects	StressControl|Biocontrol	Neutralizing ABiotic Stress	Neutralizing Salinity Stress	Salinity Stress-Halotolerance Related Enzymes	ykgA->PGPT0014323	1
Indirect Effects	StressControl|Biocontrol	Neutralizing ABiotic Stress	Neutralizing Salinity Stress	Salinity Stress-Halotolerance Related Enzymes	ytaB->PGPT0014330	1
Indirect Effects	StressControl|Biocontrol	Neutralizing ABiotic Stress	Neutralizing Salinity Stress	Salinity Stress-Halotolerance Related Enzymes	yvrE->PGPT0014335	1
Indirect	StressCon	Neutralizing ABiotic	Neutralizing Salinity	Salinity Stress-	ywsB->PGPT0022161	1

Effects	trol|Biocontrol	Stress	Stress	Halotolerance Related Enzymes		
Indirect Effects	StressControl|Biocontrol	Neutralizing ABiotic Stress	Neutralizing Salinity Stress	Salinity Stress-Magnesium transport	citS->PGPT0001490	2
Indirect Effects	StressControl|Biocontrol	Neutralizing ABiotic Stress	Neutralizing Salinity Stress	Salinity Stress-Magnesium transport	corA|yfjQ->PGPT0014010	5
Indirect Effects	StressControl|Biocontrol	Neutralizing ABiotic Stress	Neutralizing Salinity Stress	Salinity Stress-Magnesium transport	corC->PGPT0004695	1
Indirect Effects	StressControl|Biocontrol	Neutralizing ABiotic Stress	Neutralizing Salinity Stress	Salinity Stress-Magnesium transport	mgtC->PGPT0014005	2
Indirect Effects	StressControl|Biocontrol	Neutralizing ABiotic Stress	Neutralizing Salinity Stress	Salinity Stress-Magnesium transport	mgtE->PGPT0013995	1
Indirect Effects	StressControl|Biocontrol	Neutralizing ABiotic Stress	Neutralizing Salinity Stress	Salinity Stress-Magnesium transport	mpfA|yhdP->PGPT0014011	4
Indirect Effects	StressControl|Biocontrol	Neutralizing ABiotic Stress	Neutralizing Salinity Stress	Salinity Stress-Magnesium transport	pmrA->PGPT0003915	3
Indirect Effects	StressControl|Biocontrol	Neutralizing ABiotic Stress	Neutralizing Salinity Stress	Salinity Stress-Potassium transport	cvrA|nhaP2->PGPT0013860	1
Indirect Effects	StressControl|Biocontrol	Neutralizing ABiotic Stress	Neutralizing Salinity Stress	Salinity Stress-Potassium transport	czcO|noxC|yrdP|trkA|hapE->PGPT0013865	1
Indirect Effects	StressControl|Biocontrol	Neutralizing ABiotic Stress	Neutralizing Salinity Stress	Salinity Stress-Potassium transport	kch|trkA|mthK|pch->PGPT0002715	1

Indirect Effects	StressControl|Biocontrol	Neutralizing ABiotic Stress	Neutralizing Salinity Stress	Salinity Stress-Potassium transport	kefG->PGPT0013810	3
Indirect Effects	StressControl|Biocontrol	Neutralizing ABiotic Stress	Neutralizing Salinity Stress	Salinity Stress-Potassium transport	lctB->PGPT0013815	1
Indirect Effects	StressControl|Biocontrol	Neutralizing ABiotic Stress	Neutralizing Salinity Stress	Salinity Stress-Proline Metabolism	TC SSS|yerK|opuE->PGPT0013955	7
Indirect Effects	StressControl|Biocontrol	Neutralizing ABiotic Stress	Neutralizing Salinity Stress	Salinity Stress-Proline Metabolism	argB->PGPT0014249	1
Indirect Effects	StressControl|Biocontrol	Neutralizing ABiotic Stress	Neutralizing Salinity Stress	Salinity Stress-Proline Metabolism	argC->PGPT0014251	1
Indirect Effects	StressControl|Biocontrol	Neutralizing ABiotic Stress	Neutralizing Salinity Stress	Salinity Stress-Proline Metabolism	argD|pqqI->PGPT0014253	3
Indirect Effects	StressControl|Biocontrol	Neutralizing ABiotic Stress	Neutralizing Salinity Stress	Salinity Stress-Proline Metabolism	argE->PGPT0014254	1
Indirect Effects	StressControl|Biocontrol	Neutralizing ABiotic Stress	Neutralizing Salinity Stress	Salinity Stress-Proline Metabolism	argHA->PGPT0014242	1
Indirect Effects	StressControl|Biocontrol	Neutralizing ABiotic Stress	Neutralizing Salinity Stress	Salinity Stress-Proline Metabolism	argJ->PGPT0014244	1
Indirect Effects	StressControl|Biocontrol	Neutralizing ABiotic Stress	Neutralizing Salinity Stress	Salinity Stress-Proline Metabolism	gbuA|proV->PGPT0013630	2
Indirect Effects	StressControl|Bioco	Neutralizing ABiotic Stress	Neutralizing Salinity Stress	Salinity Stress-Proline Metabolism	gbuB|proW->PGPT0013635	2

	ntrol					
Indirect Effects	StressControl|Biocontrol	Neutralizing ABiotic Stress	Neutralizing Salinity Stress	Salinity Stress-Proline Metabolism	gbuC|proX->PGPT0013640	2
Indirect Effects	StressControl|Biocontrol	Neutralizing ABiotic Stress	Neutralizing Salinity Stress	Salinity Stress-Proline Metabolism	prdF->PGPT0014240	1
Indirect Effects	StressControl|Biocontrol	Neutralizing ABiotic Stress	Neutralizing Salinity Stress	Salinity Stress-Proline Metabolism	proA->PGPT0014215	2
Indirect Effects	StressControl|Biocontrol	Neutralizing ABiotic Stress	Neutralizing Salinity Stress	Salinity Stress-Proline Metabolism	proB->PGPT0014220	2
Indirect Effects	StressControl|Biocontrol	Neutralizing ABiotic Stress	Neutralizing Salinity Stress	Salinity Stress-Proline Metabolism	proC->PGPT0014225	3
Indirect Effects	StressControl|Biocontrol	Neutralizing ABiotic Stress	Neutralizing Salinity Stress	Salinity Stress-Proline Metabolism	yobR->PGPT0014246	1
Indirect Effects	StressControl|Biocontrol	Neutralizing ABiotic Stress	Neutralizing Salinity Stress	Salinity Stress-Sodium transport	TC DAACS->PGPT0013965	2
Indirect Effects	StressControl|Biocontrol	Neutralizing ABiotic Stress	Neutralizing Salinity Stress	Salinity Stress-Sodium transport	TC DASS|yflS->PGPT0013960	1
Indirect Effects	StressControl|Biocontrol	Neutralizing ABiotic Stress	Neutralizing Salinity Stress	Salinity Stress-Sodium transport	TC NSS->PGPT0013950	3
Indirect Effects	StressControl|Biocontrol	Neutralizing ABiotic Stress	Neutralizing Salinity Stress	Salinity Stress-Sodium transport	TC SSS|yerK|opuE->PGPT0013955	7
Indirect	StressCon	Neutralizing ABiotic	Neutralizing Salinity	Salinity Stress-Sodium	glnT->PGPT0000745	2

Effects	trol|Biocontrol	Stress	Stress	transport		
Indirect Effects	StressControl|Biocontrol	Neutralizing ABiotic Stress	Neutralizing Salinity Stress	Salinity Stress-Sodium transport	gltS->PGPT0000810	1
Indirect Effects	StressControl|Biocontrol	Neutralizing ABiotic Stress	Neutralizing Salinity Stress	Salinity Stress-Sodium transport	maeN->PGPT0001690	2
Indirect Effects	StressControl|Biocontrol	Neutralizing ABiotic Stress	Neutralizing Salinity Stress	Salinity Stress-Sodium transport	mnhA->PGPT0013895	1
Indirect Effects	StressControl|Biocontrol	Neutralizing ABiotic Stress	Neutralizing Salinity Stress	Salinity Stress-Sodium transport	mnhB->PGPT0013900	1
Indirect Effects	StressControl|Biocontrol	Neutralizing ABiotic Stress	Neutralizing Salinity Stress	Salinity Stress-Sodium transport	mnhC->PGPT0013905	1
Indirect Effects	StressControl|Biocontrol	Neutralizing ABiotic Stress	Neutralizing Salinity Stress	Salinity Stress-Sodium transport	mnhD->PGPT0013910	1
Indirect Effects	StressControl|Biocontrol	Neutralizing ABiotic Stress	Neutralizing Salinity Stress	Salinity Stress-Sodium transport	mnhE->PGPT0013915	1
Indirect Effects	StressControl|Biocontrol	Neutralizing ABiotic Stress	Neutralizing Salinity Stress	Salinity Stress-Sodium transport	mnhF->PGPT0013920	1
Indirect Effects	StressControl|Biocontrol	Neutralizing ABiotic Stress	Neutralizing Salinity Stress	Salinity Stress-Sodium transport	mnhG->PGPT0013925	1
Indirect Effects	StressControl|Biocontrol	Neutralizing ABiotic Stress	Neutralizing Salinity Stress	Salinity Stress-Sodium transport	natA->PGPT0013875	1

Indirect Effects	StressControl|Biocontrol	Neutralizing ABiotic Stress	Neutralizing Salinity Stress	Salinity Stress-Sodium transport	natB->PGPT0013870	2
Indirect Effects	StressControl|Biocontrol	Neutralizing ABiotic Stress	Neutralizing Salinity Stress	Salinity Stress-Sodium transport	nhaC->PGPT0013945	4
Indirect Effects	StressControl|Biocontrol	Neutralizing ABiotic Stress	Neutralizing Salinity Stress	Salinity Stress-Sodium transport	nhaK|TC CPA1->PGPT0013930	2
Indirect Effects	StressControl|Biocontrol	Neutralizing ABiotic Stress	Neutralizing Salinity Stress	Salinity Stress-Sodium transport	panS|yocS|ybaS->PGPT0013890	2
Indirect Effects	StressControl|Biocontrol	Neutralizing ABiotic Stress	Neutralizing Salinity Stress	Salinity Stress-Sodium transport	rnfD|rsxD->PGPT0013275	1
Indirect Effects	StressControl|Biocontrol	Neutralizing ABiotic Stress	Neutralizing Salinity Stress	Salinity Stress-Sucrose Metabolism	scrA|sacP|sacX|ptsS->PGPT0014190	3
Indirect Effects	StressControl|Biocontrol	Neutralizing ABiotic Stress	Neutralizing Salinity Stress	Salinity Stress-Sulfate|Thiosulfate transport	TC SULP->PGPT0003020	4
Indirect Effects	StressControl|Biocontrol	Neutralizing ABiotic Stress	Neutralizing Salinity Stress	Salinity Stress-Sulfate|Thiosulfate transport	cysA->PGPT0002990	1
Indirect Effects	StressControl|Biocontrol	Neutralizing ABiotic Stress	Neutralizing Salinity Stress	Salinity Stress-Sulfate|Thiosulfate transport	cysT|cysU->PGPT0003000	1
Indirect Effects	StressControl|Biocontrol	Neutralizing ABiotic Stress	Neutralizing Salinity Stress	Salinity Stress-Sulfate|Thiosulfate transport	cysW->PGPT0003005	1
Indirect Effects	StressControl|Bioco	Neutralizing ABiotic Stress	Neutralizing Salinity Stress	Salinity Stress-Sulfate|Thiosulfate	sbp->PGPT0003015	1

	ntrol			transport		
Indirect Effects	StressControl|Biocontrol	Neutralizing ABiotic Stress	Neutralizing Salinity Stress	Salinity Stress-Trehalose Metabolism	crr->PGPT0014090	1
Indirect Effects	StressControl|Biocontrol	Neutralizing ABiotic Stress	Neutralizing Salinity Stress	Salinity Stress-Trehalose Metabolism	treB->PGPT0014075	1
Indirect Effects	StressControl|Biocontrol	Neutralizing ABiotic Stress	Neutralizing Salinity Stress	Salinity Stress-Trehalose Metabolism	treC->PGPT0014095	1
Indirect Effects	StressControl|Biocontrol	Neutralizing ABiotic Stress	Neutralizing Salinity Stress	Salinity Stress-Trehalose Metabolism	treR2->PGPT0014110	2
Indirect Effects	StressControl|Biocontrol	Neutralizing ABiotic Stress	Neutralizing Salinity Stress	Salinity Stress-Vitamin B2|Riboflavin Metabolism	RFK|FMN1->PGPT0008615	1
Indirect Effects	StressControl|Biocontrol	Neutralizing ABiotic Stress	Neutralizing Salinity Stress	Salinity Stress-Vitamin B2|Riboflavin Metabolism	bluB|drgA->PGPT0006810	1
Indirect Effects	StressControl|Biocontrol	Neutralizing ABiotic Stress	Neutralizing Salinity Stress	Salinity Stress-Vitamin B2|Riboflavin Metabolism	nfrA1|ywcG->PGPT0000290	1
Indirect Effects	StressControl|Biocontrol	Neutralizing ABiotic Stress	Neutralizing Salinity Stress	Salinity Stress-Vitamin B2|Riboflavin Metabolism	nfrA2|ycnD->PGPT0000295	1
Indirect Effects	StressControl|Biocontrol	Neutralizing ABiotic Stress	Neutralizing Salinity Stress	Salinity Stress-Vitamin B2|Riboflavin Metabolism	ribA->PGPT0007985	1
Indirect Effects	StressControl|Biocontrol	Neutralizing ABiotic Stress	Neutralizing Salinity Stress	Salinity Stress-Vitamin B2|Riboflavin Metabolism	ribBA->PGPT0007990	1
Indirect	StressCon	Neutralizing ABiotic	Neutralizing Salinity	Salinity Stress-Vitamin	ribD->PGPT0008555	2

Effects	trol|Biocontrol	Stress	Stress	B2|Riboflavin Metabolism		
Indirect Effects	StressControl|Biocontrol	Neutralizing ABiotic Stress	Neutralizing Salinity Stress	Salinity Stress-Vitamin B2|Riboflavin Metabolism	ribE|RIB5|ribC->PGPT0008610	1
Indirect Effects	StressControl|Biocontrol	Neutralizing ABiotic Stress	Neutralizing Salinity Stress	Salinity Stress-Vitamin B2|Riboflavin Metabolism	ribF->PGPT0008625	1
Indirect Effects	StressControl|Biocontrol	Neutralizing ABiotic Stress	Neutralizing Salinity Stress	Salinity Stress-Vitamin B2|Riboflavin Metabolism	ribH|RIB4->PGPT0008605	1
Indirect Effects	StressControl|Biocontrol	Neutralizing ABiotic Stress	Neutralizing Salinity Stress	Salinity Stress-Vitamin B2|Riboflavin Metabolism	ssuE->PGPT0003045	1
Indirect Effects	StressControl|Biocontrol	Neutralizing ABiotic Stress	Neutralizing Salinity Stress	Salinity Stress-Vitamin B2|Riboflavin Metabolism	ycsE|yitU|ywtE->PGPT0008595	5
Indirect Effects	StressControl|Biocontrol	Neutralizing ABiotic Stress	Neutralizing Salinity Stress	Salinity Stress-Vitamin B2|Riboflavin Metabolism	yigB->PGPT0008585	1
Indirect Effects	StressControl|Biocontrol	Neutralizing ABiotic Stress	Neutralizing Salinity Stress	Salinity Stress-Vitamin B6|Pyridoxine|Pyridoxal|PyridoxAmine Biosynthesis	pdxA->PGPT0009165	1
Indirect Effects	StressControl|Biocontrol	Neutralizing ABiotic Stress	Neutralizing Salinity Stress	Salinity Stress-Vitamin B6|Pyridoxine|Pyridoxal|PyridoxAmine Biosynthesis	pdxK|pdxY->PGPT0009125	1
Indirect Effects	StressControl|Biocontrol	Neutralizing ABiotic Stress	Neutralizing Salinity Stress	Salinity Stress-Vitamin B6|Pyridoxine|Pyridoxal|PyridoxAmine Biosynthesis	pdxS|pdx1|yaaD->PGPT0009180	2

Indirect Effects	StressControl|Biocontrol	Neutralizing ABiotic Stress	Neutralizing Salinity Stress	Salinity Stress-Vitamin B6|Pyridoxine|Pyridoxal|PyridoxAmine Biosynthesis	pdxT|pdx2|yaaE->PGPT0009185	2
Indirect Effects	StressControl|Biocontrol	Neutralizing ABiotic Stress	Neutralizing Salinity Stress	Salinity Stress-Vitamin B6|Pyridoxine|Pyridoxal|PyridoxAmine Biosynthesis	serA->PGPT0009155	2
Indirect Effects	StressControl|Biocontrol	Neutralizing ABiotic Stress	Neutralizing Salinity Stress	Salinity Stress-Vitamin B6|Pyridoxine|Pyridoxal|PyridoxAmine Biosynthesis	serC|pdxF->PGPT0009160	1
Indirect Effects	StressControl|Biocontrol	Neutralizing ABiotic Stress	Neutralizing Salinity Stress	Salinity Stress-Vitamin B6|Pyridoxine|Pyridoxal|PyridoxAmine Biosynthesis	thrC->PGPT0009175	5
Indirect Effects	StressControl|Biocontrol	Neutralizing ABiotic Stress	Neutralizing Salinity Stress	Salinity Stress-Vitamin B9|Folate Metabolism	fhs->PGPT0008065	1
Indirect Effects	StressControl|Biocontrol	Neutralizing ABiotic Stress	Neutralizing Salinity Stress	Salinity Stress-Vitamin B9|Folate Metabolism	fmt->PGPT0008125	1
Indirect Effects	StressControl|Biocontrol	Neutralizing ABiotic Stress	Neutralizing Salinity Stress	Salinity Stress-Vitamin B9|Folate Metabolism	folA->PGPT0007945	1
Indirect Effects	StressControl|Biocontrol	Neutralizing ABiotic Stress	Neutralizing Salinity Stress	Salinity Stress-Vitamin B9|Folate Metabolism	folB->PGPT0007905	1
Indirect Effects	StressControl|Biocontrol	Neutralizing ABiotic Stress	Neutralizing Salinity Stress	Salinity Stress-Vitamin B9|Folate Metabolism	folC->PGPT0007975	1
Indirect	StressCon	Neutralizing ABiotic	Neutralizing Salinity	Salinity Stress-Vitamin	folD->PGPT0008075	1

Effects	trol|Biocontrol	Stress	Stress	B9|Folate Metabolism		
Indirect Effects	StressControl|Biocontrol	Neutralizing ABiotic Stress	Neutralizing Salinity Stress	Salinity Stress-Vitamin B9|Folate Metabolism	folE->PGPT0007875	2
Indirect Effects	StressControl|Biocontrol	Neutralizing ABiotic Stress	Neutralizing Salinity Stress	Salinity Stress-Vitamin B9|Folate Metabolism	folE2->PGPT0007880	2
Indirect Effects	StressControl|Biocontrol	Neutralizing ABiotic Stress	Neutralizing Salinity Stress	Salinity Stress-Vitamin B9|Folate Metabolism	folK->PGPT0007910	1
Indirect Effects	StressControl|Biocontrol	Neutralizing ABiotic Stress	Neutralizing Salinity Stress	Salinity Stress-Vitamin B9|Folate Metabolism	folP->PGPT0007915	1
Indirect Effects	StressControl|Biocontrol	Neutralizing ABiotic Stress	Neutralizing Salinity Stress	Salinity Stress-Vitamin B9|Folate Metabolism	gcvT->PGPT0008130	1
Indirect Effects	StressControl|Biocontrol	Neutralizing ABiotic Stress	Neutralizing Salinity Stress	Salinity Stress-Vitamin B9|Folate Metabolism	glyA->PGPT0008090	1
Indirect Effects	StressControl|Biocontrol	Neutralizing ABiotic Stress	Neutralizing Salinity Stress	Salinity Stress-Vitamin B9|Folate Metabolism	metH->PGPT0008135	2
Indirect Effects	StressControl|Biocontrol	Neutralizing ABiotic Stress	Neutralizing Salinity Stress	Salinity Stress-Vitamin B9|Folate Metabolism	pabA->PGPT0008000	1
Indirect Effects	StressControl|Biocontrol	Neutralizing ABiotic Stress	Neutralizing Salinity Stress	Salinity Stress-Vitamin B9|Folate Metabolism	pabB->PGPT0008005	1
Indirect Effects	StressControl|Biocontrol	Neutralizing ABiotic Stress	Neutralizing Salinity Stress	Salinity Stress-Vitamin B9|Folate Metabolism	pabC->PGPT0008020	1

Indirect Effects	StressControl|Biocontrol	Neutralizing ABiotic Stress	Neutralizing Salinity Stress	Salinity Stress-Vitamin B9|Folate Metabolism	phoA->PGPT0002570	2
Indirect Effects	StressControl|Biocontrol	Neutralizing ABiotic Stress	Neutralizing Salinity Stress	Salinity Stress-Vitamin B9|Folate Metabolism	phoD->PGPT0002575	1
Indirect Effects	StressControl|Biocontrol	Neutralizing ABiotic Stress	Neutralizing Salinity Stress	Salinity Stress-Vitamin B9|Folate Metabolism	purH->PGPT0008110	2
Indirect Effects	StressControl|Biocontrol	Neutralizing ABiotic Stress	Neutralizing Salinity Stress	Salinity Stress-Vitamin B9|Folate Metabolism	purN->PGPT0008095	1
Indirect Effects	StressControl|Biocontrol	Neutralizing ABiotic Stress	Neutralizing Salinity Stress	Salinity Stress-Vitamin B9|Folate Metabolism	purU->PGPT0008155	1
Indirect Effects	StressControl|Biocontrol	Neutralizing ABiotic Stress	Neutralizing Salinity Stress	Salinity Stress-Vitamin B9|Folate Metabolism	ribA->PGPT0007985	1
Indirect Effects	StressControl|Biocontrol	Neutralizing ABiotic Stress	Neutralizing Salinity Stress	Salinity Stress-Vitamin B9|Folate Metabolism	ribBA->PGPT0007990	1
Indirect Effects	StressControl|Biocontrol	Neutralizing ABiotic Stress	Neutralizing Salinity Stress	Salinity Stress-Vitamin B9|Folate Metabolism	thyA->PGPT0008145	1
Indirect Effects	StressControl|Biocontrol	Neutralizing ABiotic Stress	Neutralizing Salinity Stress	Salinity Stress-Vitamin B9|Folate Metabolism	ygfA|fthC|yqgN|folN->PGPT0008170	1
Indirect Effects	StressControl|Biocontrol	Neutralizing ABiotic Stress	Neutralizing Salinity Stress	Salinity Stress-Vitamin B9|Folate Metabolism	yitJ->PGPT0008140	1
Indirect Effects	StressControl|Bioco	Neutralizing ABiotic Stress	Neutralizing Salinity Stress	Salinity Stress Signaling	degS->PGPT0014285	1

	ntrol					
Indirect Effects	StressControl|Biocontrol	Neutralizing ABiotic Stress	Neutralizing Salinity Stress	Salinity Stress Signaling	degU->PGPT0012750	2
Indirect Effects	StressControl|Biocontrol	Neutralizing ABiotic Stress	Nitrosative|Oxidative Stress|ROS Scavenging	Detoxification Of Peroxidized Compounds	bcp|PRXQ|DOT5->PGPT0013120	1
Indirect Effects	StressControl|Biocontrol	Neutralizing ABiotic Stress	Nitrosative|Oxidative Stress|ROS Scavenging	Detoxification Of Peroxidized Compounds	bsaA|gpx|btuE->PGPT0013115	1
Indirect Effects	StressControl|Biocontrol	Neutralizing ABiotic Stress	Nitrosative|Oxidative Stress|ROS Scavenging	Detoxification Of Peroxidized Compounds	cpo->PGPT0013125	3
Indirect Effects	StressControl|Biocontrol	Neutralizing ABiotic Stress	Nitrosative|Oxidative Stress|ROS Scavenging	Detoxification Of Peroxidized Compounds	efeB->PGPT0003720	1
Indirect Effects	StressControl|Biocontrol	Neutralizing ABiotic Stress	Nitrosative|Oxidative Stress|ROS Scavenging	Detoxification Of Peroxidized Compounds	ohrB|osmC|ohr|ykzA->PGPT0013160	4
Indirect Effects	StressControl|Biocontrol	Neutralizing ABiotic Stress	Nitrosative|Oxidative Stress|ROS Scavenging	Detoxification Of Peroxidized Compounds	ohrR->PGPT0013155	12
Indirect Effects	StressControl|Biocontrol	Neutralizing ABiotic Stress	Nitrosative|Oxidative Stress|ROS Scavenging	Detoxification Of Peroxidized Compounds	oxyR->PGPT0012965	13
Indirect Effects	StressControl|Biocontrol	Neutralizing ABiotic Stress	Nitrosative|Oxidative Stress|ROS Scavenging	Detoxification Of Peroxidized Compounds	tpx->PGPT0013105	1
Indirect Effects	StressControl|Biocontrol	Neutralizing ABiotic Stress	Nitrosative|Oxidative Stress|ROS Scavenging	Detoxification Of Peroxidized Compounds	tsaA|PRDX2 4|ahpC->PGPT0013130	2
Indirect	StressCon	Neutralizing ABiotic	Nitrosative|Oxidative	Detoxification Of	chrC|sodB|sodA-	2

Effects	trol|Biocontrol	Stress	Stress|ROS Scavenging	SuperOxide Anion Radicals	>PGPT0004190	
Indirect Effects	StressControl|Biocontrol	Neutralizing ABiotic Stress	Nitrosative|Oxidative Stress|ROS Scavenging	Detoxification Of SuperOxide Anion Radicals	chrR->PGPT0004195	1
Indirect Effects	StressControl|Biocontrol	Neutralizing ABiotic Stress	Nitrosative|Oxidative Stress|ROS Scavenging	Detoxification Of SuperOxide Anion Radicals	sodC|sod1->PGPT0013181	2
Indirect Effects	StressControl|Biocontrol	Neutralizing ABiotic Stress	Nitrosative|Oxidative Stress|ROS Scavenging	Nitric Oxide Reduction	nsrR|yjeB->PGPT0013005	2
Indirect Effects	StressControl|Biocontrol	Neutralizing ABiotic Stress	Nitrosative|Oxidative Stress|ROS Scavenging	Nitrosative|Oxidative Stress|ROS Regulatory Proteins	arcB|argF|argI->PGPT0020080	1
Indirect Effects	StressControl|Biocontrol	Neutralizing ABiotic Stress	Nitrosative|Oxidative Stress|ROS Scavenging	Nitrosative|Oxidative Stress|ROS Regulatory Proteins	dps|dpsA->PGPT0004055	2
Indirect Effects	StressControl|Biocontrol	Neutralizing ABiotic Stress	Nitrosative|Oxidative Stress|ROS Scavenging	Nitrosative|Oxidative Stress|ROS Regulatory Proteins	oxyR->PGPT0012965	13
Indirect Effects	StressControl|Biocontrol	Neutralizing ABiotic Stress	Nitrosative|Oxidative Stress|ROS Scavenging	Nitrosative|Oxidative Stress|ROS Regulatory Proteins	perR->PGPT0012945	1
Indirect Effects	StressControl|Biocontrol	Neutralizing ABiotic Stress	Nitrosative|Oxidative Stress|ROS Scavenging	Nitrosative|Oxidative Stress|ROS Regulatory Proteins	spx|spxA->PGPT0012940	3
Indirect Effects	StressControl|Biocontrol	Neutralizing ABiotic Stress	Nitrosative|Oxidative Stress|ROS Scavenging	Nitrosative|Oxidative Stress|ROS Regulatory Proteins	yggE->PGPT0012980	1
Indirect Effects	StressControl|Biocontrol	Neutralizing ABiotic Stress	Nitrosative|Oxidative Stress|ROS Scavenging	Oxidative Stress-Arylpolyene Biosynthesis	ABC CD A->PGPT0013345	5

Indirect Effects	StressControl|Biocontrol	Neutralizing ABiotic Stress	Nitrosative|Oxidative Stress|ROS Scavenging	Oxidative Stress-Arylpolyene Biosynthesis	ABC CD P->PGPT0013350	2
Indirect Effects	StressControl|Biocontrol	Neutralizing ABiotic Stress	Nitrosative|Oxidative Stress|ROS Scavenging	Oxidative Stress-Arylpolyene Biosynthesis	UPF0176 Protein->PGPT0013330	3
Indirect Effects	StressControl|Biocontrol	Neutralizing ABiotic Stress	Nitrosative|Oxidative Stress|ROS Scavenging	Oxidative Stress-Arylpolyene Biosynthesis	acpP->PGPT0011375	2
Indirect Effects	StressControl|Biocontrol	Neutralizing ABiotic Stress	Nitrosative|Oxidative Stress|ROS Scavenging	Oxidative Stress-Arylpolyene Biosynthesis	acpS->PGPT0008840	1
Indirect Effects	StressControl|Biocontrol	Neutralizing ABiotic Stress	Nitrosative|Oxidative Stress|ROS Scavenging	Oxidative Stress-Arylpolyene Biosynthesis	clsC|ymdC->PGPT0007730	2
Indirect Effects	StressControl|Biocontrol	Neutralizing ABiotic Stress	Nitrosative|Oxidative Stress|ROS Scavenging	Oxidative Stress-Arylpolyene Biosynthesis	fabF->PGPT0008360	1
Indirect Effects	StressControl|Biocontrol	Neutralizing ABiotic Stress	Nitrosative|Oxidative Stress|ROS Scavenging	Oxidative Stress-Arylpolyene Biosynthesis	ybgC->PGPT0001850	3
Indirect Effects	StressControl|Biocontrol	Neutralizing ABiotic Stress	Nitrosative|Oxidative Stress|ROS Scavenging	Oxidative Stress-Arylpolyene Biosynthesis	ymfI|fabG|efpI->PGPT0003180	14
Indirect Effects	StressControl|Biocontrol	Neutralizing ABiotic Stress	Nitrosative|Oxidative Stress|ROS Scavenging	Oxidative Stress-Carotenoid Biosynthesis	crtE|ispA->PGPT0007560	1
Indirect Effects	StressControl|Biocontrol	Neutralizing ABiotic Stress	Nitrosative|Oxidative Stress|ROS Scavenging	Oxidative Stress-Carotenoid Biosynthesis	crtH|crtISO->PGPT0007400	1
Indirect Effects	StressControl|Bioco	Neutralizing ABiotic Stress	Nitrosative|Oxidative Stress|ROS	Oxidative Stress-Carotenoid	crtI->PGPT0007405	1

	ntrol		Scavenging	Biosynthesis		
Indirect Effects	StressControl|Biocontrol	Neutralizing ABiotic Stress	Nitrosative|Oxidative Stress|ROS Scavenging	Oxidative Stress-Carotenoid Biosynthesis	crtM->PGPT0007420	2
Indirect Effects	StressControl|Biocontrol	Neutralizing ABiotic Stress	Nitrosative|Oxidative Stress|ROS Scavenging	Oxidative Stress-Carotenoid Biosynthesis	crtN->PGPT0007425	1
Indirect Effects	StressControl|Biocontrol	Neutralizing ABiotic Stress	Nitrosative|Oxidative Stress|ROS Scavenging	Oxidative Stress-Carotenoid Biosynthesis	crTo->PGPT0007435	1
Indirect Effects	StressControl|Biocontrol	Neutralizing ABiotic Stress	Nitrosative|Oxidative Stress|ROS Scavenging	Oxidative Stress-Carotenoid Biosynthesis	crtP->PGPT0007440	2
Indirect Effects	StressControl|Biocontrol	Neutralizing ABiotic Stress	Nitrosative|Oxidative Stress|ROS Scavenging	Oxidative Stress-Carotenoid Biosynthesis	crtQ->PGPT0007450	1
Indirect Effects	StressControl|Biocontrol	Neutralizing ABiotic Stress	Nitrosative|Oxidative Stress|ROS Scavenging	Oxidative Stress-Carotenoid Biosynthesis	cruC->PGPT0007495	1
Indirect Effects	StressControl|Biocontrol	Neutralizing ABiotic Stress	Nitrosative|Oxidative Stress|ROS Scavenging	Oxidative Stress-Carotenoid Biosynthesis	cruD->PGPT0007500	1
Indirect Effects	StressControl|Biocontrol	Neutralizing ABiotic Stress	Nitrosative|Oxidative Stress|ROS Scavenging	Oxidative Stress-Chaperones	grxC->PGPT0013201	3
Indirect Effects	StressControl|Biocontrol	Neutralizing ABiotic Stress	Nitrosative|Oxidative Stress|ROS Scavenging	Oxidative Stress-Formaldehyde Fixation	hxlA|yckG->PGPT0013295	3
Indirect Effects	StressControl|Biocontrol	Neutralizing ABiotic Stress	Nitrosative|Oxidative Stress|ROS Scavenging	Oxidative Stress-Formaldehyde Fixation	hxlB|yckF->PGPT0013290	2
Indirect	StressCon	Neutralizing ABiotic	Nitrosative|Oxidative	Oxidative Stress-	ggt->PGPT0002935	6

Effects	trol|Biocontrol	Stress	Stress|ROS Scavenging	Glutathion Metabolism		
Indirect Effects	StressControl|Biocontrol	Neutralizing ABiotic Stress	Nitrosative|Oxidative Stress|ROS Scavenging	Oxidative Stress-Lactoylglutathione Lyase	gloA|ywbC->PGPT0013300	11
Indirect Effects	StressControl|Biocontrol	Neutralizing ABiotic Stress	Nitrosative|Oxidative Stress|ROS Scavenging	Oxidative Stress-Lipoic Acid Metabolism	lipA->PGPT0003935	1
Indirect Effects	StressControl|Biocontrol	Neutralizing ABiotic Stress	Nitrosative|Oxidative Stress|ROS Scavenging	Oxidative Stress-Lipoic Acid Metabolism	lipL->PGPT0003940	1
Indirect Effects	StressControl|Biocontrol	Neutralizing ABiotic Stress	Nitrosative|Oxidative Stress|ROS Scavenging	Oxidative Stress-Oxidative Stress Related Protein	rpoS->PGPT0014685	1
Indirect Effects	StressControl|Biocontrol	Neutralizing ABiotic Stress	Nitrosative|Oxidative Stress|ROS Scavenging	Oxidative Stress-Oxidative Stress Related Protein	yciG|ymdF|gsiB->PGPT0002680	3
Indirect Effects	StressControl|Biocontrol	Neutralizing ABiotic Stress	Nitrosative|Oxidative Stress|ROS Scavenging	Oxidative Stress-OxidoReductases	dltE->PGPT0013220	1
Indirect Effects	StressControl|Biocontrol	Neutralizing ABiotic Stress	Nitrosative|Oxidative Stress|ROS Scavenging	Oxidative Stress-OxidoReductases	fpr->PGPT0013215	1
Indirect Effects	StressControl|Biocontrol	Neutralizing ABiotic Stress	Nitrosative|Oxidative Stress|ROS Scavenging	Oxidative Stress-OxidoReductases	katE|CAT|catB|srpA->PGPT0014380	3
Indirect Effects	StressControl|Biocontrol	Neutralizing ABiotic Stress	Nitrosative|Oxidative Stress|ROS Scavenging	Oxidative Stress-OxidoReductases	katN|ctjC|ydbD|yjqC->PGPT0013240	3
Indirect Effects	StressControl|Biocontrol	Neutralizing ABiotic Stress	Nitrosative|Oxidative Stress|ROS Scavenging	Oxidative Stress-OxidoReductases	qor->PGPT0013255	4

Indirect Effects	StressControl|Biocontrol	Neutralizing ABiotic Stress	Nitrosative|Oxidative Stress|ROS Scavenging	Oxidative Stress-OxidoReductases	rnfD|rsxD->PGPT0013275	1
Indirect Effects	StressControl|Biocontrol	Neutralizing ABiotic Stress	Nitrosative|Oxidative Stress|ROS Scavenging	Oxidative Stress-Spermidine|Putrescine Metabolism	gsp->PGPT0007755	1
Indirect Effects	StressControl|Biocontrol	Neutralizing ABiotic Stress	Nitrosative|Oxidative Stress|ROS Scavenging	Oxidative Stress-Spermidine|Putrescine Metabolism	paiA->PGPT0007785	10
Indirect Effects	StressControl|Biocontrol	Neutralizing ABiotic Stress	Nitrosative|Oxidative Stress|ROS Scavenging	Oxidative Stress-Spermidine|Putrescine Metabolism	patA1->PGPT0007155	1
Indirect Effects	StressControl|Biocontrol	Neutralizing ABiotic Stress	Nitrosative|Oxidative Stress|ROS Scavenging	Oxidative Stress-Spermidine|Putrescine Metabolism	potA->PGPT0007840	1
Indirect Effects	StressControl|Biocontrol	Neutralizing ABiotic Stress	Nitrosative|Oxidative Stress|ROS Scavenging	Oxidative Stress-Spermidine|Putrescine Metabolism	potB->PGPT0007835	1
Indirect Effects	StressControl|Biocontrol	Neutralizing ABiotic Stress	Nitrosative|Oxidative Stress|ROS Scavenging	Oxidative Stress-Spermidine|Putrescine Metabolism	potC->PGPT0007830	1
Indirect Effects	StressControl|Biocontrol	Neutralizing ABiotic Stress	Nitrosative|Oxidative Stress|ROS Scavenging	Oxidative Stress-Spermidine|Putrescine Metabolism	potD->PGPT0007825	1
Indirect Effects	StressControl|Biocontrol	Neutralizing ABiotic Stress	Nitrosative|Oxidative Stress|ROS Scavenging	Oxidative Stress-Spermidine|Putrescine Metabolism	puuP->PGPT0007795	4
Indirect Effects	StressControl|Biocontrol	Neutralizing ABiotic Stress	Nitrosative|Oxidative Stress|ROS Scavenging	Oxidative Stress-Spermidine|Putrescine Metabolism	speB->PGPT0007775	3
Indirect Effects	StressControl|Bioco	Neutralizing ABiotic Stress	Nitrosative|Oxidative Stress|ROS	Oxidative Stress-Spermidine|Putrescin	speE|SRM|SPEC3|SPSD-	3

	ntrol		Scavenging	e Metabolism	>PGPT0007750	
Indirect Effects	StressControl|Biocontrol	Neutralizing ABiotic Stress	Nitrosative|Oxidative Stress|ROS Scavenging	Oxidative Stress-Spermidine|Putrescine Metabolism	speG->PGPT0007790	6
Indirect Effects	StressControl|Biocontrol	Neutralizing ABiotic Stress	Nitrosative|Oxidative Stress|ROS Scavenging	Oxidative Stress-Sulfoxide Reductases	msrA->PGPT0013065	1
Indirect Effects	StressControl|Biocontrol	Neutralizing ABiotic Stress	Nitrosative|Oxidative Stress|ROS Scavenging	Oxidative Stress-Terpenoid Derivate Production	crtE|ispA->PGPT0007560	1
Indirect Effects	StressControl|Biocontrol	Neutralizing ABiotic Stress	Nitrosative|Oxidative Stress|ROS Scavenging	Oxidative Stress-Terpenoid Derivate Production	dxs->PGPT0008960	1
Indirect Effects	StressControl|Biocontrol	Neutralizing ABiotic Stress	Nitrosative|Oxidative Stress|ROS Scavenging	Oxidative Stress-Terpenoid Derivate Production	gcpE|ispG->PGPT0007580	1
Indirect Effects	StressControl|Biocontrol	Neutralizing ABiotic Stress	Nitrosative|Oxidative Stress|ROS Scavenging	Oxidative Stress-Terpenoid Derivate Production	hepST->PGPT0007531	1
Indirect Effects	StressControl|Biocontrol	Neutralizing ABiotic Stress	Nitrosative|Oxidative Stress|ROS Scavenging	Oxidative Stress-Terpenoid Derivate Production	idi->PGPT0007530	1
Indirect Effects	StressControl|Biocontrol	Neutralizing ABiotic Stress	Nitrosative|Oxidative Stress|ROS Scavenging	Oxidative Stress-Terpenoid Derivate Production	ispD->PGPT0007590	1
Indirect Effects	StressControl|Biocontrol	Neutralizing ABiotic Stress	Nitrosative|Oxidative Stress|ROS Scavenging	Oxidative Stress-Terpenoid Derivate Production	ispE->PGPT0007605	1
Indirect Effects	StressControl|Biocontrol	Neutralizing ABiotic Stress	Nitrosative|Oxidative Stress|ROS Scavenging	Oxidative Stress-Terpenoid Derivate Production	ispF->PGPT0007595	2
Indirect	StressCon	Neutralizing ABiotic	Nitrosative|Oxidative	Oxidative Stress-	ispH|lytB-	2

Effects	trol|Biocontrol	Stress	Stress|ROS Scavenging	Terpenoid Derivate Production	>PGPT0007615	
Indirect Effects	StressControl|Biocontrol	Neutralizing ABiotic Stress	Nitrosative|Oxidative Stress|ROS Scavenging	Oxidative Stress-Terpenoid Derivate Production	sphC->PGPT0007620	1
Indirect Effects	StressControl|Biocontrol	Neutralizing ABiotic Stress	Nitrosative|Oxidative Stress|ROS Scavenging	Oxidative Stress-Thioredoxines|Thioesterases	menI|ydiI|ydiL|ydiI|ybdB->PGPT0001845	1
Indirect Effects	StressControl|Biocontrol	Neutralizing ABiotic Stress	Nitrosative|Oxidative Stress|ROS Scavenging	Oxidative Stress-Thioredoxines|Thioesterases	trxA->PGPT0013055	3
Indirect Effects	StressControl|Biocontrol	Neutralizing ABiotic Stress	Nitrosative|Oxidative Stress|ROS Scavenging	Oxidative Stress-Thioredoxines|Thioesterases	trxB->PGPT0013060	6
Indirect Effects	StressControl|Biocontrol	Neutralizing ABiotic Stress	Nitrosative|Oxidative Stress|ROS Scavenging	Oxidative Stress-Thioredoxines|Thioesterases	yusE->PGPT0013063	1
Indirect Effects	StressControl|Biocontrol	Neutralizing ABiotic Stress	Nitrosative|Oxidative Stress|ROS Scavenging	Oxidative Stress-Ubiquinone|Coenzyme Q Pathway	ubiE->PGPT0009535	1
Indirect Effects	StressControl|Biocontrol	Neutralizing ABiotic Stress	Nitrosative|Oxidative Stress|ROS Scavenging	Oxidative Stress-Ubiquinone|Coenzyme Q Pathway	ubiX|bsdB->PGPT0009565	1
Indirect Effects	StressControl|Biocontrol	Neutralizing ABiotic Stress	Nitrosative|Oxidative Stress|ROS Scavenging	Oxidative Stress-Vitamin B3|Niacin Biosynthesis	deoD->PGPT0013385	1
Indirect Effects	StressControl|Biocontrol	Neutralizing ABiotic Stress	Nitrosative|Oxidative Stress|ROS Scavenging	Oxidative Stress-Vitamin B3|Niacin Biosynthesis	iunH->PGPT0013465	2
Indirect Effects	StressControl|Biocontrol	Neutralizing ABiotic Stress	Nitrosative|Oxidative Stress|ROS Scavenging	Oxidative Stress-Vitamin B3|Niacin Biosynthesis	nadA->PGPT0013365	1

Indirect Effects	StressControl|Biocontrol	Neutralizing ABiotic Stress	Nitrosative|Oxidative Stress|ROS Scavenging	Oxidative Stress-Vitamin B3|Niacin Biosynthesis	nadB->PGPT0013355	1
Indirect Effects	StressControl|Biocontrol	Neutralizing ABiotic Stress	Nitrosative|Oxidative Stress|ROS Scavenging	Oxidative Stress-Vitamin B3|Niacin Biosynthesis	nadC->PGPT0013370	1
Indirect Effects	StressControl|Biocontrol	Neutralizing ABiotic Stress	Nitrosative|Oxidative Stress|ROS Scavenging	Oxidative Stress-Vitamin B3|Niacin Biosynthesis	nadD->PGPT0013435	3
Indirect Effects	StressControl|Biocontrol	Neutralizing ABiotic Stress	Nitrosative|Oxidative Stress|ROS Scavenging	Oxidative Stress-Vitamin B3|Niacin Biosynthesis	nadE->PGPT0013445	2
Indirect Effects	StressControl|Biocontrol	Neutralizing ABiotic Stress	Nitrosative|Oxidative Stress|ROS Scavenging	Oxidative Stress-Vitamin B3|Niacin Biosynthesis	npdA->PGPT0013485	1
Indirect Effects	StressControl|Biocontrol	Neutralizing ABiotic Stress	Nitrosative|Oxidative Stress|ROS Scavenging	Oxidative Stress-Vitamin B3|Niacin Biosynthesis	pncA->PGPT0013470	2
Indirect Effects	StressControl|Biocontrol	Neutralizing ABiotic Stress	Nitrosative|Oxidative Stress|ROS Scavenging	Oxidative Stress-Vitamin B3|Niacin Biosynthesis	pncB->PGPT0013375	2
Indirect Effects	StressControl|Biocontrol	Neutralizing ABiotic Stress	Nitrosative|Oxidative Stress|ROS Scavenging	Oxidative Stress-Vitamin B3|Niacin Biosynthesis	pncC->PGPT0013455	1
Indirect Effects	StressControl|Biocontrol	Neutralizing ABiotic Stress	Nitrosative|Oxidative Stress|ROS Scavenging	Oxidative Stress-Vitamin B3|Niacin Biosynthesis	punA->PGPT0013380	1
Indirect Effects	StressControl|Biocontrol	Neutralizing ABiotic Stress	Nitrosative|Oxidative Stress|ROS Scavenging	Oxidative Stress-Vitamin B3|Niacin Biosynthesis	yfkN->PGPT0013420	1
Indirect Effects	StressControl|Bioco	Neutralizing ABiotic Stress	Nitrosative|Oxidative Stress|ROS	Oxidative Stress-Vitamin	pdxA->PGPT0009165	1

	ntrol		Scavenging	B6|Pyridoxine|Pyridoxal|PyridoxAmine Biosynthesis		
Indirect Effects	StressControl|Biocontrol	Neutralizing ABiotic Stress	Nitrosative|Oxidative Stress|ROS Scavenging	Oxidative Stress-Vitamin B6|Pyridoxine|Pyridoxal|PyridoxAmine Biosynthesis	pdxK|pdxY->PGPT0009125	1
Indirect Effects	StressControl|Biocontrol	Neutralizing ABiotic Stress	Nitrosative|Oxidative Stress|ROS Scavenging	Oxidative Stress-Vitamin B6|Pyridoxine|Pyridoxal|PyridoxAmine Biosynthesis	pdxS|pdx1|yaaD->PGPT0009180	2
Indirect Effects	StressControl|Biocontrol	Neutralizing ABiotic Stress	Nitrosative|Oxidative Stress|ROS Scavenging	Oxidative Stress-Vitamin B6|Pyridoxine|Pyridoxal|PyridoxAmine Biosynthesis	pdxT|pdx2|yaaE->PGPT0009185	2
Indirect Effects	StressControl|Biocontrol	Neutralizing ABiotic Stress	Nitrosative|Oxidative Stress|ROS Scavenging	Oxidative Stress-Vitamin B6|Pyridoxine|Pyridoxal|PyridoxAmine Biosynthesis	serA->PGPT0009155	2
Indirect Effects	StressControl|Biocontrol	Neutralizing ABiotic Stress	Nitrosative|Oxidative Stress|ROS Scavenging	Oxidative Stress-Vitamin B6|Pyridoxine|Pyridoxal|PyridoxAmine Biosynthesis	serC|pdxF->PGPT0009160	1
Indirect Effects	StressControl|Biocontrol	Neutralizing ABiotic Stress	Nitrosative|Oxidative Stress|ROS Scavenging	Oxidative Stress-Vitamin B6|Pyridoxine|Pyridoxal|PyridoxAmine	thrC->PGPT0009175	5

				Biosynthesis		
Indirect Effects	StressControl|Biocontrol	Neutralizing ABiotic Stress	Nitrosative|Oxidative Stress|ROS Scavenging	Oxidative Stress-Vitamin C|Ascorbic Acid Biosynthesis	dkgA->PGPT0001320	3
Indirect Effects	StressControl|Biocontrol	Neutralizing Biotic Stress	Antiprotozoan|Antiprotistal Activity	Antiprotistal-Alkyresorcinol|Alkypyrone Biosynthesis	bpsA|srsA|->PGPT0011290	1
Indirect Effects	StressControl|Biocontrol	Neutralizing Biotic Stress	Antiprotozoan|Antiprotistal Activity	Antiprotistal-Alkyresorcinol|Alkypyrone Biosynthesis	bpsB|srsB->PGPT0011295	1
Indirect Effects	StressControl|Biocontrol	Neutralizing Biotic Stress	Bactericidal Compounds|Antibiotics	Bactericidal-Alkyresorcinol|AlkypyroneS Metabolism	bpsA|srsA|->PGPT0011290	1
Indirect Effects	StressControl|Biocontrol	Neutralizing Biotic Stress	Bactericidal Compounds|Antibiotics	Bactericidal-Alkyresorcinol|AlkypyroneS Metabolism	bpsB|srsB->PGPT0011295	1
Indirect Effects	StressControl|Biocontrol	Neutralizing Biotic Stress	Bactericidal Compounds|Antibiotics	Bactericidal-Ansamitocin|Rifamycin Metabolism	tktA|tktB->PGPT0011200	4
Indirect Effects	StressControl|Biocontrol	Neutralizing Biotic Stress	Bactericidal Compounds|Antibiotics	Bactericidal-Antibiotic Phosphonate Substances	bcpC|aepY->PGPT0011310	1
Indirect Effects	StressControl|Biocontrol	Neutralizing Biotic Stress	Bactericidal Compounds|Antibiotics	Bactericidal-Antibiotic Phosphonate Substances	pepM|aepX->PGPT0011300	1
Indirect Effects	StressControl|Biocontrol	Neutralizing Biotic Stress	Bactericidal Compounds|Antibiotics	Bactericidal-Antibiotic Phosphonate Substances	phnW->PGPT0002485	1
Indirect Effects	StressControl|Biocontrol	Neutralizing Biotic Stress	Bactericidal Compounds|Antibiotics	Bactericidal-Aurachin Metabolism	acpP->PGPT0011375	2
Indirect	StressCon	Neutralizing Biotic	Bactericidal	Bactericidal-Aurachin	auaJ->PGPT0011400	1

Effects	trol|Biocontrol	Stress	Compounds|Antibiotics	Metabolism		
Indirect Effects	StressControl|Biocontrol	Neutralizing Biotic Stress	Bactericidal Compounds|Antibiotics	Bactericidal-Bacillaene Metabolism	baeS|pksS->PGPT0010175	3
Indirect Effects	StressControl|Biocontrol	Neutralizing Biotic Stress	Bactericidal Compounds|Antibiotics	Bactericidal-Bacitracin A Metabolism	bceA|vraD->PGPT0010200	2
Indirect Effects	StressControl|Biocontrol	Neutralizing Biotic Stress	Bactericidal Compounds|Antibiotics	Bactericidal-Bacitracin A Metabolism	bceR->PGPT0010230	1
Indirect Effects	StressControl|Biocontrol	Neutralizing Biotic Stress	Bactericidal Compounds|Antibiotics	Bactericidal-Bacitracin A Metabolism	bcsS->PGPT0010225	1
Indirect Effects	StressControl|Biocontrol	Neutralizing Biotic Stress	Bactericidal Compounds|Antibiotics	Bactericidal-Bacteriocins|Colicins	cvpA->PGPT0011655	1
Indirect Effects	StressControl|Biocontrol	Neutralizing Biotic Stress	Bactericidal Compounds|Antibiotics	Bactericidal-Bacteriocins|Lantibiotic|Nisin	lanM|cylM->PGPT0011680	1
Indirect Effects	StressControl|Biocontrol	Neutralizing Biotic Stress	Bactericidal Compounds|Antibiotics	Bactericidal-Bacteriocins|Lantibiotic|Nisin	nisK|spaK->PGPT0011705	2
Indirect Effects	StressControl|Biocontrol	Neutralizing Biotic Stress	Bactericidal Compounds|Antibiotics	Bactericidal-Bacteriocins|Lantibiotic|Nisin	nisR|spaR->PGPT0011710	1
Indirect Effects	StressControl|Biocontrol	Neutralizing Biotic Stress	Bactericidal Compounds|Antibiotics	Bactericidal-Bacteriocins|Lantibiotic|Nisin	nprR|nprA->PGPT0011700	3
Indirect Effects	StressControl|Biocontrol	Neutralizing Biotic Stress	Bactericidal Compounds|Antibiotics	Bactericidal-Bacteriocins|Lantibiotic|Nisin	nukE|mcdE|sboE->PGPT0011720	2

Indirect Effects	StressControl|Biocontrol	Neutralizing Biotic Stress	Bactericidal Compounds|Antibiotics	Bactericidal-Bacteriocins|Lantibiotic|Nisin	nukF|mcdF|sbOf->PGPT0011725	2
Indirect Effects	StressControl|Biocontrol	Neutralizing Biotic Stress	Bactericidal Compounds|Antibiotics	Bactericidal-Bacteriocins|Lantibiotic|Nisin	nukG|mcdG|sboG->PGPT0011715	2
Indirect Effects	StressControl|Biocontrol	Neutralizing Biotic Stress	Bactericidal Compounds|Antibiotics	Bactericidal-Bacteriocins|Lantibiotic|Nisin	rseP->PGPT0011685	1
Indirect Effects	StressControl|Biocontrol	Neutralizing Biotic Stress	Bactericidal Compounds|Antibiotics	Bactericidal-Bacteriocins|Lantibiotic|Nisin	ybhF|yadG|ecsA->PGPT0006725	14
Indirect Effects	StressControl|Biocontrol	Neutralizing Biotic Stress	Bactericidal Compounds|Antibiotics	Bactericidal-Bacteriocins|Lantibiotic|Nisin	ybhS|ecsB->PGPT0006730	6
Indirect Effects	StressControl|Biocontrol	Neutralizing Biotic Stress	Bactericidal Compounds|Antibiotics	Bactericidal-Butirosin Metabolism	btrF->PGPT0010015	1
Indirect Effects	StressControl|Biocontrol	Neutralizing Biotic Stress	Bactericidal Compounds|Antibiotics	Bactericidal-Cycloserine Metabolism	dcsA->PGPT0011510	1
Indirect Effects	StressControl|Biocontrol	Neutralizing Biotic Stress	Bactericidal Compounds|Antibiotics	Bactericidal-Erythromycin Metabolism	eryBII->PGPT0010855	1
Indirect Effects	StressControl|Biocontrol	Neutralizing Biotic Stress	Bactericidal Compounds|Antibiotics	Bactericidal-Phosphomycin Biosynthesis	bcpC|aepY->PGPT0011310	1
Indirect Effects	StressControl|Biocontrol	Neutralizing Biotic Stress	Bactericidal Compounds|Antibiotics	Bactericidal-Phosphomycin Biosynthesis	pepM|aepX->PGPT0011300	1
Indirect Effects	StressControl|Bioco	Neutralizing Biotic Stress	Bactericidal Compounds|Antibioti	Bactericidal-Oxa-|Avermectin	aveBI->PGPT0011160	1

	ntrol		cs	Metabolism		
Indirect Effects	StressControl|Biocontrol	Neutralizing Biotic Stress	Bactericidal Compounds|Antibiotics	Bactericidal-Polymyxin Metabolism	pmrA->PGPT0003915	3
Indirect Effects	StressControl|Biocontrol	Neutralizing Biotic Stress	Bactericidal Compounds|Antibiotics	Bactericidal-Prodigiosin Metabolism	fabD|bmyD->PGPT0008350	1
Indirect Effects	StressControl|Biocontrol	Neutralizing Biotic Stress	Bactericidal Compounds|Antibiotics	Bactericidal-Prodigiosin Metabolism	fabI->PGPT0008370	1
Indirect Effects	StressControl|Biocontrol	Neutralizing Biotic Stress	Bactericidal Compounds|Antibiotics	Bactericidal-Prodigiosin Metabolism	redL->PGPT0011615	1
Indirect Effects	StressControl|Biocontrol	Neutralizing Biotic Stress	Bactericidal Compounds|Antibiotics	Bactericidal-Prodigiosin Metabolism	ymfI|fabG|efpI->PGPT0003180	14
Indirect Effects	StressControl|Biocontrol	Neutralizing Biotic Stress	Bactericidal Compounds|Antibiotics	Bactericidal-Spermidine|Putrescine Metabolism	gsp->PGPT0007755	1
Indirect Effects	StressControl|Biocontrol	Neutralizing Biotic Stress	Bactericidal Compounds|Antibiotics	Bactericidal-Spermidine|Putrescine Metabolism	paiA->PGPT0007785	10
Indirect Effects	StressControl|Biocontrol	Neutralizing Biotic Stress	Bactericidal Compounds|Antibiotics	Bactericidal-Spermidine|Putrescine Metabolism	patA1->PGPT0007155	1
Indirect Effects	StressControl|Biocontrol	Neutralizing Biotic Stress	Bactericidal Compounds|Antibiotics	Bactericidal-Spermidine|Putrescine Metabolism	potA->PGPT0007840	1
Indirect Effects	StressControl|Biocontrol	Neutralizing Biotic Stress	Bactericidal Compounds|Antibiotics	Bactericidal-Spermidine|Putrescine Metabolism	potB->PGPT0007835	1
Indirect	StressCon	Neutralizing Biotic	Bactericidal	Bactericidal-	potC->PGPT0007830	1

Effects	trol|Biocontrol	Stress	Compounds|Antibiotics	Spermidine|Putrescine Metabolism		
Indirect Effects	StressControl|Biocontrol	Neutralizing Biotic Stress	Bactericidal Compounds|Antibiotics	Bactericidal-Spermidine|Putrescine Metabolism	potD->PGPT0007825	1
Indirect Effects	StressControl|Biocontrol	Neutralizing Biotic Stress	Bactericidal Compounds|Antibiotics	Bactericidal-Spermidine|Putrescine Metabolism	puuP->PGPT0007795	4
Indirect Effects	StressControl|Biocontrol	Neutralizing Biotic Stress	Bactericidal Compounds|Antibiotics	Bactericidal-Spermidine|Putrescine Metabolism	speB->PGPT0007775	3
Indirect Effects	StressControl|Biocontrol	Neutralizing Biotic Stress	Bactericidal Compounds|Antibiotics	Bactericidal-Spermidine|Putrescine Metabolism	speE|SRM|SPEC3|SPSD->PGPT0007750	3
Indirect Effects	StressControl|Biocontrol	Neutralizing Biotic Stress	Bactericidal Compounds|Antibiotics	Bactericidal-Spermidine|Putrescine Metabolism	speG->PGPT0007790	6
Indirect Effects	StressControl|Biocontrol	Neutralizing Biotic Stress	Bactericidal Compounds|Antibiotics	Bactericidal-Toxoflavin Metabolism	ribA->PGPT0007985	1
Indirect Effects	StressControl|Biocontrol	Neutralizing Biotic Stress	Bactericidal Compounds|Antibiotics	Bactericidal-Toxoflavin Metabolism	ribD->PGPT0008555	2
Indirect Effects	StressControl|Biocontrol	Neutralizing Biotic Stress	Bactericidal Compounds|Antibiotics	Bactericidal-Toxoflavin Metabolism	ToxF->PGPT0009795	3
Indirect Effects	StressControl|Biocontrol	Neutralizing Biotic Stress	Bactericidal Compounds|Antibiotics	Bactericidal-Toxoflavin Metabolism	ToxG|mexH->PGPT0009800	1
Indirect Effects	StressControl|Biocontrol	Neutralizing Biotic Stress	Bactericidal Compounds|Antibiotics	Bactericidal-Type II Polyketide-Actinorhodin|Meder	actVA6->PGPT0010305	1

				mycin|Dihydrokalafungin Metabolism		
Indirect Effects	StressControl|Biocontrol	Neutralizing Biotic Stress	Bactericidal Compounds|Antibiotics	Bactericidal-Type II Polyketide-Aklavinone-Aclacinomycin Metabolism	rdmC->PGPT0010490	1
Indirect Effects	StressControl|Biocontrol	Neutralizing Biotic Stress	Bactericidal Compounds|Antibiotics	Bactericidal-Type II Polyketide-Aklavinone-rhodomycin Metabolism	rdmC->PGPT0010490	1
Indirect Effects	StressControl|Biocontrol	Neutralizing Biotic Stress	Bactericidal Compounds|Antibiotics	Bactericidal-Type II Polyketide-Oxy-|Chlor-|Tetracycline Metabolism	otrA|tetM|teTo->PGPT0010355	3
Indirect Effects	StressControl|Biocontrol	Neutralizing Biotic Stress	Bactericidal Compounds|Antibiotics	Bactericidal-Type II Polyketide-Oxy-|Chlor-|Tetracycline Metabolism	oxyE->PGPT0010330	1
Indirect Effects	StressControl|Biocontrol	Neutralizing Biotic Stress	Bactericidal Compounds|Antibiotics	Bactericidal-Type II Polyketide-Oxy-|Chlor-|Tetracycline Metabolism	oxyL->PGPT0010325	1
Indirect Effects	StressControl|Biocontrol	Neutralizing Biotic Stress	Bactericidal Compounds|Antibiotics	Bactericidal-Type II Polyketide-Tetracenomycin Metabolism	tcmG|elmG->PGPT0010600	1
Indirect Effects	StressControl|Biocontrol	Neutralizing Biotic Stress	Bactericidal Compounds|Antibiotics	Bactericidal-Xylostasin Metabolism	btrF->PGPT0010015	1
Indirect	StressCon	Neutralizing Biotic	Biotic Stress	Biotic Stress	aroA->PGPT0012865	1

Effects	trol|Biocontrol	Stress	Resistance-Phenazine Derivates	Resistance-Phenazine-1-Carboxylic Acid Biosynthesis		
Indirect Effects	StressControl|Biocontrol	Neutralizing Biotic Stress	Biotic Stress Resistance-Phenazine Derivates	Biotic Stress Resistance-Phenazine-1-Carboxylic Acid Biosynthesis	aroB->PGPT0012875	2
Indirect Effects	StressControl|Biocontrol	Neutralizing Biotic Stress	Biotic Stress Resistance-Phenazine Derivates	Biotic Stress Resistance-Phenazine-1-Carboxylic Acid Biosynthesis	aRod->PGPT0012885	1
Indirect Effects	StressControl|Biocontrol	Neutralizing Biotic Stress	Biotic Stress Resistance-Phenazine Derivates	Biotic Stress Resistance-Phenazine-1-Carboxylic Acid Biosynthesis	aroE->PGPT0012890	1
Indirect Effects	StressControl|Biocontrol	Neutralizing Biotic Stress	Biotic Stress Resistance-Phenazine Derivates	Biotic Stress Resistance-Phenazine-1-Carboxylic Acid Biosynthesis	arOf->PGPT0012910	2
Indirect Effects	StressControl|Biocontrol	Neutralizing Biotic Stress	Biotic Stress Resistance-Phenazine Derivates	Biotic Stress Resistance-Phenazine-1-Carboxylic Acid Biosynthesis	aroG->PGPT0012915	2
Indirect Effects	StressControl|Biocontrol	Neutralizing Biotic Stress	Biotic Stress Resistance-Phenazine Derivates	Biotic Stress Resistance-Phenazine-1-Carboxylic Acid Biosynthesis	aroL|aroK->PGPT0012900	1
Indirect Effects	StressControl|Biocontrol	Neutralizing Biotic Stress	Biotic Stress Resistance-Phenazine Derivates	Biotic Stress Resistance-Phenazine-1-Carboxylic Acid Biosynthesis	aroQ|qutE->PGPT0012905	1
Indirect	StressCon	Neutralizing Biotic	Biotic Stress	Biotic Stress	cyclohexadieny|preph	1

Effects	trol|Biocontrol	Stress	Resistance-Phenazine Derivates	Resistance-Phenazine-1-Carboxylic Acid Biosynthesis	enate Dehydrogenase|3 PhosphoShikimate 1 carboxyvinylTransferase->PGPT0012870	
Indirect Effects	StressControl|Biocontrol	Neutralizing Biotic Stress	Biotic Stress Resistance-Phenazine Derivates	Biotic Stress Resistance-Phenazine Biosynthesis	phzF|yddE->PGPT0012805	2
Indirect Effects	StressControl|Biocontrol	Neutralizing Biotic Stress	Biotic Stress Resistance-Phenazine Derivates	Biotic Stress Resistance-Phenazine Biosynthesis	trpE|phnA->PGPT0007095	3
Indirect Effects	StressControl|Biocontrol	Neutralizing Biotic Stress	Biotic Stress Resistance-Phenazine Derivates	Biotic Stress Resistance-Phenazine Biosynthesis	yddE->PGPT0012850	2
Indirect Effects	StressControl|Biocontrol	Neutralizing Biotic Stress	Biotic Stress Resistance-Volatiles	Biotic Stress Resistance-1|2-Propanediol Volatile Biosynthesis	gldA|dhaD->PGPT0008270	1
Indirect Effects	StressControl|Biocontrol	Neutralizing Biotic Stress	Biotic Stress Resistance-Volatiles	Biotic Stress Resistance-Acetoin|2|3-Butanediol Volatile Biosynthesis	aceF|pdhC->PGPT0001390	2
Indirect Effects	StressControl|Biocontrol	Neutralizing Biotic Stress	Biotic Stress Resistance-Volatiles	Biotic Stress Resistance-Acetoin|2|3-Butanediol Volatile Biosynthesis	acoA->PGPT0008230	1
Indirect Effects	StressControl|Biocontrol	Neutralizing Biotic Stress	Biotic Stress Resistance-Volatiles	Biotic Stress Resistance-Acetoin|2|3-Butanediol Volatile	acoB->PGPT0008235	1

				Biosynthesis		
Indirect Effects	StressControl|Biocontrol	Neutralizing Biotic Stress	Biotic Stress Resistance-Volatiles	Biotic Stress Resistance-Acetoin|2|3-Butanediol Volatile Biosynthesis	acoR->PGPT0001030	6
Indirect Effects	StressControl|Biocontrol	Neutralizing Biotic Stress	Biotic Stress Resistance-Volatiles	Biotic Stress Resistance-Acetoin|2|3-Butanediol Volatile Biosynthesis	acuB->PGPT0008220	4
Indirect Effects	StressControl|Biocontrol	Neutralizing Biotic Stress	Biotic Stress Resistance-Volatiles	Biotic Stress Resistance-Acetoin|2|3-Butanediol Volatile Biosynthesis	acuC->PGPT0008225	1
Indirect Effects	StressControl|Biocontrol	Neutralizing Biotic Stress	Biotic Stress Resistance-Volatiles	Biotic Stress Resistance-Acetoin|2|3-Butanediol Volatile Biosynthesis	budA|aldC|aldB|alsD->PGPT0008180	1
Indirect Effects	StressControl|Biocontrol	Neutralizing Biotic Stress	Biotic Stress Resistance-Volatiles	Biotic Stress Resistance-Acetoin|2|3-Butanediol Volatile Biosynthesis	budB|ilvK|alsS|ilvB|ilvG|ilvI->PGPT0008185	4
Indirect Effects	StressControl|Biocontrol	Neutralizing Biotic Stress	Biotic Stress Resistance-Volatiles	Biotic Stress Resistance-Acetoin|2|3-Butanediol Volatile Biosynthesis	budC->PGPT0008190	3
Indirect	StressCon	Neutralizing Biotic	Biotic Stress	Biotic Stress	butA|ydjL|budC-	1

Effects	trol|Biocontrol	Stress	Resistance-Volatiles	Resistance-Acetoin|2|3-Butanediol Volatile Biosynthesis	>PGPT0008195	
Indirect Effects	StressControl|Biocontrol	Neutralizing Biotic Stress	Biotic Stress Resistance-Volatiles	Biotic Stress Resistance-Acetoin|2|3-Butanediol Volatile Biosynthesis	butB->PGPT0008200	1
Indirect Effects	StressControl|Biocontrol	Neutralizing Biotic Stress	Biotic Stress Resistance-Volatiles	Biotic Stress Resistance-Acetoin|2|3-Butanediol Volatile Biosynthesis	ilvH|ilvN->PGPT0008205	1
Indirect Effects	StressControl|Biocontrol	Neutralizing Biotic Stress	Biotic Stress Resistance-Volatiles	Biotic Stress Resistance-Acetoin|2|3-Butanediol Volatile Biosynthesis	lpd|pdhD->PGPT0001380	3
Indirect Effects	StressControl|Biocontrol	Neutralizing Biotic Stress	Biotic Stress Resistance-Volatiles	Biotic Stress Resistance-Alcohol|Ketone Volatile Metabolism	OXCT Like->PGPT0008335	1
Indirect Effects	StressControl|Biocontrol	Neutralizing Biotic Stress	Biotic Stress Resistance-Volatiles	Biotic Stress Resistance-Alcohol|Ketone Volatile Metabolism	adh1->PGPT0008305	2
Indirect Effects	StressControl|Biocontrol	Neutralizing Biotic Stress	Biotic Stress Resistance-Volatiles	Biotic Stress Resistance-Alcohol|Ketone Volatile Metabolism	adh2 2->PGPT0008295	3
Indirect	StressCon	Neutralizing Biotic	Biotic Stress	Biotic Stress	bdh->PGPT0008310	2

Effects	trol|Biocontrol	Stress	Resistance-Volatiles	Resistance-Alcohol|Ketone Volatile Metabolism		
Indirect Effects	StressControl|Biocontrol	Neutralizing Biotic Stress	Biotic Stress Resistance-Volatiles	Biotic Stress Resistance-Alcohol|Ketone Volatile Metabolism	gbsB->PGPT0008300	1
Indirect Effects	StressControl|Biocontrol	Neutralizing Biotic Stress	Biotic Stress Resistance-Volatiles	Biotic Stress Resistance-Alcohol|Ketone Volatile Metabolism	hmgL->PGPT0008315	2
Indirect Effects	StressControl|Biocontrol	Neutralizing Biotic Stress	Biotic Stress Resistance-Volatiles	Biotic Stress Resistance-Alcohol|Ketone Volatile Metabolism	scoA->PGPT0008325	2
Indirect Effects	StressControl|Biocontrol	Neutralizing Biotic Stress	Biotic Stress Resistance-Volatiles	Biotic Stress Resistance-Alcohol|Ketone Volatile Metabolism	scoB->PGPT0008330	1
Indirect Effects	StressControl|Biocontrol	Neutralizing Biotic Stress	Biotic Stress Resistance-Volatiles	Biotic Stress Resistance-Volatile Related Fatty Acid Metabolism	accA->PGPT0001695	1
Indirect Effects	StressControl|Biocontrol	Neutralizing Biotic Stress	Biotic Stress Resistance-Volatiles	Biotic Stress Resistance-Volatile Related Fatty Acid Metabolism	accB|bccP->PGPT0001700	3
Indirect Effects	StressControl|Biocontrol	Neutralizing Biotic Stress	Biotic Stress Resistance-Volatiles	Biotic Stress Resistance-Volatile Related Fatty Acid Metabolism	accC->PGPT0001705	3
Indirect	StressCon	Neutralizing Biotic	Biotic Stress	Biotic Stress	accD->PGPT0001710	1

Effects	trol|Biocontrol	Stress	Resistance-Volatiles	Resistance-Volatile Related Fatty Acid Metabolism		
Indirect Effects	StressControl|Biocontrol	Neutralizing Biotic Stress	Biotic Stress Resistance-Volatiles	Biotic Stress Resistance-Volatile Related Fatty Acid Metabolism	acd->PGPT0008385	2
Indirect Effects	StressControl|Biocontrol	Neutralizing Biotic Stress	Biotic Stress Resistance-Volatiles	Biotic Stress Resistance-Volatile Related Fatty Acid Metabolism	desA->PGPT0008400	2
Indirect Effects	StressControl|Biocontrol	Neutralizing Biotic Stress	Biotic Stress Resistance-Volatiles	Biotic Stress Resistance-Volatile Related Fatty Acid Metabolism	fabD|bmyD->PGPT0008350	1
Indirect Effects	StressControl|Biocontrol	Neutralizing Biotic Stress	Biotic Stress Resistance-Volatiles	Biotic Stress Resistance-Volatile Related Fatty Acid Metabolism	fabF->PGPT0008360	1
Indirect Effects	StressControl|Biocontrol	Neutralizing Biotic Stress	Biotic Stress Resistance-Volatiles	Biotic Stress Resistance-Volatile Related Fatty Acid Metabolism	fabH->PGPT0008355	4
Indirect Effects	StressControl|Biocontrol	Neutralizing Biotic Stress	Biotic Stress Resistance-Volatiles	Biotic Stress Resistance-Volatile Related Fatty Acid Metabolism	fabI->PGPT0008370	1
Indirect Effects	StressControl|Biocontrol	Neutralizing Biotic Stress	Biotic Stress Resistance-Volatiles	Biotic Stress Resistance-Volatile Related Fatty Acid Metabolism	fabL->PGPT0008375	1
Indirect	StressCon	Neutralizing Biotic	Biotic Stress	Biotic Stress	fabZ->PGPT0008365	3

Effects	trol|Biocontrol	Stress	Resistance-Volatiles	Resistance-Volatile Related Fatty Acid Metabolism		
Indirect Effects	StressControl|Biocontrol	Neutralizing Biotic Stress	Biotic Stress Resistance-Volatiles	Biotic Stress Resistance-Volatile Related Fatty Acid Metabolism	fadB->PGPT0008390	2
Indirect Effects	StressControl|Biocontrol	Neutralizing Biotic Stress	Biotic Stress Resistance-Volatiles	Biotic Stress Resistance-Volatile Related Fatty Acid Metabolism	fadD->PGPT0008380	6
Indirect Effects	StressControl|Biocontrol	Neutralizing Biotic Stress	Biotic Stress Resistance-Volatiles	Biotic Stress Resistance-Volatile Related Fatty Acid Metabolism	fadN->PGPT0008395	1
Indirect Effects	StressControl|Biocontrol	Neutralizing Biotic Stress	Biotic Stress Resistance-Volatiles	Biotic Stress Resistance-Volatile Related Fatty Acid Metabolism	ymfI|fabG|efpI->PGPT0003180	14
Indirect Effects	StressControl|Biocontrol	Neutralizing Biotic Stress	Fungicidal Compounds|Antibiotics	Fungicidal-Alkyresorcinol|Alkypyrone Metabolism	bpsA|srsA|->PGPT0011290	1
Indirect Effects	StressControl|Biocontrol	Neutralizing Biotic Stress	Fungicidal Compounds|Antibiotics	Fungicidal-Alkyresorcinol|Alkypyrone Metabolism	bpsB|srsB->PGPT0011295	1
Indirect Effects	StressControl|Biocontrol	Neutralizing Biotic Stress	Fungicidal Compounds|Antibiotics	Fungicidal-Ansamitocin P-3 Metabolism	asm17->PGPT0011950	1
Indirect Effects	StressControl|Biocontrol	Neutralizing Biotic Stress	Fungicidal Compounds|Antibiotics	Fungicidal-Ansamitocin|Rifamycin Metabolism	tktA|tktB->PGPT0011200	4
Indirect	StressCon	Neutralizing Biotic	Fungicidal	Fungicidal-	chitin deAcetylase-	2

Effects	trol|Biocontrol	Stress	Compounds|Antibiotics	Chitinolytic Activities	>PGPT0012156	
Indirect Effects	StressControl|Biocontrol	Neutralizing Biotic Stress	Fungicidal Compounds|Antibiotics	Fungicidal-Fungal Glycogen Degradation	pulA->PGPT0012195	2
Indirect Effects	StressControl|Biocontrol	Neutralizing Biotic Stress	Fungicidal Compounds|Antibiotics	Fungicidal-Motility-Mediated Defense Signaling	lysS->PGPT0012225	1
Indirect Effects	StressControl|Biocontrol	Neutralizing Biotic Stress	Fungicidal Compounds|Antibiotics	Fungicidal-Motility-Mediated Defense Signaling	yajQ->PGPT0012210	1
Indirect Effects	StressControl|Biocontrol	Neutralizing Biotic Stress	Fungicidal Compounds|Antibiotics	Fungicidal-Motility-Mediated Defense Signaling	ybgC->PGPT0001850	3
Indirect Effects	StressControl|Biocontrol	Neutralizing Biotic Stress	Fungicidal Compounds|Antibiotics	Fungicidal-Toxoflavin Metabolism	ribA->PGPT0007985	1
Indirect Effects	StressControl|Biocontrol	Neutralizing Biotic Stress	Fungicidal Compounds|Antibiotics	Fungicidal-Toxoflavin Metabolism	ribD->PGPT0008555	2
Indirect Effects	StressControl|Biocontrol	Neutralizing Biotic Stress	Fungicidal Compounds|Antibiotics	Fungicidal-Toxoflavin Metabolism	ToxF->PGPT0009795	3
Indirect Effects	StressControl|Biocontrol	Neutralizing Biotic Stress	Fungicidal Compounds|Antibiotics	Fungicidal-Toxoflavin Metabolism	ToxG|mexH->PGPT0009800	1
Indirect Effects	StressControl|Biocontrol	Neutralizing Biotic Stress	Insecticidal Compounds	Insecticidal-Gamma-Aminobutyric Acid Biosynthesis	gabP->PGPT0007665	4
Indirect Effects	StressControl|Biocontrol	Neutralizing Biotic Stress	Insecticidal Compounds	Insecticidal-Gamma-Aminobutyric Acid Biosynthesis	gabT->PGPT0007660	1

Indirect Effects	StressControl|Biocontrol	Neutralizing Biotic Stress	Insecticidal Compounds	Insecticidal-Gamma-Aminobutyric Acid Biosynthesis	gadA|gadB->PGPT0007630	1
Indirect Effects	StressControl|Biocontrol	Neutralizing Biotic Stress	Insecticidal Compounds	Insecticidal-Gamma-Aminobutyric Acid Biosynthesis	puuE->PGPT0007140	3
Indirect Effects	StressControl|Biocontrol	Neutralizing Biotic Stress	Other Biotic Stress Resistance Genes	Biotic Stress Resistance-Bacilysin Metabolism	bacC->PGPT0012770	1
Indirect Effects	StressControl|Biocontrol	Neutralizing Biotic Stress	Other Biotic Stress Resistance Genes	Biotic Stress Resistance-Bacilysin Metabolism	bacF->PGPT0012760	1
Indirect Effects	StressControl|Biocontrol	Neutralizing Biotic Stress	Other Biotic Stress Resistance Genes	Biotic Stress Resistance-Bacilysin Metabolism	degU->PGPT0012750	2
Indirect Effects	StressControl|Biocontrol	Neutralizing Biotic Stress	Other Biotic Stress Resistance Genes	Biotic Stress Resistance-Bacilysin Metabolism	hemQ->PGPT0008490	1
Indirect Effects	StressControl|Biocontrol	Neutralizing Biotic Stress	Other Biotic Stress Resistance Genes	Biotic Stress Resistance-Bacilysin Metabolism	scoC|hpr->PGPT0012755	3
Indirect Effects	StressControl|Biocontrol	Neutralizing Biotic Stress	Tumoricidal Compounds	Tumoricidal-Ansamitocin P-3 Metabolism	asm17->PGPT0011950	1
Indirect Effects	StressControl|Biocontrol	Neutralizing Biotic Stress	Tumoricidal Compounds	Tumoricidal-Type II Polyketide-Aklavinone-Aclacinomycin Metabolism	rdmC->PGPT0010490	1
Indirect Effects	StressControl|Biocontrol	Universal Stress Response	Stress Signaling Proteins	Other Stress Response Proteins	aldH|dhaS->PGPT0006875	5

Indirect Effects	StressControl|Biocontrol	Universal Stress Response	Stress Signaling Proteins	Other Stress Response Proteins	csbA->PGPT0015022	1
Indirect Effects	StressControl|Biocontrol	Universal Stress Response	Stress Signaling Proteins	Other Stress Response Proteins	csbB|gtrB|yfdH->PGPT0014540	4
Indirect Effects	StressControl|Biocontrol	Universal Stress Response	Stress Signaling Proteins	Other Stress Response Proteins	csbX->PGPT0015024	1
Indirect Effects	StressControl|Biocontrol	Universal Stress Response	Stress Signaling Proteins	Other Stress Response Proteins	dps|dpsA->PGPT0004055	2
Indirect Effects	StressControl|Biocontrol	Universal Stress Response	Stress Signaling Proteins	Other Stress Response Proteins	gabD->PGPT0001580	5
Indirect Effects	StressControl|Biocontrol	Universal Stress Response	Stress Signaling Proteins	Other Stress Response Proteins	ispD->PGPT0007590	1
Indirect Effects	StressControl|Biocontrol	Universal Stress Response	Stress Signaling Proteins	Other Stress Response Proteins	ispF->PGPT0007595	2
Indirect Effects	StressControl|Biocontrol	Universal Stress Response	Stress Signaling Proteins	Other Stress Response Proteins	prkA|yeaG->PGPT0014345	1
Indirect Effects	StressControl|Biocontrol	Universal Stress Response	Stress Signaling Proteins	Other Stress Response Proteins	pyrF->PGPT0014965	1
Indirect Effects	StressControl|Biocontrol	Universal Stress Response	Stress Signaling Proteins	Other Stress Response Proteins	sigM|rpoE->PGPT0015025	1
Indirect Effects	StressControl|Bioco	Universal Stress Response	Stress Signaling Proteins	Other Stress Response Proteins	ydaG->PGPT0015019	2

	ntrol					
Indirect Effects	StressControl|Biocontrol	Universal Stress Response	Stress Signaling Proteins	Other Stress Response Proteins	ydhK->PGPT0015018	1
Indirect Effects	StressControl|Biocontrol	Universal Stress Response	Stress Signaling Proteins	Other Stress Response Proteins	yfhM->PGPT0015015	2
Indirect Effects	StressControl|Biocontrol	Universal Stress Response	Stress Signaling Proteins	Other Stress Response Proteins	yfkH->PGPT0014525	3
Indirect Effects	StressControl|Biocontrol	Universal Stress Response	Stress Signaling Proteins	Other Stress Response Proteins	yfkM|pfpI|yraA->PGPT0015010	3
Indirect Effects	StressControl|Biocontrol	Universal Stress Response	Stress Signaling Proteins	Other Stress Response Proteins	yjgD->PGPT0015032	1
Indirect Effects	StressControl|Biocontrol	Universal Stress Response	Stress Signaling Proteins	Other Stress Response Proteins	ykgB|pgl->PGPT0014975	2
Indirect Effects	StressControl|Biocontrol	Universal Stress Response	Stress Signaling Proteins	Other Stress Response Proteins	yqhQ->PGPT0015034	1
Indirect Effects	StressControl|Biocontrol	Universal Stress Response	Stress Signaling Proteins	Other Stress Response Proteins	ytxH->PGPT0015027	4
Indirect Effects	StressControl|Biocontrol	Universal Stress Response	Stress Signaling Proteins	Other Stress Response Proteins	ytxJ->PGPT0015028	1
Indirect Effects	StressControl|Biocontrol	Universal Stress Response	Stress Signaling Proteins	Other Stress Response Proteins	yugU->PGPT0015021	1
Indirect	StressCon	Universal Stress	Stress Signaling	Other Stress Response	yvgN->PGPT0014985	2

Effects	trol|Biocontrol	Response	Proteins	Proteins		
Indirect Effects	StressControl|Biocontrol	Universal Stress Response	Stress Signaling Proteins	Stress Signal-Amino Acid Starvation Response	rpoS->PGPT0014685	1
Indirect Effects	StressControl|Biocontrol	Universal Stress Response	Stress Signaling Proteins	Stress Signal-Biofilm Formation Response	lacA->PGPT0014340	1
Indirect Effects	StressControl|Biocontrol	Universal Stress Response	Stress Signaling Proteins	Stress Signal-Carbohydrate Starvation Response	clp|crp->PGPT0015075	2
Indirect Effects	StressControl|Biocontrol	Universal Stress Response	Stress Signaling Proteins	Stress Signal-Carbohydrate Starvation Response	crr->PGPT0014090	1
Indirect Effects	StressControl|Biocontrol	Universal Stress Response	Stress Signaling Proteins	Stress Signal-Carbon Starvation Response	cstA->PGPT0015060	1
Indirect Effects	StressControl|Biocontrol	Universal Stress Response	Stress Signaling Proteins	Stress Signal-Cell Growth Signaling	abrB->PGPT0014790	6
Indirect Effects	StressControl|Biocontrol	Universal Stress Response	Stress Signaling Proteins	Stress Signal-Cell Growth Signaling	divIVA->PGPT0014805	1
Indirect Effects	StressControl|Biocontrol	Universal Stress Response	Stress Signaling Proteins	Stress Signal-Cell Growth Signaling	dnaA->PGPT0014800	1
Indirect Effects	StressControl|Biocontrol	Universal Stress Response	Stress Signaling Proteins	Stress Signal-Cell Growth Signaling	ftsW|spoVE->PGPT0014810	5
Indirect Effects	StressControl|Biocontrol	Universal Stress Response	Stress Signaling Proteins	Stress Signal-Cell Growth Signaling	gpsB->PGPT0014806	1

Indirect Effects	StressControl|Biocontrol	Universal Stress Response	Stress Signaling Proteins	Stress Signal-Cell Growth Signaling	sigH|sigG|sigF|sigE->PGPT0014795	8
Indirect Effects	StressControl|Biocontrol	Universal Stress Response	Stress Signaling Proteins	Stress Signal-Chaperone|Proteases	clpX->PGPT0014600	1
Indirect Effects	StressControl|Biocontrol	Universal Stress Response	Stress Signaling Proteins	Stress Signal-Chaperone|Proteases	cssR->PGPT0015125	1
Indirect Effects	StressControl|Biocontrol	Universal Stress Response	Stress Signaling Proteins	Stress Signal-Chaperone|Proteases	cssS->PGPT0015120	1
Indirect Effects	StressControl|Biocontrol	Universal Stress Response	Stress Signaling Proteins	Stress Signal-Chaperone|Proteases	degP|htrA->PGPT0015105	3
Indirect Effects	StressControl|Biocontrol	Universal Stress Response	Stress Signaling Proteins	Stress Signal-Chaperone|Proteases	groEL|mopA->PGPT0014570	1
Indirect Effects	StressControl|Biocontrol	Universal Stress Response	Stress Signaling Proteins	Stress Signal-Chaperone|Proteases	prc|ctpA->PGPT0015095	1
Indirect Effects	StressControl|Biocontrol	Universal Stress Response	Stress Signaling Proteins	Stress Signal-DNA Damage Defense|Repair	dinB->PGPT0014881	1
Indirect Effects	StressControl|Biocontrol	Universal Stress Response	Stress Signaling Proteins	Stress Signal-DNA Damage Defense|Repair	dps|dpsA->PGPT0004055	2
Indirect Effects	StressControl|Biocontrol	Universal Stress Response	Stress Signaling Proteins	Stress Signal-DNA Damage Defense|Repair	gph->PGPT0001730	3
Indirect Effects	StressControl|Bioco	Universal Stress Response	Stress Signaling Proteins	Stress Signal-DNA Damage	lexA->PGPT0014895	1

	ntrol			Defense|Repair		
Indirect Effects	StressControl|Biocontrol	Universal Stress Response	Stress Signaling Proteins	Stress Signal-DNA Damage Defense|Repair	radA->PGPT0014905	1
Indirect Effects	StressControl|Biocontrol	Universal Stress Response	Stress Signaling Proteins	Stress Signal-DNA Damage Defense|Repair	recA->PGPT0007235	3
Indirect Effects	StressControl|Biocontrol	Universal Stress Response	Stress Signaling Proteins	Stress Signal-DNA Damage Defense|Repair	umuC->PGPT0014882	5
Indirect Effects	StressControl|Biocontrol	Universal Stress Response	Stress Signaling Proteins	Stress Signal-DNA Damage Defense|Repair	uvrA->PGPT0014915	1
Indirect Effects	StressControl|Biocontrol	Universal Stress Response	Stress Signaling Proteins	Stress Signal-DNA Damage Defense|Repair	uvrB->PGPT0014920	1
Indirect Effects	StressControl|Biocontrol	Universal Stress Response	Stress Signaling Proteins	Stress Signal-DNA Damage Defense|Repair	uvrC->PGPT0014925	1
Indirect Effects	StressControl|Biocontrol	Universal Stress Response	Stress Signaling Proteins	Stress Signal-DNA Damage Defense|Repair	yugI->PGPT0014890	3
Indirect Effects	StressControl|Biocontrol	Universal Stress Response	Stress Signaling Proteins	Stress Signal-DNA Damage Defense|Repair	yxlJ|aag->PGPT0014885	1
Indirect Effects	StressControl|Biocontrol	Universal Stress Response	Stress Signaling Proteins	Stress Signal-Envelope Stress Response	baeS->PGPT0003985	1
Indirect Effects	StressControl|Biocontrol	Universal Stress Response	Stress Signaling Proteins	Stress Signal-Envelope Stress Response	liaR->PGPT0014870	4
Indirect	StressCon	Universal Stress	Stress Signaling	Stress Signal-Envelope	liaS->PGPT0014865	1

Effects	trol|Biocontrol	Response	Proteins	Stress Response		
Indirect Effects	StressControl|Biocontrol	Universal Stress Response	Stress Signaling Proteins	Stress Signal-Mutagenesis By DNA Polymerases	dinB->PGPT0014881	1
Indirect Effects	StressControl|Biocontrol	Universal Stress Response	Stress Signaling Proteins	Stress Signal-Mutagenesis By DNA Polymerases	umuC->PGPT0014882	5
Indirect Effects	StressControl|Biocontrol	Universal Stress Response	Stress Signaling Proteins	Stress Signal-Phosphate Starvation Response	phoH->PGPT0002700	1
Indirect Effects	StressControl|Biocontrol	Universal Stress Response	Stress Signaling Proteins	Stress Signal-Phosphate Starvation Response	psiE|yjbA->PGPT0015090	1
Indirect Effects	StressControl|Biocontrol	Universal Stress Response	Stress Signaling Proteins	Stress Signal-Phosphate Starvation Response	yxiE->PGPT0002681	4
Indirect Effects	StressControl|Biocontrol	Universal Stress Response	Stress Signaling Proteins	Stress Signal-Polyhydroxybutyrate Production	phaJ|croR->PGPT0014741	2
Indirect Effects	StressControl|Biocontrol	Universal Stress Response	Stress Signaling Proteins	Stress Signal-Polyhydroxybutyrate Production	phbB|phaB->PGPT0014735	1
Indirect Effects	StressControl|Biocontrol	Universal Stress Response	Stress Signaling Proteins	Stress Signal-Polyhydroxybutyrate Production	phbC|phaC->PGPT0014740	1
Indirect Effects	StressControl|Biocontrol	Universal Stress Response	Stress Signaling Proteins	Stress Signal-Sporulation Signaling	bOfC->PGPT0014765	1
Indirect Effects	StressControl|Biocontrol	Universal Stress Response	Stress Signaling Proteins	Stress Signal-Sporulation Signaling	disA->PGPT0014770	1

Indirect Effects	StressControl|Biocontrol	Universal Stress Response	Stress Signaling Proteins	Stress Signal-Sporulation Signaling	paiB|yumE->PGPT0014760	1
Indirect Effects	StressControl|Biocontrol	Universal Stress Response	Stress Signaling Proteins	Stress Signal-Sporulation Signaling	sinR->PGPT0014755	2
Indirect Effects	StressControl|Biocontrol	Universal Stress Response	Stress Signaling Proteins	Stress Signal-Sporulation Signaling	spo0A->PGPT0014750	1
Indirect Effects	StressControl|Biocontrol	Universal Stress Response	Stress Signaling Proteins	Stress Signal-Stringent Stress Response	ywaC|yjbM->PGPT0014825	1
Indirect Effects	StressControl|Biocontrol	Universal Stress Response	Stress Signaling Proteins	Stress Signal-Transcriptional Regulation	arsC->PGPT0004715	2
Indirect Effects	StressControl|Biocontrol	Universal Stress Response	Stress Signaling Proteins	Stress Signal-Transcriptional Regulation	arsC1->PGPT0004720	1
Indirect Effects	StressControl|Biocontrol	Universal Stress Response	Stress Signaling Proteins	Stress Signal-Transcriptional Regulation	rpoE|sigW->PGPT0014960	10
Indirect Effects	StressControl|Biocontrol	Universal Stress Response	Stress Signaling Proteins	Stress Signal-Transcriptional Regulation	rsbRD|yqhA->PGPT0014945	16
Indirect Effects	StressControl|Biocontrol	Universal Stress Response	Stress Signaling Proteins	Stress Signal-Transcriptional Regulation	rsbV->PGPT0014350	1
Indirect Effects	StressControl|Biocontrol	Universal Stress Response	Stress Signaling Proteins	Stress Signal-Transcriptional Regulation	rsbW->PGPT0014950	1
Indirect Effects	StressControl|Bioco	Universal Stress Response	Stress Signaling Proteins	Stress Signal-Transcriptional	rsbX->PGPT0014955	1

	ntrol			Regulation		
Indirect Effects	StressControl|Biocontrol	Universal Stress Response	Stress Signaling Proteins	Stress Signal-Transcriptional Regulation	yozG->PGPT0014940	3
Indirect Effects	StressControl|Biocontrol	Universal Stress Response	Stress Signaling Proteins	Stress Signal-Transcriptional Regulation	ytvA|pfyP->PGPT0014656	1
Putative PGPTs	PutativeFunctions	Putative Functions-1	Putative-Cell Fate Control	Putative-Cell Fate Control-1	pleD->PGPT0015900	2
Putative PGPTs	PutativeFunctions	Putative Functions-1	Putative-Energy Metabolism	Putative-Photosynthesis Electron Transfer	petB->PGPT0030840	1
Putative PGPTs	PutativeFunctions	Putative Functions-1	Putative-Energy Metabolism	Putative-Photosynthesis Electron Transfer	petC->PGPT0030830	1
Putative PGPTs	PutativeFunctions	Putative Functions-1	Putative-Oxidoreductase Activity	Putative-Oxidoreductase Activity-1	yqjQ->PGPT0030485	6
Putative PGPTs	PutativeFunctions	Putative Functions-1	Putative-Polycyclic Aromatic Hydrocarbon Catabolism	Putative-Polycyclic Aromatic Hydrocarbon Catabolism-1	phnA|yjdM->PGPT0030500	1
Putative PGPTs	PutativeFunctions	Putative Functions-1	Putative-Polycyclic Aromatic Hydrocarbon Catabolism	Putative-Polycyclic Aromatic Hydrocarbon Catabolism-2	phnB|yjdN->PGPT0030505	1
Putative PGPTs	PutativeFunctions	Putative Functions-1	Putative-Transamidase Activity	Putative-Transamidase Activity-1	gatB|yqeY->PGPT0030490	1
Putative PGPTs	PutativeFunctions	Putative Functions-1	Putative-Transcriptional Control	Putative-Transcriptional Control-1	greA->PGPT0030495	1
